# Synthesis and Organocatalytic Activity of Imidazolidinone Organocatalysts †

**DOI:** 10.3390/molecules31152684

**Published:** 2026-07-31

**Authors:** Nejc Petek, Luka Ciber, Amalija Golobič, Andrej Mihevc, Franc Požgan, Jurij Svete, Bogdan Štefane, Uroš Grošelj

**Affiliations:** Faculty of Chemistry and Chemical Technology, University of Ljubljana, Večna pot 113, SI-1000 Ljubljana, Slovenia; nejc.petek@fkkt.uni-lj.si (N.P.); luka.ciber@fkkt.uni-lj.si (L.C.); amalija.golobic@fkkt.uni-lj.si (A.G.); mihewc@gmail.com (A.M.); franc.pozgan@fkkt.uni-lj.si (F.P.); jurij.svete@fkkt.uni-lj.si (J.S.); bogdan.stefane@fkkt.uni-lj.si (B.Š.)

**Keywords:** asymmetric organocatalysis, imidazolidinone organocatalysts, iminium ion organocatalysis, reversal of stereoselectivity, enantioselectivity, self-regeneration of stereocenters (SRS)

## Abstract

A series of *cis*-5-arylmethyl-2-alkyl-3-methylimidazolidin-4-ones, prepared from arylmethyl-substituted α-amino acids, and a series of *trans*- and *cis*-2-(fluoromethyl)-2,3-dimethylimidazolidin-4-ones, derived from L-valine and L-leucine, were synthesized and fully characterized. Their catalytic activity was evaluated in the addition of 1-methylindole to cinnamaldehyde. Using the *cis*-5-arylmethyl-2-alkyl-3-methylimidazolidin-4-one catalysts, enantioselectivities of up to 78% *ee* (*S*) were achieved. Notably, with the L-valine-derived *cis*-imidazolidinone organocatalyst, the highest reversal of stereoselectivity to date (92% *ee*, (*R*) at –43 °C) was observed.

## 1. Introduction

Chiral imidazolidinones are a privileged class of heterocycles in stereoselective and asymmetric synthesis. In Dieter Seebach’s self-regeneration of stereocenters (SRS) concept, chiral imidazolidinones serve as conformationally defined chiral environments that efficiently transmit stereochemical information during the synthesis of chiral α-substituted amino acid derivatives [1]. A major conceptual advance occurred with the seminal work of David W. C. MacMillan, who demonstrated in 2000 that chiral imidazolidinones are highly effective organocatalysts, activating α,β-unsaturated aldehydes through iminium ion formation in a highly enantioselective organocatalytic Diels–Alder reaction [2]. The use of chiral imidazolidinones quickly expanded to other reactions, including Friedel–Crafts alkylation [3], intramolecular Michael additions [4,5], 1,3-dipolar cycloadditions [6], α-fluorination [7], and many others [8,9], establishing imidazolidinones as central scaffolds in asymmetric aminocatalysis and highlighting their role in making organocatalysis a general catalytic paradigm [10,11,12]. The use of imidazolidinone organocatalysts has expanded to the total synthesis of natural products [13,14,15,16] and has been adapted for recoverable, immobilized, and continuous-flow catalytic systems suitable for process-scale applications [17,18,19]. Importantly, chiral imidazolidinones have paved the way for asymmetric photoredox organocatalysis [9,20,21,22], SOMO catalysis [23], and various forms of synergistic catalysis [24,25].

**Figure 1:**
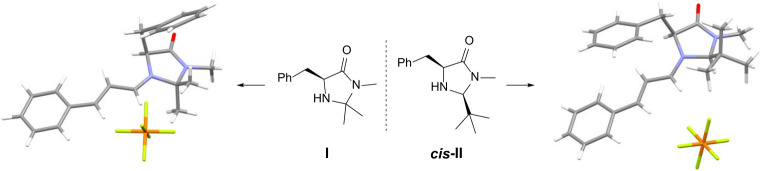
First-generation **I** and second-generation ***cis*-II** MacMillan organocatalysts, along with the experimentally determined cinnamaldehyde-derived iminium ion reactive intermediates.

Chiral imidazolidinone iminium ion reactive intermediates of 2-substituted 5-benzyl-3-methylimidazolidin-4-ones and cinnamaldehyde were prepared by Grošelj et al., and their X-ray crystal structures as PF_6_/BF_4_ salts were determined [26]. The structures closely matched the DFT-calculated structures [27]. Three distinct conformations were identified in the solid state, and two were confirmed in solution by NMR analysis. Additionally, subtle effects of the substitution pattern on the configuration of the exocyclic iminium bond ((*E*) or (*Z*)) and on the conformation of the benzylic bond in solution were elucidated by NMR analysis [26,27,28,29,30]. Figure 1 shows first-generation **I** [2] and second-generation ***cis*-II** [5] MacMillan organocatalysts, along with the experimentally determined cinnamaldehyde-derived iminium ion reactive intermediates [26].

**Figure 2:**
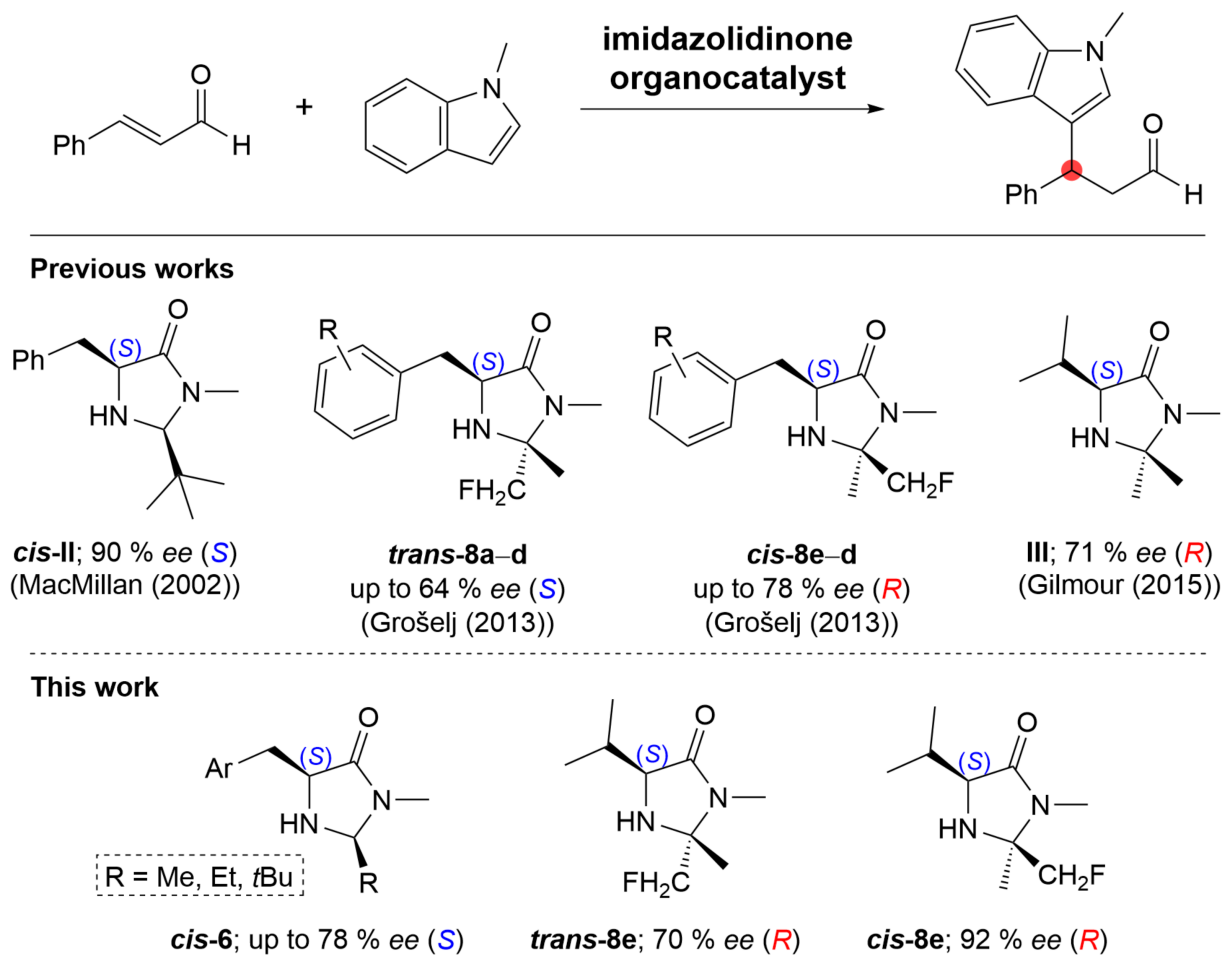
Addition of 1-methyl-1*H*-indole to cinnamaldehyde with imidazolidinone organocatalysts: previous works and current research, with emphasis on selectivity reversal using catalysts that share the same (*S*)-absolute configuration at position 5 of the imidazolidinone ring. MacMillan 2002 [5], Grošelj 2013 [34], Gilmour 2015 [35].

Gilmour et al. used the fluorine–iminium ion gauche effect to design conformational probes for imidazolidinone organocatalysis without introducing additional steric constraints. The predetermined configuration of the benzylic fluorine defined the topology of the iminium ion intermediate and provided insight into the noncovalent interactions important for catalyst design [31,32]. Later, the same group showed a correlation between the quadrupole moment of the aryl shielding group and catalytic selectivity, implicating a cation–π interaction as a contributing factor in the addition of uncharged nucleophiles to iminium salts derived from MacMillan’s first-generation catalyst [2,33]. Among the studied imidazolidinone organocatalysts, *cis*-5-(substituted)benzyl-2-(fluoromethyl)-2,3-dimethylimidazolidin-4-ones ***cis*-8a**–**d**, in which the *cis*-methyl group of the MacMillan first-generation catalyst **I** [2] is replaced by a CH_2_F group, reversed the product configuration from (*S*) to (*R*) absolute configuration (up to 78% *ee* (*R*)) in the addition of 1-methyl-1*H*-indole to cinnamaldehyde (Figure 2) [34]. For the same reaction, a simple L-valine imidazolidinone catalyst analog of the first-generation MacMillan organocatalyst **III** also showed a reversal of selectivity (71% *ee* (*R*)), explained by intermolecular aromatic (CH–π and cation–π) interactions between the incipient iminium cation and the indole ring (Figure 2) [35].

The catalytic activity of *trans*- and *cis*-5-(substituted)benzyl-2-(fluoromethyl)-2,3-dimethylimidazolidin-4-ones **8b**–**d** in the addition of 1-methylindole to cinnamaldehyde has previously been reported, but their synthesis and characterization were not described [34]. Here, we report the synthesis and full characterization of *trans*- and *cis*-5-(substituted)benzyl-2-(fluoromethyl)-2,3-dimethylimidazolidin-4-ones **8b**–**d**, as well as novel 5-arylmethyl-2-alkyl-3-methylimidazolidin-4-ones **6a**–**i** and L-valine-**8e**- and L-leucine-**8f**-derived 2-(fluoromethyl)-2,3-dimethylimidazolidin-4-ones. The catalytic activity of **6a**–**i**, **8e**, and **8f** in the addition of 1-methylindole to cinnamaldehyde was investigated. Notably, with the L-valine-derived imidazolidinone ***cis*-8e**, the highest reversal of stereoselectivity to date (92% *ee*, (*R*); at –43 °C) was observed, compared to the first-generation **I** (44% *ee*, (*S*); at –43 °C) and second-generation ***cis*-II** (90% *ee*, (*S*); at –61 °C) MacMillan organocatalysts (Figure 2).

## 2. Results and Discussion

**Scheme 1:**
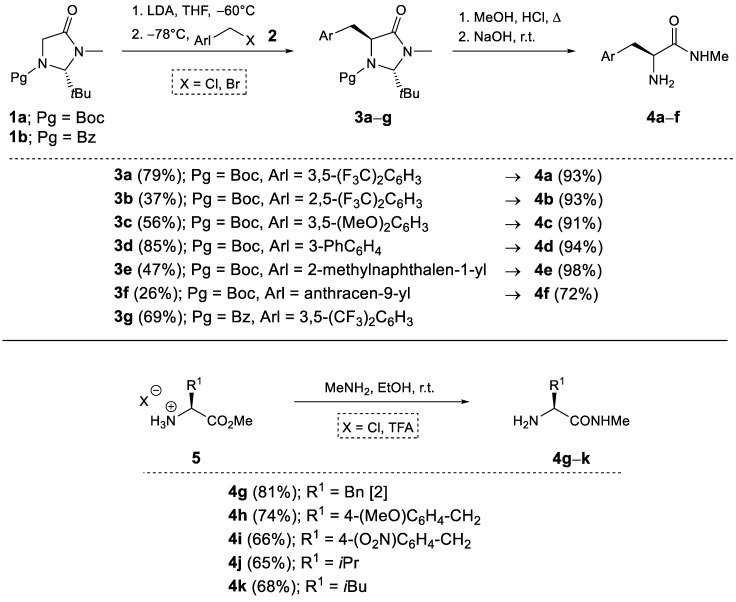
Synthesis of *N*-methyl amides **4a**–**k**.

*Synthesis*: All imidazolidinone organocatalysts reported here were prepared from (*S*)-configured α-amino acid *N*-methyl amides **4a**–**k**. Amino amides **4a**–**f** were synthesized in two steps from *tert*-butyl (*S*)-2-(*tert*-butyl)-3-methyl-4-oxoimidazolidine-1-carboxylate (**1a**) (Scheme 1). Low-temperature alkylation of **1a** with substituted benzyl bromides **2a**–**d**, 1-(chloromethyl)-2-methylnaphthalene (**2e**), and 9-(chloromethyl)anthracene (**2f**) at –78 °C using lithium diisopropylamide (LDA) as a base stereoselectively yielded the corresponding Boc-protected *trans*-2,5-disubstituted imidazolidinones **3a**–**f** in 26–85% yields. Similarly, alkylation of (*S*)-1-benzoyl-2-(*tert*-butyl)-3-methylimidazolidin-4-one (**1b**) with 1-(bromomethyl)-3,5-bis(trifluoromethyl)benzene (**2a**) gave imidazolidinone **3g** at a 69% yield. Subsequent hydrolysis of imidazolidinones **3a**–**f** under acidic conditions in a water/methanol mixture at elevated temperature, followed by neutralization, produced amino amides **4a**–**f** with 72–98% yields. In contrast, (substituted) L-phenylalanine **4g**–**i**, L-valine **4j**, and L-leucine-derived **4k** amino-*N*-methylamide derivatives were prepared from the corresponding methyl ester ammonium salts **5a**–**e** with 65–81% yields by neutralization with aqueous NaHCO_3_, followed by treatment of the free amine with methylamine in ethanol or, alternatively, by direct treatment with methylamine in ethanol followed by extraction with aqueous NaHCO_3_ (Scheme 1). For details, see the Supplementary Materials.

**Scheme 2:**
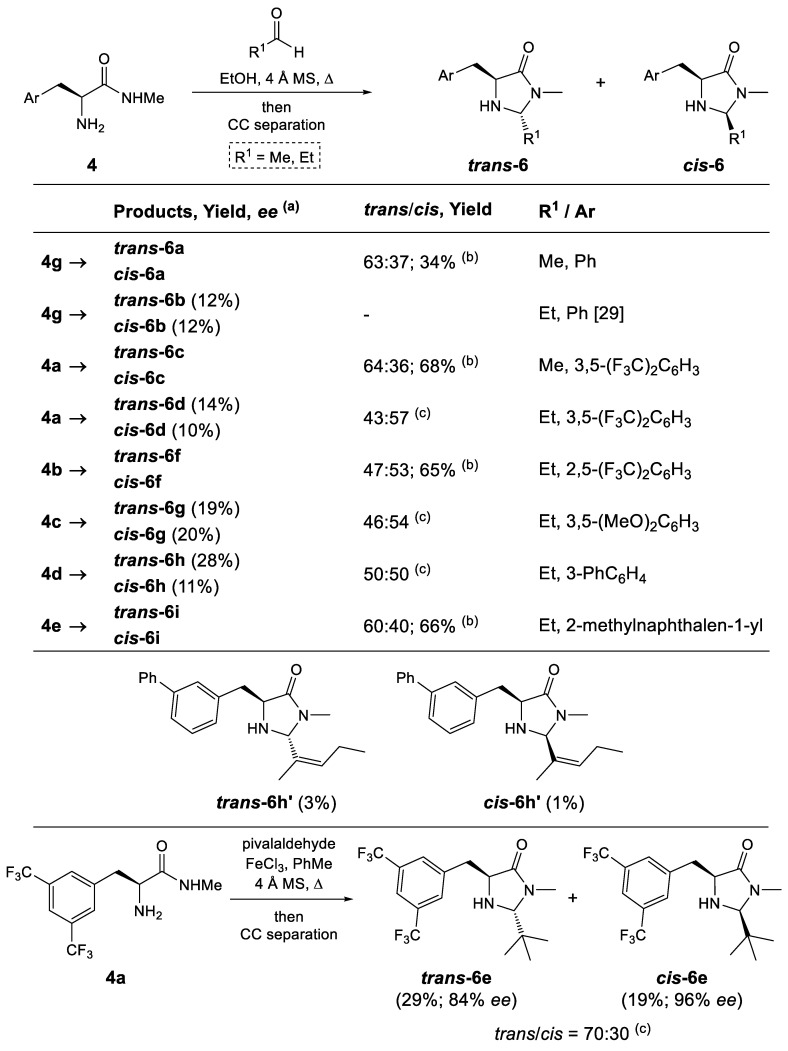
Synthesis of imidazolidinone organocatalysts **6a**–**i**, part 1. The products of individual reactions are listed in the order in which the separated products elute. ^(a)^ Isolated yield of pure isomer after column chromatography; ^(b)^ *cis*/*trans* ratio in CDCl_3_ after column chromatography; ^(c)^ *cis*/*trans* ratio in CDCl_3_ of the crude reaction mixture. For details, see the Supplementary Materials.

**Scheme 3:**
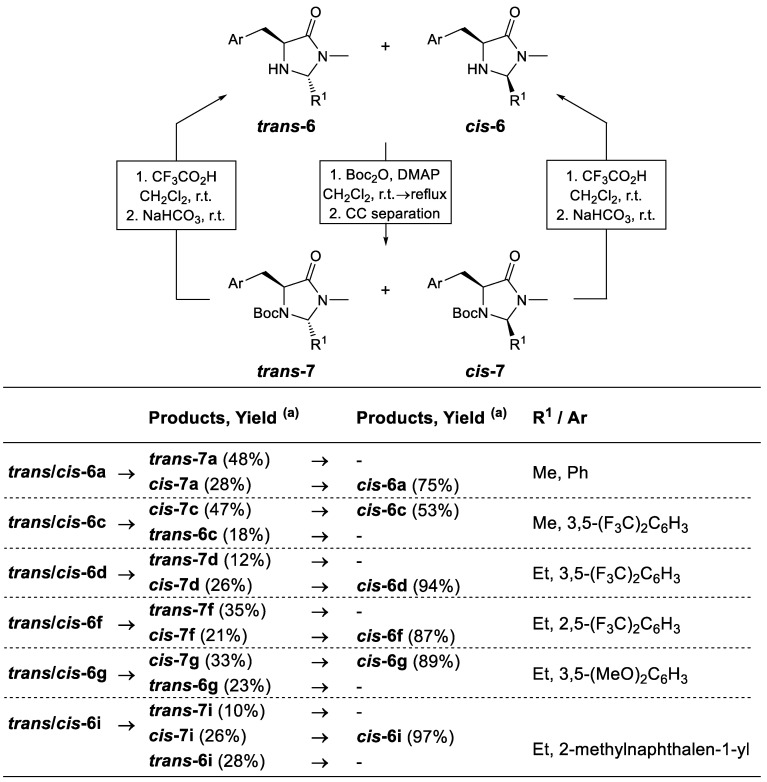
Synthesis of imidazolidinone organocatalysts **6a**,**c**,**d**,**f**,**g**,**h**, part 2. The products of individual reactions are listed in the order in which the separated products eluted. ^(a)^ Isolated yield of pure isomer after column chromatography. For details, see the Supplementary Materials.

**Scheme 4:**
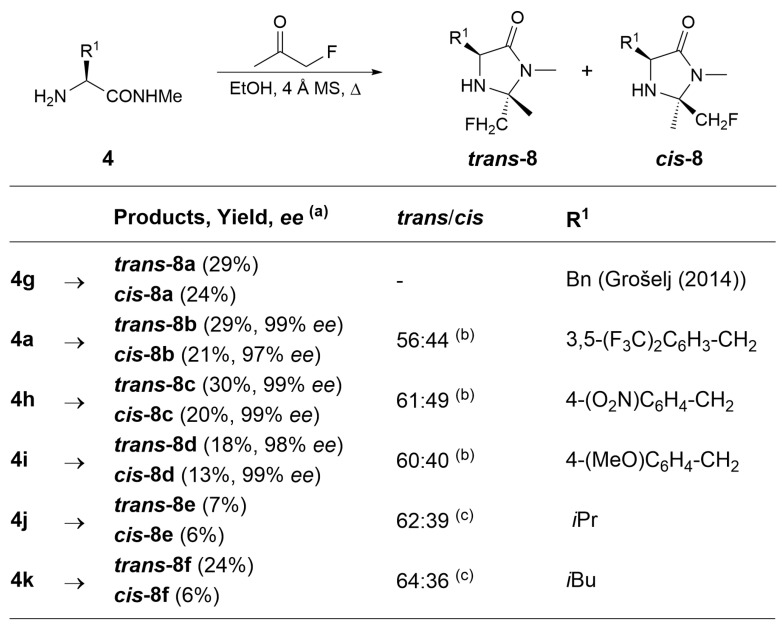
Synthesis of imidazolidinone organocatalysts 8a–f. The products of individual reactions are listed in the order in which the separated products eluted. ^(a)^ Isolated yield of pure isomer after column chromatography; ^(b)^ cis/trans ratio in CDCl3 after column chromatography; ^(c)^ cis/trans ratio in CDCl3 of the crude reaction mixture. For details, see the Supplementary Materials. Grošelj 2014 [29].

Next, amino amides **4a**–**e** and **4g**–**k** were cyclized to the corresponding imidazolidinone organocatalysts using selected aldehydes and a ketone (Scheme 2, Scheme 3 and Scheme 4). Treating (*S*)-arylmethyl-amino amides **4a**–**e** and **4g** with acetaldehyde, propionaldehyde, or pivalaldehyde in the presence of 4 Å molecular sieves—and FeCl_3_ in the case of pivalaldehyde—using a Dean–Stark apparatus produced the corresponding mixtures of *trans*- and *cis*-5-arylmethyl-2-alkyl-3-methylimidazolidin-4-ones, ***trans*-6a**–**i** and ***cis*-6a**–**i**, at ratios ranging from 70:30 to 43:57, respectively (Scheme 2). In the cyclization of amino amide **4d** with propionaldehyde, in addition to the expected imidazolidinones ***trans*-6h** and ***cis*-6h**, 2-(pent-2-en-2-yl)-substituted imidazolidinones ***trans*-6h′** and ***cis*-6h′** were also isolated at very low yields (3% and 1%, respectively). These products formed via the condensation of amino amide **4d** with (*Z*)-2-methylpent-2-enal, which was generated in situ from propionaldehyde. The ***trans*-6b**,**d**,**e**,**g**,**h** and ***cis*-6b**,**d**,**e**,**g**,**h** geometric isomers were successfully separated by column chromatography and isolated at 10–29% yields (Scheme 2).

Stereoisomers ***trans*-6a**,**c**,**d**,**f**,**g**,**i** and ***cis*-6a**,**c**,**d**,**f**,**g**,**i**, which could not be (fully) separated by column chromatography, were *N*-Boc-protected using Boc_2_O in the presence of triethylamine and DMAP, and then separated by column chromatography (Scheme 3). For details, see the Supplementary Materials. Thus, compounds ***trans*-7a**,**d**,**f**,**i** and ***cis*-7a**,**c**,**d**,**f**,**g**,**i** were isolated with 12–48% yields. Notably, when mixtures of ***trans*-6c**,**g**,**i** and ***cis*-6c**,**g**,**i** were treated with excess Boc_2_O at elevated temperature for an extended period, only the *cis*-isomers (***cis*-6c**,**g**,**i**) were selectively Boc-protected, while the *trans*-isomers were either not protected (***trans*-6c**,**g**) or only partially protected (***trans*-6i**). A simple conformational analysis shows that the amino group (lone electron pair) of *cis*-disubstituted imidazolidinone isomers **6**, which are partially planarized by the carbonyl group, is much less hindered than in the corresponding *trans* isomers, where the lone pair on nitrogen is always *cis* to one of the substituents in the α position. This is probably the main reason for the observed preference in the *N*-Boc protection reaction, a kinetic effect. The *N*-Boc-protected *cis*-imidazolidinones **7** are also thermodynamically more stable than their *trans* isomers, by approximately 2.3 kcal/mol, as calculated by MM2 energy minimization in Chem3D for compounds ***cis*-7a**/***trans*-7a**. Subsequent treatment of ***cis*-7a**,**c**,**d**,**f**,**g**,**i** with trifluoroacetic acid, followed by neutralization with NaHCO_3_, yielded the *cis*-imidazolidinones **6a**,**c**,**d**,**f**,**g**,**i** at 53–97% yields (Scheme 3).

Similarly, treatment of (substituted) *L*-phenylalanine **4a**,**g**–**i**, L-valine **4j**, and L-leucine-derived **4k** amino-*N*-methylamide derivatives with fluoroacetone yielded mixtures of *trans*- and *cis*-5-substituted-2-(fluoromethyl)-2,3-dimethylimidazolidin-4-ones ***trans*-8a**–**f** and ***cis*-8a**–**f**, at ratios ranging from 56:44 to 64:36, respectively. The ***trans*-8a**–**f** and ***cis*-8a**–**f** geometrical isomers were separated by column chromatography and isolated at 6–30% yields (Scheme 4).

**Scheme 5:**
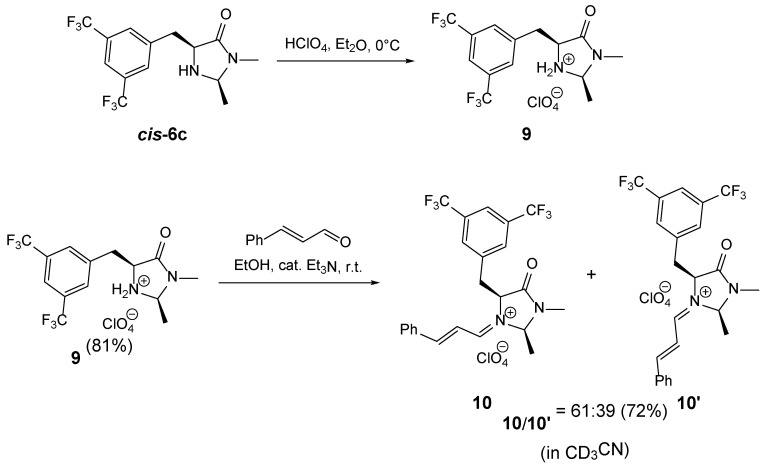
Synthesis of ammonium salt **9** and iminium salt **10**/**10′**.

Finally, (2*S*,5*S*)-5-(3,5-bis(trifluoromethyl)benzyl)-2,3-dimethylimidazolidin-4-one (***cis*-6c**) was converted to ammonium perchlorate **9** with an 81% yield by treatment with HClO_4_ in diethyl ether. Subsequent treatment of ammonium salt **9** with cinnamaldehyde and triethylamine in anhydrous ethanol gave the corresponding iminium salt at a 72% yield as a mixture of major (*E*,*E*)-**10** and minor (*Z*,*E*)-**10′** geometric isomers at a 61:39 ratio in CD_3_CN (Scheme 5).

**Figure 3:**
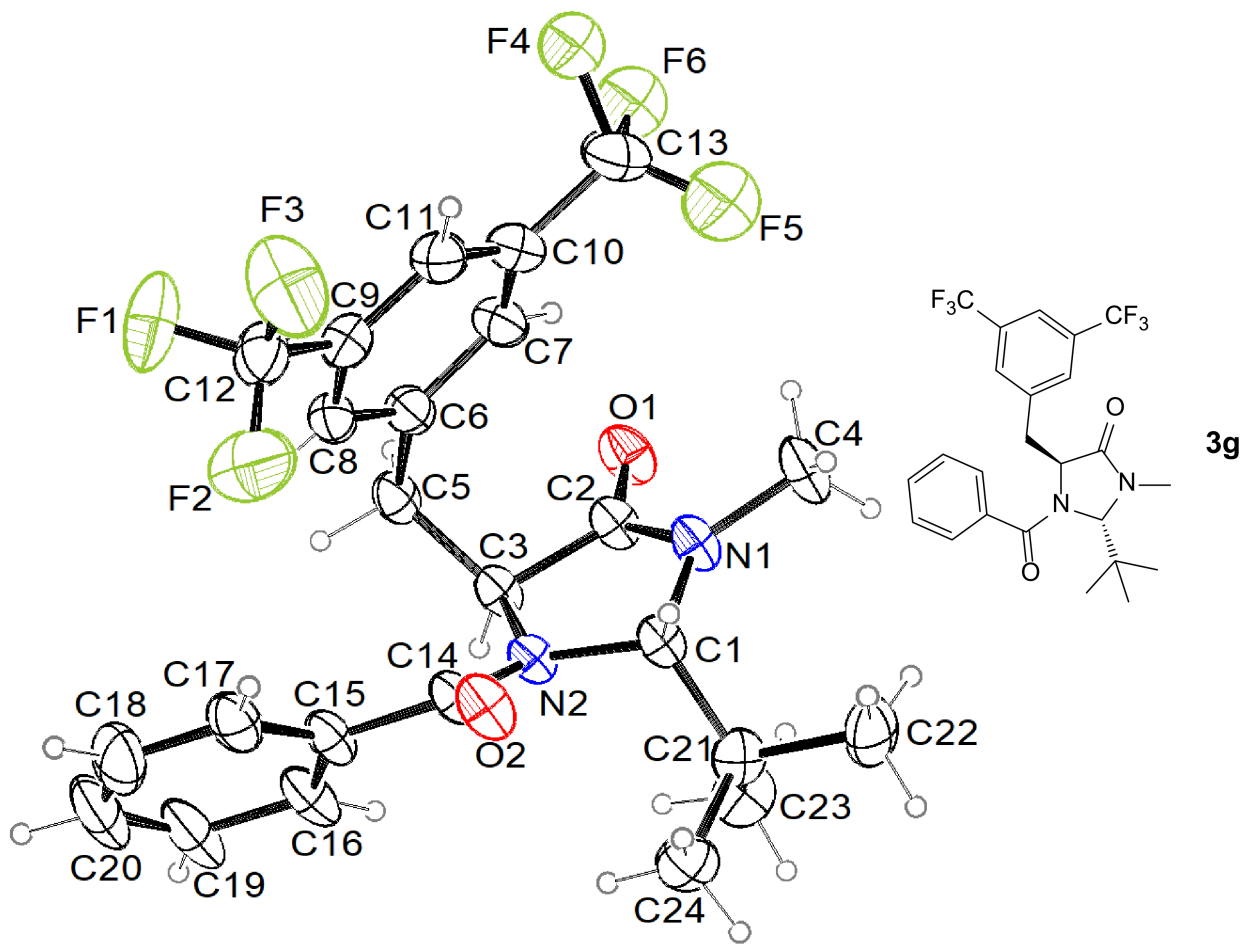
Molecular structure of product **3g**. Crystal Data: C_24_H_24_F_6_N_2_O_2_ (M = 486.45 g/mol): monoclinic, space group *C*2 (no. 5), a = 32.4006(14) Å, b = 6.0403(2) Å, c = 12.4330(7) Å, β = 91.003(4)°, V = 2432.88(19) Å^3^, Z = 4, T = 293(1) K, μ(CuKα) = 1.005 mm^−1^, D_calc_ = 1.328 g/cm^3^; 13,791 reflections measured (7.112° ≤ 2Θ ≤ 150.34°), 4951 unique (R_int_ = 0.0384), which were used in all calculations; Flack parameter = 0.08(18); the final R1 was 0.0886 (I > 2σ(I)) and wR2 was 0.2820 (all data).

**Figure 4:**
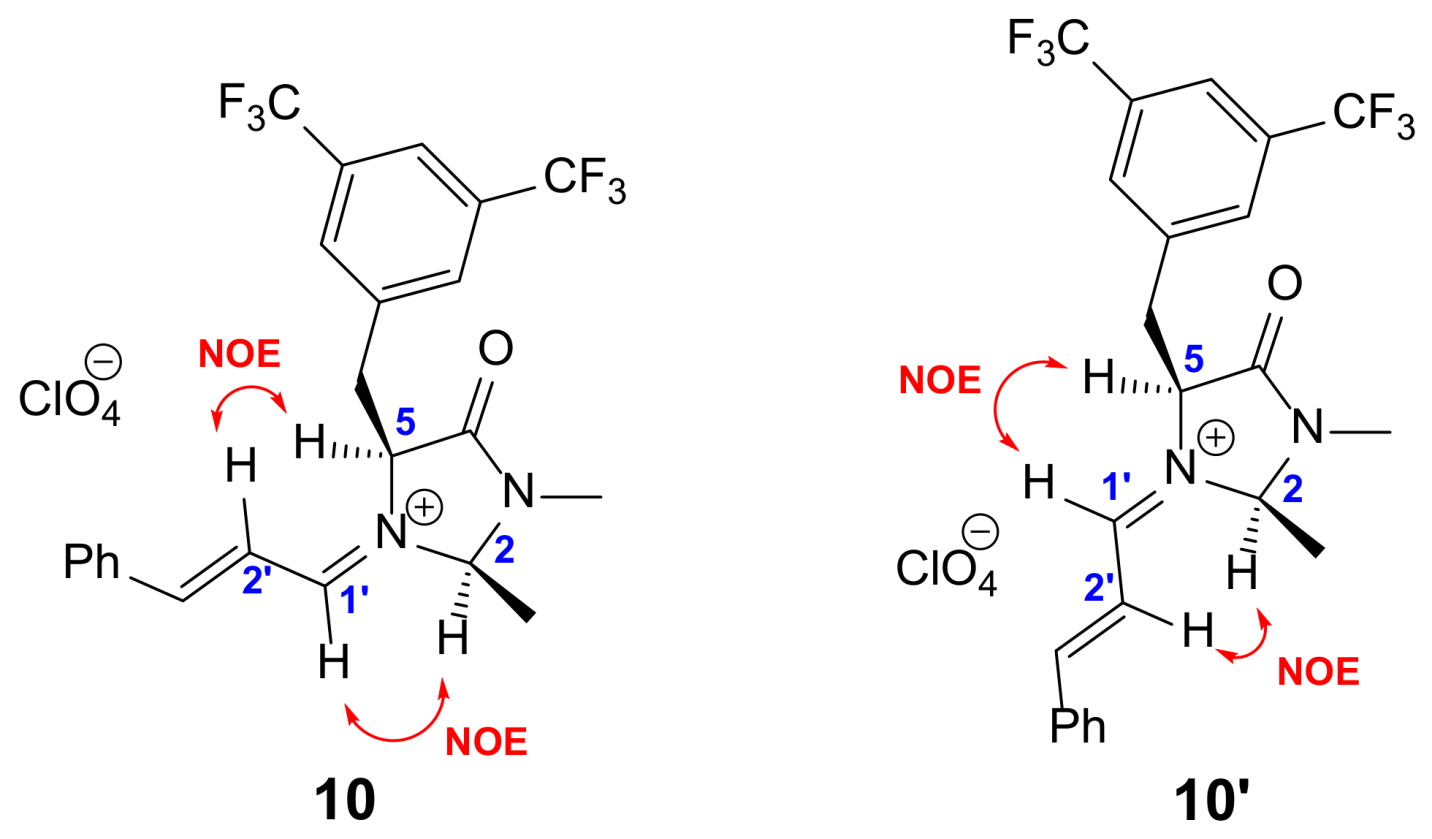
The key NOEs crucial for determining the configuration of the exocyclic double bonds in (*E*,*E*)-**10** and (*Z*,*E*)-**10′**.

*Structure Determination:* The structures of the novel compounds were confirmed by spectroscopic methods (^1^H and ^13^C NMR, 2D NMR, IR) and high-resolution mass spectrometry. The diastereomeric ratios of the compounds were determined by proton spectra, integrating the non-overlapping signals. The structure and (2*S*,5*S*)-absolute configuration of product **3g** were confirmed by single-crystal X-ray diffraction analysis (Figure 3). The (*E*,*E*)-configuration around the exocyclic double bonds of the major iminium ion **10** was confirmed by the NOE signal in the NOESY spectra between H-C(5) and H-C(2′), and between H-C(2) and H-C(1′). Similarly, the NOE signal between H-C(5) and H-C(1′), and between H-C(2) and H-C(2′), confirmed the (*Z*,*E*)-configuration of the minor isomer **10′** (Figure 4). For details, see the Supplementary Materials. Unfortunately, NOESY measurements of imidazolidinones **6** and **8** failed to confirm the *cis* or *trans* geometry.

As a rule of thumb, the *trans*-isomers, ***trans*-6** and ***trans*-8**, elute first from the column, followed by the *cis*-isomers, ***cis*-6** and ***cis*-8**, as observed in our previous publication [29]. This can tentatively be attributed to the lone pair on the nitrogen of the *cis*-isomers being available for interaction with the stationary phase, whereas in the *trans*-isomers, the nitrogen electron pair is shielded on both sides of the five-membered heterocycle. In addition, the key proton chemical shifts used to correlate the *cis*- and *trans*-isomers of the final catalysts **6** and **8** are presented in Table 1. The chemical shifts for the previously published compounds ***cis*-6b**, ***trans*-6b**, ***cis*-8b**, ***trans*-8b**, and ***cis*-II** are provided as references [29]. There is a correlation for isomers ***cis*-6a**–**g** in the proton spectra, with proton H–C(5) ranging from 3.70 to 3.86 ppm and proton H–C(2) ranging from 4.05 to 4.47 ppm. In the corresponding ***trans*-6b**,**c**,**d**,**e**,**g**,**h** isomers, H–C(5) is shifted downfield by 0.03 to 0.14 ppm, ranging from 3.81 to 3.93 ppm, while H–C(2) is shifted upfield by 0.15 to 0.45 ppm, ranging from 3.76 to 4.15 ppm. Only the naphthalene-1-yl-substituted catalysts ***cis*-6i** and ***trans*-6i** do not follow this correlation. Similarly, there is a correlation for the geminal CH_2_F protons of the 5-benzyl-substituted isomers ***cis*-8a**–**d**, which appear at 3.95–4.02 ppm and 4.14–4.18 ppm, respectively. In the corresponding ***trans*-8a**–**d** isomers, the geminal CH_2_F protons are shifted downfield by 0.14–0.20 ppm, appearing at 4.13–4.21 ppm and 4.29–4.37 ppm, respectively. This correlation does not apply to the 5-alkyl-substituted catalysts ***cis*-8e**,**f** and ***trans*-8e**,**f** (Table 1).

**Scheme 6:**
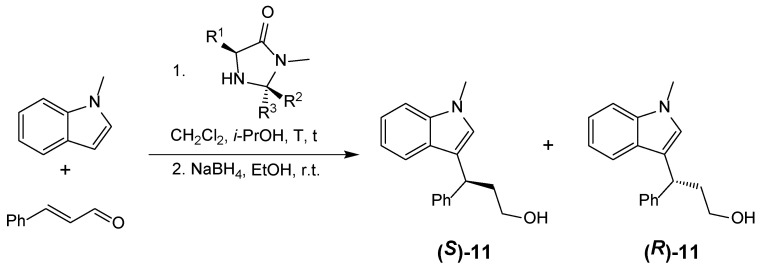
Asymmetric organocatalyzed addition of 1-methyl-1*H*-indole to cinnamaldehyde, followed by reduction and determination of enantiomeric excess.

*Organocatalysis*: The catalytic activity of organocatalysts **6** and **8** was evaluated in the addition of 1-methyl-1*H*-indole to cinnamaldehyde to produce 3-indolyl-3-phenylpropanal and, after reduction, the corresponding alcohol **11** (Scheme 6, Table 2) [9]. Table 2 also includes results for the previously reported activity of the catalysts ***cis*-8b**, ***trans*-8b**, and **III**. For comparison, the catalytic activity of catalysts **I** and ***cis*-II** was re-evaluated. The Supplementary Materials include all chromatograms for the previously reported catalytic activities of catalysts ***cis*-8a**–**d** and ***trans*-8a**–**d** [34]. Of the newly synthesized compounds, catalysts ***trans*-6c**,**d**, ***cis*-6a**–**i**, ***trans*-8e**,**f**, and ***cis*-8e**,**f** were evaluated (Table 2). The *trans*-2-alkyl-substituted catalysts ***trans*-6c**,**d** showed both incomplete conversion and very low enantioselectivity (up to 6% *ee*, (*R*), Entries 1 and 2). In the *cis*-5-arylmethyl-2-alkyl-3-methylimidazolidin-4-one series ***cis*-6a**–**i**, (*S*)-enantioselectivities from 27% *ee* to 78% *ee* were achieved (Entries 3–16). Catalyst ***cis*-6e**, featuring 2-*tert*-butyl and 5-(3,5-trifluoromethyl)benzyl substituents, gave the product with the highest enantioselectivity (78% *ee*, (*S*), at –41 °C) at full conversion (Entry 12), coming closest in enantioselectivity to the MacMillan second-generation catalyst ***cis*-II** (Entry 9, 87% *ee*, (*S*), at –41 °C, full conversion). The substituted catalysts ***cis*-6a**–**d**,**f**–**i**, with 2-methyl and 2-ethyl substituents, were inferior to the 2-*tert*-butyl-substituted catalyst ***cis*-6e**. In the *cis*-5-arylmethyl-2-ethyl/2-methyl series of catalysts ***cis*-6a**–**d**,**f**–**i**, the highest enantioselectivity was achieved with catalyst ***cis*-6i**, bearing the 2-methylnaphthalen-1-yl substituent at position 5 (Entry 16, 69% *ee*, (*S*), at –42 °C, full conversion).

In the 5-alkyl-2-(fluoromethyl)-2,3-dimethylimidazolidin-4-one series, the ***trans*-8e**,**f** catalysts gave the product with up to 70% *ee* at –43 °C with reversed (*R*)-stereoinduction (Entries 20 and 21), as expected from the results of Gilmour’s L-valine-derived catalyst **III** (Entry 18, 71% *ee* (*R*)) [35]. The L-valine-derived *cis*-imidazolidinone organocatalyst ***cis*-8e** (Entry 24) exhibited the highest reversal of enantioselectivity with full conversion to date (92% *ee*, (*R*), at –43 °C), surpassing the enantioselectivity achieved by the second-generation MacMillan organocatalyst ***cis*-II** (90% *ee*, (*S*)) [5], as well as catalyst **III** (Entry 18, 71% *ee* (*R*)) [35] and our previously reported catalyst ***cis*-8b** (Entry 22, 78% *ee*, (*R*), at –41 °C, full conversion) [29]. The L-leucine-derived catalyst ***cis*-8f** gave the product with incomplete conversion and 85% *ee* (*R*) at –43 °C (Entry 26). Temperature significantly affected enantioselectivity and conversion, as shown by the results for catalyst ***cis*-II** (Entries 8–10: room temperature, 72% *ee* (*S*); –41 °C, 87% *ee* (*S*), both with full conversion; –61 °C, 90% *ee* (*S*), partial conversion) and catalyst ***cis*-8e** (Entries 23–25: room temperature, 67% *ee* (*R*); –43 °C, 92% *ee* (*R*), both with full conversion; –63 °C, 93% *ee* (*S*), partial conversion).

The reversed stereoselectivity observed with catalysts ***cis*-8e** and ***cis*-8f**, as well as ***trans*-8e** and ***trans*-8f**, can be tentatively rationalized by intermolecular aromatic (CH–π and cation–π) interactions between the incipient iminium cation and the indole ring, as proposed by Gilmour et al. for the closely related catalyst **III** [35].

## 3. Materials and Methods

All reactions were performed under argon in dried glassware using anhydrous solvents, except when aqueous reagents were used. Solvents used for extractions and chromatography were of technical grade and distilled before use. Extracts were dried over technical-grade anhydrous Na_2_SO_4_. Melting points were determined using a Kofler micro hot stage and an SRS OptiMelt MPA100 Automated Melting Point System (Stanford Research Systems, Sunnyvale, CA, USA). NMR spectra were recorded on a Bruker Avance DPX spectrometer and a Bruker UltraShield 500 plus spectrometer (Bruker, Billerica, Massachusetts, USA) at 300 and 500 MHz for ^1^H and at 75.5 and 126 MHz for ^13^C, respectively, using DMSO-*d*_6_, CD_3_CN, and CDCl_3_ as solvents, with TMS as the internal standard. Microanalyses for C, H, and N were performed on a PerkinElmer CHNS/O Analyzer 2400 Series II (PerkinElmer, Waltham, MA, USA). Mass spectra were recorded on an Agilent 6224 Accurate Mass TOF LC/MS (Agilent Technologies, Santa Clara, CA, USA), and IR spectra on a PerkinElmer Spectrum BX FTIR spectrophotometer (PerkinElmer, Waltham, MA, USA). Column chromatography was performed on silica gel (Silica gel 60, particle size 0.035–0.070 mm; Sigma-Aldrich, St. Louis, MO, USA). Medium-pressure liquid chromatography (MPLC) was performed using a Büchi Flash Chromatography System (Büchi Fraction Collector C-660, Büchi Pump Module C-605, Büchi Control Unit C-620) on silica gel (LiChrosphere^®^ Si 60, 12 µm, and/or LiChroprep^®^ Si 60, 12–15 µm); column dimensions (wet filled): 22 × 460 mm, 36 × 460 mm, and 40 × 460 mm; backpressure: 10–20 Bar; detection: UV 254 nm (BÜCHI Labortechnik AG, Flawil, Switzerland). HPLC analyses were performed on an Agilent 1260 Infinity LC (Agilent Technologies, Santa Clara, CA, USA) using Chiralpak AD-H (0.46 cm ø × 25 cm) and Chiralcel OD-H (0.46 cm ø × 25 cm) chiral columns (CHIRAL TECHNOLOGIES, INC., West Chester, PA, USA). Optical rotations were measured on a PerkinElmer 241MC Polarimeter (PerkinElmer, Waltham, MA, USA). Low reaction temperatures were maintained using a Julabo FT902 immersion cooler (JULABO GmbH, Seelbach, Germany). All commercially available chemicals were obtained from Sigma-Aldrich (St. Louis, MO, USA).

(*S*)-2-Amino-*N*-methyl-3-phenylpropanamide (**4g**) [36], (*S*)-5-benzyl-2,2,3-trimethylimidazolidin-4-one (**I**) [29], (2*S*,5*S*)-5-benzyl-2-(*tert*-butyl)-3-methylimidazolidin-4-one (**II**) [29], (2*R*,5*S*)-5-benzyl-2-ethyl-3-methylimidazolidin-4-one (***trans*-6b**) [29], (2*S*,5*S*)-5-benzyl-2-ethyl-3-methylimidazolidin-4-one (***cis*-6b**) [29], (2*R*,5*S*)-5-benzyl-2-(fluoromethyl)-2,3-dimethylimidazolidin-4-one (***trans*-8a**) [29], and (2*S*,5*S*)-5-benzyl-2-(fluoromethyl)-2,3-dimethylimidazolidin-4-one (***cis*-8a**) [29] were prepared following procedures described in the literature.

*Alkylation of (*S*)-*N*-protected-2-(*tert*-butyl)-3-methylimidazolidin-4-ones—General Procedure 1 (GP1)*

To a solution of LDA (2.0 M in THF/heptane/ethylbenzene, 1.1 equiv.) cooled to –60 °C under argon, a solution of (*S*)-*N*-protected-2-(*tert*-butyl)-3-methylimidazolidin-4-one **1** (1 equiv.) in anhydrous THF (V_1_) was added over 15 min. The resulting mixture was stirred at –60 °C for 1 h, then cooled to –78 °C, and a solution of alkylating reagent **2** (1.1 equiv.) in anhydrous THF (V_2_) was added over 15 min. The mixture was stirred at –78 °C for 4 h and then quenched with saturated aqueous NH_4_Cl and warmed to room temperature. The reaction mixture was diluted with EtOAc and washed sequentially with aqueous NaHSO_4_ (1 M), saturated aqueous NaHCO_3_, and saturated aqueous NaCl. The organic phase was dried over anhydrous Na_2_SO_4_, filtered, and the volatiles were evaporated in vacuo. The residue was purified by column chromatography (EtOAc/petroleum ether) to yield alkylated imidazolidinone **3**.

*Synthesis of* tert*-butyl (2*S*,5*S*)-5-(3,5-bis(trifluoromethyl)benzyl)-2-(*tert*-butyl)-3-methyl-4-oxoimidazolidine-1-carboxylate (***3a***)*

Following *GP1*: Prepared from LDA (22 mmol, 11 mL), *tert*-butyl (*S*)-2-(*tert*-butyl)-3-methyl-4-oxoimidazolidine-1-carboxylate (**1a**) (20 mmol, 5.127 g), THF (V_1_ = 30 mL), 1-(bromomethyl)-3,5-bis(trifluoromethyl)benzene (**2a**) (22 mmol, ω = 0.97, 6.964 g (4.157 mL)), THF (V_2_ = 10 mL), NH_4_Cl (50 mL), EtOAc (300 mL), NaHSO_4_ (90 mL), NaHCO_3_ (150 mL), and NaCl (100 mL); isolation by column chromatography (petroleum ether/EtOAc = 6:1). Yield: 7.623 g (15.80 mmol, 79%) of white solid; m.p. = 92–94 °C. [*α*]_D_^r.t.^ = +17.9 (c = 2.0 mg/mL, CH_2_Cl_2_). EI-HRMS: *m/z* = 483.2061 (MH^+^); C_22_H_29_F_6_N_2_O_3_ requires *m/z* = 483.2077 (MH^+^). CHN microanalysis: C_22_H_28_F_6_N_2_O_3_ requires C, 54.77; H, 5.85; N, 5.81. Found: C, 54.73; H, 5.80; N, 5.82. *ν*_max_ 3457, 2970, 1698, 1484, 1458, 1443, 1413, 1383, 1366, 1346, 1332, 1279, 1252, 1174, 1129, 1032, 996, 956, 900, 846, 776, 755, 724, 709, 683 cm^−1^. ^1^H-NMR (300 MHz, CDCl_3_): *δ* 0.95 (*s*, *t*Bu), 1.44 (s, *t*Bu), 2.86 (*s*, NMe), 3.36 (*dd*, *J* = 2.1; 14.2 Hz, 1H, CH_2_), 3.97 (br *s*, 1H, CH_2_), 4.34–4.39 (br *m*, CH), 4.59 (*s*, CH), 7.64 (*s*, 2 arom. H), 7.71 (*s*, 1 arom. H). ^13^C-NMR (75 MHz, CDCl_3_): *δ* 26.8, 28.2, 31.9, 41.1, 60.2, 81.4, 81.9, 120.7–121.0 (*m*), 123.6 (*q*, *J* = 272.5 Hz), 130.3–130.6 (*m*), 131.3 (*q*, *J* = 33.0 Hz), 139.1, 152.5, 171.1 (one signal missing).

*Synthesis of* tert*-butyl (2*S*,5*S*)-5-(2,5-bis(trifluoromethyl)benzyl)-2-(*tert*-butyl)-3-methyl-4-oxoimidazolidine-1-carboxylate (***3b***)*

Following *GP1*: Prepared from LDA (16 mmol, 8 mL), *tert*-butyl (*S*)-2-(*tert*-butyl)-3-methyl-4-oxoimidazolidine-1-carboxylate (**1a**) (14.545 mmol, 3.729 g), THF (V_1_ = 20 mL), 2-(bromomethyl)-1,4-bis(trifluoromethyl)benzene (**2b**) (16 mmol, ω = 0.97, 5.064 g (3.025 mL)), THF (V_2_ = 5 mL), NH_4_Cl (50 mL), EtOAc (300 mL), NaHSO_4_ (80 mL), NaHCO_3_ (100 mL), and NaCl (100 mL); isolation by column chromatography (petroleum ether/EtOAc = 5:1). Yield: 2.586 g (5.382 mmol, 37%) of white solid; m.p. = 95–98 °C. [*α*]_D_^r.t.^ = +12.99 (c = 4.75 mg/mL, CH_2_Cl_2_). EI-HRMS: *m/z* = 483.2081 (MH^+^); C_22_H_29_F_6_N_2_O_3_ requires *m/z* = 483.2077 (MH^+^). CHN microanalysis: C_22_H_28_F_6_N_2_O_3_ requires C, 54.77; H, 5.85; N, 5.81. Found: C, 55.01; H, 6.01; N, 5.85. *ν*_max_ 2969, 1698, 1482, 1461, 1433, 1417, 1388, 1367, 1333, 1300, 1259, 1160, 1123, 1094, 1039, 1022, 980, 956, 920, 891, 850, 799, 772, 755, 737, 710, 663, 622 cm^−1^. ^1^H-NMR (500 MHz, CDCl_3_): *δ* 1.02 (*s*, 9H), 1.23 (*s*, 9H), 3.05 (*s*, 3H), 3.70 (*dd*, *J* = 3.5, 17.2 Hz, 1H), 3.87 (br *d*, *J* = 13.2 Hz, 1H), 4.44 (*t*, *J* = 4.8 Hz, 1H), 5.06 (*s*, 1H), 7.30 (*s*, 1H), 7.54 (*d*, *J* = 8.2 Hz, 1H), 7.76 (*d*, *J* = 8.2 Hz, 1H). ^13^C-NMR (126 MHz, CDCl_3_): *δ* 26.49, 27.87, 30.45, 32.18, 40.83, 57.95, 81.40, 81.59, 123.01 (*q*, *J* = 3.8 Hz), 123.74 (*q*, *J* = 274.6 Hz), 123.57 (*q*, *J* = 272.6 Hz), 124.86 (*d*, *J* = 6.5 Hz), 127.10 (*q*, *J* = 6.0 Hz), 132.42 (*q*, *J* = 31.2 Hz), 133.46 (*q*, *J* = 32.5 Hz), 138.03, 152.65, 171.39.

*Synthesis of* tert*-butyl (2*S*,5*S*)-2-(*tert*-butyl)-5-(3,5-dimethoxybenzyl)-3-methyl-4-oxoimidazolidine-1-carboxylate (***3c***)* [33]

Following *GP1*: Prepared from LDA (21 mmol, 10.5 mL), *tert*-butyl (*S*)-2-(*tert*-butyl)-3-methyl-4-oxoimidazolidine-1-carboxylate (**1a**) (19.091 mmol, 4.894 g), THF (V_1_ = 20 mL), 1-(bromomethyl)-3,5-dimethoxybenzene (**2c**) (21 mmol, ω = 0.95, 5.108 g), THF (V_2_ = 5 mL), NH_4_Cl (80 mL), EtOAc (450 mL), NaHSO_4_ (120 mL), NaHCO_3_ (150 mL), and NaCl (150 mL); isolation by column chromatography (1. petroleum ether/EtOAc = 5:1 then 2. petroleum ether/EtOAc = 2:1). Yield: 4.346 g (10.691 mmol, 56%) of white solid; m.p. = 83–86 °C. [*α*]_D_^r.t.^ = +33.6 (c = 3.55 mg/mL, CH_2_Cl_2_). EI-HRMS: *m/z* = 407.2537 (MH^+^); C_22_H_35_N_2_O_5_ requires *m/z* = 407.254 (MH^+^). CHN microanalysis: C_22_H_34_N_2_O_5_ requires C, 65.00; H, 8.43; N, 6.89. Found: C, 65.00; H, 8.64; N, 6.61. *ν*_max_ 2970, 2838, 1692, 1593, 1456, 1430, 114,02, 1374, 1302, 1291, 1256, 1205, 1156, 1145, 1120, 1068, 1058, 1033, 1011, 996, 957, 927, 888, 864, 845, 826, 809, 787, 767, 718, 694, 655, 613 cm^−1^. ^1^H-NMR (500 MHz, CDCl_3_): *δ* 0.93 (*s*, 9H), 1.49 (br *s*, 9H), 2.84 (*s*, 3H), 3.15 (*d*, *J* = 13.9 Hz, 1H), 3.73 (*s*, 6H), 3.75 (*d*, *J* = 11.1 Hz, 1H), 4.29 (*s*, 1H), 4.65 (*s*, 1H), 6.29 (*t*, *J* = 2.2 Hz, 1H), 6.33 (br *s*, 2H). ^13^C-NMR (126 MHz, CDCl_3_): *δ* 26.70, 28.39, 32.02, 33.91, 41.06, 55.34, 60.64, 81.09, 81.13, 99.08, 108.17, 138.14, 152.96, 160.38, 171.55.

*Synthesis of* tert*-butyl (2*S*,5*S*)-5-([1,1′-biphenyl]-3-ylmethyl)-2-(*tert*-butyl)-3-methyl-4-oxoimidazolidine-1-carboxylate (***3d***)*

Following *GP1*: Prepared from LDA (18 mmol, 9 mL), *tert*-butyl (*S*)-2-(*tert*-butyl)-3-methyl-4-oxoimidazolidine-1-carboxylate (**1a**) (16.364 mmol, 4.195 g), THF (V_1_ = 15 mL), 3-(bromomethyl)-1,1′-biphenyl (**2d**) (18 mmol, ω = 0.97, 4.586 g), THF (V_2_ = 10 mL), NH_4_Cl (80 mL), EtOAc (400 mL), NaHSO_4_ (140 mL), NaHCO_3_ (180 mL), and NaCl (180 mL); isolation by column chromatography (1. petroleum ether/EtOAc = 5:1 then 2. petroleum ether/EtOAc = 1:1). Yield: 5.876 g (13.909 mmol, 85%) of white solid; m.p. = 82.5–85.8 °C. [*α*]_D_^r.t.^ = +66.01 (c = 1.34 mg/mL, CH_2_Cl_2_). EI-HRMS: *m/z* = 423.2639 (MH^+^); C_26_H_35_N_2_O_3_ requires *m/z* = 423.2642 (MH^+^). CHN microanalysis: C_26_H_34_N_2_O_3_ requires C, 73.90; H, 8.11; N, 6.63. Found: C, 74.10; H, 8.39; N, 6.63. *ν*_max_ 2966, 1692, 1599, 1575, 1478, 1457, 1440, 13,990, 1378, 1363, 1339, 1304, 1257, 1164, 114, 1028, 1008, 996, 980, 946, 904, 881, 864, 827, 814, 783, 767, 753, 716, 703, 652, 615 cm^−1^. ^1^H-NMR (500 MHz, CDCl_3_): *δ* 0.93 (*s*, 9H), 1.42 (br *s*, 9H), 2.76 (br *s*, 3H), 3.26 (*dd*, *J* = 2.1, 14.1 Hz, 1H), 3.89 (br *s*, 1H), 4.36 (*s*, 1H), 4.60 (*s*, 1H), 7.14 (br *d*, *J* = 7.3 Hz, 1H), 7.24–7.35 (*m*, 2H), 7.38–7.44 (*m*, 4H), 7.54–7.60 (*m*, 2H). ^13^C-NMR (126 MHz, CDCl_3_): *δ* 26.72, 28.37, 31.96, 33.61, 41.09, 60.87, 81.15, 81.19, 125.51, 127.23, 127.26, 128.40, 128.77, 128.96, 129.18, 136.45, 140.89, 141.22, 152.82, 171.54.

*Synthesis of* tert*-butyl (2*S*,5*S*)-2-(*tert*-butyl)-3-methyl-5-((2-methylnaphthalen-1-yl)methyl)-4-oxoimidazolidine-1-carboxylate (***3e***)*

Following *GP1*: Prepared from LDA (11 mmol, 5.5 mL), *tert*-butyl (*S*)-2-(*tert*-butyl)-3-methyl-4-oxoimidazolidine-1-carboxylate (**1a**) (10 mmol, 2.564 g), THF (V_1_ = 15 mL), 1-(chloromethyl)-2-methylnaphthalene (**2e**) (11 mmol, ω = 0.97, 2.162 g), THF (V_2_ = 20 mL), NH_4_Cl (50 mL), EtOAc (250 mL), NaHSO_4_ (50 mL), NaHCO_3_ (70 mL), and NaCl (60 mL); isolation by column chromatography (1. petroleum ether/EtOAc = 5:1 then 2. petroleum ether/EtOAc = 3:1). Yield: 1.930 g (4.700 mmol, 47%) of white solid; m.p. = 163–166 °C. [*α*]_D_^r.t.^ = –195.2 (c = 2.9 mg/mL, CH_2_Cl_2_). EI-HRMS: *m/z* = 411.2646 (MH^+^); C_25_H_35_N_2_O_3_ requires *m/z* = 411.26422 (MH^+^). *ν*_max_ 2964, 2917, 1717, 1700, 1599, 1512, 1477, 1454, 1430, 1363, 1330, 1293, 1253, 1157, 1111, 1033, 1011, 968, 916, 871, 854, 814, 779, 750, 697, 659, 632 cm^−1^. ^1^H-NMR (500 MHz, CDCl_3_): *δ* 0.96 (*s*, 9H), 1.63 (*s*, 9H), 2.46 (*s*, 3H), 2.96 (*s*, 3H), 3.03 (*dd*, *J* = 10.7, 13.7 Hz, 1H), 4.43 (*dd*, *J* = 3.5, 10.6 Hz, 1H), 4.52 (br *d*, *J* = 11.8 Hz, 1H), 5.16 (br *s*, 1H), 7.32 (*d*, *J* = 8.4 Hz, 1H), 7.40 (*t*, *J* = 7.4 Hz, 1H), 7.47–7.56 (*m*, 1H), 7.67 (*d*, *J* = 8.3 Hz, 1H), 7.81 (*d*, *J* = 8.0 Hz, 1H), 8.26 (*d*, *J* = 7.8 Hz, 1H). ^13^C-NMR (126 MHz, CDCl_3_): *δ* 21.17, 26.48, 28.57, 30.86, 31.85, 41.09, 57.59, 80.50, 81.72, 124.01, 124.39, 125.93, 126.94, 128.87, 129.27, 130.00, 132.56, 135.78, 153.72, 170.98 (1 signal missing).

*Synthesis of* tert*-butyl (2*S*,5*S*)-5-(anthracen-9-ylmethyl)-2-(*tert*-butyl)-3-methyl-4-oxoimidazolidine-1-carboxylate (***3f***)*

Following *GP1*: Prepared from LDA (9 mmol, 4.5 mL), *tert*-butyl (*S*)-2-(*tert*-butyl)-3-methyl-4-oxoimidazolidine-1-carboxylate (**1a**) (8.182 mmol, 2.097 g), THF (V_1_ = 10 mL), 9-(chloromethyl)anthracene (**2f**) (9 mmol, ω = 0.95, 2.148 g), THF (V_2_ = 20 mL), NH_4_Cl (50 mL), EtOAc (200 mL), NaHSO_4_ (50 mL), NaHCO_3_ (80 mL), and NaCl (80 mL); isolation by column chromatography (1. petroleum ether/EtOAc = 5:1 then 2. petroleum ether/EtOAc = 1:2). Yield: 950 mg (2.127 mmol, 26%) of white solid; m.p. = 169–175 °C. EI-HRMS: *m/z* = 447.26417 (MH^+^); C_28_H_35_N_2_O_3_ requires *m/z* = 447.26422 (MH^+^). *ν*_max_ 2966, 1707, 1678, 1624, 1525, 1478, 1450, 1432, 1401, 1374, 1362, 1302, 1249, 1163, 1138, 1118, 1031, 968, 917, 891, 856, 778, 760, 734, 659, 634 cm^−1^. ^1^H-NMR (500 MHz, CDCl_3_): *δ* 0.97 (*s*, 9H), 1.65 (*s*, 9H), 2.94 (*s*, 3H), 3.69 (*dd*, *J* = 10.5, 14.1 Hz, 1H), 4.61 (*dd*, *J* = 4.1, 10.3 Hz, 1H), 4.95 (*d*, *J* = 12.0 Hz, 1H), 5.22 (*s*, 1H), 7.42–7.59 (*m*, 4H), 8.01 (*d*, *J* = 8.1 Hz, 2H), 8.33 (*d*, *J* = 8.4 Hz, 2H), 8.39 (*s*, 1H). ^13^C-NMR (126 MHz, CDCl_3_): *δ* 26.50, 28.57, 30.00, 31.90, 41.10, 58.41, 80.63, 81.80, 124.72, 124.97, 124.99, 125.47, 126.86, 129.33, 130.97, 131.54, 153.72, 170.73.

*Synthesis of (2*S*,5*S*)-1-benzoyl-5-(3,5-bis(trifluoromethyl)benzyl)-2-(*tert*-butyl)-3-methylimidazolidin-4-one (***3g***)*

Following *GP1*: Prepared from LDA (15.6 mmol, 7.8 mL), (*S*)-1-benzoyl-2-(*tert*-butyl)-3-methylimidazolidin-4-one (**1b**) (14.18 mmol, 3.692 g), THF (V_1_ = 40 mL), 1-(bromomethyl)-3,5-bis(trifluoromethyl)benzene (**2a**) (15.6 mmol, ω = 0.97, 4.938 g (2.948 mL)), THF (V_2_ = 5 mL), NH_4_Cl (40 mL), EtOAc (250 mL), NaHSO_4_ (60 mL), NaHCO_3_ (100 mL), and NaCl (100 mL); isolation by crystallization of the crude product (after extraction) from a mixture of *n*-hexane and ethyl acetate. Yield: 4.760 g (9.784 mmol, 69%) of white solid; m.p. = 163.7–166.8 °C. [*α*]_D_^r.t.^ = +69.76 (c = 3.8 mg/mL, CH_2_Cl_2_). EI-HRMS: *m/z* = 487.1815 (MH^+^); C_24_H_25_F_6_N_2_O_2_ requires: *m/z* = 487.1815 (MH^+^). CHN microanalysis: C_24_H_25_F_6_N_2_O_2_ requires C, 59.26; H, 4.97; N, 5.76. Found: C, 59.22; H, 4.81; N, 5.75. *ν*_max_ 2970, 2933, 1693, 1642, 1579, 1479, 1449, 1400, 1372, 1336, 1279, 1261, 1166, 1027, 969, 924, 906, 892, 878, 843, 804, 769, 756, 707, 695, 683, 657, 634 cm^−1^. ^1^H-NMR (500 MHz, DMSO-*d*_6_): *δ* 0.91 (br *s*, 9H), 2.60 (br *s*, 1H), 2.89 (s, 3H), 3.12 (br *s*, 1H), 5.05 (br *s*, 1H), 5.34 (br *s*, 1H), 6.77–7.90 (*m*, 7H), 7.96 (*s*, 1H). ^13^C-NMR (126 MHz, DMSO-*d*_6_): *δ* 25.97, 31.20, 35.93, 40.56, 60.23, 79.38, 120.64–120.90 (*m*), 123.21 (*q*, *J* = 272.7 Hz), 128.26, 128.91, 129.88, 129.93 (*q*, *J* = 32.7 Hz), 132.14, 135.65, 138.73, 169.22, 170.30.

*Hydrolysis of imidazolidinones* **3** *to amino amides* **4***—General Procedure 2 (GP2)*

To a solution of imidazolidinones **3** in methanol, HCl (aq., 1 M, V_1_) was added, and the resulting mixture was heated under reflux for 20 h. Methanol was evaporated in vacuo using a water pump, then HCl (aq., 2 M, V_2_) was added to the residue, and the mixture was extracted with CHCl_3_ (V_3_). The organic phase was discarded, and the aqueous phase was basified with NaOH (aq., 2 M) to pH 10–11. The mixture was extracted with CHCl_3_ (V_3_), dried over anhydrous Na_2_SO_4_, filtered, and the volatiles were evaporated in vacuo to obtain amino amides **4**.

*Synthesis of (*S*)-2-amino-3-(3,5-bis(trifluoromethyl)phenyl)-*N*-methylpropanamide (***4a***)*

Following *GP2*: Prepared from *tert*-butyl (2*S*,5*S*)-5-(3,5-bis(trifluoromethyl)benzyl)-2-(*tert*-butyl)-3-methyl-4-oxoimidazolidine-1-carboxylate (**3a**) (18.86 mmol, 9.100 g), MeOH (120 mL), HCl (V_1_ = 110 mL), HCl (V_2_ = 20 mL), CHCl_3_ (V_3_ = 2 × 60 mL), and NaOH (until pH = 10–11 CHCl_3_ (V_4_ = 5 × 60 mL). Yield: 5.512 g (17.540 mmol, 93%) of white solid; m.p. = 98–100 °C. [*α*]_D_^r.t.^ = –46.5 (c = 1.7 mg/mL, CH_2_Cl_2_). EI-HRMS: *m/z* = 315.0928 (MH^+^); C_12_H_13_F_6_N_2_O requires *m/z* = 315.0927 (MH^+^). CHN microanalysis: C_12_H_12_F_6_N_2_O requires C, 45.87; H, 3.85; N, 8.92. Found: C, 45.90; H, 3.65; N, 8.92. *ν*_max_ 2960, 1641, 1532, 1467, 1406, 1386, 1356, 1338, 1296, 1162, 1125, 942, 906, 843, 762, 708 cm^−1^. ^1^H-NMR (500 MHz, CDCl_3_): *δ* 1.28 (br *s*, 2H), 2.82 (*d*, *J* = 5.0 Hz, 3H), 2.97 (*dd*, *J* = 8.4, 14.0 Hz, 1H), 3.35 (*dd*, *J* = 4.2, 14.0 Hz, 1H), 3.68 (*dd*, *J* = 4.2, 8.4 Hz, 1H), 7.17 (br *s*, 1H), 7.68 (*s*, 2H), 7.78 (*s*, 1H). ^13^C-NMR (126 MHz, CDCl_3_): *δ* 26.00, 40.89, 56.16, 121.04 (*p*, *J* = 3.8 Hz), 123.38 (*q*, *J* = 272.8 Hz), 129.62 (*d*, *J* = 3.5 Hz), 132.00 (*q*, *J* = 33.3 Hz), 140.67, 173.78.

*Synthesis of (*S*)-2-amino-3-(2,5-bis(trifluoromethyl)phenyl)-*N*-methylpropanamide (***4b***)*

Following *GP2*: Prepared from *tert*-butyl (2*S*,5*S*)-5-(2,5-bis(trifluoromethyl)benzyl)-2-(*tert*-butyl)-3-methyl-4-oxoimidazolidine-1-carboxylate (**3b**) (5.259 mmol, 2.537 g), MeOH (40 mL), HCl (V_1_ = 35 mL), HCl (V_2_ = 10 mL), CHCl_3_ (V_3_ = 2 × 40 mL), and NaOH (until pH = 10–11 CHCl_3_ (V_4_ = 5 × 30 mL). Yield: 1.537 g (4.891 mmol, 93%) of white solid; m.p. = 78–81 °C. [*α*]_D_^r.t.^ = –21.9 (c = 0.94 mg/mL, CH_2_Cl_2_). EI-HRMS: *m/z* = 315.0927 (MH^+^); C_12_H_13_F_6_N_2_O requires *m/z* = 315.0927 (MH^+^). CHN microanalysis: C_12_H_12_F_6_N_2_O requires C, 45.87; H, 3.85; N, 8.92. Found: C, 45.83; H, 3.71; N, 8.86. *ν*_max_ 3386, 3317, 2971, 1642, 1553, 1510, 1417, 1334, 1307, 1264, 1236, 1184, 1167, 1118, 1085, 1037, 965, 951, 928, 911, 881, 837, 750, 694, 670, 645 cm^−1^. ^1^H-NMR (500 MHz, CDCl_3_): *δ* 1.34 (br *s*, 2H), 2.85 (*d*, *J* = 5.0 Hz, 3H), 3.01 (*ddd*, *J* = 1.3, 9.2, 14.7 Hz, 1H), 3.54 (*dd*, *J* = 4.5, 14.7 Hz, 1H), 3.67 (*dd*, *J* = 4.5, 9.2 Hz, 1H), 7.21 (br *s*, 1H), 7.62 (*d*, *J* = 8.3 Hz, 1H), 7.72 (*s*, 1H), 7.81 (*d*, *J* = 8.2 Hz, 1H). ^13^C-NMR (126 MHz, CDCl_3_): *δ* 26.04, 37.38 (*d*, *J* = 1.8 Hz), 56.40, 123.36 (*q*, *J* = 273.0 Hz), 123.81 (*q*, *J* = 274.3 Hz), 123.86 (*q*, *J* = 3.6 Hz), 127.08 (*q*, *J* = 5.8 Hz), 128.43 (*q*, *J* = 3.6 Hz), 132.79 (*q*, *J* = 29.7 Hz), 134.15 (*q*, *J* = 33.0 Hz), 138.84 (*d*, *J* = 1.7 Hz), 174.13.

*Synthesis of (*S*)-2-amino-3-(3,5-dimethoxyphenyl)-*N*-methylpropanamide (***4c***)* [33]

Following *GP2*: Prepared from *tert*-butyl (2*S*,5*S*)-2-(*tert*-butyl)-5-(3,5-dimethoxybenzyl)-3-methyl-4-oxoimidazolidine-1-carboxylate (**3c**) (9.781 mmol, 3.976 g), MeOH (80 mL), HCl (V_1_ = 65 mL), HCl (V_2_ = 20 mL), CHCl_3_ (V_3_ = 2 × 50 mL), and NaOH (until pH = 10–11 CHCl_3_ (V_4_ = 5 × 50 mL). Yield: 2.121 g (8.901 mmol, 91%) of white solid; m.p. = 59.8–62 °C. [*α*]_D_^r.t.^ = –63.9 (c = 2.9 mg/mL, CH_2_Cl_2_). EI-HRMS: *m/z* = 239.1385 (MH^+^); C_12_H_19_N_2_O_3_ requires *m/z* = 239.1390 (MH^+^). CHN microanalysis: C_12_H_18_N_2_O_3_ requires C, 60.49; H, 7.61; N, 11.76. Found: C, 60.62; H, 7.73; N, 11.42. *ν*_max_ 3379, 3315, 2957, 2863, 2836, 1735, 1637, 1594, 1524, 1462, 1444, 1427, 1400, 1346, 1332, 1290, 1204, 1146, 1096, 1081, 1056, 993, 955, 906, 876, 838, 822, 786, 743, 690, 656, 610 cm^−1^. ^1^H-NMR (500 MHz, CDCl_3_): *δ* 1.46 (br *s*, 2H), 2.60 (*dd*, *J* = 9.7, 13.6 Hz, 1H), 2.83 (*d*, *J* = 5.0 Hz, 3H), 3.24 (*dd*, *J* = 3.9, 13.6 Hz, 1H), 3.60 (*dd*, *J* = 3.9, 9.6 Hz, 1H), 3.78 (*s*, 6H), 6.31–6.40 (*m*, 3H), 7.33 (br *s*, 1H). ^13^C-NMR (126 MHz, CDCl_3_): *δ* 25.97, 41.46, 55.44, 56.49, 98.88, 107.27, 140.52, 161.13, 174.91.

*Synthesis of (*S*)-3-([1,1′-biphenyl]-3-yl)-2-amino-*N*-methylpropanamide (***4d***)*

Following *GP2*: Prepared from *tert*-butyl (2*S*,5*S*)-5-([1,1′-biphenyl]-3-ylmethyl)-2-(*tert*-butyl)-3-methyl-4-oxoimidazolidine-1-carboxylate (**3d**) (13.851 mmol, 5.853 g), MeOH (115 mL), HCl (V_1_ = 100 mL), HCl (V_2_ = 30 mL), CHCl_3_ (V_3_ = 2 × 50 mL), and NaOH (until pH = 10–11 CHCl_3_ (V_4_ = 5 × 60 mL). Yield: 3.311 g (13.020 mmol, 94%) of white solid; m.p. = 68–71 °C. [*α*]_D_^r.t.^ = –59.7 (c = 0.94 mg/mL, CH_2_Cl_2_). EI-HRMS: *m/z* = 255.1489 (MH^+^); C_16_H_19_N_2_O requires *m/z* = 255.1488 (MH^+^). CHN microanalysis: C_16_H_18_N_2_O requires C, 75.56; H, 7.13; N, 11.01. Found: C, 75.37; H, 7.16; N, 10.98. *ν*_max_ 3377, 3349, 2884, 1637, 1523, 1477, 1449, 1419, 1403, 1258, 1217, 1156, 1109, 1074, 1026, 1000, 990, 902, 885, 848, 797, 775, 757, 720, 698, 649, 618 cm^−1^. ^1^H-NMR (500 MHz, CDCl_3_): *δ* 1.31 (br *s*, 2H), 2.73 (*dd*, *J* = 9.5, 13.8 Hz, 1H), 2.83 (*d*, *J* = 5.0 Hz, 3H), 3.36 (*dd*, *J* = 3.9, 13.8 Hz, 1H), 3.65 (*dd*, *J* = 3.9, 9.5 Hz, 1H), 7.17–7.23 (*m*, 1H), 7.30 (br *s*, 1H), 7.33–7.51 (*m*, 6H), 7.55–7.61 (*m*, 2H). ^13^C-NMR (126 MHz, CDCl_3_): *δ* 25.96, 41.25, 56.64, 125.74, 127.23, 127.54, 128.17, 128.27, 128.92, 129.24, 138.68, 140.93, 141.75, 174.91.

*Synthesis of (*S*)-2-amino-*N*-methyl-3-(2-methylnaphthalen-1-yl)propanamide (***4e***)*

Following *GP2*: Prepared from *tert*-butyl (2*S*,5*S*)-2-(*tert*-butyl)-3-methyl-5-((2-methylnaphthalen-1-yl)methyl)-4-oxoimidazolidine-1-carboxylate (**3e**) (3.605 mmol, 1.480 g), MeOH (30 mL), HCl (V_1_ = 25 mL), HCl (V_2_ = 10 mL), CHCl_3_ (V_3_ = 2 × 30 mL), and NaOH (until pH = 10–11 CHCl_3_ (V_4_ = 5 × 30 mL). Yield: 856 mg (3.533 mmol, 98%) of white solid; m.p. = 93–96 °C. [*α*]_D_^r.t.^ = –11.1 (c = 0.96 mg/mL, CH_2_Cl_2_). EI-HRMS: *m/z* = 243.1492 (MH^+^); C_15_H_19_N_2_O requires *m/z* = 243.1492 (MH^+^). CHN microanalysis: C_15_H_18_N_2_O requires C, 74.35; H, 7.49; N, 11.56. Found: C, 73.99; H, 7.77; N, 11.52. *ν*_max_ 3376, 3329, 3033, 2944, 1900, 1648, 1528, 1405, 1280, 1259, 1218, 1153, 1101, 1033, 977, 958, 918, 875, 860, 839, 805, 778, 737, 680 cm^−1^. ^1^H-NMR (500 MHz, CDCl_3_): *δ* 1.27 (br *s*, 2H), 2.56 (*s*, 3H), 2.83 (*d*, *J* = 5.0 Hz, 3H), 3.20 (*td*, *J* = 3.3, 12.0 Hz, 1H), 3.75–3.84 (*m*, 2H), 7.13 (br *s*, 1H), 7.31 (*d*, *J* = 8.4 Hz, 1H), 7.39–7.46 (*m*, 1H), 7.48–7.55 (*m*, 1H), 7.66 (*d*, *J* = 8.4 Hz, 1H), 7.80 (*d*, *J* = 7.8 Hz, 1H), 8.19 (*d*, *J* = 8.6 Hz, 1H). ^13^C-NMR (126 MHz, CDCl_3_): *δ* 20.88, 25.96, 33.70, 56.31, 123.85, 124.96, 126.47, 127.19, 128.79, 129.34, 131.50, 132.60, 132.73, 134.67, 175.38.

*Synthesis of (*S*)-2-amino-3-(anthracen-9-yl)-*N*-methylpropanamide (***4f***)*

Following *GP2*: Prepared from *tert*-butyl (2*S*,5*S*)-5-(anthracen-9-ylmethyl)-2-(*tert*-butyl)-3-methyl-4-oxoimidazolidine-1-carboxylate (**3f**) (2.100 mmol, 938 mg), MeOH (40 mL), HCl (V_1_ = 15 mL), HCl (V_2_ = 10 mL), CHCl_3_ (V_3_ = 2 × 30 mL), and NaOH (until pH = 10–11 CHCl_3_ (V_4_ = 5 × 30 mL). Yield: 421 mg (1.512 mmol, 72%) of white solid; m.p. = 98–102 °C. [*α*]_D_^r.t.^ = +9.92 (c = 1.1 mg/mL, CH_2_Cl_2_). EI-HRMS: *m/z* = 279.1492 (MH^+^); C_18_H_19_N_2_O requires *m/z* = 279.1491 (MH^+^). *ν*_max_ 3295, 3050, 2938, 2184, 2162, 2085, 2009, 1940, 1650, 1523, 1445, 1407, 1348, 1316, 1284, 1258, 1227, 1157, 1103, 1020, 995, 955, 931, 884, 840, 787, 731, 698, 648 cm^−1^. ^1^H-NMR (500 MHz, CDCl_3_): *δ* 1.24 (br *s*, 2H), 2.67 (*d*, *J* = 5.0 Hz, 3H), 3.57 (*dd*, *J* = 10.4, 14.6 Hz, 1H), 3.73 (*dd*, *J* = 3.5, 10.3 Hz, 1H), 4.16 (*dd*, *J* = 3.9, 14.6 Hz, 1H), 7.06 (br *s*, 1H), 7.27–7.35 (*m*, 2H), 7.36–7.42 (*m*, 2H), 7.85 (*d*, *J* = 8.4 Hz, 2H), 8.21–8.27 (*m*, 3H). ^13^C-NMR (126 MHz, CDCl_3_): *δ* 26.03, 32.70, 57.34, 124.46, 125.18, 126.30, 126.97, 129.43, 130.55, 130.65, 131.66, 175.29.

*Synthesis of (*S*)-2-amino-3-(4-methoxyphenyl)-*N*-methylpropanamide (***4h***)* [37]

Crude methyl (*S*)-2-((*tert*-butoxycarbonyl)amino)-3-(4-methoxyphenyl)propanoate (**B**), prepared from (*tert*-butoxycarbonyl)-L-tyrosine (**A**) (28.4 mmol, 8.00 g) following the literature procedure [38], was dissolved in CH_3_CO_2_H (TFA, 5 mL) and stirred at room temperature for 12 h. Volatile components were evaporated in vacuo, and the residue was suspended in CH_2_Cl_2_ (200 mL) and washed with saturated aqueous NaHCO_3_ (50 mL). The organic phase was dried over anhydrous Na_2_SO_4_, filtered, and volatile components were evaporated in vacuo to yield crude methyl (*S*)-2-amino-3-(4-methoxyphenyl)propanoate (**5a**). Crude **5a** was dissolved in a solution of MeNH_2_ (8 M in EtOH, 30 mL) and stirred at room temperature for 12 h. Volatile components were evaporated in vacuo to give crude **4h**, ready for cyclization with fluoroacetone. Yield: 4.377 g (21.016 mmol, 74%; **A**→**4h**) of white solid. ^1^H-NMR (300 MHz, CDCl_3_): *δ* 1.31 (br *s*, NH_2_), 2.65 (*dd*, *J* = 9.2; 13.8 Hz, 1H of CH_2_), 2.81 (*d*, *J* = 5.0 Hz, NH*Me*), 3.19 (*dd*, *J* = 4.1; 13.8 Hz, 1H of CH_2_), 3.56 (*dd*, *J* = 4.0; 9.2 Hz, CH), 3.79 (*s*, OMe), 6.82–6.88 (*m*, 2 arom. H), 7.10–7.16 (*m*, 2 arom. H), 7.21 (br s, *NH*Me).

*Synthesis of (*S*)-2-amino-*N*-methyl-3-(4-nitrophenyl)propanamide (***4i***)* [39]

(*S*)-(+)-4-Nitrophenylalanine methyl ester hydrochloride (**5b**) (20.8 mmol, 5.422 g) was suspended in CH_2_Cl_2_ (200 mL) and washed with saturated aqueous NaHCO_3_ (50 mL). The organic phase was dried over anhydrous Na_2_SO_4_, filtered, and the volatile components were evaporated in vacuo. The residue was dissolved in a solution of MeNH_2_ (8 M in EtOH, 30 mL) and stirred at room temperature for 12 h. Volatile components were partially evaporated in vacuo (ca. 2/3), followed by the addition of Et_2_O (50 mL) to the residue. The precipitate was collected by filtration and washed with cold (0 °C) Et_2_O (50 mL) to give **4i**, ready for cyclization with fluoroacetone. Yield: 3.065 g (13.728 mmol, 66%) of yellowish solid. ^1^H-NMR (300 MHz, CDCl_3_): *δ* 1.28 (br *s*, NH_2_), 2.82 (*d*, *J* = 5.0 Hz, NH*Me*), 2.92 (*dd*, *J* = 8.6; 13.8 Hz, 1H of CH_2_), 3.35 (*dd*, *J* = 4.2; 13.8 Hz, 1H of CH_2_), 3.66 (*dd*, *J* = 4.2; 8.6 Hz, CH), 7.20 (br *s*, *NH*Me), 7.37–7.42 (*m*, 2 arom. H), 8.14–8.22 (*m*, 2 arom. H).

*Synthesis of α-amino amides* **2** *from α-amino esters* **1***—General Procedure 3 (GP3)*

α-Amino acid methyl ester hydrochloride **5** (30 mmol) was dissolved in an ethanolic solution of MeNH_2_ (33 wt.% in absolute ethanol, 40 mL), and the reaction mixture was stirred at room temperature for 48 h. Volatile components were evaporated in vacuo, and the residue was suspended in CH_2_Cl_2_ (150 mL) and washed with saturated aqueous NaHCO_3_ (2 × 20 mL) and saturated aqueous NaCl (2 × 20 mL). The organic phase was dried over anhydrous Na_2_SO_4_, filtered, and the volatiles were evaporated in vacuo.

*Synthesis of (*S*)-2-amino-*N*,3-dimethylbutanamide (***4j***)* [35]

Following *GP3*: Prepared from methyl (*S*)-2-amino-3-methylbutanoate hydrochloride (**5c**) (30.0 mmol, 5.029 g). Yield: 2.539 g (19.5 mmol, 65%) of colorless oil. ^1^H-NMR (300 MHz, CDCl_3_): *δ* 0.82 (*d*, *J* = 6.9 Hz, 3H), 0.98 (*d*, *J* = 7.0 Hz, 3H), 1.34 (br *s*, 2H), 2.24–2.38 (*m*, 1H), 2.82 (*dd*, *J* = 0.5, 5.0 Hz, 3H), 3.23 (*dd*, *J* = 0.6 Hz, 3.9, 1H), 7.28 (br *s*, 1H).

*Synthesis of (*S*)-2-amino-*N*,4-dimethylpentanamide (***4k***)* [40]

Following *GP3*: Prepared from (*S*)-2-Amino-4-methylpentanoic acid methyl ester hydrochloride (**5d**) (30.0 mmol, 5.450 g). Yield: 2.942 g (20.4 mmol, 68%) of colorless oil. ^1^H-NMR (300 MHz, CDCl_3_): *δ* 0.93 (*d*, *J* = 6.2 Hz, 3H), 0.96 (*d*, *J* = 6.3 Hz, 3H), 1.26–1.46 (*m*, 3H), 1.64–1.80 (*m*, 2H), 2.81 (*dd*, *J* = 0.4, 5.0 Hz, 3H), 3.38 (*dd*, *J* = 3.5, 9.8 Hz, 1H), 7.26 (br *s*, 1H).

*Synthesis of imidazolidinones* **6** *from amino amides* **4***—General Procedure 4 (GP4)*

To a solution of α-amino amide **4** (1 equiv.) in anhydrous ethanol under argon, propanal or acetaldehyde (1.15 equiv.) was added. The reaction mixture was heated at reflux under argon using a Dean–Stark apparatus filled with freshly dried molecular sieves (4 Å MS) for 20 h. The volatiles were evaporated in vacuo, and the residue was purified by column chromatography (Silica gel 60). The fractions containing the pure separated isomers ***trans*-6** and ***cis*-6** were combined, and the volatiles were evaporated in vacuo. The products were stored under argon at 5 °C; ***trans*-6** and ***cis*-6** mixed fractions were combined separately and then used in the subsequent *N*-Boc protection procedure (see *General Procedure 5 (GP5)*).

N*-Boc protection of imidazolidinones* **6***—General Procedure 5 (GP5)*

To a solution of imidazolidinone **6** (1 equiv.) in anhydrous CH_2_Cl_2_ under argon at room temperature, Boc_2_O (2.2 equiv.), Et_3_N (2.2 equiv.), and 4-dimethylaminopyridine (DMAP, 0.2 equiv.) were added. The reaction mixture was heated at reflux under argon for 16 h. The volatiles were evaporated in vacuo, and the residue was purified by flash column chromatography (Silica gel 60). The fractions containing the pure separated isomers ***trans*-7** and ***cis*-7** were combined, and the volatiles were evaporated in vacuo. If the two geometric isomers did not separate or separated with low yields, column chromatography was repeated.

N*-Boc deprotection of imidazolidinones* **7***—General Procedure 6 (GP6)*

To a solution of *N*-Boc protected imidazolidinone **7** (1 equiv.) in anhydrous CH_2_Cl_2_ under argon at 0 °C, an equal volume of trifluoroacetic acid was added. The reaction mixture was stirred at room temperature for t_1_ h. The volatiles were evaporated in vacuo, and the residue was dissolved in CH_2_Cl_2_ (50 mL) and then washed with saturated aqueous NaHCO_3_ (20 mL) and saturated aqueous NaCl (10 mL). The organic phase was dried over anhydrous Na_2_SO_4_, filtered, and the volatiles were evaporated in vacuo to give **6**.

*Synthesis of (2*R*,5*S*)-5-benzyl-2,3-dimethylimidazolidin-4-one (**trans*****-6a***) and (2*S*,5*S*)-5-benzyl-2,3-dimethylimidazolidin-4-one (**cis*****-6a***)*

Following *GP4*: Prepared from (*S*)-2-amino-*N*-methyl-3-phenylpropanamide (**4g**) (5.0 mmol, 891 mg), EtOH (30 mL), and ethanal (5.75 mmol, 323 μL); column chromatography: petroleum ether/EtOAc = 3:1 to elute ***trans*-6a** and ***cis*-6a** as a mixture. The two diastereomers could not be separated by column chromatography; ***trans*-6a**/***cis*-6a** = 63:37 (after column chromatography in CDCl_3_). Yield: 347 mg (1.70 mmol, 34%) of yellowish oil.

*Synthesis of* tert*-butyl (2*S*,5*S*)-5-benzyl-2,3-dimethyl-4-oxoimidazolidine-1-carboxylate (**trans*****-7a***) and* tert*-butyl (2*R*,5*S*)-5-benzyl-2,3-dimethyl-4-oxoimidazolidine-1-carboxylate (**cis*****-7a***)*

Following *GP5*: Prepared from (2*R*,5*S*)-5-benzyl-2,3-dimethylimidazolidin-4-one (***trans*-6a**) and (2*S*,5*S*)-5-benzyl-2,3-dimethylimidazolidin-4-one (***cis*-6a**) (***trans*-6a**/***cis*-6a** = 63:37, 1.69 mmol, 345 mg), CH_2_Cl_2_ (5 mL), Boc_2_O (3.718 mmol, 811 mg), Et_3_N (3.718 mmol, 518 μL), and DMAP (0.338 mmol, 41 mg); column chromatography: petroleum ether/EtOAc = 3:1.

***trans*-7a**: elutes first from the column. Yield: 247 mg (0.811 mmol, 48%) of yellowish oil. Two rotamers at a 54:46 ratio in CDCl_3_. ^1^H-NMR (500 MHz, CDCl_3_) for the major rotamer: *δ*: 1.32 (*d*, *J* = 5.4 Hz, 3H), 1.52 (*s*, 9H), 2.61 (*s*, 3H), 3.13–3.15 (*m*, 1H), 3.67 (*dd*, *J* = 5.4, 13.6 Hz, 1H), 4.01 (*qd*, *J* = 2.2, 5.4 Hz, 1H), 4.39–4.45 (*m*, 1H), 7.06–7.15 (*m*, 2H), 7.16–7.28 (*m*, 3H). ^1^H-NMR (500 MHz, CDCl_3_) for the minor rotamer: *δ* 1.39 (*d*, *J* = 5.4 Hz, 3H), 1.60 (*s*, 9H), 2.55 (*s*, 3H), 3.08–3.12 (*m*, 1H), 3.39 (*dd*, *J* = 4.9, 13.7 Hz, 1H), 4.06 (*qd*, *J* = 2.1, 5.4 Hz, 1H), 4.32–4.37 (*m*, 1H). ^13^C-NMR (126 MHz, CDCl_3_) for both rotamers: *δ* 18.69, 19.86, 26.22, 26.40, 28.57, 28.71, 34.10, 36.04, 59.75, 60.11, 70.68, 70.99, 80.94, 81.17, 126.86, 127.06, 128.07, 128.34, 129.87, 130.03, 135.68, 136.12, 152.21, 152.39, 168.84, 169.01.

***cis*-7a**: elutes second from the column. Yield: 144 mg (0.473 mmol, 28%) of yellowish oil. Two rotamers at a 56:44 ratio in CDCl_3_. ^1^H-NMR (500 MHz, CDCl_3_) for the major rotamer: *δ*: 0.50 (*d*, *J* = 5.6 Hz, 3H), 1.54 (*s*, 9H), 2.70 (*s*, 3H), 3.11–3.26 (*m*, 2H), 4.35 (*t*, *J* = 4.1 Hz, 1H), 4.84 (*q*, *J* = 5.7 Hz, 1H), 7.07–7.13 (*m*, 2H), 7.16–7.28 (*m*, 3H). ^1^H-NMR (500 MHz, CDCl_3_) for the minor rotamer: *δ* 0.38 (*d*, *J* = 5.6 Hz, 3H), 1.51 (*s*, 9H), 2.69 (*s*, 3H), 3.35 (*dd*, *J* = 5.9, 13.7 Hz, 1H), 4.44 (*d*, *J* = 5.8 Hz, 1H), 4.74 (*q*, *J* = 5.6 Hz, 1H). ^13^C-NMR (126 MHz, CDCl_3_) for both rotamers: *δ* 18.55, 18.76, 26.24, 28.42, 28.48, 35.43, 36.60, 60.59, 60.91, 70.45, 70.53, 80.83, 80.97, 126.75, 126.88, 128.14, 128.24, 130.31, 130.35, 136.54, 136.86, 152.71, 153.25, 168.87 (2 signals missing).

*Synthesis of (2*S*,5*S*)-5-benzyl-2,3-dimethylimidazolidin-4-one (**cis*****-6a***)*

Following *GP6*: Prepared from *tert*-butyl (2*R*,5*S*)-5-benzyl-2,3-dimethyl-4-oxoimidazolidine-1-carboxylate (***cis*-7a**) (0.131 mmol, 40 mg), CH_2_Cl_2_ (2 mL), and CF_3_CO_2_H (3 mL), 5 h. Yield: 20 mg (0.09825 mmol, 75%) of yellowish oil. [*α*]_D_^r.t.^ = [*α*]_D_^r.t.^ = –66.1 (c = 0.65 mg/mL, CH_2_Cl_2_). EI-HRMS: *m/z* = 205.1337 (MH^+^); C_12_H_17_N_2_O requires *m/z* = 205.1335 (MH^+^); *ν*_max_ 3324, 3056, 2972, 2929, 2857, 2828, 1672, 1604, 1484, 1455, 1437, 1416, 1402, 1331, 1269, 1227, 1192, 1120, 1103, 1039, 1015, 983, 898, 842, 807, 754, 698, 621, 603 cm^−1^. ^1^H-NMR (500 MHz, CDCl_3_): *δ* 1.15 (*d*, *J* = 5.7 Hz, 3H), 1.72 (br *s*, 1H), 2.77 (*s*, 3H), 2.98 (*dd*, *J* = 7.1, 14.3 Hz, 1H), 3.19 (*dd*, *J* = 4.5, 14.3 Hz, 1H), 3.71 (*dd*, *J* = 4.4, 7.1 Hz, 1H), 4.35 (*qd*, *J* = 1.1, 5.7 Hz, 1H), 7.17–7.36 (*m*, 5H). ^13^C-NMR (126 MHz, CDCl_3_): *δ* 20.55, 26.80, 37.42, 60.65, 70.63, 126.92, 128.75, 129.52, 137.26, 174.62.

*Synthesis of (2*R*,5*S*)-5-(3,5-bis(trifluoromethyl)benzyl)-2,3-dimethylimidazolidin-4-one (**trans*****-6c***) and (2*S*,5*S*)-5-(3,5-bis(trifluoromethyl)benzyl)-2,3-dimethylimidazolidin-4-one (**cis*****-6c***)*

Following *GP4*: Prepared from (*S*)-2-amino-3-(3,5-bis(trifluoromethyl)phenyl)-*N*-methylpropanamide (**4a**) (4.790 mmol, 1.505 g), EtOH (30 mL), and ethanal (11.4 mmol, 640 μL); column chromatography: EtOAc to elute ***trans*-6c** and ***cis*-6c** as a mixture. The two diastereomers could not be separated by column chromatography; ***trans*-6c**/***cis*-6c** = 64:36 (after column chromatography in CDCl_3_). Yield: 1.108 g (3.257 mmol, 68%) of yellowish oil. EI-HRMS: *m/z* = 341.1078 (MH^+^); C_14_H_15_F_6_N_2_O requires: *m/z* = 341.1083 (MH^+^).

*Synthesis of* tert*-butyl (2*R*,5*S*)-5-(3,5-bis(trifluoromethyl)benzyl)-2,3-dimethyl-4-oxoimidazolidine-1-carboxylate (**cis*****-7c***) and (2*R*,5*S*)-5-(3,5-bis(trifluoromethyl)benzyl)-2,3-dimethylimidazolidin-4-one (**trans*****-6c***)*

Following *GP5*: Prepared from (2*R*,5*S*)-5-(3,5-bis(trifluoromethyl)benzyl)-2,3-dimethylimidazolidin-4-one (***trans*-6c**) and (2*S*,5*S*)-5-(3,5-bis(trifluoromethyl)benzyl)-2,3-dimethylimidazolidin-4-one (***cis*-6c**) (***trans*-6c**/***cis*-6c** = 64:36, 3.230 mmol, 1.099 g), CH_2_Cl_2_ (7 mL), Boc_2_O (6.460 mmol, 1.410 g), Et_3_N (6.460 mmol, 900 μL), and DMAP (0.646 mmol, 79 mg); first column chromatography: petroleum ether/EtOAc = 1:1; ***trans*-6c** and ***cis*-7c** did not separate, but several other impurities were removed; second column chromatography: petroleum ether/EtOAc = 1:1, ***trans*-6c** and ***cis*-7c** separated.

***cis*-7c**: elutes first from the column. Yield: 669 mg (1.518 mmol, 47%) of yellowish oil. [*α*]_D_^r.t.^ = +67.9 (c = 1.40 mg/mL, CH_2_Cl_2_). EI-HRMS: *m/z* = 441.1601 (MH^+^); C_19_H_23_F_6_N_2_O_3_ requires *m/z* = 441.1607 (MH^+^); *ν*_max_ 2979, 1700, 1445, 1411, 1378, 1343, 1315, 1275, 1168, 1125, 1060, 1030, 963, 947, 902, 889, 844, 774, 724, 708, 682, 659 cm^−1^. Two rotamers at a 58:42 ratio in CDCl_3_. ^1^H-NMR (500 MHz, CDCl_3_) for the major rotamer: *δ*: *δ* 0.41 (br *s*, 3H), 1.51 (br *s*, 9H), 2.69 (br *s*, 3H), 3.28–3.42 (*m*, 2H), 4.47 (br *s*, 1H), 4.77 (br *s*, 1H), 7.58 (*s*, 2H), 7.75 (*s*, 1H). ^1^H-NMR (500 MHz, CDCl_3_) for the minor rotamer: *δ* 0.55 (br *s*, 3H), 1.54 (br *s*, 9H), 3.50–3.58 (*m*, 1H), 4.39 (br *s*, 1H), 4.87 (br *s*, 1H). ^13^C-NMR (126 MHz, CDCl_3_) for both rotamers: *δ* 19.10, 19.33, 26.29, 28.39, 35.42, 36.72, 60.22, 60.42, 70.35, 70.49, 81.78, 120.80, 123.39 (*q*, *J* = 272.4 Hz), 130.63, 131.62 (*q*, *J* = 32.5 Hz), 139.42, 139.70, 153.00, 153.18, 168.08.

***trans*-6c**: elutes second from the column. Yield: 198 mg (0.581 mmol, 18%) of yellowish oil. [*α*]_D_^r.t.^ = –31.8 (c = 1.15 mg/mL, CH_2_Cl_2_). EI-HRMS: *m/z* = 341.1077 (MH^+^); C_14_H_15_F_6_N_2_O requires *m/z* = 341.1083 (MH^+^); *ν*_max_ 2934, 1687, 1433, 1404, 1379, 1274, 1167, 1120, 899, 841, 790, 725, 706, 682, 643 cm^−1^. ^1^H-NMR (500 MHz, CDCl_3_): *δ* 1.29 (*d*, *J* = 5.7 Hz, 3H), 1.83 (br *s*, 1H), 2.72 (*s*, 3H), 2.98 (*dd*, *J* = 7.4, 13.8 Hz, 1H), 3.13 (*dd*, *J* = 4.0, 13.8 Hz, 1H), 3.90 (*dd*, *J* = 4.0, 7.1 Hz, 1H), 4.02 (*qd*, *J* = 1.4, 5.7 Hz, 1H), 7.76 (*s*, 3H). ^13^C-NMR (126 MHz, CDCl_3_): *δ* 20.52, 26.61, 38.14, 59.89, 71.28, 120.70 (*p*, *J* = 3.9 Hz), 123.48 (*q*, *J* = 272.6 Hz), 130.05 (*d*, *J* = 3.8 Hz), 131.50 (*q*, *J* = 33.1 Hz), 140.79, 173.54.

*Synthesis of (2*S*,5*S*)-5-(3,5-bis(trifluoromethyl)benzyl)-2,3-dimethylimidazolidin-4-one (**cis*****-6c***)*

Following *GP6*: Prepared from *tert*-butyl (2*R*,5*S*)-5-(3,5-bis(trifluoromethyl)benzyl)-2,3-dimethyl-4-oxoimidazolidine-1-carboxylate (***cis*-7c**) (1.258 mmol, 554 mg), CH_2_Cl_2_ (5 mL), and CF_3_CO_2_H (5 mL), 2 h. Yield: 227 mg (0.667 mmol, 53%) of yellowish oil. [*α*]_D_^r.t.^ = –31.0 (c = 1.3 mg/mL, CH_2_Cl_2_). EI-HRMS: *m/z* = 341.1803 (MH^+^); C_14_H_15_F_6_N_2_O requires *m/z* = 341.1083 (MH^+^); *ν*_max_ 3326, 2978, 2932, 2867, 1687, 1379, 1274, 1167, 1120, 898, 842, 706, 682 cm^−1^. ^1^H-NMR (500 MHz, CDCl_3_): *δ* 1.18 (*d*, *J* = 5.7 Hz, 3H), 1.81 (br *s*, 1H), 2.79 (*s*, 3H), 2.97 (*dd*, *J* = 7.9, 14.3 Hz, 1H), 3.32 (*dd*, *J* = 3.9, 14.3 Hz, 1H), 3.81 (*dd*, *J* = 3.9, 7.9 Hz, 1H), 4.47 (*qd*, *J* = 1.2, 5.8 Hz, 1H), 7.75 (*s*, 3H). ^13^C-NMR (126 MHz, CDCl_3_): *δ* 21.42, 26.74, 38.42, 60.09, 70.42, 120.75 (*dt*, *J* = 4.0, 8.1 Hz), 123.46 (*q*, *J* = 272.7 Hz), 129.89 (*d*, *J* = 3.7 Hz), 131.62 (*q*, *J* = 33.2 Hz), 140.63, 173.04.

*Synthesis of (2*R*,5*S*)-5-(3,5-bis(trifluoromethyl)benzyl)-2-ethyl-3-methylimidazolidin-4-one (**trans*****-6d***) and (2*S*,5*S*)-5-(3,5-bis(trifluoromethyl)benzyl)-2-ethyl-3-methylimidazolidin-4-one (**cis*****-6d***)*

Following *GP4*: Prepared from (*S*)-2-amino-3-(3,5-bis(trifluoromethyl)phenyl)-*N*-methylpropanamide (**4a**) (4.716 mmol, 1.482 g), EtOH (30 mL), and propanal (5.423 mmol, ω = 0.97, 403 μL); column chromatography: 1. EtOAc/toluene/Et_3_N = 25:5:1 to elute ***trans*-6d**; 2. EtOAc/toluene/Et_3_N = 25:5:1 to elute ***cis*-6d**. ***trans*-6d**/***cis*-6d** = 43:57 (crude reaction mixture in CDCl_3_).

***trans*-6d**: elutes first from the column. Yield: 234 mg (0.660 mmol, 14%) of yellowish oil. [*α*]_D_^r.t.^ = –39.06 (c = 1.55 mg/mL, CH_2_Cl_2_). EI-HRMS: *m/z* = 355.1238 (MH^+^); C_15_H_17_F_6_N_2_O requires *m/z* = 355.1240 (MH^+^); *ν*_max_ 3325, 2969, 2932, 1686, 1622, 1464, 1434, 1406, 1379, 1343, 1275, 1167, 1124, 990, 941, 924, 897, 841, 769, 724, 706, 682, 650 cm^−1^. ^1^H-NMR (500 MHz, CDCl_3_): *δ* 0.86 (*t*, *J* = 7.5 Hz, 3H), 1.49 (*dp*, *J* = 7.3, 14.3 Hz, 1H), 1.67–1.78 (*m*, 1H), 1.83 (br *s*, 1H), 2.70 (*s*, 3H), 2.97 (*dd*, *J* = 7.2, 13.8 Hz, 1H), 3.14 (*dd*, *J* = 4.1, 13.8 Hz, 1H), 3.89 (*dd*, *J* = 4.4, 6.8 Hz, 1H), 3.92–3.95 (*m*, 1H), 7.74 (*s*, 3H). ^13^C-NMR (126 MHz, CDCl_3_): *δ* 7.14, 26.29, 26.82, 38.44, 59.84, 75.58, 120.69 (*dt*, *J* = 4.0, 8.0 Hz), 123.49 (*q*, *J* = 272.6 Hz), 130.11 (*q*, *J* = 3.9 Hz), 131.51 (*q*, *J* = 33.1 Hz), 140.71, 173.64.

***cis*-6d**: elutes second from the column. Yield: 167 mg (0.472 mmol, 10%) of yellowish oil. [*α*]_D_^r.t.^ = –12.2 (c = 1.15 mg/mL, CH_2_Cl_2_). EI-HRMS: *m/z* = 355.1240 (MH^+^); C_15_H_17_F_6_N_2_O requires *m/z* = 355.1240 (MH^+^); *ν*_max_ 3327, 2955, 2925, 1678, 1484, 1466, 1441, 1412, 1377, 1347, 1311, 1274, 1163, 1122, 1076, 1058, 996, 962, 942, 917, 897, 882, 847, 833, 815, 771, 749, 723, 705, 681, 634, 614 cm^−1^. ^1^H-NMR (500 MHz, CDCl_3_): *δ* 0.76 (*t*, *J* = 7.4 Hz, 3H), 1.14 (*dp*, *J* = 7.3, 14.5 Hz, 1H), 1.63–1.75 (*m*, 1H), 1.64 (br *s*, 1H), 2.78 (*s*, 3H), 2.99 (*dd*, *J* = 7.5, 14.0 Hz, 1H), 3.25 (*dd*, *J* = 4.0, 14.0 Hz, 1H), 3.86 (*dd*, *J* = 4.0, 7.5 Hz, 1H), 4.37 (*ddd*, *J* = 1.2, 2.7, 7.3 Hz, 1H), 7.74 (*s*, 3H). ^13^C-NMR (126 MHz, CDCl_3_): *δ* 6.94, 26.97, 27.05, 38.70, 59.75, 74.59, 120.68 (*dt*, *J* = 3.9, 7.8 Hz), 123.50 (*q*, *J* = 272.6 Hz), 130.13 (q, *J* = 3.8 Hz), 131.56 (*q*, *J* = 33.2 Hz), 140.73, 172.92.

*Synthesis of* tert*-butyl (2*S*,5*S*)-5-(3,5-bis(trifluoromethyl)benzyl)-2-ethyl-3-methyl-4-oxoimidazolidine-1-carboxylate (**trans*****-7d***) and* tert*-butyl (2*R*,5*S*)-5-(3,5-bis(trifluoromethyl)benzyl)-2-ethyl-3-methyl-4-oxoimidazolidine-1-carboxylate (**cis*****-7d***)*

Following *GP5*: Prepared from (2*R*,5*S*)-5-(3,5-bis(trifluoromethyl)benzyl)-2-ethyl-3-methylimidazolidin-4-one (***trans*-6d**) and (2*S*,5*S*)-5-(3,5-bis(trifluoromethyl)benzyl)-2-ethyl-3-methylimidazolidin-4-one (***cis*-6d**) (1.967 mmol, 697 mg), CH_2_Cl_2_ (10 mL), Boc_2_O (4.327 mmol, 944 mg), Et_3_N (4.327 mmol, 603 μL), and DMAP (0.393 mmol, 48 mg); column chromatography: petroleum ether/EtOAc = 5:1.

***trans*-7d**: elutes first from the column. Yield: 107 mg (0.236 mmol, 12%) of yellowish oil. [*α*]_D_^r.t.^ = +63.8 (c = 1.0 mg/mL, CH_2_Cl_2_). EI-HRMS: *m/z* = 455.1761 (MH^+^); C_20_H_25_F_6_N_2_O_3_ requires *m/z* = 455.1764 (MH^+^); *ν*_max_ 2978, 2936, 1798, 1703, 1622, 1462, 1414, 1381, 1341, 1277, 1226, 1169, 1129, 1107, 1067, 1045, 975, 954, 938, 899, 843, 780, 740, 724, 709, 682, 654 cm^−1^. Two rotamers at a 72:28 ratio in CDCl_3_. ^1^H-NMR (500 MHz, CDCl_3_) for the major rotamer: *δ* 0.66 (*t*, *J* = 7.4 Hz, 3H), 1.49 (*s*, 9H), 1.51–1.58 (*m*, 1H), 2.02–2.14 (*m*, 1H), 2.62 (*s*, 3H), 3.29 (*dd*, *J* = 2.0, 13.6 Hz, 1H), 3.84 (*dd*, *J* = 5.5, 13.6 Hz, 1H), 4.22 (*q*, *J* = 2.3 Hz, 1H), 4.44 (*dt*, *J* = 2.1, 4.8 Hz, 1H), 7.60 (*s*, 2H), 7.74 (*s*, 1H). ^1^H-NMR (500 MHz, CDCl_3_) for the minor rotamer: *δ* 0.61 (*t*, *J* = 7.4 Hz, 3H), 1.59 (*s*, 9H), 2.32–2.42 (*m*, 1H), 2.64 (*s*, 3H), 3.34 (*dd*, *J* = 2.0, 13.7 Hz, 1H), 3.51 (*dd*, *J* = 5.6, 13.7 Hz, 1H), 4.28 (*q*, *J* = 2.4 Hz, 1H), 4.35 (*dt*, *J* = 2.2, 5.3 Hz, 1H), 7.58 (*s*, 2H). ^13^C-NMR (126 MHz, CDCl_3_) for both rotamers: *δ* 4.70, 21.65, 23.22, 26.31, 26.36, 28.37, 28.59, 33.94, 35.92, 60.07, 74.13, 74.37, 81.66, 81.81, 120.76–120.96 (*m*), 121.00–121.09 (*m*), 123.47 (*q*, *J* = 272.7 Hz), 130.03 (*d*, *J* = 3.5 Hz), 130.15–130.36 (*m*), 131.44 (*q*, *J* = 33.2 Hz), 138.75, 139.12, 151.79, 152.12, 168.83, 168.87.

***cis*-7d**: elutes second from the column. Yield: 232 mg (0.511 mmol, 26%) of yellowish oil. [*α*]_D_^r.t.^ = +9.3 (c = 1.35 mg/mL, CH_2_Cl_2_). EI-HRMS: *m/z* = 455.1761 (MH^+^); C_20_H_25_F_6_N_2_O_3_ requires *m/z* = 455.1764 (MH^+^); *ν*_max_ 2977, 2936, 1703, 1622, 1461, 1411, 1370, 1328, 1277, 1168, 1129, 1078, 1051, 992, 956, 900, 890, 844, 773, 724, 707, 682 cm^−1^. ^1^H-NMR (500 MHz, CDCl_3_): *δ* 0.56 (br *s*, 3H), 0.99 (br *s*, 1H), 1.18–1.34 (*m*, 1H), 1.46 (*s*, 9H), 2.78 (*s*, 3H), 3.32 (br *s*, 2H), 4.37 (br *s*, 1H), 4.85 (br *d*, 1H), 7.67 (*s*, 2H), 7.74 (*s*, 1H). ^13^C-NMR (126 MHz, CDCl_3_): *δ* 7.56, 26.42, 27.06, 28.35, 37.92, 60.58, 74.99, 81.88, 120.86, 123.44 (*q*, *J* = 272.7 Hz), 130.49, 131.67 (*q*, *J* = 32.9 Hz), 139.81, 154.15, 168.80.

*Synthesis of (2*S*,5*S*)-5-(3,5-bis(trifluoromethyl)benzyl)-2-ethyl-3-methylimidazolidin-4-one (**cis*****-6d***)*

Following *GP6*: Prepared from *tert*-butyl (2*R*,5*S*)-5-(3,5-bis(trifluoromethyl)benzyl)-2-ethyl-3-methyl-4-oxoimidazolidine-1-carboxylate (***cis*-7d**) (0.739 mmol, 336 mg), CH_2_Cl_2_ (7 mL), and CF_3_CO_2_H (7 mL), 2 h. Yield: 246 mg (0.695 mmol, 94%) of yellowish oil. For characterization of ***cis*-6d**, see above.

*Synthesis of (2*R*,5*S*)-5-(2,5-bis(trifluoromethyl)benzyl)-2-ethyl-3-methylimidazolidin-4-one (**trans*****-6f***) and (2*S*,5*S*)-5-(2,5-bis(trifluoromethyl)benzyl)-2-ethyl-3-methylimidazolidin-4-one (**cis*****-6f***)*

Following *GP4*: Prepared from (*S*)-2-amino-3-(2,5-bis(trifluoromethyl)phenyl)-*N*-methylpropanamide (**4b**) (4.170 mmol, 1.310 g), EtOH (40 mL), and propanal (4.800 mmol, ω = 0.97, 357 μL); column chromatography: petroleum ether/EtOAc = 1:1 to elute ***trans*-6f** and ***cis*-6f** as a mixture. The two diastereomers could not be separated by column chromatography; ***trans*-6f**/***cis*-6f** = 47:53 (after column chromatography in CDCl_3_). Yield: 960 mg (2.7105 mmol, 65%) of yellowish oil.

*Synthesis of* tert*-butyl (2*S*,5*S*)-5-(2,5-bis(trifluoromethyl)benzyl)-2-ethyl-3-methyl-4-oxoimidazolidine-1-carboxylate (**trans*****-7f***) and* tert*-butyl (2*R*,5*S*)-5-(2,5-bis(trifluoromethyl)benzyl)-2-ethyl-3-methyl-4-oxoimidazolidine-1-carboxylate (**cis*****-7f***)*

Following *GP5*: Prepared from (2*R*,5*S*)-5-(2,5-bis(trifluoromethyl)benzyl)-2-ethyl-3-methylimidazolidin-4-one (***trans*-6f**) and (2*S*,5*S*)-5-(2,5-bis(trifluoromethyl)benzyl)-2-ethyl-3-methylimidazolidin-4-one (***cis*-6f**) (***trans*-6f**/***cis*-6f** = 47:53, 2.710 mmol, 960 mg), CH_2_Cl_2_ (6 mL), Boc_2_O (5.962 mmol, 1.301 g), Et_3_N (5.962 mmol, 831 μL), and DMAP (0.542 mmol, 66 mg); first column chromatography: petroleum ether/EtOAc = 10:1; second column chromatography: petroleum ether/EtOAc = 5:1 for the separation of mixed fractions ***trans*-7f**/***cis*-7f** obtained after the first column chromatography.

***trans*-7f**: elutes first from the column. Yield: 431 mg (0.9485 mmol, 35%) of orange oil. [*α*]_D_^r.t.^ = +34.3 (c = 2.1 mg/mL, CH_2_Cl_2_). EI-HRMS: *m/z* = 455.1766 (MH^+^); C_20_H_25_F_6_N_2_O_3_ requires *m/z* = 455.1764 (MH^+^); *ν*_max_ 2978, 2937, 2884, 1785, 1702, 1460, 1413, 1381, 1312, 1268, 1164, 1118, 1088, 1040, 964, 923, 901, 840, 784, 766, 750, 717, 645 cm^−1^. Two rotamers at a 67:33 ratio in CDCl_3_. ^1^H-NMR (500 MHz, CDCl_3_) for the major rotamer: *δ* 0.71 (*t*, *J* = 7.3 Hz, 3H), 1.49 (*s*, 9H), 1.65–1.79 (*m*, 1H), 2.53–2.65 (m, 1H), 2.84 (*s*, 3H), 3.34–3.46 (*m*, 1H), 3.53 (*dd*, *J* = 5.0, 15.2 Hz, 1H), 4.50 (*t*, *J* = 5.6 Hz, 1H), 5.09 (*s*, 1H), 7.56–7.83 (*m*, 3H). ^1^H-NMR (500 MHz, CDCl_3_) for the minor rotamer: *δ* 0.76 (*t*, *J* = 7.4 Hz, 3H), 1.54 (*s*, 9H), 1.77–1.89 (*m*, 1H), 2.13–2.25 (*m*, 1H), 2.79 (s, 3H), 3.74 (*dd*, *J* = 4.6, 15.1 Hz, 1H), 4.56 (*t*, *J* = 5.8 Hz, 1H). ^13^C-NMR (126 MHz, CDCl_3_) for both rotamers: *δ* 4.49, 4.66, 20.96, 23.63, 26.63, 26.65, 27.41, 27.52, 32.37 (*d*, *J* = 2.3 Hz), 34.95 (*d*, *J* = 2.3 Hz), 59.40, 59.56, 74.17, 74.30, 85.86, 86.13, 123.41 (*q*, *J* = 272.9 Hz), 123.71 (*q*, *J* = 274.3 Hz), 123.75 (*q*, *J* = 3.8 Hz), 124.01 (*q*, *J* = 3.8 Hz), 126.89–127.22 (*m*), 128.05 (*q*, *J* = 3.6 Hz), 128.50 (*q*, *J* = 3.9 Hz), 132.60 (*q*, *J* = 30.0 Hz), 132.71 (*q*, *J* = 30.4 Hz), 133.56 (*q*, *J* = 33.2 Hz), 133.72 (*q*, *J* = 32.7 Hz), 136.40, 136.77, 146.20, 146.35, 146.69, 146.92, 167.78, 167.96.

***cis*-7f**: elutes second from the column. Yield: 259 mg (0.569 mmol, 21%) of yellowish oil. EI-HRMS: *m/z* = 455.1764 (MH^+^); C_20_H_25_F_6_N_2_O_3_ requires *m/z* = 455.1764 (MH^+^). ^1^H-NMR (300 MHz, CDCl_3_): *δ* 0.91 (*t*, *J* = 7.4 Hz, 3H), 1.23 (br *s*, 10H), 1.84 (br *s*, 1H), 2.86 (*s*, 3H), 3.09 (br *s*, 1H), 3.55 (*dd*, *J* = 4.6, 14.6 Hz, 1H), 4.46 (br *s*, 1H), 5.01 (br *s*, 1H), 7.61 (br *d*, *J* = 8.2 Hz, 1H), 7.71 (*s*, 1H), 7.79 (br *d*, *J* = 8.2 Hz, 1H). ^13^C-NMR (126 MHz, CDCl_3_): *δ* 8.03, 27.05, 27.23, 27.93, 36.22, 60.71, 75.46, 81.46, 123.43 (*q*, *J* = 272.5 Hz), 123.72, 123.76 (*q*, *J* = 274.3 Hz), 126.89 (*q*, *J* = 5.7 Hz), 129.38, 132.75 (*q*, *J* = 30.5 Hz), 133.70, 138.14, 154.54, 169.19.

*Synthesis of (2*S*,5*S*)-5-(2,5-bis(trifluoromethyl)benzyl)-2-ethyl-3-methylimidazolidin-4-one (**cis*****-6f***)*

Following *GP6*: Prepared from *tert*-butyl (2*R*,5*S*)-5-(2,5-bis(trifluoromethyl)benzyl)-2-ethyl-3-methyl-4-oxoimidazolidine-1-carboxylate (***cis*-7f**) (0.810 mmol, 368.1 mg), CH_2_Cl_2_ (5 mL), and CF_3_CO_2_H (5 mL), 2 h. Yield: 250 mg (0.7047 mmol, 87%) of yellowish oil. [*α*]_D_^r.t.^ = –23.5 (c = 1.15 mg/mL, CH_2_Cl_2_). EI-HRMS: *m/z* = 355.1242 (MH^+^); C_15_H_17_F_6_N_2_O requires *m/z* = 355.1240 (MH^+^); *ν*_max_ 3345, 2980, 2968, 2939, 1680, 1508, 1484, 1460, 1442, 1422, 1405, 1339, 1311, 1292, 1278, 1241, 1199, 1163, 1115, 1088, 1073, 1059, 1039, 994, 961, 930, 913, 893, 880, 835, 818, 778, 750, 729, 678, 639 cm^−1^. ^1^H-NMR (500 MHz, CDCl_3_): *δ* 0.83 (*t*, *J* = 7.4 Hz, 3H), 1.41 (*dp*, *J* = 7.3, 14.4 Hz, 1H), 1.72–1.83 (*m*, 2H), 2.83 (*s*, 3H), 2.94 (*dd*, *J* = 9.1, 14.8 Hz, 1H), 3.58 (*dd*, *J* = 3.3, 14.8 Hz, 1H), 3.84 (*dd*, *J* = 3.6, 8.9 Hz, 1H), 4.38 (*d*, *J* = 5.5 Hz, 1H), 7.60 (*d*, *J* = 8.2 Hz, 1H), 7.78 (*d*, *J* = 8.2 Hz, 1H), 7.82 (*s*, 1H). ^13^C-NMR (126 MHz, CDCl_3_): *δ* 6.86, 26.83, 27.00, 35.57, 59.57, 74.54, 123.48 (*q*, *J* = 272.9 Hz), 123.65 (*q*, *J* = 3.7 Hz), 123.86 (*q*, *J* = 274.3 Hz), 126.83 (*q*, *J* = 5.7 Hz), 128.93 (*q*, *J* = 3.6 Hz), 132.53 (*q*, *J* = 29.9 Hz), 133.83 (*q*, *J* = 32.4 Hz), 138.74 (*d*, *J* = 1.2 Hz), 173.46.

*Synthesis of (2*R*,5*S*)-5-(3,5-dimethoxybenzyl)-2-ethyl-3-methylimidazolidin-4-one (**trans*****-6g***) and (2*S*,5*S*)-5-(3,5-dimethoxybenzyl)-2-ethyl-3-methylimidazolidin-4-one (**cis*****-6g***)*

Following *GP4*: Prepared from (*S*)-2-amino-3-(3,5-dimethoxyphenyl)-*N*-methylpropanamide (**4c**) (5.061 mmol, 1.206 g), EtOH (40 mL), and propanal (5.820 mmol, ω = 0.97, 433 μL); column chromatography: (1) petroleum ether/EtOAc = 5:1, then (2) petroleum ether/EtOAc = 2:1; ***trans*-6g**/***cis*-6g** = 46:54 (crude reaction mixture in CDCl_3_).

***trans*-6g**: elutes first from the column. Yield: 268 mg (0.660 mmol, 19%) of yellowish oil. [*α*]_D_^r.t.^ = –74.5 (c = 3.1 mg/mL, CH_2_Cl_2_). EI-HRMS: *m/z* = 279.1700 (MH^+^); C_15_H_23_N_2_O_3_ requires *m/z* = 279.1703 (MH^+^); *ν*_max_ 3336, 2933, 2838, 1683, 1593, 1459, 1428, 1402, 1316, 1293, 1204, 1148, 1064, 991, 927, 828, 695, 634 cm^−1^. ^1^H-NMR (500 MHz, CDCl_3_): *δ* 0.86 (*t*, *J* = 7.4 Hz, 3H), 1.48 (*dp*, *J* = 7.3, 14.3 Hz, 1H), 1.62–1.73 (*m*, 1H), 1.99 (br *s*, 1H), 2.76 (*s*, 3H), 2.85 (*dd*, *J* = 7.3, 13.9 Hz, 1H), 3.02 (*dd*, *J* = 4.1, 13.9 Hz, 1H), 3.76 (*s*, 6H), 3.81 (*ddd*, *J* = 1.5, 4.3, 6.1 Hz, 1H), 4.15 (*ddd*, *J* = 1.6, 2.8, 6.9 Hz, 1H), 6.33 (*t*, *J* = 2.2 Hz, 1H), 6.41 (*d*, *J* = 2.2 Hz, 2H). ^13^C-NMR (126 MHz, CDCl_3_): *δ* 7.48, 26.54, 27.02, 38.34, 55.41, 59.91, 75.31, 98.96, 107.44, 139.98, 160.92, 174.18.

***cis*-6g**: elutes second from the column. Yield: 282 mg (1.012 mmol, 20%) of yellowish oil. [*α*]_D_^r.t.^ = –47.6 (c = 2.15 mg/mL, CH_2_Cl_2_). EI-HRMS: *m/z* = 279.1701 (MH^+^); C_15_H_23_N_2_O_3_ requires *m/z* = 279.1703 (MH^+^); *ν*_max_ 3338, 2933, 2838, 1685, 1592, 1685, 1592, 1458, 1428, 1403, 1346, 1315, 1295, 1204, 1148, 1057, 992, 923, 830, 696 cm^−1^. ^1^H-NMR (500 MHz, CDCl_3_): *δ* 0.71 (*t*, *J* = 7.5 Hz, 3H), 1.35 (*dp*, *J* = 7.3, 14.4 Hz, 1H), 1.65–1.76 (*m*, 1H), 1.82 (br *s*, 1H), 2.77 (*s*, 3H), 2.91 (*dd*, *J* = 7.1, 14.0 Hz, 1H), 3.10 (*dd*, *J* = 4.2, 14.0 Hz, 1H), 3.70 (*dd*, *J* = 4.4, 6.8 Hz, 1H), 3.76 (*s*, 6H), 4.30 (*ddd*, *J* = 1.3, 2.8, 6.6 Hz, 1H), 6.33 (*t*, *J* = 2.2 Hz, 1H), 6.41 (*d*, *J* = 2.2 Hz, 2H). ^13^C-NMR (126 MHz, CDCl_3_): *δ* 6.64, 25.90, 26.98, 37.93, 55.45, 60.24, 74.66, 99.03, 107.48, 139.78, 161.06, 174.74.

*Synthesis of* tert*-butyl (2*R*,5*S*)-5-(3,5-dimethoxybenzyl)-2-ethyl-3-methyl-4-oxoimidazolidine-1-carboxylate (**cis*****-7g***) and (2*R*,5*S*)-5-(3,5-dimethoxybenzyl)-2-ethyl-3-methylimidazolidin-4-one (**trans*****-6g***)*

Following *GP5*: Prepared from (2*R*,5*S*)-5-(3,5-dimethoxybenzyl)-2-ethyl-3-methylimidazolidin-4-one (***trans*-6g**) and (2*S*,5*S*)-5-(3,5-dimethoxybenzyl)-2-ethyl-3-methylimidazolidin-4-one (***cis*-6g**) (1.275 mmol, 355 mg), CH_2_Cl_2_ (6 mL), Boc_2_O (2.805 mmol, 612 mg), Et_3_N (2.805 mmol, 391 μL), and DMAP (0.255 mmol, 31 mg). First column chromatography: petroleum ether/EtOAc = 1:1; ***trans*-6g** and ***cis*-7g** did not separate, but several other impurities were removed. Second column chromatography: petroleum ether/EtOAc = 1:1; ***trans*-6g** and ***cis*-7g** separated.

***cis*-7g**: elutes first from the column. Yield: 159 mg (0.421 mmol, 33%) of yellowish oil. [*α*]_D_^r.t.^ = +54.6 (c = 0.9 mg/mL, CH_2_Cl_2_). EI-HRMS: *m/z* = 379.222 (MH^+^); C_20_H_31_N_2_O_5_ requires *m/z* = 379.2227 (MH^+^); *ν*_max_ 2971, 2932, 1698, 1594, 1458, 1430, 1409, 1367, 1328, 1295, 1256, 1204, 1150, 1064, 963, 920, 838, 771, 696 cm^−1^. Two rotamers at a 68:32 ratio in CDCl_3_. ^1^H-NMR (500 MHz, CDCl_3_) for the major rotamer: *δ* 0.73 (br *s*, 3H), 1.01 (br *s*, 1H), 1.26 (br *s*, 1H), 1.44 (br *s*, 9H), 2.80 (*s*, 3H), 2.98–3.28 (*m*, 2H), 3.74 (*s*, 6H), 4.33 (br *s*, 1H), 4.90 (br *s*, 1H), 6.32 (*s*, 1H), 6.36 (*d*, *J* = 2.2 Hz, 2H). ^1^H-NMR (500 MHz, CDCl_3_) for the minor rotamer: *δ* 4.75 (br *s*, 1H). ^13^C-NMR (126 MHz, CDCl_3_) for both rotamers: *δ* 8.46, 26.90, 27.08, 28.28, 36.76, 38.32, 55.29, 61.01, 75.11, 80.98, 99.25, 107.95, 139.20, 154.33, 160.68, 169.50.

***trans*-6g**: elutes second from the column. Yield: 82 mg (0.291 mmol, 23%) of yellowish oil. For full characterization data, see above.

*Synthesis of (2*S*,5*S*)-5-(3,5-dimethoxybenzyl)-2-ethyl-3-methylimidazolidin-4-one (**cis*****-6g***)*

Following *GP6*: Prepared from tert-butyl (2R,5S)-5-(3,5-dimethoxybenzyl)-2-ethyl-3-methyl-4-oxoimidazolidine-1-carboxylate (***cis*-7g**) (0.317 mmol, 120 mg), CH_2_Cl_2_ (3 mL), and CF_3_CO_2_H (3 mL), 2 h. Yield: 79 mg (0.284 mmol, 89%) of yellowish oil. For characterization of ***cis*-6g**, see above.

*Synthesis of (2*R*,5*S*)-5-([1,1′-biphenyl]-3-ylmethyl)-3-methyl-2-((*Z*)-pent-2-en-2-yl)imidazolidin-4-one (**trans*****-6h′***), (2*S*,5*S*)-5-([1,1′-biphenyl]-3-ylmethyl)-3-methyl-2-((Z)-pent-2-en-2-yl)imidazolidin-4-one (**cis*****-6h′***), (2*R*,5*S*)-5-([1,1′-biphenyl]-3-ylmethyl)-2-ethyl-3-methylimidazolidin-4-one (**trans*****-6h***), and (2*S*,5*S*)-5-([1,1′-biphenyl]-3-ylmethyl)-2-ethyl-3-methylimidazolidin-4-one (**cis*****-6h***)*

Following *GP4*: Prepared from (*S*)-3-([1,1′-biphenyl]-3-yl)-2-amino-*N*-methylpropanamide (**4d**) (5.0 mmol, 1.272 g), EtOH (35 mL), and propanal (5.750 mmol, ω = 0.97, 428 μL); column chromatography: EtOAc; ***trans*-6h**/***cis*-6h** = 50:50 (crude reaction mixture in CDCl_3_).

***trans*-6h′**: elutes first from the column. Yield: 50 mg (0.150 mmol, 3%) of yellowish oil. [*α*]_D_^r.t.^ = –35.1 (c = 1.65 mg/mL, CH_2_Cl_2_). EI-HRMS: *m/z* = 335.2119 (MH^+^); C_22_H_27_N_2_O requires *m/z* = 335.2118 (MH^+^); *ν*_max_ 3331, 3033, 2962, 2872, 1684, 1599, 1479, 1421, 1400, 1333, 1312, 1269, 1167, 1119, 1089, 1040, 996, 897, 878, 835, 797, 753, 699, 637, 616 cm^−1^. ^1^H-NMR (500 MHz, CDCl_3_): *δ* 0.94 (*t*, *J* = 7.6 Hz, 3H), 1.45 (*d*, *J* = 1.3 Hz, 3H), 1.92 (br *s*, 1H), 2.03 (*p*, *J* = 7.4 Hz, 2H), 2.62 (*s*, 3H), 2.91 (*dd*, *J* = 7.9, 13.7 Hz, 1H), 3.19 (*dd*, *J* = 3.8, 13.7 Hz, 1H), 3.98 (*ddd*, *J* = 2.1, 3.8, 8.0 Hz, 1H), 4.40 (*d*, *J* = 2.0 Hz, 1H), 5.42 (*td*, *J* = 1.5, 7.1 Hz, 1H), 7.22–7.28 (*m*, 1H), 7.31–7.52 (*m*, 6H), 7.55–7.61 (*m*, 2H). ^13^C-NMR (126 MHz, CDCl_3_): *δ* 9.40, 13.85, 21.16, 26.65, 39.38, 60.55, 81.49, 125.58, 127.24, 127.41, 128.39, 128.51, 128.86, 129.00, 132.15, 134.27, 138.49, 141.08, 141.48, 173.74.

***cis*-6h′**: elutes second from the column. Yield: 17 mg (0.05 mmol, 1%) of yellowish oil. [*α*]_D_^r.t.^ = –29.1 (c = 1.05 mg/mL, CH_2_Cl_2_). EI-HRMS: *m/z* = 335.2113 (MH^+^); C_22_H_27_N_2_O requires *m/z* = 335.2118 (MH^+^); *ν*_max_ 3339, 3032, 2962, 2929, 2872, 1692, 1599, 1479, 1453, 1422, 1399, 1326, 1268, 1156, 1096, 1076, 1043, 1002, 900, 871, 833, 801, 753, 699, 650, 615 cm^−1^. ^1^H-NMR (500 MHz, CDCl_3_): *δ* 0.94 (*t*, *J* = 7.5 Hz, 3H), 1.17 (*s*, 3H), 1.80 (br *s*, 1H), 1.97–2.06 (*m*, 2H), 2.64 (s, 3H), 3.08 (*dd*, *J* = 7.2, 13.9 Hz, 1H), 3.24 (*dd*, *J* = 4.1, 13.9 Hz, 1H), 3.79–3.85 (*m*, 1H), 4.59 (*d*, *J* = 1.7 Hz, 1H), 5.51 (*td*, *J* = 1.5, 7.1 Hz, 1H), 7.22–7.28 (*m*, 1H), 7.31–7.39 (*m*, 2H), 7.40–7.49 (*m*, 4H), 7.54–7.60 (*m*, 2H). ^13^C-NMR (126 MHz, CDCl_3_): *δ* 8.88, 13.79, 21.28, 26.84, 37.96, 60.09, 81.10, 125.72, 127.28, 127.45, 128.54, 128.66, 128.91, 129.21, 131.35, 135.35, 138.20, 141.15, 141.68, 174.15.

***trans*-6h**: elutes first from the column. Yield: 412 mg (1.40 mmol, 28%) of yellowish oil. [*α*]_D_^r.t.^ = –78.5 (c = 1.0 mg/mL, CH_2_Cl_2_). EI-HRMS: *m/z* = 295.1804 (MH^+^); C_19_H_23_N_2_O requires *m/z* = 295.1805 (MH^+^); *ν*_max_ 3475, 3325, 3057, 3032, 2964, 2924, 2875, 1681, 1599, 1574, 1478, 1454, 1431, 1402, 1343, 1267, 1116, 1076, 1025, 987, 939, 900, 797, 755, 700, 633, 616 cm^−1^. ^1^H-NMR (500 MHz, CDCl_3_): *δ* 0.86 (*t*, *J* = 7.4 Hz, 3H), 1.41–1.53 (*m*, 1H), 1.62–1.73 (*m*, 1H), 1.90 (br *s*, 1H), 2.73 (*s*, 3H), 2.98 (*dd*, *J* = 7.3, 13.9 Hz, 1H), 3.15 (*dd*, *J* = 4.1, 13.9 Hz, 1H), 3.85–3.91 (*m*, 1H), 4.08 (*ddd*, *J* = 1.5, 2.7, 6.8 Hz, 1H), 7.22–7.28 (*m*, 1H), 7.31–7.39 (*m*, 2H), 7.40–7.49 (*m*, 3H), 7.49–7.52 (*m*, 1H), 7.56–7.62 (*m*, 2H). ^13^C-NMR (126 MHz, CDCl_3_): *δ* 7.40, 26.44, 26.96, 38.36, 60.14, 75.35, 125.60, 127.22, 127.42, 128.47, 128.59, 128.87, 128.98, 138.23, 141.04, 141.42, 174.29.

***cis*-6h**: elutes second from the column. Yield: 162 mg (0.550 mmol, 11%) of yellowish oil. [*α*]_D_^r.t.^ = –40.2 (c = 1.05 mg/mL, CH_2_Cl_2_). EI-HRMS: *m/z* = 295.1804 (MH^+^); C_19_H_23_N_2_O requires *m/z* = 295.1805 (MH^+^); *ν*_max_ 3497, 3338, 3058, 3031, 2964, 2924, 2874, 1684, 1599, 1574, 1479, 1454, 1422, 1403, 1346, 1272, 1077, 1025, 994, 899, 802, 755, 701, 647, 615 cm^−1^. ^1^H-NMR (500 MHz, CDCl_3_): *δ* 0.66 (*t*, *J* = 7.5 Hz, 3H), 1.22–1.36 (*m*, 1H), 1.62–1.73 (*m*, 1H), 1.75 (br *s*, 1H), 2.75 (*s*, 3H), 3.03 (*dd*, *J* = 7.1, 14.0 Hz, 1H), 3.22 (*dd*, *J* = 4.2, 14.0 Hz, 1H), 3.75 (*dd*, *J* = 4.5, 6.7 Hz, 1H), 4.25–4.31 (*m*, 1H), 7.20–7.26 (*m*, 1H), 7.30–7.38 (*m*, 2H), 7.38–7.50 (*m*, 4H), 7.53–7.59 (*m*, 2H). ^13^C-NMR (126 MHz, CDCl_3_): *δ* 6.42, 25.79, 26.84, 37.72, 60.23, 74.48, 125.56, 127.10, 127.31, 128.32, 128.44, 128.77, 129.01, 137.96, 140.94, 141.43, 174.46.

*Synthesis of (2*R*,5*S*)-2-ethyl-3-methyl-5-((2-methylnaphthalen-1-yl)methyl)imidazolidin-4-one (**trans*****-6i***) and (2*S*,5*S*)-2-ethyl-3-methyl-5-((2-methylnaphthalen-1-yl)methyl)imidazolidin-4-one (**cis*****-6i***)*

Following *GP4*: Prepared from (*S*)-2-amino-*N*-methyl-3-(2-methylnaphthalen-1-yl)propanamide (**4e**) (3.110 mmol, 754 mg), EtOH (20 mL), and propanal (3.5765 mmol, ω = 0.97, 266 μL); column chromatography: EtOAc to elute ***trans*-6i** and ***cis*-6i** as a mixture. The two diastereomers could not be separated by column chromatography; ***trans*-6i**/***cis*-6i** = 60:40 (after column chromatography in CDCl_3_). Yield: 580 mg (2.053 mmol, 66%) of yellowish oil.

*Synthesis of* tert*-butyl (2*S*,5*S*)-2-ethyl-3-methyl-5-((2-methylnaphthalen-1-yl)methyl)-4-oxoimidazolidine-1-carboxylate (**trans*****-7i***),* tert*-butyl (2*R*,5*S*)-2-ethyl-3-methyl-5-((2-methylnaphthalen-1-yl)methyl)-4-oxoimidazolidine-1-carboxylate (**cis*****-7i***), and (2*R*,5*S*)-2-ethyl-3-methyl-5-((2-methylnaphthalen-1-yl)methyl)imidazolidin-4-one (**trans*****-6i***)*

Following *GP5*: Prepared from (2*R*,5*S*)-2-ethyl-3-methyl-5-((2-methylnaphthalen-1-yl)methyl)imidazolidin-4-one (***trans*-6i**) and (2*S*,5*S*)-2-ethyl-3-methyl-5-((2-methylnaphthalen-1-yl)methyl)imidazolidin-4-one (***cis*-6i**) (***trans*-6i**/***cis*-6i** = 60:40 1.985 mmol, 561 mg), CH_2_Cl_2_ (7 mL), Boc_2_O (4.367 mmol, 953 mg), Et_3_N (4.367 mmol, 609 μL), and DMAP (0.397 mmol, 49 mg); column chromatography: petroleum ether/EtOAc = 2:1.

***trans*-7i**: elutes first from the column. Yield: 76 mg (0.1985 mmol, 10%) of yellowish oil. [*α*]_D_^r.t.^ = –73.2 (c = 1.6 mg/mL, CH_2_Cl_2_). EI-HRMS: *m/z* = 383.2322 (MH^+^); C_23_H_31_N_2_O_3_ requires *m/z* = 383.2329 (MH^+^); *ν*_max_ 2967, 2926, 1709, 1688, 1599, 1476, 1455, 1433, 1409, 1379, 1363, 1317, 1262, 1164, 1124, 1086, 1041, 972, 913, 861, 822, 795, 779, 755, 672, 617 cm^−1^. Two rotamers at a 66:34 ratio in CDCl_3_. ^1^H-NMR (500 MHz, CDCl_3_) for the major rotamer: *δ* 0.55–0.64 (*m*, 3H), 1.18 (*s*, 9H), 1.61–1.70 (*m*, 1H), 2.48 (*s*, 3H), 2.59 (*ddd*, *J* = 2.8, 7.6, 15.3 Hz, 1H), 2.78 (*s*, 3H), 3.50 (*dd*, *J* = 6.5, 14.4 Hz, 1H), 3.70 (*dd*, *J* = 7.3, 14.3 Hz, 1H), 4.55–4.63 (*m*, 1H), 5.10 (*d*, *J* = 2.4 Hz, 1H), 7.30 (*d*, *J* = 8.2 Hz, 1H), 7.34–7.43 (*m*, 1H), 7.43–7.53 (*m*, 1H), 7.66 (*d*, *J* = 8.3 Hz, 1H), 7.79 (*d*, *J* = 8.1 Hz, 1H), 8.15 (*d*, *J* = 8.5 Hz, 1H). ^1^H NMR (500 MHz, CDCl_3_) for the minor rotamer: *δ* 1.47 (*s*, 9H), 2.18–2.26 (*m*, 1H), 2.50 (*s*, 3H), 2.67 (*s*, 3H), 3.43 (*dd*, *J* = 8.2, 14.0 Hz, 1H), 4.03 (*dd*, *J* = 4.6, 14.0 Hz, 1H), 4.85 (*s*, 1H), 7.63 (*d*, *J* = 8.4 Hz, 1H), 7.76 (*d*, *J* = 8.4 Hz, 1H), 8.32 (*d*, *J* = 8.4 Hz, 1H). ^13^C-NMR (126 MHz, CDCl_3_) for the major rotamer: *δ* 4.50, 20.92, 21.28, 26.54, 28.00, 33.09, 58.70, 73.84, 81.08, 123.84, 124.66, 126.00, 127.03, 128.72, 129.38, 130.53, 132.59, 132.95, 135.04, 152.51, 169.78. ^13^C NMR (126 MHz, CDCl_3_) for the minor rotamer: *δ* 21.12, 23.05, 26.47, 28.53, 30.65, 58.41, 73.49, 80.99, 124.37, 124.43, 126.90, 128.48, 129.23, 130.31, 132.54, 133.03, 135.43, 152.37, 169.56.

***cis*-7i**: elutes second from the column. Yield: 197 mg (0.516 mmol, 26%) of yellowish solid, m.p. = 125–128 °C. [*α*]_D_^r.t.^ = –23.0 (c = 1.0 mg/mL, CH_2_Cl_2_). EI-HRMS: *m/z* = 383.2331 (MH^+^); C_23_H_31_N_2_O_3_ requires *m/z* = 383.2329 (MH^+^); *ν*_max_ 3051, 2971, 2933, 2876, 1697, 1375, 1254, 1156, 1034, 915, 859, 817, 747 cm^−1^. ^1^H-NMR (500 MHz, CDCl_3_): 0.44–1.13 (*m*, 9H), 1.48 (br *s*, 3H), 1.99 (br *s*, 1H), 2.52 (*s*, 3H), 2.85 (br *s*, 3H), 3.45 (br *s*, 1H), 3.71 (*d*, *J* = 11.8 Hz, 1H), 4.58 (*t*, *J* = 6.7 Hz, 1H), 5.00 (br *d*, *J* = 11.2 Hz, 1H), 7.28 (*d*, *J* = 8.4 Hz, 1H), 7.33–7.42 (*m*, 1H), 7.43–7.50 (*m*, 1H), 7.64 (*d*, *J* = 8.3 Hz, 1H), 7.76 (*d*, *J* = 8.1 Hz, 1H), 8.21 (br *s*, 1H) (one signal missing). ^13^C-NMR (126 MHz, CDCl_3_): *δ* 8.45, 20.81, 27.18, 27.53, 33.06, 60.06, 75.62, 80.82, 123.92, 124.51, 125.95, 126.88, 128.44, 129.12, 131.53, 132.53, 133.02, 134.42, 154.65, 170.07 (one signal missing).

***trans*-6i**: elutes third from the column. Yield: 157 mg (0.556 mmol, 28%) of yellowish oil. [*α*]_D_^r.t.^ = –28.78 (c = 3.75 mg/mL, CH_2_Cl_2_). EI-HRMS: *m/z* = 283.1809 (MH^+^); C_18_H_23_N_2_O requires *m/z* = 283.1805 (MH^+^); *ν*_max_ 3307, 3049, 2965, 2935, 2875, 1683, 1597, 1570, 1511, 1433, 1401, 1349, 1268, 1166, 1127, 1089, 1072, 1028, 970, 906, 862, 810, 783, 741, 684, 640 cm^−1^. ^1^H-NMR (500 MHz, CDCl_3_): *δ* 0.77 (*t*, *J* = 7.4 Hz, 3H), 1.45 (*dp*, *J* = 7.3, 14.3 Hz, 1H), 1.61–1.73 (*m*, 2H), 2.57 (*s*, 3H), 2.81 (*s*, 3H), 3.22 (*dd*, *J* = 10.1, 14.3 Hz, 1H), 3.64 (*dd*, *J* = 3.4, 14.4 Hz, 1H), 3.92–3.99 (*m*, 1H), 4.38 (*ddd*, *J* = 1.7, 2.8, 6.6 Hz, 1H), 7.30 (*d*, *J* = 8.4 Hz, 1H), 7.36–7.43 (*m*, 1H), 7.45–7.52 (*m*, 1H), 7.64 (*d*, *J* = 8.4 Hz, 1H), 7.78 (*d*, *J* = 8.0 Hz, 1H), 8.16 (*d*, *J* = 8.6 Hz, 1H). ^13^C-NMR (126 MHz, CDCl_3_): *δ* 7.07, 21.04, 26.23, 26.84, 31.17, 60.05, 75.16, 123.83, 124.67, 126.11, 126.83, 128.65, 129.28, 131.95, 132.39, 132.60, 134.76, 174.76.

*Synthesis of (2*S*,5*S*)-2-ethyl-3-methyl-5-((2-methylnaphthalen-1-yl)methyl)imidazolidin-4-one (**cis*****-6i***)*

Following *GP6*: Prepared from *tert*-butyl (2*R*,5*S*)-2-ethyl-3-methyl-5-((2-methylnaphthalen-1-yl)methyl)-4-oxoimidazolidine-1-carboxylate (***cis*-7i**) (0.482 mmol, 184.1 mg), CH_2_Cl_2_ (5 mL), and CF_3_CO_2_H (5 mL), 2 h. Yield: 132 mg (0.4675 mmol, 97%) of yellowish oil. [*α*]_D_^r.t.^ = –1.52 (c = 0.8 mg/mL, CH_2_Cl_2_). EI-HRMS: *m/z* = 283.1808 (MH^+^); C_18_H_23_N_2_O requires *m/z* = 283.1805 (MH^+^); *ν*_max_ 3329, 3049, 2964, 2874, 1685, 1598, 1511, 1433, 1403, 1382, 1350, 1271, 1114, 1045, 973, 863, 809, 780, 742, 686 cm^−1^. ^1^H-NMR (500 MHz, CDCl_3_): *δ* 0.82 (*t*, *J* = 7.4 Hz, 3H), 1.46 (*dp*, *J* = 7.3, 14.2 Hz, 1H), 1.67–1.76 (*m*, 1H), 1.78 (br *s*, 1H), 2.61 (*s*, 3H), 2.83 (*s*, 3H), 3.30 (*dd*, *J* = 9.7, 14.4 Hz, 1H), 3.81 (*dd*, *J* = 3.3, 14.4 Hz, 1H), 3.92 (*dd*, *J* = 2.5, 9.6 Hz, 1H), 4.26 (*ddd*, *J* = 1.5, 2.7, 6.8 Hz, 1H), 7.31 (*d*, *J* = 8.4 Hz, 1H), 7.35–7.44 (*m*, 1H), 7.43–7.51 (*m*, 1H), 7.64 (*d*, *J* = 8.4 Hz, 1H), 7.79 (*d*, *J* = 7.9 Hz, 1H), 8.19 (*d*, *J* = 8.6 Hz, 1H). ^13^C-NMR (126 MHz, CDCl_3_): *δ* 6.99, 21.14, 26.91, 26.94, 31.85, 60.08, 74.28, 124.12, 124.77, 126.11, 126.88, 128.68, 129.40, 132.08, 132.49, 132.71, 134.71, 174.48.

*Synthesis of (2*R*,5*S*)-5-(3,5-bis(trifluoromethyl)benzyl)-2-(*tert*-butyl)-3-methylimidazolidin-4-one (**trans*****-6e***) and (2*S*,5*S*)-5-(3,5-bis(trifluoromethyl)benzyl)-2-(*tert*-butyl)-3-methylimidazolidin-4-one (**cis*****-6e***)*

To a suspension of (*S*)-2-amino-3-(3,5-bis(trifluoromethyl)phenyl)-*N*-methylpropanamide (**4a**) (2.60 mmol, 817 mg) and anhydrous FeCl_3_ (0.52 mmol, 85 mg) in anhydrous toluene (7 mL) under argon; pivalaldehyde (2.68 mmol, ω = 0.98, 303 μL) was added. The reaction mixture was heated at reflux under argon using a Dean–Stark apparatus filled with freshly dried molecular sieves (4 Å MS) for 10 h. After cooling, saturated aqueous NaHCO_3_ (50 mL) was added, and the mixture was extracted with EtOAc (3 × 80 mL). The combined organic phase was dried over anhydrous Na_2_SO_4_, filtered, and the volatile components were evaporated in vacuo. The residue was purified by flash column chromatography (EtOAc, ***trans*-6e**:***cis*-6e** = 70:30), followed by MPLC (EtOAc/petroleum ether = 2:1).

***trans*-6e**: elutes first from the column. Yield: 288 mg (0.754 mmol, 29%, 84% *ee*) of yellowish oil. [*α*]_D_^r.t.^ = –27.1 (c = 1.2 mg/mL, CH_2_Cl_2_). HPLC (Chiralcel OD-H, *n*-hexane/*i*-PrOH = 96:4, flow rate 1.0 mL/min, λ = 205 nm): *t*R = 8.9 min (major); 12.9 min (minor). EI-HRMS: *m/z* = 383.1551 (MH^+^); C_17_H_21_F_6_N_2_O requires *m/z* = 383.1553 (MH^+^); *ν*_max_ 3365, 2962, 1694, 1622, 1484, 1434, 1401, 1381, 1341, 1280, 1173, 1127, 1101, 925, 899, 844, 724, 708, 683 cm^−1^. ^1^H-NMR (500 MHz, CDCl_3_): *δ* 0.92 (*s*, *t*Bu), 2.02 (br *s*, NH), 2.86 (*s*, MeN), 2.97 (*dd*, *J* = 6.7; 14.2 Hz, 1H, CH_2_), 3.19 (*dd*, *J* = 4.2; 14.0 Hz, 1H, CH_2_), 3.76 (*d*, *J* = 1.9 Hz, H-C(2)), 3.90–3.96 (*m*, H-C(5)), 7.74 (*s*, 2 arom. H), 7.76 (*s*, 1 arom. H). ^13^C-NMR (75 MHz, CDCl_3_): *δ* 25.7, 31.4, 37.5, 39.0, 59.3, 83.9, 120.4–120.7 (*m*), 123.5 (*q*, *J* = 272.5 Hz), 129.2–130.1 (*m*), 131.5 (*q*, *J* = 33.1 Hz), 140.7, 174.6.

***cis*-6e**: elutes second from the column. Yield: 189 mg (0.494 mmol, 19%, 96% *ee*) of yellowish solid; m.p. 66–75 °C. [*α*]_D_^r.t.^ = –26.2 (c = 0.8 mg/mL, CH_2_Cl_2_). HPLC (Chiralcel OD-H, *n*-hexane/*i*-PrOH = 96:4, flow rate 1.0 mL/min, λ = 205 nm): *t*R = 14.1 min (major); 15.5 min (minor). EI-HRMS: *m/z* = 383.1541 (MH^+^); C_17_H_21_F_6_N_2_O requires *m/z* = 383.1553 (MH^+^). CHN microanalysis: C_17_H_20_F_6_N_2_O requires C, 53.40; H, 5.27; N, 7.33. Found: C, 53.35; H, 5.01; N, 7.42. *ν*_max_ 3346, 2960, 1682, 1622, 1485, 1398, 1380, 1341, 1279, 1175, 1136, 898, 842, 724, 707, 683 cm^−1^. ^1^H-NMR (300 MHz, CDCl_3_): *δ* 0.86 (*s*, *t*Bu), 1.90 (*s*, NH), 2.94 (*s*, MeN), 2.96 (*dd*, *J* = 8.0; 13.7 Hz, 1H, CH_2_), 3.25 (*dd*, *J* = 3.8; 13.7 Hz, 1H, CH_2_), 3.82 (*ddd*, *J* = 1.0; 3.8; 8.3 Hz, H-C(5)), 4.14 (*d*, *J* = 1.4 Hz, H-C(2)), 7.74 (*s*, 3 arom. H). ^13^C-NMR (75 MHz, CDCl_3_): *δ* 25.3, 30.8, 35.6, 39.3, 59.0, 82.3, 120.5–120.8 (*m*), 123.6 (*q*, *J* = 272.6 Hz), 130.1–130.3 (*m*), 131.7 (*q*, *J* = 33.1 Hz), 141.2, 173.7.

*Synthesis of imidazolidinones from α-amino amides* **4** *and fluoroacetone—General Procedure 7 (GP7)*

To a solution of α-amino amide **4** (1 equiv.) in anhydrous ethanol under argon, fluoroacetone was added. The reaction mixture was heated at reflux under argon using a Dean–Stark apparatus filled with freshly dried molecular sieves (4 Å MS) for 24 h. The volatiles were evaporated in vacuo, and the residue was purified by column chromatography (Silica gel 60) or flash column chromatography (Silica gel 60), followed by MPLC. The fractions containing the pure separated isomers ***trans*-8** and ***cis*-8** were combined, and the volatiles were evaporated in vacuo. The product was stored under argon at 5 °C. **Fluoroacetone must be handled with extreme care, as it is classified as Acute Toxicity Category 2. Acute Toxicity Category 2 is a global hazard classification for chemicals that are highly toxic or fatal in small amounts, indicating severe health risks, such as death or serious damage, from a single or short exposure via oral, dermal, or inhalation routes**.

*Synthesis of (2*R*,5*S*)-5-(3,5-bis(trifluoromethyl)benzyl)-2-(fluoromethyl)-2,3-dimethylimidazolidin-4-one (**trans*****-8b***) and (2*S*,5*S*)-5-(3,5-bis(trifluoromethyl)benzyl)-2-(fluoromethyl)-2,3-dimethylimidazolidin-4-one (**cis*****-8b***)*

Following *GP7*: Prepared from (*S*)-2-amino-3-(3,5-bis(trifluoromethyl)phenyl)-*N*-methylpropanamide (**4a**) (3.69 mmol, 1.16 g), EtOH (50 mL), and fluoroacetone (13.15 mmol, ω = 0.98, 968 μL); flash CC (EtOAc) to separate ***trans*-8b**/***cis*-8b** as a mixture from the rest of the impurities (***trans*-8b**:***cis*-8b** = 56:44), then MPLC (EtOAc/petroleum ether = 1:3).

***trans*-8b**: elutes first from the column. Yield: 398 mg (1.070 mmol, 29%, 99% *ee*) of yellowish oil. [*α*]_D_^r.t.^ = –32.8 (c = 2.3 mg/mL, CH_2_Cl_2_). HPLC (Chiralpak AD-H, *n*-hexane/*i*-PrOH = 98:2, flow rate 1.0 mL/min, λ = 205 nm): *t*R = 12.1 min (minor); 13.5 min (major). EI-HRMS: *m/z* = 373.1153 (MH^+^); C_15_H_16_F_7_N_2_O requires *m/z* = 373.1145 (MH^+^); *ν*_max_ 3327, 2942, 1682, 1623, 1538, 1470, 1434, 1404, 1383, 1343, 1280, 1171, 1126, 1038, 928, 900, 842, 725, 708, 683 cm^−1^. ^1^H-NMR (300 MHz, CDCl_3_): *δ* 1.13 (*d*, *J* = 2.6 Hz, Me), 1.90 (br *s*, NH), 2.80 (*s*, MeN), 3.01 (*dd*, *J* = 7.1; 14.2 Hz, 1H, CH_2_), 3.25 (*dd*, *J* = 4.2; 14.2 Hz, 1H, CH_2_), 3.98 (*dd*, *J* = 4.2; 7.0, Hz, H-C(5)), 4.18 (*dd*, *J* = 9.9; 39.7 Hz, 1H, CH_2_F), 4.33 (*dd*, *J* = 9.9, 40.1 Hz, 1H, CH_2_F), 7.73 (*s*, 2 arom. H), 7.76 (*s*, 1 arom. H). ^13^C-NMR (75 MHz, CDCl_3_): *δ* 22.1 (*d*, *J* = 2.5 Hz), 25.7 (*d*, *J* = 1.0 Hz), 38.8, 59.4 (*d*, *J* = 0.8 Hz), 76.5 (*d*, *J* = 18.3 Hz), 85.4 (*d*, *J* = 180.2 Hz), 120.7–121.0 (*m*), 123.5 (*q*, *J* = 272.6 Hz), 130.0–130.2 (*m*), 131.7 (*q*, *J* = 33.2 Hz), 140.4, 172.8.

***cis*-8b**: elutes second from the column. Yield: 288 mg (0.775 mmol, 21%, 97% *ee*) of yellowish oil. [*α*]_D_^r.t.^ = –55.7 (c = 1.1 mg/mL, CH_2_Cl_2_). HPLC (Chiralpak AD-H, *n*-hexane/*i*-PrOH = 98:2, flow rate 1.0 mL/min, λ = 205 nm): *t*R = 8.7 min (minor); 10.1 min (major). EI-HRMS: *m/z* = 373.1145 (MH^+^); C_15_H_16_F_7_N_2_O requires *m/z* = 373.1145 (MH^+^); *ν*_max_ 3332, 2983, 1682, 1623, 1470, 1434, 1404, 1383, 1345, 1277, 1171, 1132, 1013, 931, 898, 842, 726, 707, 683 cm^−1^. ^1^H-NMR (300 MHz, CDCl_3_): *δ* 1.38 (*d*, *J* = 2.8 Hz, Me), 1.95 (br *s*, NH), 2.85 (*s*, MeN), 2.93 (*dd*, *J* = 8.6; 13.9 Hz, 1H, CH_2_), 3.24 (*dd*, *J* = 3.7; 13.9 Hz, 1H, CH_2_), 3.91–3.97 (*m*, H-C(5)), 3.98 (*dd*, *J* = 9.9; 74.5 Hz, 1H, CH_2_F), 4.14 (*dd*, *J* = 9.9; 74.7 Hz, 1H, CH_2_F), 7.73 (*s*, 2 arom. H), 7.75 (*s*, 1 arom. H). ^13^C-NMR (75 MHz, CDCl_3_): *δ* 21.0 (*d*, *J* = 1.9 Hz), 25.7 (*d*, *J* = 2.3 Hz), 39.1, 59.1, 76.7 (*d*, *J* = 18.6 Hz), 86.3 (*d*, *J* = 180.9 Hz), 120.5–120.7 (*m*), 123.5 (*q*, *J* = 272.5 Hz), 130.0–130.2 (*m*), 131.5 (*q*, *J* = 33.1 Hz), 141.0, 172.6.

*Synthesis of (2*R*,5*S*)-2-(fluoromethyl)-2,3-dimethyl-5-(4-nitrobenzyl)imidazolidin-4-one (**trans-8c**) and (2*S*,5*S*)-2-(fluoromethyl)-2,3-dimethyl-5-(4-nitrobenzyl)imidazolidin-4-one (**cis-8c**)*

Following *GP7*: Prepared from (*S*)-2-amino-*N*-methyl-3-(4-nitrophenyl)propanamide (**4i**) (10.71 mmol, 2.391 g), EtOH (50 mL), and fluoroacetone (13.15 mmol, ω = 0.98, 968 μL); flash CC (EtOAc) to separate ***trans*-8c**/***cis*-8c** as a mixture from the rest of the impurities (***trans*-8c**:***cis*-8c** = 61:49), then MPLC (EtOAc).

***trans*-8c**: elutes first from the column. Yield: 904 mg (3.213 mmol, 30%, 99% *ee*) of yellowish oil. [*α*]_D_^r.t.^ = –63.1 (c = 2.4 mg/mL, CH_2_Cl_2_). HPLC (Chiralcel OD-H, *n*-hexane/*i*-PrOH = 70:30, flow rate 1.0 mL/min, λ = 205 nm): *t*R = 8.8 min (major); 11.7 min (minor). EI-HRMS: *m/z* = 282.1265 (MH^+^); C_13_H_17_FN_3_O_3_ requires *m/z* = 282.1248 (MH^+^); *ν*_max_ 3319, 2952, 1694, 1651, 1605, 1519, 1434, 1403, 1346, 1283, 1145, 1110, 1082, 1034, 854, 825, 747, 700 cm^−1^. ^1^H-NMR (300 MHz, CDCl_3_): *δ* 1.22 (*d*, *J* = 2.6 Hz, Me), 2.03 (br *s*, NH), 2.83 (*s*, MeN), 2.98 (*dd*, *J* = 7.6; 14.2 Hz, 1H, CH_2_), 3.27 (*dd*, *J* = 4.2; 14.2 Hz, 1H, CH_2_), 3.96 (*dd*, *J* = 4.2; 7.5 Hz, H-C(5)), 4.21 (*dd*, *J* = 9.8; 34.9 Hz, 1H, CH_2_F), 4.37 (*dd*, *J* = 9.8; 35.4 Hz, 1H, CH_2_F), 7.42–7.49 (*m*, 2 arom. H), 8.10–8.17 (*m*, 2 arom. H). ^13^C-NMR (75 MHz, CDCl_3_): *δ* 21.7 (*d*, *J* = 2.7 Hz), 25.6 (*d*, *J* = 1.1 Hz), 38.5, 59.3 (*d*, *J* = 1.2 Hz), 76.3 (*d*, *J* = 18.1 Hz), 85.5 (*d*, *J* = 179.7 Hz), 123.4, 130.4, 145.5, 146.8, 173.0.

***cis*-8c**: elutes second from the column. Yield: 603 mg (2.142 mmol, 20%, 99% *ee*) of yellowish oil. [*α*]_D_^r.t.^ = –86.0 (c = 0.9 mg/mL, CH_2_Cl_2_). HPLC (Chiralcel OD-H, *n*-hexane/*i*-PrOH = 80:20, flow rate 1.0 mL/min, λ = 205 nm): *t*R = 11.6 min (minor); 12.8 min (major). EI-HRMS: *m/z* = 282.1242 (MH^+^); C_13_H_17_FN_3_O_3_ requires *m/z* = 282.1248 (MH^+^); *ν*_max_ 3332, 2952, 1694, 1652, 1605, 1520, 1434, 1403, 1346, 1287, 1145, 1109, 1083, 1036, 1016, 863, 855, 823, 748, 700 cm^−1^. ^1^H-NMR (300 MHz, CDCl_3_): *δ* 1.36 (*d*, *J* = 2.7 Hz, Me), 2.08 (*s*, NH), 2.86 (*s*, MeN), 2.90 (*dd*, *J* = 8.8; 13.8 Hz, 1H, CH_2_), 3.25 (*dd*, *J* = 3.8; 13.8 Hz, 1H, CH_2_), 3.90–3.97 (*m*, H-C(5)), 4.02 (*dd*, *J* = 10.0; 61.4 Hz, 1H, CH_2_F), 4.18 (*dd*, *J* = 10.0; 61.6 Hz, 1H, CH_2_F), 7.39–7.46 (*m*, 2 arom. H), 8.11–8.19 (*m*, 2 arom. H). ^13^C-NMR (75 MHz, CDCl_3_): *δ* 20.9 (*d*, *J* = 1.9 Hz), 25.8 (*d*, *J* = 2.2 Hz), 39.2 (*d*, *J* = 0.9 Hz), 59.1, 76.6 (*d*, *J* = 18.6 Hz), 86.1 (*d*, *J* = 181.0 Hz), 123.5, 130.6, 146.3, 146.8, 172.6.

*Synthesis of (2*R*,5*S*)-2-(fluoromethyl)-5-(4-methoxybenzyl)-2,3-dimethylimidazolidin-4-one (**trans*****-8d***) and (2*S*,5*S*)-2-(fluoromethyl)-5-(4-methoxybenzyl)-2,3-dimethylimidazolidin-4-one (**cis*****-8d***)*

Following *GP7*: Prepared from (*S*)-2-amino-3-(4-methoxyphenyl)-*N*-methylpropanamide (**4h**) (10.71 mmol, 2.23 g), EtOH (50 mL), and fluoroacetone (13.15 mmol, ω = 0.98, 968 μL); flash CC (EtOAc) to separate ***trans*-8d**/***cis*-8d** as a mixture from the rest of the impurities (***trans*-8d**:***cis*-8d** = 60:40), then MPLC (EtOAc).

***trans*-8d**: elutes first from the column. Yield: 513 mg (1.928 mmol, 18%, 98% *ee*) of yellowish oil. [*α*]_D_^r.t.^ = –60.5 (c = 2.8 mg/mL, CH_2_Cl_2_). HPLC (Chiralcel OD-H, *n*-hexane/*i*-PrOH = 93:7, flow rate 1.0 mL/min, λ = 205 nm): *t*R = 18.5 min (major); 20.4 min (minor). EI-HRMS: *m/z* = 267.1511 (MH^+^); C_14_H_20_FN_2_O_2_ requires *m/z* = 267.1503 (MH^+^); *ν*_max_ 3456, 2955, 1682, 1613, 1583, 1514, 1434, 1403, 1249, 1180, 1146, 1111, 1080, 1032, 852, 817 cm^−1^. ^1^H-NMR (300 MHz, CDCl_3_): *δ* 1.10 (*d*, *J* = 2.4 Hz, Me), 1.74 (br *s*, NH), 2.81 (*s*, MeN), 3.03 (*d*, *J* = 5.3 Hz, CH_2_), 3.79 (*s*, MeO), 3.80 (*t*, *J* = 5.4 Hz, H-C(5)), 4.13 (*dd*, *J* = 9.6; 16.4 Hz, 1H, CH_2_F), 4.29 (*dd*, *J* = 9.6; 16.6 Hz, 1H, CH_2_F), 6.81–6.87 (*m*, 2 arom. H), 7.08–7.16 (*m*, 2 arom. H). ^13^C-NMR (75 MHz, CDCl_3_): *δ* 20.9 (*d*, *J* = 2.5 Hz), 25.5 (*d*, *J* = 2.0 Hz), 36.4, 54.9, 59.4 (*d*, *J* = 0.7 Hz), 75.8 (*d*, *J* = 18.3 Hz), 86.1 (*d*, *J* = 179.7 Hz), 113.8, 128.4, 130.3, 158.4, 173.7.

***cis*-8d**: elutes second from the column. Yield: 371 mg (1.392 mmol, 13%, 99% *ee*) of yellowish oil. [*α*]_D_^r.t.^ = –78.9 (c = 1.4 mg/mL, CH_2_Cl_2_). HPLC (Chiralpak AD-H, *n*-hexane/*i*-PrOH = 94:6, flow rate 1.0 mL/min, λ = 205 nm): *t*R = 21.7 min (major); 23.5 min (minor). EI-HRMS: *m/z* = 267.1510 (MH^+^); C_14_H_20_FN_2_O_2_ requires *m/z* = 267.1503 (MH^+^); *ν*_max_ 3341, 2965, 1682, 1614, 1514, 1470, 1434, 1403, 1300, 1249, 1180, 1111, 1082, 1035, 850, 823 cm^−1^. ^1^H-NMR (300 MHz, CDCl_3_): *δ* 1.32 (*d*, *J* = 2.8 Hz, Me), 2.09 (*s*, NH), 2.79 (*dd*, *J* = 8.3; 14.2 Hz, 1H, CH_2_), 2.84 (*s*, MeN), 3.10 (*dd*, *J* = 3.9; 14.0 Hz, 1H, CH_2_), 3.78 (*s*, MeO), 3.82–3.88 (*m*, H-C(5)), 3.98 (*dd*, *J* = 9.1; 56.7 Hz, 1H, CH_2_F), 4.14 (*dd*, *J* = 9.0; 56.6 Hz, 1H, CH_2_F), 6.80–6.87 (*m*, 2 arom. H), 7.12–7.18 (*m*, 2 arom. H). ^13^C-NMR (75 MHz, CDCl_3_): *δ* 20.8 (*d*, *J* = 1.6 Hz), 25.6 (*d*, *J* = 2.4 Hz), 37.9, 55.1, 59.4, 76.2 (*d*, *J* = 18.8 Hz), 86.0 (*d*, *J* = 180.5 Hz), 113.7, 129.7, 130.4, 158.3, 173.2.

*Synthesis of (2*R*,5*S*)-2-(fluoromethyl)-5-isopropyl-2,3-dimethylimidazolidin-4-one (**trans*****-8e***) and (2*S*,5*S*)-2-(fluoromethyl)-5-isopropyl-2,3-dimethylimidazolidin-4-one (**cis*****-8e***)*

Following *GP7*: Prepared from (*S*)-2-amino-*N*,3-dimethylbutanamide (**4j**) (18.518 mmol, 2.411 g), EtOH (80 mL), and fluoroacetone (22.222 mmol, ω = 0.98, 1.637 mL); column chromatography: EtOAc/petroleum ether = 1:5; ***trans*-8e**/***cis*-8e** = 62:38 (crude reaction mixture in CDCl_3_).

***trans*-8e**: elutes first from the column. Yield: 244.0 mg (1.296 mmol, 7%) of yellowish solid; m.p. = 56.5–72.9 °C. [*α*]_D_^r.t.^ = –11.3 (c = 0.95 mg/mL, CH_2_Cl_2_). EI-HRMS: *m/z* = 189.1399 (MH^+^); C_9_H_18_FN_2_O requires *m/z* = 189.1398 (MH^+^); *ν*_max_ 3338, 2961, 2840, 1681, 1427, 1403, 1382, 1348, 1296, 1148, 1098, 1025, 990, 896, 803, 763, 666, 610 cm^−1^. ^1^H-NMR (500 MHz, CDCl_3_): *δ* 0.89 (*d*, *J* = 6.8 Hz, 3H), 1.03 (*d*, *J* = 7.1 Hz, 3H), 1.41 (*d*, *J* = 2.4, 3H), 2.18 (*dt*, *J* = 3.5, 7.0 Hz, 1H), 2.85 (*d*, *J* = 0.8 Hz, 3H), 3.53 (*d*, *J* = 3.7 Hz, 1H), 4.23 (*dd*, *J* = 9.4, 38.7 Hz, 1H), 4.33 (*dd*, *J* = 9.6, 39.1 Hz, 1H), NH proton is missing. ^13^C-NMR (126 MHz, CDCl_3_): δ 16.63, 19.36, 21.69 (*d*, *J* = 2.3 Hz), 25.77 (*d*, *J* = 1.9 Hz), 29.50, 63.80, 75.85 (*d*, *J* = 18.3 Hz), 86.42 (*d*, *J* = 180.3 Hz), 174.21.

***cis*-8e**: elutes second from the column. Yield: 209.0 mg (1.111 mmol, 6%) of yellowish oil. [*α*]_D_^r.t.^ = –37.8 (c = 1.545 mg/mL, CH_2_Cl_2_). EI-HRMS: *m/z* = 189.4020 (MH^+^); C_9_H_18_FN_2_O requires *m/z* = 189.1398 (MH^+^); *ν*_max_ 3339, 2959, 1684, 1426, 1399, 1295, 1207, 1150, 1041, 1005, 907, 775, 641 cm^−1^. ^1^H-NMR (500 MHz, CDCl_3_): *δ* 0.88 (*d*, *J* = 6.7 Hz, 3H), 1.02 (*d*, *J* = 6.9 Hz, 3H), 1.37 (*d*, *J* = 2.8 Hz, 3H), 1.86 (br *s*, 1H), 2.13 (*td*, *J* = 4.1, 6.9 Hz, 1H), 2.85 (*d*, *J* = 0.9 Hz, 3H), 3.53 (*dd*, *J* = 2.2, 4.1 Hz, 1H), 4.23 (*dd*, *J* = 10.1, 25.9 Hz, 1H), 4.33 (*dd*, *J* = 10.0, 25.3 Hz, 1H). ^13^C-NMR (126 MHz, CDCl_3_): *δ* 16.80, 19.49, 21.05 (*d*, *J* = 1.7 Hz), 25.64 (*d*, *J* = 2.3 Hz), 29.94, 62.76, 75.82 (*d*, *J* = 19.1 Hz), 86.55 (*d*, *J* = 180.7 Hz), 173.37.

*Synthesis of (2*R*,5*S*)-2-(fluoromethyl)-5-isobutyl-2,3-dimethylimidazolidin-4-one (**trans*****-8f***) and (2*S*,5*S*)-2-(fluoromethyl)-5-isobutyl-2,3-dimethylimidazolidin-4-one (**cis*****-8f***)*

Following *GP7*: Prepared from (*S*)-2-amino-*N*,4-dimethylpentanamide (**4k**) (18.440 mmol, 2.660 g), EtOH (60 mL), and fluoroacetone (22.128 mmol, ω = 0.98, 1.630 mL); column chromatography: EtOAc/petroleum ether = 1:5; ***trans*-8f**/***cis*-8f** = 64:36 (crude reaction mixture in CDCl_3_).

***trans*-8f**: elutes first from the column. Yield: 895 mg (4.426 mmol, 24%) of yellowish solid; m.p. = 47–52 °C. [*α*]_D_^r.t.^ = +5.7 (c = 1.545 mg/mL, CH_2_Cl_2_). EI-HRMS: *m/z* = 203.1555 (MH^+^); C_10_H_20_FN_2_O requires *m/z* = 203.1554 (MH^+^); *ν*_max_ 3274, 2954, 2870, 1678, 1467, 1428, 1401, 1383, 1366, 1292, 1256, 1160, 1079, 995, 876, 843, 823, 758, 671, 652, 609 cm^−1^. ^1^H-NMR (500 MHz, CDCl_3_): *δ* 0.95 (*d*, *J* = 6.3 Hz, 3H), 0.97 (*d*, *J* = 6.4 Hz, 3H), 1.22–1.32 (*m*, 1H), 1.42 (*d*, *J* = 2.3 Hz, 3H), 1.64 (br *s*, 1H), 1.76–1.90 (*m*, 2H), 2.86 (*s*, 3H), 3.53 (*dd*, *J* = 3.1, 10.0 Hz, 1H), 4.23 (*dd*, *J* = 9.6, 22.6 Hz, 1H), 4.33 (*dd*, *J* = 9.6, 22.7 Hz, 1H). ^13^C-NMR (126 MHz, CDCl_3_): *δ* 21.68, 21.85 (*d*, *J* = 2.5 Hz), 23.47, 25.47, 26.07 (*d*, *J* = 2.2 Hz), 42.69, 57.27, 76.10 (*d*, *J* = 18.5 Hz), 86.35 (*d*, *J* = 180.2 Hz), 175.64.

***cis*-8f**: elutes second from the column. Yield: 224.8 mg (1.106 mmol, 6%) of yellowish solid; m.p. = 47–53 °C. [*α*]_D_^r.t.^ = +27.9 (c = 1.0 mg/mL, CH_2_Cl_2_). EI-HRMS: *m/z* = 203.1556 (MH^+^); C_10_H_20_FN_2_O requires *m/z* = 203.1554 (MH^+^); *ν*_max_ 3118, 2955, 2870, 1673, 1430, 1402, 1365, 1288, 1202, 1172, 1146, 1080, 1032, 999, 926, 893, 843, 830, 801, 762, 647, 630, 615 cm^−1^. ^1^H-NMR (500 MHz, CDCl_3_): *δ* 0.93 (*d*, *J* = 6.4 Hz, 3H), 0.96 (*d*, *J* = 6.5 Hz, 3H), 1.29–1.33 (*m*, 1H), 1.34 (*d*, *J* = 2.7 Hz, 3H), 1.72–1.89 (*m*, 2H), 1.96 (br *s*, 1H), 2.85 (*s*, 3H), 3.56–3.62 (*m*, 1H), 4.22 (*dd*, *J* = 10.1, 47.5 Hz, 1H), 4.33 (*dd*, *J* = 10.1, 47.6 Hz, 1H). ^13^C-NMR (126 MHz, CDCl_3_): *δ* 20.61 (*d*, *J* = 2.1 Hz), 21.57, 23.51, 25.75 (*d*, *J* = 2.1 Hz), 25.76, 42.41, 56.44, 76.60 (*d*, *J* = 18.5 Hz), 85.39 (*d*, *J* = 180.7 Hz), 175.04.

*Synthesis of (2*S*,5*S*)-5-(3,5-bis(trifluoromethyl)benzyl)-2,3-dimethyl-4-oxoimidazolidin-1-ium perchlorate (***9***)*

A solution of (2*S*,5*S*)-5-(3,5-bis(trifluoromethyl)benzyl)-2,3-dimethylimidazolidin-4-one (***cis*-6b**) (0.420 mmol, 143 mg) in Et_2_O (20 mL) was cooled to 0 °C. A mixture of HClO_4_ (0.441 mmol, 70% in H_2_O, 38 µL) in Et_2_O (5 mL) was then added. The product **9**, which precipitated from the reaction mixture, was collected by filtration and washed with anhydrous Et_2_O (2 × 5 mL). Yield: 159 mg (0.340 mmol, 81%) of white solid; m.p. = 169.9–172.0 °C. [*α*]_D_^r.t.^ = –60.0 (c = 1.20 mg/mL, CH_2_Cl_2_). EI-HRMS: *m/z* = 341.1082 (M^+^); C_14_H_15_F_6_N_2_O^+^ requires *m/z* = 341.1083 (M^+^). CHN microanalysis: C_14_H_15_ClF_6_N_2_O_5_ requires C, 38.15; H, 3.43; N, 6.36. Found: C, 38.30; H, 3.56; N, 6.25. *ν*_max_ 3009, 1702, 1491, 1424, 1385, 1345, 1283, 1173, 1124, 1101, 1041, 932, 905, 879, 859, 840, 790, 729, 706, 684, 654, 622 cm^−1^. ^1^H-NMR (500 MHz, CD_3_CN): *δ* 1.55 (*d*, *J* = 6.0 Hz, 3H), 2.82 (*s*, 3H), 3.22 (*dd*, *J* = 9.5, 15.2 Hz, 1H), 3.56 (*dd*, *J* = 5.1, 15.2 Hz, 1H), 4.31 (*dd*, *J* = 5.1, 9.6 Hz, 1H), 4.83 (*q*, *J* = 6.0 Hz, 1H), 7.97 (*s*, 3H) (2 signals missing). ^13^C-NMR (126 MHz, CD_3_CN): *δ* 17.52, 27.21, 34.65, 60.09, 70.38, 122.35–122.66 (*m*), 124.52 (*q*, *J* = 271.8 Hz), 131.13–131.44 (*m*), 132.18 (*q*, *J* = 33.2 Hz), 139.38, 167.33.

*Synthesis of (2*R*,5*S*,*E*)-5-(3,5-bis(trifluoromethyl)benzyl)-2,3-dimethyl-4-oxo-1-((*E*)-3-phenylallylidene)imidazolidin-1-ium perchlorate (***10***) and (2*R*,5*S*,*Z*)-5-(3,5-bis(trifluoromethyl)benzyl)-2,3-dimethyl-4-oxo-1-((*E*)-3-phenylallylidene)imidazolidin-1-ium perchlorate (***10′***)*

To a solution of (2*S*,5*S*)-5-(3,5-bis(trifluoromethyl)benzyl)-2,3-dimethyl-4-oxoimidazolidin-1-ium perchlorate (**9**) (0.229 mmol, 101 mg) in anhydrous EtOH (1 mL) under argon, *trans*-cinnamaldehyde (0.241 mmol, 30.3 μL) and anhydrous Et_3_N (1 μL) were added, and the reaction mixture was stirred at room temperature for 30 min. The product **10**/**10′**, which precipitated from the reaction mixture, was collected by filtration and washed with anhydrous Et_2_O (2 × 5 mL); **10**/**10′** = 61:39 (in CD_3_CN). Yield: 91 mg (0.165 mmol, 72%) of yellow solid; m.p. = 124.8–128.8 °C. [*α*]_D_^r.t.^ = –382.4 (c = 1.25 mg/mL, CH_2_Cl_2_). *ν*_max_ 3506, 2944, 1714, 1623, 1604, 1589, 1487, 1441, 1422, 1406, 1382, 1343, 1324, 1276, 1174, 1143, 1073, 999, 928, 907, 885, 844, 767, 724, 709, 682, 653, 622 cm^−1^. ^1^H-NMR (500 MHz, CD_3_CN) for compound **10**: *δ* 1.17 (*d*, *J* = 6.1 Hz, 3H), 2.82 (*s*, 3H), 3.56–3.61 (*m*, 1H), 3.65 (*dd*, *J* = 6.1, 14.8 Hz, 1H), 5.17 (*t*, *J* = 5.7 Hz, 1H), 5.44 (*q*, *J* = 6.1 Hz, 1H), 7.11 (*dd*, *J* = 10.9, 15.0 Hz, 1H), 7.54–8.03 (*m*, 8H), 8.17 (*d*, *J* = 15.0 Hz, 1H), 8.69 (*d*, *J* = 10.9 Hz, 1H). ^1^H-NMR (500 MHz, CD_3_CN) for compound **10′**: *δ* 1.30 (*d*, *J* = 6.0 Hz, 3H), 2.86 (*s*, 3H), 4.93 (*t*, *J* = 6.3 Hz, 1H), 5.73 (*q*, *J* = 6.1 Hz, 1H), 7.26 (*dd*, *J* = 11.0, 14.9 Hz, 2H), 8.15 (*d*, *J* = 15.0 Hz, 1H), 8.54 (*d*, *J* = 11.0 Hz, 1H). ^13^C-NMR (126 MHz, CD_3_CN) for compound **10**: *δ* 19.75, 27.24, 37.50, 64.14, 78.64, 117.68, 123.02–123.12 (*m*), 124.29 (*q*, *J* = 272.0 Hz), 130.63, 131.92–132.07 (*m*), 132.42 (*q*, *J* = 33.2 Hz), 132.50, 134.31, 136.27, 138.39, 165.45, 167.30, 170.29. ^13^C-NMR (126 MHz, CD_3_CN) for compound **10′**: *δ* 19.44, 27.27, 38.43, 66.81, 75.73, 116.90, 122.83–123.02 (*m*), 124.38 (*d*, *J* = 272.0 Hz), 130.70, 131.82–131.97 (*m*), 132.58, 134.33, 136.30, 138.36, 165.16, 167.26, 169.70 (1 signal missing).

*General procedure for the Michael addition of 1-methylindole to* trans*-cinnamaldehyde*

To a solution of catalyst (0.13 mmol unless otherwise stated) in a mixture of anhydrous CH_2_Cl_2_ (850 µL) and anhydrous *i*-PrOH (150 µL) under Ar, TFA (10 µL) was added, and the mixture was cooled to the desired temperature (T). The solution was stirred for 10 min before *trans*-cinnamaldehyde (1.5 mmol, 189 µL) was added. After stirring for an additional 20 min, 1-methylindole (97%, 0.50 mmol, 64 µL) was added in one portion. The reaction mixture was stirred at a constant temperature (T) for 48 h (unless otherwise stated). The reaction mixture was quickly transferred (especially if 1-methylindole was not completely consumed according to TLC analysis) into a suspension of NaBH_4_ (1.5 g) in EtOH (5 mL) and stirred at room temperature. After 1 h, the suspension was treated with saturated aqueous NaHCO_3_ (20 mL), and the mixture was extracted with Et_2_O (30 mL). The organic layer was separated, filtered through a short plug of silica gel, washed with Et_2_O, and the volatile components were evaporated in vacuo. The residue was analyzed by chiral HPLC: Chiralpak AD-H, *n*-hexane/*i*-PrOH = 90:10, flow rate 1.0 mL/min, λ = 205 nm, t_R_ = 10.7 min (*R*-isomer), t_R_ = 13.1 min (*S*-isomer). Absolute configuration was determined by comparing the relative retention time from the HPLC analysis with reported data [5].

*X-Ray Crystallography:* Single-crystal diffraction data were collected on a SuperNova dual-source diffractometer with an Atlas detector at room temperature using a mirror monochromator and CuKα radiation with *λ* = 1.54184 Å. The diffraction data were processed using CrysAlis Pro software (Version 1.171.36.28; Agilent Technologies: Yarnton) [41]. Structure was solved by direct methods, using Sir97 [42]. Full-matrix least-squares refinements on *F*^2^ were done with anisotropic displacement parameters for all non-hydrogen atoms—with the exception of fluorine atoms of one disordered CF_3_ group, which were refined with isotropic displacement parameters. H atoms were observed in a difference Fourier map. They were finally placed at expected calculated positions and treated as a riding model. Shelxl-2025/1 software [43] was used for structure refinement and interpretation. A drawing of the molecular structure (Figure 3) was produced using Ortep-III [44]. Structural and other crystallographic details on data collection and refinement have been deposited with the Cambridge Crystallographic Data Centre under the supplementary publication numbers CCDC 2562406. These data can be obtained free of charge via https://www.ccdc.cam.ac.uk/structures/ (accessed on 19 July 2026) (or from the CCDC, 12 Union Road, Cambridge CB2 1EZ, UK; fax: +44 1223 336033; e-mail: deposit@ccdc.cam.ac.uk).

## 4. Conclusions

All imidazolidinone organocatalysts **6a**–**i** and **8a**–**f** were prepared from (*S*)-configured α-amino acid *N*-methyl amides **4a**–**k**. Amino amides **4a**–**f** were synthesized in two steps from *tert*-butyl (*S*)-2-(*tert*-butyl)-3-methyl-4-oxoimidazolidine-1-carboxylate (**1a**) via alkylation with substituted benzyl halides **2a**–**f**, yielding the corresponding Boc-protected *trans*-2,5-disubstituted imidazolidinones **3a**–**f**, followed by acid-catalyzed hydrolysis to give amino amides **4a**–**f**. Substituted L-phenylalanine **4g**–**i**, L-valine **4j**, and L-leucine-derived **4k** amino-*N*-methylamide derivatives were prepared from the corresponding methyl ester ammonium salts **5a**–**e** by amidation with excess methylamine. Cyclization of amino amides **4a**–**e**,**g** with acetaldehyde, propionaldehyde, or pivalaldehyde produced mixtures of the corresponding *trans*- and *cis*-5-arylmethyl-2-alkyl-3-methylimidazolidin-4-ones, ***trans*-6a**–**i** and ***cis*-6a**–**i**. The isomer mixtures were separated by column chromatography. For mixtures that could not be separated by column chromatography, *N*-Boc protection was performed, followed by separation by column chromatography. Subsequent deprotection with trifluoroacetic acid and neutralization with NaHCO_3_ yielded the corresponding imidazolidinones. In this way, ***cis*-6a**–**i** and ***trans*-6b**–**e**,**g**–**i** were isolated. Similarly, treatment of (substituted) L-phenylalanine **4a**,**g**–**i**, L-valine **4j**, and L-leucine-derived **4k** amino-*N*-methylamide derivatives with fluoroacetone yielded mixtures of *trans*- and *cis*-5-substituted-2-(fluoromethyl)-2,3-dimethylimidazolidin-4-ones, ***trans*-8a**–**f** and ***cis*-8a**–**f**, which were separated by column chromatography. As a general rule, the *trans*-isomers, ***trans*-6** and ***trans*-8**, eluted first from the column, followed by the *cis*-isomers, ***cis*-6** and ***cis*-8**. The synthetic approach allows easy synthesis of imidazolidinones with diverse substitutions at C-2 and C-5.

Of the new catalysts tested (***trans*-6c**,**d**, ***cis*-6a**–**i**, ***trans*-8e**,**f**, ***cis*-8e**,**f**), the *trans*-2-alkyl-substituted catalysts ***trans*-6c**,**d** showed both incomplete conversion and very low enantioselectivity (up to 6% *ee*, (*R*)). In the *cis*-5-arylmethyl-2-alkyl-3-methylimidazolidin-4-one series ***cis*-6a**–**i**, (*S*)-enantioselectivities of up to 78% *ee* with full conversion were achieved. In the *cis*-5-arylmethyl-2-ethyl/2-methyl series of catalysts ***cis*-6a**–**d**,**f**–**i**, the highest enantioselectivity was achieved with catalyst ***cis*-6i**, which has a 2-methylnaphthalen-1-yl substituent at position 5 (69% *ee*, (*S*), at –42 °C, full conversion). Therefore, the combination of 2-*tert*-butyl and 5-(substituted)naphthyl substituents appears promising for future studies. In the 5-alkyl-2-(fluoromethyl)-2,3-dimethylimidazolidin-4-one series, the ***trans*-8e**,**f** catalysts gave the product with up to 70% *ee*, (*R*)-enantioselectivity, while the L-valine-derived *cis*-imidazolidinone organocatalyst ***cis*-8e** exhibited the highest reversal of stereoselectivity with full conversion to date (92% *ee*, (*R*) at –43 °C), surpassing the enantioselectivity achieved by the second-generation MacMillan organocatalyst ***cis*-II** (90% *ee*, (*S*)-enantioselectivity). The catalytic activity of the L-valine-derived organocatalyst ***cis*-8e** requires further mechanistic investigation and evaluation in additional organocatalyzed transformations catalyzed by imidazolidinone catalysts.

## Figures and Tables

**Table 1:** The key proton chemical shifts for the *cis* and *trans* isomers ^(a)^.

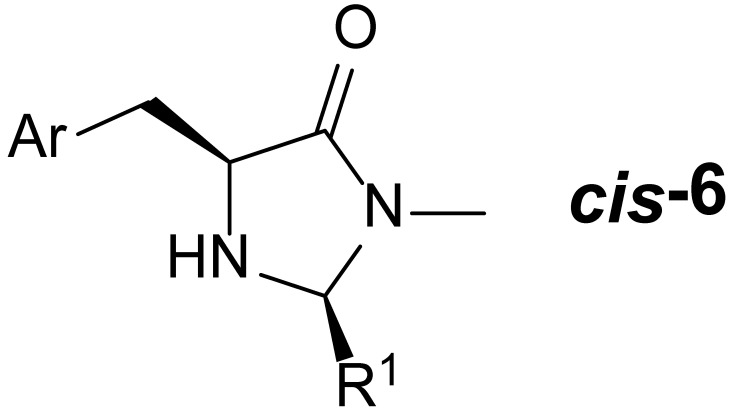			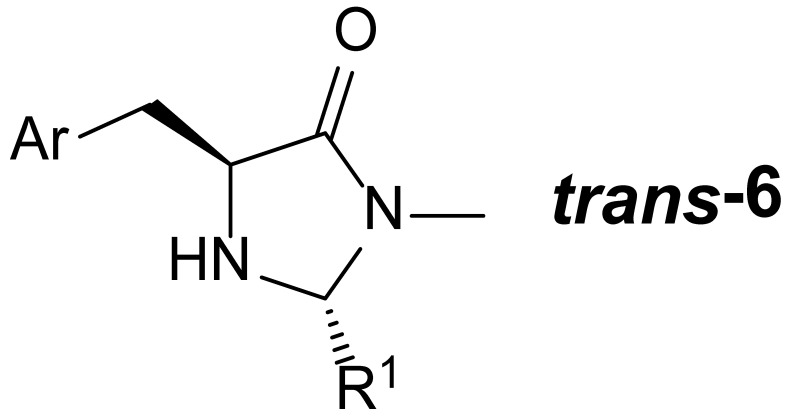		
Compound	H-C(5) Δ ^(c)^	H-C(2) Δ ^(c)^	Compound	H-C(5)	H-C(2)
***cis*** **-6a**	3.71; 0.11	4.35; 0.11	^(^^b)^		
***cis*-6b** [29]	3.73; 0.11	4.29; –0.25	***trans*-6b** [29]	3.84	4.04
***cis*** **-6c**	3.81; 0.09	4.47; –0.45	***trans*** **-6c**	3.90	4.02
***cis*** **-6d**	3.86; 0.03	4.37; –0.44	***trans*** **-6d**	3.89	3.93
***cis*-II** [29]	3.70; 0.14	4.05; –0.25	***trans*-II** [29]	3.84	3.80
***cis*** **-6e**	3.82; 0.11	4.14; –0.38	***trans*** **-6e**	3.93	3.76
***cis*** **-6f**	3.84; 0.00	4.38; 0.00	^(^^b)^		
***cis*** **-6g**	3.70; 0.11	4.30; –0.15	***trans*** **-6g**	3.81	4.15
***cis*** **-6h**	3.75; 0.13	4.28; –0.20	***trans*** **-6h**	3.88	4.08
***cis*** **-6i**	3.92; 0.03	4.26; 0.12	***trans*** **-6i**	3.95	4.38
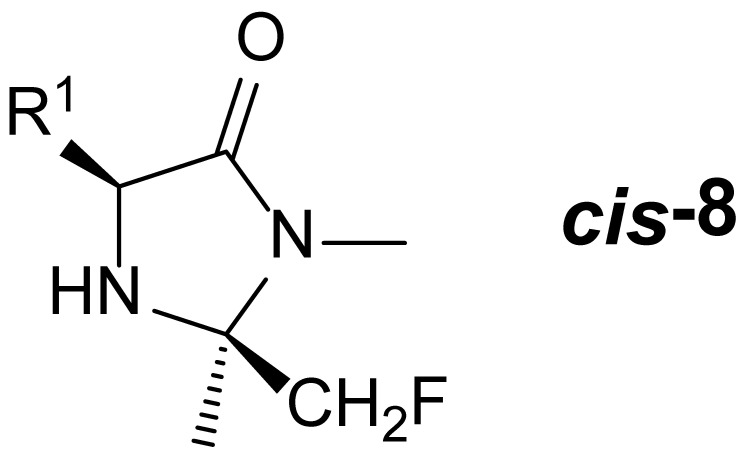			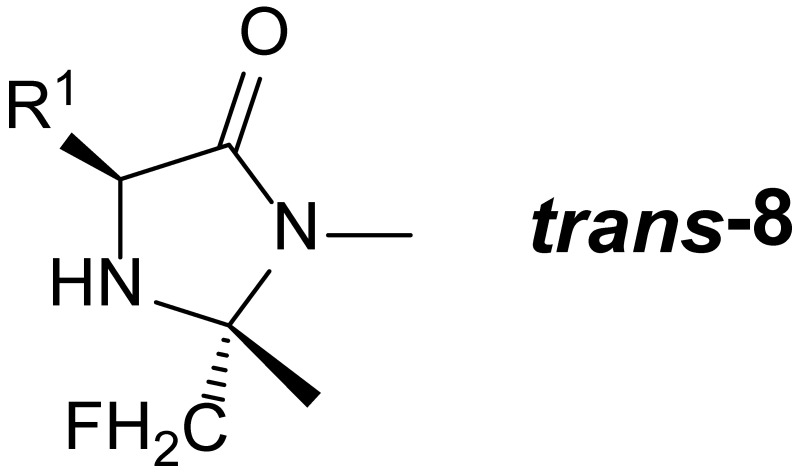		
**Compound**	**H_α_-CHF Δ** ^(c)^	**H_β_-CHF Δ** ^(c)^	**Compound**	**H_α_-CHF**	**H_β_-CHF**
***cis*-8a** [29]	3.95; 0.18	4.15; 0.14	***trans*-8a** [29]	4.13	4.29
***cis*** **-8b**	3.98; 0.20	4.14; 0.19	***trans*** **-8b**	4.18	4.33
***cis*** **-8c**	4.02; 0.19	4.18; 0.19	***trans*** **-8c**	4.21	4.37
***cis*** **-8d**	3.98; 0.15	4.14; 0.15	***rans*** **-8d**	4.13	4.29
***cis*** **-8e**	4.23; 0.00	4.33; 0.00	***trans*** **-8e**	4.23	4.33
***cis*** **-8f**	4.22; 0.01	4.33; 0.00	***trans*** **-8f**	4.23	4.33

^(a)^ Mean values of chemical shifts in ppm are presented without multiplicity and coupling constants; for details, see the Supplementary Materials. ^(b)^ Isomers were not synthesized. ^(c)^ Δ represents the difference in chemical shifts of the respective protons of the *cis*- and *trans*-isomer pairs, i.e., *trans*-H-C(5)—*cis*-H-C(5), *trans*-H-C(2)—*cis*-H-C(2), and *trans*-CH_2_F—*cis*-CH_2_F.

**Table 2:** Evaluation of organocatalysts in addition of 1-methyl-1*H*-indole to cinnamaldehyde.

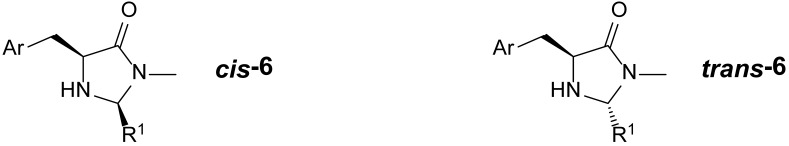					
Compound		R^1^, Ar	Temperature [°C]	Time [h]	*ee* [%] ^(a)^
1	***trans*** **-6c**	Me, 3,5-(F_3_C)_2_C_6_H_3_	–40 ^(b)^	72	6 (*R*)
2	***trans*** **-6d**	Et, 3,5-(F_3_C)_2_C_6_H_3_	–60 ^(b)^	45	4 (*R*)
3	***cis*** **-6a**	Me, Ph	–40	24	40 (*S*)
4	***cis*** **-6b**	Et, Ph	–40 ^(b)^	72	51 (*S*)
5	***cis*** **-6c**	Me, 3,5-(F_3_C)_2_C_6_H_3_	0 ^(b)^	72	27 (*S*)
6	***cis*** **-6c**	Me, 3,5-(F_3_C)_2_C_6_H_3_	–40 ^(b)^	72	35 (*S*)
7	***cis*** **-6d**	Et, 3,5-(F_3_C)_2_C_6_H_3_	–60	45	36 (*S*)
8	***cis*** **-II**	*t*Bu, Ph	25	2	72 (*S*)
9	***cis*** **-II**	*t*Bu, Ph	–41	48	87 (*S*)
10	***cis*** **-II**	*t*Bu, Ph	–61 ^(b)^	48	90 (*S*)
11	***cis*** **-6e**	*t*Bu, 3,5-(F_3_C)_2_C_6_H_3_	25	2	70 (*S*)
12	***cis*** **-6e**	*t*Bu, 3,5-(F_3_C)_2_C_6_H_3_	–41	48	78 (*S*)
13	***cis*** **-6f**	Et, 2,5-(F_3_C)_2_C_6_H_3_	–40	48	55 (*S*)
14	***cis*** **-6g**	Et, 3,5-(MeO)_2_C_6_H_3_	–42 ^(b)^	96	51 (*S*)
15	***cis*** **-6h**	Et, 3-PhC_6_H_4_	–40 ^(b)^	72	47 (*S*)
16	***cis*** **-6i**	Et, 2-methylnaphthalen-1-yl	–42	96	69 (*S*)
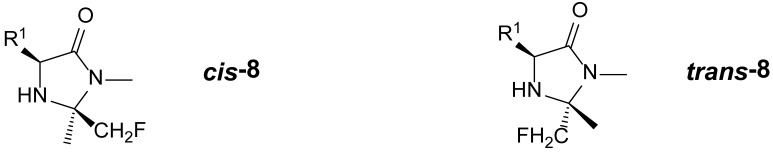					
**Compound**		**R^1^**	**Temperature [°C]**	**Time [h]**	***ee*** **[%] ^(a)^**
17	**I**	Bn, 2,2-di-Me	–41 ^(b)^	48	44 (*S*)
18	**III** [35]	*i*Pr, 2,2-di-Me	–55	-	71 (*R*)
19	***trans*-8b** ^(c)^ [34]	3,5-(F_3_C)_2_C_6_H_3_-CH_2_	–41	48	40 (*S*)
20	***trans*** **-8e**	*i*Pr	–43	46	70 (*R*)
21	***trans*** **-8f**	*i*Bu	–43 ^(b)^	45	58 (*R*)
22	***cis*-8b** ^(c)^ [34]	3,5-(F_3_C)_2_C_6_H_3_-CH_2_	–41	48	78 (*R*)
23	***cis*** **-8e**	*i*Pr	25	1.5	67 (*R*)
24	***cis*** **-8e**	*i*Pr	–43	46	92 (*R*)
25	***cis*** **-8e**	*i*Pr	–63 ^(b)^	48	93 (*R*)
26	***cis*** **-8f**	*i*Bu	–43 ^(b)^	45	85 (*R*)

If not stated otherwise, 20 mol% catalyst was used. ^(a)^ Enantiomer excess (*ee*) determined by HPLC on Chiralpak AD-H, with hexane/*i*PrOH 90:10; ^(b)^ partial conversion at this temperature; ^(c)^ 26 mol% catalyst used.

## Data Availability

The original contributions presented in this study are included in the article/Supplementary Materials. Further inquiries can be directed to the corresponding author.

## Supplementary Materials

The following supporting information can be downloaded at https://www.mdpi.com/article/10.3390/molecules31152684/s1.

## References

[1] Seebach D., Sting A.R., Hoffmann M. (1996). Self-Regeneration of Stereocenters (SRS)—Applications, Limitations, and Abandonment of a Synthetic Principle. Angew. Chem. Int. Ed. Eng..

[2] Ahrendt K.A., Borths C.J., MacMillan D.W.C. (2000). New Strategies for Organic Catalysis:  The First Highly Enantioselective Organocatalytic Diels−Alder Reaction. J. Am. Chem. Soc..

[3] Paras N.A., MacMillan D.W.C. (2001). New Strategies in Organic Catalysis:  The First Enantioselective Organocatalytic Friedel−Crafts Alkylation. J. Am. Chem. Soc..

[4] Hechavarria Fonseca M.T., List B. (2004). Catalytic Asymmetric Intramolecular Michael Reaction of Aldehydes. Angew. Chem. Int. Ed..

[5] Austin J.F., MacMillan D.W.C. (2002). Enantioselective Organocatalytic Indole Alkylations. Design of a New and Highly Effective Chiral Amine for Iminium Catalysis. J. Am. Chem. Soc..

[6] Jen W.S., Wiener J.J.M., MacMillan D.W.C. (2000). New Strategies for Organic Catalysis:  The First Enantioselective Organocatalytic 1,3-Dipolar Cycloaddition. J. Am. Chem. Soc..

[7] Beeson T.D., MacMillan D.W.C. (2005). Enantioselective Organocatalytic α-Fluorination of Aldehydes. J. Am. Chem. Soc..

[8] MacMillan D.W.C. (2008). The advent and development of organocatalysis. Nature.

[9] Cozzi P.G., Gualandi A., Mengozzi L., Wilson C.M., North M., North M. (2015). Imidazolidinones as Asymmetric Organocatalysts. Sustainable Catalysis: Without Metals or Other Endangered Elements, Part 2.

[10] Dalko P.I. (2013). Comprehensive Enantioselective Organocatalysis: Catalysts, Reactions, and Applications.

[11] Mukherjee S., Yang J.W., Hoffmann S., List B. (2007). Asymmetric Enamine Catalysis. Chem. Rev..

[12] Dalko P.I. (2026). Enantioselective Organocatalysis: Catalysts, Reactions, and Applications.

[13] Michrowska A., List B. (2009). Concise synthesis of ricciocarpin A and discovery of a more potent analogue. Nat. Chem..

[14] Austin J.F., Kim S.-G., Sinz C.J., Xiao W.-J., MacMillan D.W.C. (2004). Enantioselective organocatalytic construction of pyrroloindolines by a cascade addition–cyclization strategy: Synthesis of (–)-flustramine B. Proc. Natl. Acad. Sci. USA.

[15] Laforteza B.N., Pickworth M., MacMillan D.W.C. (2013). Enantioselective Total Synthesis of (−)-Minovincine in Nine Chemical Steps: An Approach to Ketone Activation in Cascade Catalysis. Angew. Chem. Int. Ed..

[16] Reiter M., Torssell S., Lee S., MacMillan D.W.C. (2010). The organocatalytic three-step total synthesis of (+)-frondosin B. Chem. Sci..

[17] Veselý J., Kamlar M. (2026). Solid-supported chiral organocatalysts: Methods, platforms, and applications in asymmetric synthesis. Catal. Today.

[18] Deepa, Singh S. (2021). Recent Development of Recoverable MacMillan Catalyst in Asymmetric Organic Transformations. Adv. Synth. Catal..

[19] Raimondi L., Faverio C., Boselli M.F. (2020). Chiral imidazolidinones: A class of priviliged organocatalysts in stereoselective organic synthesis. Phys. Sci. Rev..

[20] Nicewicz D.A., MacMillan D.W.C. (2008). Merging Photoredox Catalysis with Organocatalysis: The Direct Asymmetric Alkylation of Aldehydes. Science.

[21] Li M., Sang Y., Xue X.-S., Cheng J.-P. (2018). Origin of Stereocontrol in Photoredox Organocatalysis of Asymmetric α-Functionalizations of Aldehydes. J. Org. Chem..

[22] Nielsen C.D.T., Linfoot J.D., Williams A.F., Spivey A.C. (2022). Recent progress in asymmetric synergistic catalysis—The judicious combination of selected chiral aminocatalysts with achiral metal catalysts. Org. Biomol. Chem..

[23] Beeson T.D., Mastracchio A., Hong J.-B., Ashton K., MacMillan D.W.C. (2007). Enantioselective Organocatalysis Using SOMO Activation. Science.

[24] Capacci A.G., Malinowski J.T., McAlpine N.J., Kuhne J., MacMillan D.W.C. (2017). Direct, enantioselective α-alkylation of aldehydes using simple olefins. Nat. Chem..

[25] Allen A.E., MacMillan D.W.C. (2012). Synergistic catalysis: A powerful synthetic strategy for new reaction development. Chem. Sci..

[26] Grošelj U., Schweizer W.B., Ebert M.-O., Seebach D. (2009). 5-Benzyl-3-methylimidazolidin-4-one-Derived Reactive Intermediates of Organocatalysis—A Comforting Resemblance of X-Ray, NMR, and DFT Solid-Phase, Liquid-Phase, and Gas-Phase Structures. Helv. Chim. Acta.

[27] Seebach D., Grošelj U., Schweizer W.B., Grimme S., Mück-Lichtenfeld C. (2010). Experimental and Theoretical Conformational Analysis of 5-Benzylimidazolidin-4-one Derivatives—A ‘Playground’ for Studying Dispersion Interactions and a ‘Windshield-Wiper’ Effect in Organocatalysis. Helv. Chim. Acta.

[28] Seebach D., Grošelj U., Badine D.M., Schweizer W.B., Beck A.K. (2008). Isolation and X-Ray Structures of Reactive Intermediates of Organocatalysis with Diphenylprolinol Ethers and with Imidazolidinones. Helv. Chim. Acta.

[29] Grošelj U., Beck A., Schweizer W.B., Seebach D. (2014). Preparation and Structures of 2-Substituted 5-Benzyl-3-methylimidazolidin-4-one-Derived Iminium Salts, Reactive Intermediates in Organocatalytic Transformations Involving α,β-Unsaturated Aldehydes. Helv. Chim. Acta.

[30] Seebach D., Gilmour R., Grošelj U., Deniau G., Sparr C., Ebert M.-O., Beck A.K., McCusker L.B., Šišak D., Uchimaru T. (2010). Stereochemical Models for Discussing Additions to α,β-Unsaturated Aldehydes Organocatalyzed by Diarylprolinol or Imidazolidinone Derivatives—Is There an ‘*(E)/(Z)-Dilemma*’?. Helv. Chim. Acta.

[31] Sparr C., Gilmour R. (2010). Fluoro-Organocatalysts: Conformer Equivalents as a Tool for Mechanistic Studies. Angew. Chem. Int. Ed..

[32] Aufiero M., Gilmour R. (2018). Informing Molecular Design by Stereoelectronic Theory: The Fluorine Gauche Effect in Catalysis. Acc. Chem. Res..

[33] Holland M.C., Metternich J.B., Mück-Lichtenfeld C., Gilmour R. (2015). Cation–π interactions in iminium ion activation: Correlating quadrupole moment & enantioselectivity. Chem. Commun..

[34] Grošelj U., Podlipnik Č., Bezenšek J., Svete J., Stanovnik B., Seebach D. (2013). Reversal of the Stereochemical Course of 1-Methyl-1*H*-indole Addition to Cinnamaldehyde with cis-5-Benzyl-(2-fluoromethyl)-2,3-dimethylimidazolidin-4-ones as Catalysts—A Puzzling ‘Fluorine Effect’. Helv. Chim. Acta.

[35] Holland M.C., Metternich J.B., Daniliuc C., Schweizer W.B., Gilmour R. (2015). Aromatic Interactions in Organocatalyst Design: Augmenting Selectivity Reversal in Iminium Ion Activation. Chem. Eur. J..

[36] Paras N.A., MacMillan D.W.C. (2002). The Enantioselective Organocatalytic 1,4-Addition of Electron-Rich Benzenes to α,β-Unsaturated Aldehydes. J. Am. Chem. Soc..

[37] Kortylewicz Z.P., Galardy R.E. (1990). Phosphoramidate peptide inhibitors of human skin fibroblast collagenase. J. Med. Chem..

[38] Bulman Page P.C., Buckley B.R., Rassias G.A., Blacker A.J. (2006). New Chiral Iminium Salt Catalysts for Asymmetric Epoxidation. Eur. J. Org. Chem..

[39] Amer M.M., Carrasco A.C., Leonard D.J., Ward J.W., Clayden J. (2018). Substituted Dihydroisoquinolinones by Iodide-Promoted Cyclocarbonylation of Aromatic α-Amino Acids. Org. Lett..

[40] Leonard D.J., Ward J.W., Clayden J. (2018). Asymmetric α-arylation of amino acids. Nature.

[41] Agilent Technologies (2013). CrysAlis PRO, Version 1.171.36.28.

[42] Altomare A., Burla M.C., Camalli M., Cascarano G.L., Giacovazzo C., Guagliardi A., Moliterni A.G.G., Polidori G., Spagna R. (1999). SIR97: A new tool for crystal structure determination and refinement. J. Appl. Crystallogr..

[43] Sheldrick G.M. (2015). Crystal structure refinement with SHELXL. Acta Crystallogr. Sect. C Struct. Chem..

[44] Farrugia L.J. (1997). ORTEP-3 for Windows—A version of ORTEP-III with a Graphical User Interface (GUI). J. Appl. Crystallogr..

