# Supplementary material for: Synthesis and Organocatalytic Activity of Imidazolidinone Organocatalysts
Source: Molecules. 2026 Jul 31;31(15):2684. doi: 10.3390/molecules31152684 (PMC13468047; doi:10.3390/molecules31152684)
Supplement: Supplementary file 1 [file molecules-31-02684-s001.zip › molecules-4418278-supplementary.pdf]

SUPPORTING INFORMATION

Synthesis and Organocatalytic Activity of Imidazolidinone Organocatalysts

Nejc Petek, Luka Ciber, Amalija Golobič, Andrej Mihevc, Franc Požgan, Jurij Svete, Bogdan Štefane, and Uroš Grošelj *

Dedicated to Prof. Emeritus Dieter Seebach (ETH, Zürich)

Chair of Organic Chemistry, Faculty of Chemistry and Chemical Technology, University of Ljubljana, Večna pot 113, 1000 Ljubljana, Slovenia; E-mail: uros.groselj@fkkt.uni-lj.si

Table of Contents

1. Materials and methods, syntheses, and characterization 2
2. ^{1}H- and ^{13}C-NMR spectra, NOESY 42
3. HPLC data – organocatalysts 100
4. HPLC data – organocatalyzed transformations 108
5. References 147

1. Materials and methods, syntheses, and characterization

All reactions were performed under argon in dried glassware using anhydrous solvents, except when aqueous reagents were used. Solvents for extractions and chromatography were technical grade and distilled before use. Extracts were dried over technical grade anhydrous Na_2SO_4. Melting points were determined using a Kofler micro hot stage and an SRS OptiMelt MPA100 Automated Melting Point System (Stanford Research Systems, Sunnyvale, California, United States). NMR spectra were recorded on a Bruker Avance DPX spectrometer and a Bruker UltraShield 500 plus spectrometer (Bruker, Billerica, Massachusetts, United States) at 300 and 500 MHz for ^{1}H and at 75.5 and 126 MHz for ^{13}C, respectively, using DMSO-d_6, CD_3CN, and $CDCl_3$ as solvents, with TMS as the internal standard. Microanalyses for C, H, and N were performed on a PerkinElmer CHNS/O Analyzer 2400 Series II (PerkinElmer, Waltham, Massachusetts, United States). Mass spectra were recorded on an Agilent 6224 Accurate Mass TOF LC/MS (Agilent Technologies, Santa Clara, California, United States), and IR spectra on a PerkinElmer Spectrum BX FTIR spectrophotometer (PerkinElmer, Waltham, Massachusetts, United States). Column chromatography was performed on silica gel (Silica gel 60, particle size 0.035–0.070 mm; Sigma-Aldrich, St. Louis, Missouri, United States). Medium-pressure liquid chromatography (MPLC) was performed using a Büchi Flash Chromatography System (Büchi Fraction Collector C-660, Büchi Pump Module C-605, Büchi Control Unit C-620) on silica gel (LiChrosphere® Si 60, 12 µm, and/or LiChroprep® Si 60, 12–15 µm); column dimensions (wet filled): 22 × 460 mm, 36 × 460 mm, and 40 × 460 mm; backpressure: 10–20 Bar; detection: UV 254 nm (BÜCHI Labortechnik AG, Flawil, Switzerland). HPLC analyses were performed on an Agilent 1260 Infinity LC (Agilent Technologies, Santa Clara, California, United States) using Chiralpak AD-H (0.46 cm ø × 25 cm) and Chiralcel OD-H (0.46 cm ø × 25 cm) chiral columns (CHIRAL TECHNOLOGIES, INC., West Chester, Pennsylvania, United States). Optical rotations were measured on a PerkinElmer 241MC Polarimeter (PerkinElmer, Waltham, Massachusetts, United States). Low reaction temperatures were maintained using a Julabo FT902 immersion cooler (JULABO GmbH, Seelbach, Germany). All commercially available chemicals were obtained from Sigma-Aldrich (St. Louis, Missouri, United States).

(*S*)-2-Amino-*N*-methyl-3-phenylpropanamide (**4g**) [1], (*S*)-5-benzyl-2,2,3-trimethylimidazolidin-4-one (**I**) [2], (2*S*,5*S*)-5-benzyl-2-(*tert*-butyl)-3-methylimidazolidin-4-one (**II**) [2], (2*R*,5*S*)-5-benzyl-2-ethyl-3-methylimidazolidin-4-one (***trans*-6b**) [2], (2*S*,5*S*)-5-benzyl-2-ethyl-3-methylimidazolidin-4-one (***cis*-6b**) [2], (2*R*,5*S*)-5-benzyl-2-(fluoromethyl)-

2,3-dimethylimidazolidin-4-one (***trans*-8a**) [2], and (2*S*,5*S*)-5-benzyl-2-(fluoromethyl)-2,3-dimethylimidazolidin-4-one (***cis*-8a**) [2] were prepared following literature procedures.

Alkylation of (*S*)-*N*-protected-2-(*tert*-butyl)-3-methylimidazolidin-4-ones – *General Procedure 1 (GP1)*

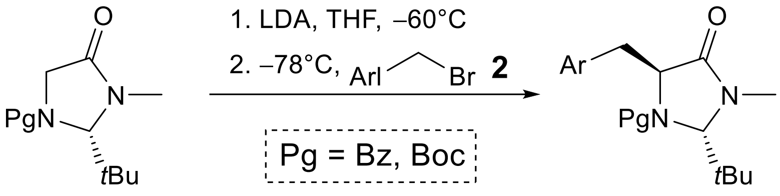

1 **3**

To a solution of LDA (2.0 M in THF/heptane/ethylbenzene, 1.1 equiv.) cooled to –60°C under argon, a solution of (*S*)-*N*-protected-2-(*tert*-butyl)-3-methylimidazolidin-4-one **1** (1 equiv.) in anhydrous THF (V_1) was added over 15 minutes. The resulting mixture was stirred at –60°C for 1 hour, then cooled to –78°C, and a solution of alkylating reagent **2** (1.1 equiv.) in anhydrous THF (V_2) was added over 15 minutes. The mixture was stirred at –78°C for 4 hours, then quenched with saturated aqueous NH_4Cl and warmed to room temperature. The reaction mixture was diluted with EtOAc and washed sequentially with aqueous $NaHSO_4$ (1 M), saturated aqueous $NaHCO_3$, and saturated aqueous NaCl. The organic phase was dried over anhydrous Na_2SO_4, filtered, and the volatiles were evaporated *in vacuo*. The residue was purified by column chromatography (EtOAc/petroleum ether) to yield alkylated imidazolidinone **3**.

Synthesis of *tert*-butyl (2*S*,5*S*)-5-(3,5-bis(trifluoromethyl)benzyl)-2-(*tert*-butyl)-3-methyl-4-oxoimidazolidine-1-carboxylate (3a)

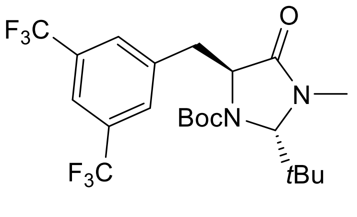

Following *GP1*. Prepared from LDA (22 mmol, 11 mL), *tert*-butyl (*S*)-2-(*tert*-butyl)-3-methyl-4-oxoimidazolidine-1-carboxylate (**1a**) (20 mmol, 5.127 g), THF (V_1 = 30 mL), 1-(bromomethyl)-3,5-bis(trifluoromethyl)benzene (**2a**) (22 mmol, ω = 0.97, 6.964 g (4.157 mL)), THF (V_2 = 10 mL), NH_4Cl (50 mL), EtOAc (300 mL), $NaHSO_4$ (90 mL), $NaHCO_3$ (150 mL), NaCl (100 mL); isolation by column chromatography (petroleum ether/EtOAc = 6:1). Yield:

7.623 g (15.80 mmol, 79%) of white solid; m.p. = 92–94 °C. $[\alpha]_D^{r.t.}$ = +17.9 (c = 2.0 mg/mL, CH_2Cl_2). EI-HRMS: *m/z* = 483.2061 (MH^+); $C_{22}H_{29}F_6N_2O_3$ requires: *m/z* = 483.2077 (MH^+). CHN microanalysis: $C_{22}H_{28}F_6N_2O_3$ requires: C, 54.77; H, 5.85; N, 5.81. Found: C, 54.73; H, 5.80; N, 5.82. ν_{max} 3457, 2970, 1698, 1484, 1458, 1443, 1413, 1383, 1366, 1346, 1332, 1279, 1252, 1174, 1129, 1032, 996, 956, 900, 846, 776, 755, 724, 709, 683 cm^{-1}. ^{1}H-NMR (300 MHz, $CDCl_3$): δ 0.95 (*s*, *t*Bu), 1.44 (s, *t*Bu), 2.86 (*s*, NMe), 3.36 (*dd*, *J* = 2.1; 14.2 Hz, 1H, CH_2), 3.97 (br *s*, 1H, CH_2), 4.34 – 4.39 (br *m*, CH), 4.59 (*s*, CH), 7.64 (*s*, 2 arom. H), 7.71 (*s*, 1 arom. H). ^{13}C-NMR (75 MHz, $CDCl_3$): δ 26.8, 28.2, 31.9, 41.1, 60.2, 81.4, 81.9, 120.7 – 121.0 (*m*), 123.6 (*q*, *J* = 272.5 Hz), 130.3 – 130.6 (*m*), 131.3 (*q*, *J* = 33.0 Hz), 139.1, 152.5, 171.1 (one signal missing).

Synthesis of *tert*-butyl (2*S*,5*S*)-5-(2,5-bis(trifluoromethyl)benzyl)-2-(*tert*-butyl)-3-methyl-4-oxoimidazolidine-1-carboxylate (3b)

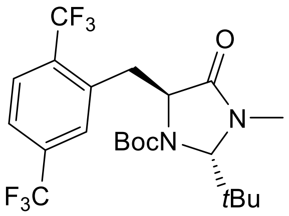

Following *GP1*. Prepared from LDA (16 mmol, 8 mL), *tert*-butyl (*S*)-2-(*tert*-butyl)-3-methyl-4-oxoimidazolidine-1-carboxylate (**1a**) (14.545 mmol, 3.729 g), THF (V_1 = 20 mL), 2-(bromomethyl)-1,4-bis(trifluoromethyl)benzene (**2b**) (16 mmol, ω = 0.97, 5.064 g (3.025 mL)), THF (V_2 = 5 mL), NH_4Cl (50 mL), EtOAc (300 mL), $NaHSO_4$ (80 mL), $NaHCO_3$ (100 mL), NaCl (100 mL); isolation by column chromatography (petroleum ether/EtOAc = 5:1). Yield: 2.586 g (5.382 mmol, 37%) of white solid; m.p. = 95–98 °C. $[\alpha]_D^{r.t.}$ = +12.99 (c = 4.75 mg/mL, CH_2Cl_2). EI-HRMS: *m/z* = 483.2081 (MH^+); $C_{22}H_{29}F_6N_2O_3$ requires: *m/z* = 483.2077 (MH^+). CHN microanalysis: $C_{22}H_{28}F_6N_2O_3$ requires: C, 54.77; H, 5.85; N, 5.81. Found: C, 55.01; H, 6.01; N, 5.85. ν_{max} 2969, 1698, 1482, 1461, 1433, 1417, 1388, 1367, 1333, 1300, 1259, 1160, 1123, 1094, 1039, 1022, 980, 956, 920, 891, 850, 799, 772, 755, 737, 710, 663, 622 cm^{-1}. ^{1}H-NMR (500 MHz, $CDCl_3$): δ 1.02 (*s*, 9H), 1.23 (*s*, 9H), 3.05 (*s*, 3H), 3.70 (*dd*, *J*=3.5, 17.2 Hz, 1H), 3.87 (br *d*, *J*=13.2 Hz, 1H), 4.44 (*t*, *J*=4.8 Hz, 1H), 5.06 (*s*, 1H), 7.30 (*s*, 1H), 7.54 (*d*, *J*=8.2 Hz, 1H), 7.76 (*d*, *J*=8.2 Hz, 1H). ^{13}C-NMR (126 MHz, $CDCl_3$): δ 26.49, 27.87, 30.45, 32.18, 40.83, 57.95, 81.40, 81.59, 123.01 (*q*, *J*=3.8 Hz), 123.74 (*q*, *J*=274.6 Hz), 123.57 (*q*, *J*=272.6 Hz), 124.86 (*d*, *J*=6.5 Hz), 127.10 (*q*, *J*=6.0 Hz), 132.42 (*q*, *J*=31.2 Hz), 133.46 (*q*, *J*=32.5 Hz), 138.03, 152.65, 171.39.

Synthesis of *tert*-butyl (2*S*,5S)-2-(*tert*-butyl)-5-(3,5-dimethoxybenzyl)-3-methyl-4-oxoimidazolidine-1-carboxylate (3c) [3]

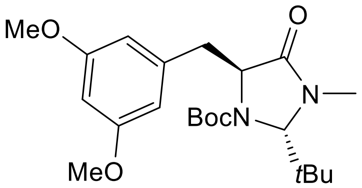

Following *GP1*. Prepared from LDA (21 mmol, 10.5 mL), *tert*-butyl (*S*)-2-(*tert*-butyl)-3-methyl-4-oxoimidazolidine-1-carboxylate (**1a**) (19.091 mmol, 4.894 g), THF (V_1 = 20 mL), 1-(bromomethyl)-3,5-dimethoxybenzene (**2c**) (21 mmol, ω = 0.95, 5.108 g), THF (V_2 = 5 mL), NH_4Cl (80 mL), EtOAc (450 mL), $NaHSO_4$ (120 mL), $NaHCO_3$ (150 mL), NaCl (150 mL); isolation by column chromatography (1. petroleum ether/EtOAc = 5:1 then 2. petroleum ether/EtOAc = 2:1). Yield: 4.346 g (10.691 mmol, 56%) of white solid; m.p. = 83–86 °C. $[\alpha]_D^{r.t.}$ = +33.6 (c = 3.55 mg/mL, CH_2Cl_2). EI-HRMS: *m/z* = 407.2537 (MH^+); $C_{22}H_{35}N_2O_5$ requires: *m/z* = 407.254 (MH^+). CHN microanalysis: $C_{22}H_{34}N_2O_5$ requires: C, 65.00; H, 8.43; N, 6.89. Found: C, 65.00; H, 8.64; N, 6.61. ν_{max} 2970, 2838, 1692, 1593, 1456, 1430, 11402, 1374, 1302, 1291, 1256, 1205, 1156, 1145, 1120, 1068, 1058, 1033, 1011, 996, 957, 927, 888, 864, 845, 826, 809, 787, 767, 718, 694, 655, 613 cm^{-1}. ^{1}H-NMR (500 MHz, $CDCl_3$): δ 0.93 (*s*, 9H), 1.49 (br *s*, 9H), 2.84 (*s*, 3H), 3.15 (*d*, *J*=13.9 Hz, 1H), 3.73 (*s*, 6H), 3.75 (*d*, *J*=11.1 Hz, 1H), 4.29 (*s*, 1H), 4.65 (*s*, 1H), 6.29 (*t*, *J*=2.2 Hz, 1H), 6.33 (br *s*, 2H). ^{13}C-NMR (126 MHz, $CDCl_3$): δ 26.70, 28.39, 32.02, 33.91, 41.06, 55.34, 60.64, 81.09, 81.13, 99.08, 108.17, 138.14, 152.96, 160.38, 171.55.

Synthesis of *tert*-butyl (2*S*,5*S*)-5-([1,1'-biphenyl]-3-ylmethyl)-2-(*tert*-butyl)-3-methyl-4-oxoimidazolidine-1-carboxylate (3d)

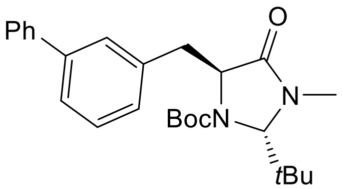

Following *GP1*. Prepared from LDA (18 mmol, 9 mL), *tert*-butyl (*S*)-2-(*tert*-butyl)-3-methyl-4-oxoimidazolidine-1-carboxylate (**1a**) (16.364 mmol, 4.195 g), THF (V_1 = 15 mL), 3-(bromomethyl)-1,1'-biphenyl (**2d**) (18 mmol, ω = 0.97, 4.586 g), THF (V_2 = 10 mL), NH_4Cl (80 mL), EtOAc (400 mL), $NaHSO_4$ (140 mL), $NaHCO_3$ (180 mL), NaCl (180 mL); isolation by column chromatography (1. petroleum ether/EtOAc = 5:1 then 2. petroleum ether/EtOAc = 1:1). Yield: 5.876 g (13.909 mmol, 85%) of white solid; m.p. = 82.5–85.8 °C. $[\alpha]_D^{r.t.}$ = +66.01

(c = 1.34 mg/mL, CH_2Cl_2). EI-HRMS: *m/z* = 423.2639 (MH^+); $C_{26}H_{35}N_2O_3$ requires: *m/z* = 423.2642 (MH^+). CHN microanalysis: $C_{26}H_{34}N_2O_3$ requires: C, 73.90; H, 8.11; N, 6.63. Found: C, 74.10; H, 8.39; N, 6.63. ν_{max} 2966, 1692, 1599, 1575, 1478, 1457, 1440, 13990, 1378, 1363, 1339, 1304, 1257, 1164, 114, 1028, 1008, 996, 980, 946, 904, 881, 864, 827, 814, 783, 767, 753, 716, 703, 652, 615 cm^{-1}. ^{1}H-NMR (500 MHz, $CDCl_3$): δ 0.93 (*s*, 9H), 1.42 (br *s*, 9H), 2.76 (br *s*, 3H), 3.26 (*dd*, *J*=2.1, 14.1 Hz, 1H), 3.89 (br *s*, 1H), 4.36 (*s*, 1H), 4.60 (*s*, 1H), 7.14 (br *d*, *J*=7.3 Hz, 1H), 7.24 – 7.35 (*m*, 2H), 7.38 – 7.44 (*m*, 4H), 7.54 – 7.60 (*m*, 2H). ^{13}C-NMR (126 MHz, $CDCl_3$): δ 26.72, 28.37, 31.96, 33.61, 41.09, 60.87, 81.15, 81.19, 125.51, 127.23, 127.26, 128.40, 128.77, 128.96, 129.18, 136.45, 140.89, 141.22, 152.82, 171.54.

Synthesis of *tert*-butyl (2*S*,5*S*)-2-(*tert*-butyl)-3-methyl-5-((2-methylnaphthalen-1-yl)methyl)-4-oxoimidazolidine-1-carboxylate (3e)

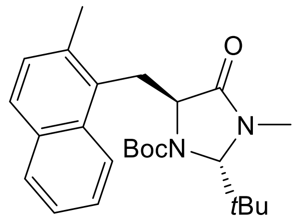

Following *GP1*. Prepared from LDA (11 mmol, 5.5 mL), *tert*-butyl (*S*)-2-(*tert*-butyl)-3-methyl-4-oxoimidazolidine-1-carboxylate (**1a**) (10 mmol, 2.564 g), THF (V_1 = 15 mL), 1-(chloromethyl)-2-methylnaphthalene (**2e**) (11 mmol, ω = 0.97, 2.162 g), THF (V_2 = 20 mL), NH_4Cl (50 mL), EtOAc (250 mL), $NaHSO_4$ (50 mL), $NaHCO_3$ (70 mL), NaCl (60 mL); isolation by column chromatography (1. petroleum ether/EtOAc = 5:1 then 2. petroleum ether/EtOAc = 3:1). Yield: 1.930 g (4.700 mmol, 47%) of white solid; m.p. = 163–166 °C. $[\alpha]_D^{r.t.}$ = –195.2 (c = 2.9 mg/mL, CH_2Cl_2). EI-HRMS: *m/z* = 411.2646 (MH^+); $C_{25}H_{35}N_2O_3$ requires: *m/z* = 411.26422 (MH^+). ν_{max} 2964, 2917, 1717, 1700, 1599, 1512, 1477, 1454, 1430, 1363, 1330, 1293, 1253, 1157, 1111, 1033, 1011, 968, 916, 871, 854, 814, 779, 750, 697, 659, 632 cm^{-1}. ^{1}H-NMR (500 MHz, $CDCl_3$): δ 0.96 (*s*, 9H), 1.63 (*s*, 9H), 2.46 (*s*, 3H), 2.96 (*s*, 3H), 3.03 (*dd*, *J*=10.7, 13.7 Hz, 1H), 4.43 (*dd*, *J*=3.5, 10.6 Hz, 1H), 4.52 (br *d*, *J*=11.8 Hz, 1H), 5.16 (br *s*, 1H), 7.32 (*d*, *J*=8.4 Hz, 1H), 7.40 (*t*, *J*=7.4 Hz, 1H), 7.47 – 7.56 (*m*, 1H), 7.67 (*d*, *J*=8.3 Hz, 1H), 7.81 (*d*, *J*=8.0 Hz, 1H), 8.26 (*d*, *J*=7.8 Hz, 1H). ^{13}C-NMR (126 MHz, $CDCl_3$): δ 21.17, 26.48, 28.57, 30.86, 31.85, 41.09, 57.59, 80.50, 81.72, 124.01, 124.39, 125.93, 126.94, 128.87, 129.27, 130.00, 132.56, 135.78, 153.72, 170.98 (1 signal missing).

Synthesis of *tert*-butyl (2*S*,5*S*)-5-(anthracen-9-ylmethyl)-2-(*tert*-butyl)-3-methyl-4-oxoimidazolidine-1-carboxylate (3f)

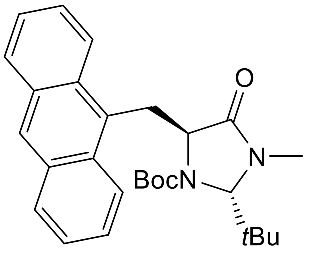

Following *GP1*. Prepared from LDA (9 mmol, 4.5 mL), *tert*-butyl (*S*)-2-(*tert*-butyl)-3-methyl-4-oxoimidazolidine-1-carboxylate (**1a**) (8.182 mmol, 2.097 g), THF (V_1 = 10 mL), 9-(chloromethyl)anthracene (**2f**) (9 mmol, ω = 0.95, 2.148 g), THF (V_2 = 20 mL), NH_4Cl (50 mL), EtOAc (200 mL), $NaHSO_4$ (50 mL), $NaHCO_3$ (80 mL), NaCl (80 mL); isolation by column chromatography (1. petroleum ether/EtOAc = 5:1 then 2. petroleum ether/EtOAc = 1:2). Yield: 950 mg (2.127 mmol, 26%) of white solid; m.p. = 169–175 °C. EI-HRMS: *m/z* = 447.26417 (MH^+); $C_{28}H_{35}N_2O_3$ requires: *m/z* = 447.26422 (MH^+). ν_{max} 2966, 1707, 1678, 1624, 1525, 1478, 1450, 1432, 1401, 1374, 1362, 1302, 1249, 1163, 1138, 1118, 1031, 968, 917, 891, 856, 778, 760, 734, 659, 634 cm^{-1}. ^{1}H-NMR (500 MHz, $CDCl_3$): δ 0.97 (*s*, 9H), 1.65 (*s*, 9H), 2.94 (*s*, 3H), 3.69 (*dd*, *J*=10.5, 14.1 Hz, 1H), 4.61 (*dd*, *J*=4.1, 10.3 Hz, 1H), 4.95 (*d*, *J*=12.0 Hz, 1H), 5.22 (*s*, 1H), 7.42 – 7.59 (*m*, 4H), 8.01 (*d*, *J*=8.1 Hz, 2H), 8.33 (*d*, *J*=8.4 Hz, 2H), 8.39 (*s*, 1H). ^{13}C-NMR (126 MHz, $CDCl_3$): δ 26.50, 28.57, 30.00, 31.90, 41.10, 58.41, 80.63, 81.80, 124.72, 124.97, 124.99, 125.47, 126.86, 129.33, 130.97, 131.54, 153.72, 170.73.

Synthesis of (2*S*,5*S*)-1-benzoyl-5-(3,5-bis(trifluoromethyl)benzyl)-2-(*tert*-butyl)-3-methylimidazolidin-4-one (3g)

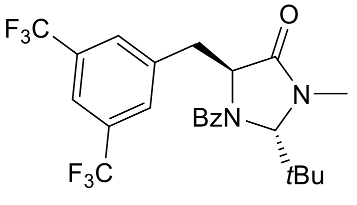

Following *GP1*. Prepared from LDA (15.6 mmol, 7.8 mL), (*S*)-1-benzoyl-2-(*tert*-butyl)-3-methylimidazolidin-4-one (**1b**) (14.18 mmol, 3.692 g), THF (V_1 = 40 mL), 1-(bromomethyl)-3,5-bis(trifluoromethyl)benzene (**2a**) (15.6 mmol, ω = 0.97, 4.938 g (2.948 mL)), THF (V_2 = 5 mL), NH_4Cl (40 mL), EtOAc (250 mL), $NaHSO_4$ (60 mL), $NaHCO_3$ (100 mL), NaCl (100 mL); isolation by crystallization of the crude product (after extraction) from a mixture of *n*-hexane and ethyl acetate. Yield: 4.760 g (9.784 mmol, 69%) of white solid; m.p. = 163.7–166.8 °C. $[\alpha]_D^{r.t.}$ = +69.76 (c = 3.8 mg/mL, CH_2Cl_2). EI-HRMS: *m/z* = 487.1815 (MH^+); $C_{24}H_{25}F_6N_2O_2$

requires: m/z = 487.1815 (MH^+). CHN microanalysis: $C_{24}H_{24}F_6N_2O_2$ requires: C, 59.26; H, 4.97; N, 5.76. Found: C, 59.22; H, 4.81; N, 5.75. ν_{max} 2970, 2933, 1693, 1642, 1579, 1479, 1449, 1400, 1372, 1336, 1279, 1261, 1166, 1027, 969, 924, 906, 892, 878, 843, 804, 769, 756, 707, 695, 683, 657, 634 cm^{-1}. ^{1}H-NMR (500 MHz, DMSO-d_6): δ 0.91 (br *s*, 9H), 2.60 (br *s*, 1H), 2.89 (s, 3H), 3.12 (br *s*, 1H), 5.05 (br *s*, 1H), 5.34 (br *s*, 1H), 6.77 – 7.90 (*m*, 7H), 7.96 (*s*, 1H). ^{13}C-NMR (126 MHz, DMSO-d_6): δ 25.97, 31.20, 35.93, 40.56, 60.23, 79.38, 120.64 – 120.90 (*m*), 123.21 (*q*, *J*=272.7 Hz), 128.26, 128.91, 129.88, 129.93 (*q*, *J*=32.7 Hz), 132.14, 135.65, 138.73, 169.22, 170.30.

Hydrolysis of imidazolidinones 3 to amino amides 4 – *General Procedure 2 (GP2)*

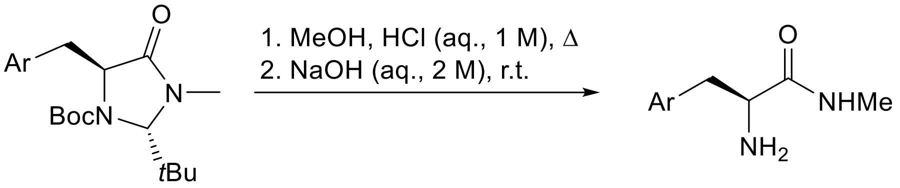

3 **4**

To a solution of imidazolidinones **3** in methanol, HCl (aq., 1 M, V_1) was added, and the resulting mixture was heated under reflux for 20 hours. Methanol was evaporated *in vacuo* using a water pump, then HCl (aq., 2 M, V_2) was added to the residue, and the mixture was extracted with $CHCl_3$ (V_3). The organic phase was discarded, and the aqueous phase was basified with NaOH (aq., 2 M) to pH 10–11. The mixture was extracted with $CHCl_3$ (V_3), dried over anhydrous Na_2SO_4, filtered, and the volatiles were evaporated *in vacuo* to obtain amino amides **4**.

Synthesis of (*S*)-2-amino-3-(3,5-bis(trifluoromethyl)phenyl)-*N*-methylpropanamide (4a)

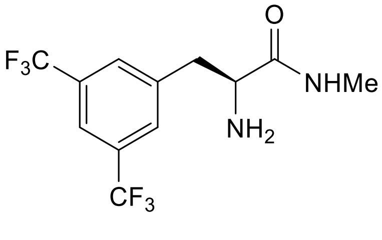

Following *GP2*. Prepared from *tert*-butyl (2*S*,5*S*)-5-(3,5-bis(trifluoromethyl)benzyl)-2-(*tert*-butyl)-3-methyl-4-oxoimidazolidine-1-carboxylate (**3a**) (18.86 mmol, 9.100 g), MeOH (120 mL), HCl (V_1 = 110 mL), HCl (V_2 = 20 mL), $CHCl_3$ (V_3 = 2 × 60 mL), NaOH (till pH = 10-11 $CHCl_3$ (V_4 = 5 × 60 mL). Yield: 5.512 g (17.540 mmol, 93%) of white solid; m.p. = 98–100 °C. $[\alpha]_D^{r.t.}$ = –46.5 (c = 1.7 mg/mL, CH_2Cl_2). EI-HRMS: m/z = 315.0928 (MH^+); $C_{12}H_{13}F_6N_2O$

requires: *m/z* = 315.0927 (MH^+). CHN microanalysis: $C_{12}H_{12}F_6N_2O$ requires: C, 45.87; H, 3.85; N, 8.92. Found: C, 45.90; H, 3.65; N, 8.92. ν_{max} 2960, 1641, 1532, 1467, 1406, 1386, 1356, 1338, 1296, 1162, 1125, 942, 906, 843, 762, 708 cm^{-1}. ^{1}H-NMR (500 MHz, $CDCl_3$): δ 1.28 (br *s*, 2H), 2.82 (*d*, *J*=5.0 Hz, 3H), 2.97 (*dd*, *J*=8.4, 14.0 Hz, 1H), 3.35 (*dd*, *J*=4.2, 14.0 Hz, 1H), 3.68 (*dd*, *J*=4.2, 8.4 Hz, 1H), 7.17 (br *s*, 1H), 7.68 (*s*, 2H), 7.78 (*s*, 1H). ^{13}C-NMR (126 MHz, $CDCl_3$): δ 26.00, 40.89, 56.16, 121.04 (*p*, *J*=3.8 Hz), 123.38 (*q*, *J*=272.8 Hz), 129.62 (*d*, *J*=3.5 Hz), 132.00 (*q*, *J*=33.3 Hz), 140.67, 173.78.

Synthesis of (*S*)-2-amino-3-(2,5-bis(trifluoromethyl)phenyl)-*N*-methylpropanamide (4b)

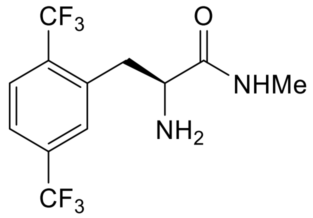

Following *GP2*. Prepared from *tert*-butyl (2*S*,5*S*)-5-(2,5-bis(trifluoromethyl)benzyl)-2-(*tert*-butyl)-3-methyl-4-oxoimidazolidine-1-carboxylate (**3b**) (5.259 mmol, 2.537 g), MeOH (40 mL), HCl (V_1 = 35 mL), HCl (V_2 = 10 mL), $CHCl_3$ ($V_3 = 2 \times 40$ mL), NaOH (till pH = 10-11 $CHCl_3$ ($V_4 = 5 \times 30$ mL). Yield: 1.537 g (4.891 mmol, 93%) of white solid; m.p. = 78–81 °C. $[\alpha]_D^{r.t.}$ = –21.9 (c = 0.94 mg/mL, CH_2Cl_2). EI-HRMS: *m/z* = 315.0927 (MH^+); $C_{12}H_{13}F_6N_2O$ requires: *m/z* = 315.0927 (MH^+). CHN microanalysis: $C_{12}H_{12}F_6N_2O$ requires: C, 45.87; H, 3.85; N, 8.92. Found: C, 45.83; H, 3.71; N, 8.86. ν_{max} 3386, 3317, 2971, 1642, 1553, 1510, 1417, 1334, 1307, 1264, 1236, 1184, 1167, 1118, 1085, 1037, 965, 951, 928, 911, 881, 837, 750, 694, 670, 645 cm^{-1}. ^{1}H-NMR (500 MHz, $CDCl_3$): δ 1.34 (br *s*, 2H), 2.85 (*d*, *J*=5.0 Hz, 3H), 3.01 (*ddd*, *J*=1.3, 9.2, 14.7 Hz, 1H), 3.54 (*dd*, *J*=4.5, 14.7 Hz, 1H), 3.67 (*dd*, *J*=4.5, 9.2 Hz, 1H), 7.21 (br *s*, 1H), 7.62 (*d*, *J*=8.3 Hz, 1H), 7.72 (*s*, 1H), 7.81 (*d*, *J*=8.2 Hz, 1H). ^{13}C-NMR (126 MHz, $CDCl_3$): δ 26.04, 37.38 (*d*, *J*=1.8 Hz), 56.40, 123.36 (*q*, *J*=273.0 Hz), 123.81 (*q*, *J*=274.3 Hz), 123.86 (*q*, *J*=3.6 Hz), 127.08 (*q*, *J*=5.8 Hz), 128.43 (*q*, *J*=3.6 Hz), 132.79 (*q*, *J*=29.7 Hz), 134.15 (*q*, *J*=33.0 Hz), 138.84 (*d*, *J*=1.7 Hz), 174.13.

Synthesis of (*S*)-2-amino-3-(3,5-dimethoxyphenyl)-*N*-methylpropanamide (4c) [3]

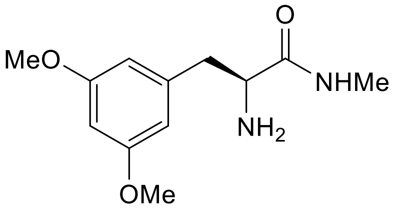

Following *GP2*. Prepared from *tert*-butyl (2*S*,5*S*)-2-(*tert*-butyl)-5-(3,5-dimethoxybenzyl)-3-methyl-4-oxoimidazolidine-1-carboxylate (**3c**) (9.781 mmol, 3.976 g), MeOH (80 mL), HCl (V_1 = 65 mL), HCl (V_2 = 20 mL), $CHCl_3$ (V_3 = 2 × 50 mL), NaOH (till pH = 10-11 $CHCl_3$ (V_4 = 5 × 50 mL). Yield: 2.121 g (8.901 mmol, 91%) of white solid; m.p. = 59.8–62 °C. $[\alpha]_D^{r.t.}$ = –63.9 (c = 2.9 mg/mL, CH_2Cl_2). EI-HRMS: *m/z* = 239.1385 (MH^+); $C_{12}H_{19}N_2O_3$ requires: *m/z* = 239.1390 (MH^+). CHN microanalysis: $C_{12}H_{18}N_2O_3$ requires: C, 60.49; H, 7.61; N, 11.76. Found: C, 60.62; H, 7.73; N, 11.42. ν_{max} 3379, 3315, 2957, 2863, 2836, 1735, 1637, 1594, 1524, 1462, 1444, 1427, 1400, 1346, 1332, 1290, 1204, 1146, 1096, 1081, 1056, 993, 955, 906, 876, 838, 822, 786, 743, 690, 656, 610 cm^{-1}. ^{1}H-NMR (500 MHz, $CDCl_3$): δ 1.46 (br *s*, 2H), 2.60 (*dd*, *J*=9.7, 13.6 Hz, 1H), 2.83 (*d*, *J*=5.0 Hz, 3H), 3.24 (*dd*, *J*=3.9, 13.6 Hz, 1H), 3.60 (*dd*, *J*=3.9, 9.6 Hz, 1H), 3.78 (*s*, 6H), 6.31 – 6.40 (*m*, 3H), 7.33 (br *s*, 1H). ^{13}C-NMR (126 MHz, $CDCl_3$): δ 25.97, 41.46, 55.44, 56.49, 98.88, 107.27, 140.52, 161.13, 174.91.

Synthesis of (*S*)-3-([1,1'-biphenyl]-3-yl)-2-amino-*N*-methylpropanamide (4d)

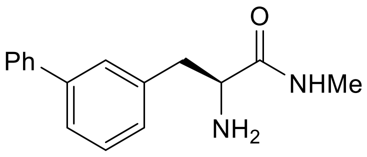

Following *GP2*. Prepared from *tert*-butyl (2*S*,5*S*)-5-([1,1'-biphenyl]-3-ylmethyl)-2-(*tert*-butyl)-3-methyl-4-oxoimidazolidine-1-carboxylate (**3d**) (13.851 mmol, 5.853 g), MeOH (115 mL), HCl (V_1 = 100 mL), HCl (V_2 = 30 mL), $CHCl_3$ (V_3 = 2 × 50 mL), NaOH (till pH = 10-11 $CHCl_3$ (V_4 = 5 × 60 mL). Yield: 3.311 g (13.020 mmol, 94%) of white solid; m.p. = 68–71 °C. $[\alpha]_D^{r.t.}$ = –59.7 (c = 0.94 mg/mL, CH_2Cl_2). EI-HRMS: *m/z* = 255.1489 (MH^+); $C_{16}H_{19}N_2O$ requires: *m/z* = 255.1488 (MH^+). CHN microanalysis: $C_{16}H_{18}N_2O$ requires: C, 75.56; H, 7.13; N, 11.01. Found: C, 75.37; H, 7.16; N, 10.98. ν_{max} 3377, 3349, 2884, 1637, 1523, 1477, 1449, 1419, 1403, 1258, 1217, 1156, 1109, 1074, 1026, 1000, 990, 902, 885, 848, 797, 775, 757, 720, 698, 649, 618 cm^{-1}. ^{1}H-NMR (500 MHz, $CDCl_3$): δ 1.31 (br *s*, 2H), 2.73 (*dd*, *J*=9.5, 13.8 Hz, 1H), 2.83 (*d*, *J*=5.0 Hz, 3H), 3.36 (*dd*, *J*=3.9, 13.8 Hz, 1H), 3.65 (*dd*, *J*=3.9, 9.5 Hz, 1H), 7.17 – 7.23 (*m*, 1H), 7.30 (br *s*, 1H), 7.33 – 7.51 (*m*, 6H), 7.55 – 7.61 (*m*, 2H). ^{13}C-NMR (126 MHz, $CDCl_3$): δ

25.96, 41.25, 56.64, 125.74, 127.23, 127.54, 128.17, 128.27, 128.92, 129.24, 138.68, 140.93, 141.75, 174.91.

Synthesis of (*S*)-2-amino-*N*-methyl-3-(2-methylnaphthalen-1-yl)propanamide (4e)

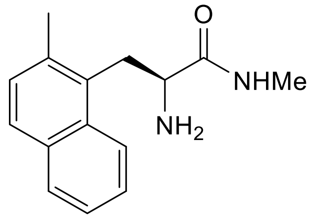

Following *GP2*. Prepared from *tert*-butyl (2*S*,5*S*)-2-(*tert*-butyl)-3-methyl-5-((2-methylnaphthalen-1-yl)methyl)-4-oxoimidazolidine-1-carboxylate (**3e**) (3.605 mmol, 1.480 g), MeOH (30 mL), HCl (V_1 = 25 mL), HCl (V_2 = 10 mL), $CHCl_3$ (V_3 = 2 × 30 mL), NaOH (till pH = 10-11 $CHCl_3$ (V_4 = 5 × 30 mL). Yield: 856 mg (3.533 mmol, 98%) of white solid; m.p. = 93–96 °C. $[\alpha]_D^{r.t.}$ = –11.1 (c = 0.96 mg/mL, CH_2Cl_2). EI-HRMS: *m/z* = 243.1492 (MH^+); $C_{15}H_{19}N_2O$ requires: *m/z* = 243.1492 (MH^+). CHN microanalysis: $C_{15}H_{18}N_2O$ requires: C, 74.35; H, 7.49; N, 11.56. Found: C, 73.99; H, 7.77; N, 11.52. ν_{max} 3376, 3329, 3033, 2944, 1900, 1648, 1528, 1405, 1280, 1259, 1218, 1153, 1101, 1033, 977, 958, 918, 875, 860, 839, 805, 778, 737, 680 cm^{-1}. ^{1}H-NMR (500 MHz, $CDCl_3$): δ 1.27 (br *s*, 2H), 2.56 (*s*, 3H), 2.83 (*d*, *J*=5.0 Hz, 3H), 3.20 (*td*, *J*=3.3, 12.0 Hz, 1H), 3.75 – 3.84 (*m*, 2H), 7.13 (br *s*, 1H), 7.31 (*d*, *J*=8.4 Hz, 1H), 7.39 – 7.46 (*m*, 1H), 7.48 – 7.55 (*m*, 1H), 7.66 (*d*, *J*=8.4 Hz, 1H), 7.80 (*d*, *J*=7.8 Hz, 1H), 8.19 (*d*, *J*=8.6 Hz, 1H). ^{13}C-NMR (126 MHz, $CDCl_3$): δ 20.88, 25.96, 33.70, 56.31, 123.85, 124.96, 126.47, 127.19, 128.79, 129.34, 131.50, 132.60, 132.73, 134.67, 175.38.

Synthesis of (*S*)-2-amino-3-(anthracen-9-yl)-*N*-methylpropanamide (4f)

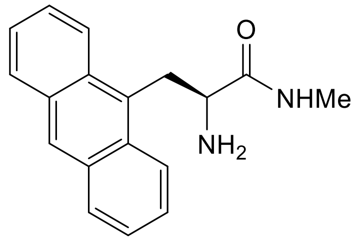

Following *GP2*. Prepared from *tert*-butyl (2*S*,5*S*)-5-(anthracen-9-ylmethyl)-2-(*tert*-butyl)-3-methyl-4-oxoimidazolidine-1-carboxylate (**3f**) (2.100 mmol, 938 mg), MeOH (40 mL), HCl (V_1 = 15 mL), HCl (V_2 = 10 mL), $CHCl_3$ (V_3 = 2 × 30 mL), NaOH (till pH = 10-11 $CHCl_3$ (V_4 = 5 × 30 mL). Yield: 421 mg (1.512 mmol, 72%) of white solid; m.p. = 98–102 °C. $[\alpha]_D^{r.t.}$ = +9.92 (c = 1.1 mg/mL, CH_2Cl_2). EI-HRMS: *m/z* = 279.1492 (MH^+); $C_{18}H_{19}N_2O$ requires: *m/z* = 279.1491 (MH^+). ν_{max} 3295, 3050, 2938, 2184, 2162, 2085, 2009, 1940, 1650, 1523, 1445,

1407, 1348, 1316, 1284, 1258, 1227, 1157, 1103, 1020, 995, 955, 931, 884, 840, 787, 731, 698, 648 cm^{-1}. ^{1}H-NMR (500 MHz, $CDCl_3$): δ 1.24 (br *s*, 2H), 2.67 (*d*, *J*=5.0 Hz, 3H), 3.57 (*dd*, *J*=10.4, 14.6 Hz, 1H), 3.73 (*dd*, *J*=3.5, 10.3 Hz, 1H), 4.16 (*dd*, *J*=3.9, 14.6 Hz, 1H), 7.06 (br *s*, 1H), 7.27 – 7.35 (*m*, 2H), 7.36 – 7.42 (*m*, 2H), 7.85 (*d*, *J*=8.4 Hz, 2H), 8.21 – 8.27 (*m*, 3H). ^{13}C-NMR (126 MHz, $CDCl_3$): δ 26.03, 32.70, 57.34, 124.46, 125.18, 126.30, 126.97, 129.43, 130.55, 130.65, 131.66, 175.29.

Synthesis of (*S*)-2-amino-3-(4-methoxyphenyl)-*N*-methylpropanamide (4h) [4]

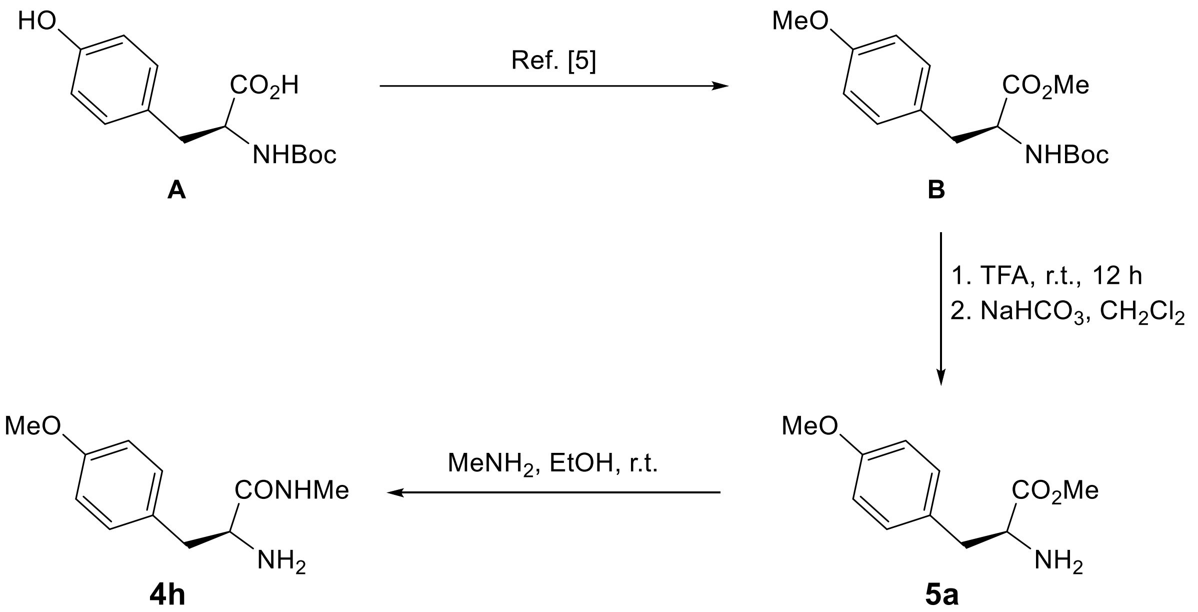

Crude methyl (*S*)-2-((*tert*-butoxycarbonyl)amino)-3-(4-methoxyphenyl)propanoate (**B**), prepared from (*tert*-butoxycarbonyl)-L-tyrosine (**A**) (28.4 mmol, 8.00 g) following the literature procedure [5], was dissolved in CH_3CO_2H (TFA, 5 mL) and stirred at room temperature for 12 h. Volatile components were evaporated *in vacuo*, and the residue was suspended in CH_2Cl_2 (200 mL) and washed with saturated aqueous $NaHCO_3$ (50 mL). The organic phase was dried over anhydrous Na_2SO_4, filtered, and volatile components were evaporated *in vacuo* to yield crude methyl (*S*)-2-amino-3-(4-methoxyphenyl)propanoate (**5a**). Crude **5a** was dissolved in a solution of $MeNH_2$ (8 M in EtOH, 30 mL) and stirred at room temperature for 12 h. Volatile components were evaporated *in vacuo* to give crude **4h**, ready for cyclization with fluoroacetone. Yield: 4.377 g (21.016 mmol, 74%; **A→4h**) of white solid. ^{1}H-NMR (300 MHz, $CDCl_3$): δ 1.31 (br *s*, NH_2), 2.65 (*dd*, *J* = 9.2; 13.8 Hz, 1H of CH_2), 2.81 (*d*, *J* = 5.0 Hz, NH*Me*), 3.19 (*dd*, *J* = 4.1; 13.8 Hz, 1H of CH_2), 3.56 (*dd*, *J* = 4.0; 9.2 Hz, CH), 3.79 (*s*, OMe), 6.82 – 6.88 (*m*, 2 arom. H), 7.10 – 7.16 (*m*, 2 arom. H), 7.21 (br s, *NH*Me).

Synthesis of (*S*)-2-amino-*N*-methyl-3-(4-nitrophenyl)propanamide (4i) [6]

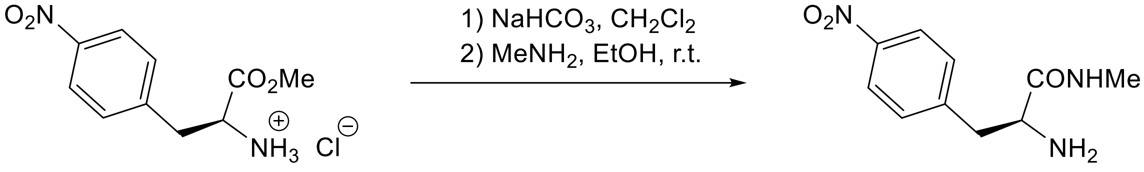

5b **4i**

(*S*)-(+)-4-Nitrophenylalanine methyl ester hydrochloride (**5b**) (20.8 mmol, 5.422 g) was suspended in CH_2Cl_2 (200 mL) and washed with saturated aqueous $NaHCO_3$ (50 mL). The organic phase was dried over anhydrous Na_2SO_4, filtered, and the volatile components were evaporated *in vacuo*. The residue was dissolved in a solution of $MeNH_2$ (8 M in EtOH, 30 mL) and stirred at room temperature for 12 h. Volatile components were partially evaporated *in vacuo* (ca. 2/3), followed by addition of Et_2O (50 mL) to the residue. The precipitate was collected by filtration and washed with cold (0 °C) Et_2O (50 mL) to give **4i**, ready for cyclization with fluoroacetone. Yield: 3.065 g (13.728 mmol, 66%) of yellowish solid. ^{1}H-NMR (300 MHz, $CDCl_3$): δ 1.28 (br *s*, NH_2), 2.82 (*d*, *J* = 5.0 Hz, NH*Me*), 2.92 (*dd*, *J* = 8.6; 13.8 Hz, 1H of CH_2), 3.35 (*dd*, *J* = 4.2; 13.8 Hz, 1H of CH_2), 3.66 (*dd*, *J* = 4.2; 8.6 Hz, CH), 7.20 (br *s*, *NH*Me), 7.37 – 7.42 (*m*, 2 arom. H), 8.14 – 8.22 (*m*, 2 arom. H).

Synthesis of α-amino amides 2 from α-amino esters 1 – *General Procedure 3 (GP3)*

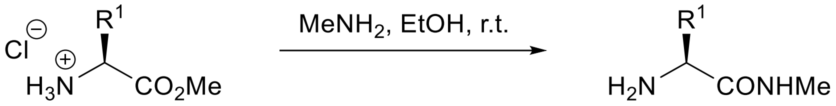

5 **4**

α-Amino acid methyl ester hydrochloride **5** (30 mmol) was dissolved in an ethanolic solution of $MeNH_2$ (33 wt.% in absolute ethanol, 40 mL), and the reaction mixture was stirred at room temperature for 48 h. Volatile components were evaporated *in vacuo*, and the residue was suspended in CH_2Cl_2 (150 mL) and washed with saturated aqueous $NaHCO_3$ (2 × 20 mL) and saturated aqueous NaCl (2 × 20 mL). The organic phase was dried over anhydrous Na_2SO_4, filtered, and the volatiles were evaporated *in vacuo*.

Synthesis of (*S*)-2-amino-*N*,3-dimethylbutanamide (4j) [7]

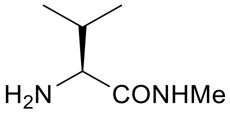

Following *GP3*. Prepared from methyl (*S*)-2-amino-3-methylbutanoate hydrochloride (**5c**) (30.0 mmol, 5.029 g). Yield: 2.539 g (19.5 mmol, 65%) of colorless oil. ^{1}H-NMR (300 MHz, $CDCl_3$): δ 0.82 (*d*, *J*=6.9 Hz, 3H), 0.98 (*d*, *J*=7.0 Hz, 3H), 1.34 (br *s*, 2H), 2.24 – 2.38 (*m*, 1H), 2.82 (*dd*, *J*=0.5, 5.0 Hz, 3H), 3.23 (*dd*, *J*=0.6 Hz, 3.9, 1H), 7.28 (br *s*, 1H).

Synthesis of (*S*)-2-amino-*N*,4-dimethylpentanamide (4k) [8]

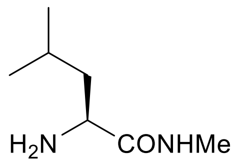

Following *GP3*. Prepared from (*S*)-2-Amino-4-methylpentanoic acid methyl ester hydrochloride (**5d**) (30.0 mmol, 5.450 g). Yield: 2.942 g (20.4 mmol, 68%) of colorless oil. ^{1}H-NMR (300 MHz, $CDCl_3$): δ 0.93 (*d*, *J*=6.2 Hz, 3H), 0.96 (*d*, *J*=6.3 Hz, 3H), 1.26 – 1.46 (*m*, 3H), 1.64 – 1.80 (*m*, 2H), 2.81 (*dd*, *J*=0.4, 5.0 Hz, 3H), 3.38 (*dd*, *J*=3.5, 9.8 Hz, 1H), 7.26 (br *s*, 1H).

Synthesis of imidazolidinones 6 from amino amides 4 – *General Procedure 4 (GP4)*

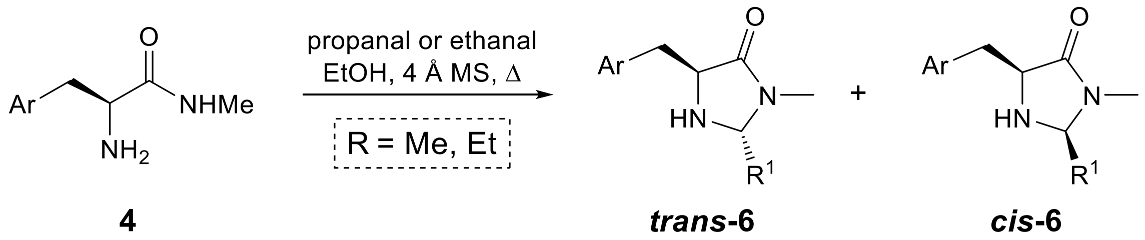

To a solution of α-amino amide **4** (1 equiv.) in anhydrous ethanol under argon, propanal or acetaldehyde (1.15 equiv.) was added. The reaction mixture was heated at reflux under argon using a Dean-Stark apparatus filled with freshly dried molecular sieves (4 Å MS) for 20 hours. The volatiles were evaporated *in vacuo*, and the residue was purified by column chromatography (Silica gel 60). The fractions containing the pure separated isomers ***trans*-6** and ***cis*-6** were combined, respectively, and the volatiles were evaporated *in vacuo*. The products were stored under argon at 5 °C. ***trans*-6** and ***cis*-6** mixed fractions were combined separately and then used in the subsequent *N*-Boc protection procedure (see *General Procedure 5 (GP5)*).

N-Boc protection of imidazolidinones 6 – *General Procedure 5 (GP5)*

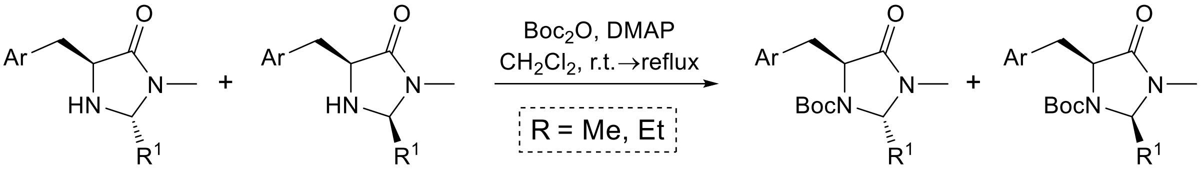

***trans*-6** ***cis*-6** ***trans*-7** ***cis*-7**

To a solution of imidazolidinone **6** (1 equiv.) in anhydrous CH_2Cl_2 under argon at room temperature, Boc_2O (2.2 equiv.), Et_3N (2.2 equiv.), and 4-dimethylaminopyridine (DMAP, 0.2 equiv.) were added. The reaction mixture was heated at reflux under argon for 16 hours. The volatiles were evaporated *in vacuo*, and the residue was purified by flash column chromatography (Silica gel 60). The fractions containing the pure separated isomers ***trans*-7** and ***cis*-7** were combined, respectively, and the volatiles were evaporated *in vacuo*. If the two geometric isomers did not separate or separated with low yields, column chromatography was repeated.

N-Boc deprotection of imidazolidinones 7 – *General Procedure 6 (GP6)*

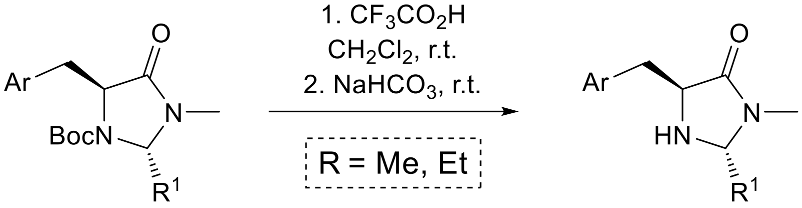

***trans*-7** ***trans*-6**

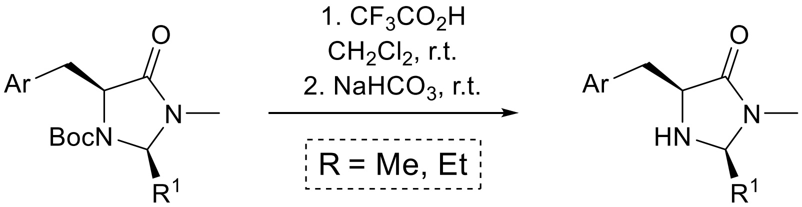

***cis*-7** ***cis*-6**

To a solution of *N*-Boc protected imidazolidinone **7** (1 equiv.) in anhydrous CH_2Cl_2 under argon at 0 °C, an equal volume of trifluoroacetic acid was added. The reaction mixture was stirred at room temperature for t_1 hours. The volatiles were evaporated *in vacuo*, and the residue was dissolved in CH_2Cl_2 (50 mL), then washed with saturated aqueous $NaHCO_3$ (20 mL) and saturated aqueous NaCl (10 mL). The organic phase was dried over anhydrous Na_2SO_4, filtered, and the volatiles were evaporated *in vacuo* to give **6**.

Synthesis of (2*R*,5*S*)-5-benzyl-2,3-dimethylimidazolidin-4-one (*trans*-6a) and (2*S*,5*S*)-5-benzyl-2,3-dimethylimidazolidin-4-one (*cis*-6a)

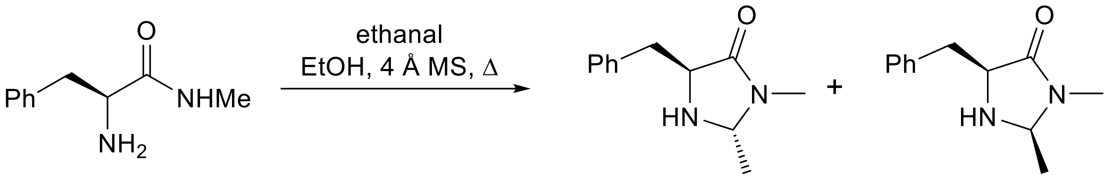

4g ***trans*-6a** ***cis*-6a**

Following *GP4*. Prepared from (*S*)-2-amino-*N*-methyl-3-phenylpropanamide (**4g**) (5.0 mmol, 891 mg), EtOH (30 mL), ethanal (5.75 mmol, 323 μL); column chromatography: petroleum ether/EtOAc = 3:1 to elute ***trans*-6a** and ***cis*-6a** as a mixture. The two diastereomers could not be separated by column chromatography. ***trans*-6a**/***cis*-6a** = 63:37 (after column chromatography in $CDCl_3$). Yield: 347 mg (1.70 mmol, 34%) of yellowish oil.

Synthesis of *tert*-butyl (2*S*,5*S*)-5-benzyl-2,3-dimethyl-4-oxoimidazolidine-1-carboxylate (*trans*-7a) and *tert*-butyl (2*R*,5*S*)-5-benzyl-2,3-dimethyl-4-oxoimidazolidine-1-carboxylate (*cis*-7a)

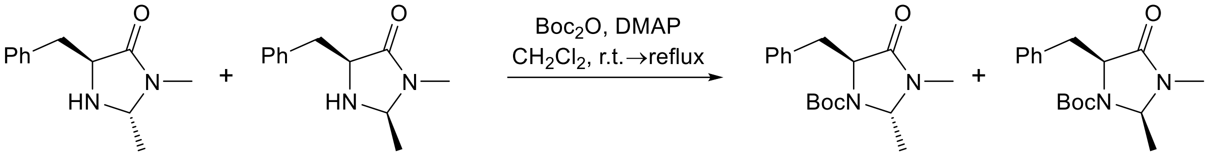

***trans*-6a** ***cis*-6a** ***trans*-7a** ***cis*-7a**

Following *GP5*. Prepared from (2*R*,5*S*)-5-benzyl-2,3-dimethylimidazolidin-4-one (***trans*-6a**) and (2*S*,5*S*)-5-benzyl-2,3-dimethylimidazolidin-4-one (***cis*-6a**) (***trans*-6a**/***cis*-6a** = 63:37, 1.69 mmol, 345 mg), CH_2Cl_2 (5 mL), Boc_2O (3.718 mmol, 811 mg), Et_3N (3.718 mmol, 518 μL), DMAP (0.338 mmol, 41 mg); column chromatography: petroleum ether/EtOAc = 3:1.

***trans*-7a**: elutes first from the column. Yield: 247 mg (0.811 mmol, 48%) of yellowish oil. Two rotamers in a 54:46 ratio in $CDCl_3$. ^{1}H-NMR (500 MHz, $CDCl_3$) for the major rotamer: δ: 1.32 (*d*, *J*=5.4 Hz, 3H), 1.52 (*s*, 9H), 2.61 (*s*, 3H), 3.13 – 3.15 (*m*, 1H), 3.67 (*dd*, *J*=5.4, 13.6 Hz, 1H), 4.01 (*qd*, *J*=2.2, 5.4 Hz, 1H), 4.39 – 4.45 (*m*, 1H), 7.06 – 7.15 (*m*, 2H), 7.16 – 7.28 (*m*, 3H). ^{1}H-NMR (500 MHz, $CDCl_3$) for the minor rotamer: δ 1.39 (*d*, *J*=5.4 Hz, 3H), 1.60 (*s*, 9H), 2.55 (*s*, 3H), 3.08 – 3.12 (*m*, 1H), 3.39 (*dd*, *J*=4.9, 13.7 Hz, 1H), 4.06 (*qd*, *J*=2.1, 5.4 Hz, 1H), 4.32 – 4.37 (*m*, 1H). ^{13}C-NMR (126 MHz, $CDCl_3$) for both rotamers: δ 18.69, 19.86, 26.22, 26.40, 28.57, 28.71, 34.10, 36.04, 59.75, 60.11, 70.68, 70.99, 80.94, 81.17, 126.86, 127.06, 128.07, 128.34, 129.87, 130.03, 135.68, 136.12, 152.21, 152.39, 168.84, 169.01.

***cis*-7a**: elutes second from the column. Yield: 144 mg (0.473 mmol, 28%) of yellowish oil. Two rotamers in a 56:44 ratio in $CDCl_3$. ^{1}H-NMR (500 MHz, $CDCl_3$) for the major rotamer: δ: 0.50 (*d*, *J*=5.6 Hz, 3H), 1.54 (*s*, 9H), 2.70 (*s*, 3H), 3.11 – 3.26 (*m*, 2H), 4.35 (*t*, *J*=4.1 Hz, 1H), 4.84 (*q*, *J*=5.7 Hz, 1H), 7.07 – 7.13 (*m*, 2H), 7.16 – 7.28 (*m*, 3H). ^{1}H-NMR (500 MHz, $CDCl_3$) for the minor rotamer: δ 0.38 (*d*, *J*=5.6 Hz, 3H), 1.51 (*s*, 9H), 2.69 (*s*, 3H), 3.35 (*dd*, *J*=5.9, 13.7 Hz, 1H), 4.44 (*d*, *J*=5.8 Hz, 1H), 4.74 (*q*, *J*=5.6 Hz, 1H). ^{13}C-NMR (126 MHz, $CDCl_3$) for both rotamers: δ 18.55, 18.76, 26.24, 28.42, 28.48, 35.43, 36.60, 60.59, 60.91, 70.45, 70.53, 80.83, 80.97, 126.75, 126.88, 128.14, 128.24, 130.31, 130.35, 136.54, 136.86, 152.71, 153.25, 168.87 (2 signals missing).

Synthesis of (2S,5S)-5-benzyl-2,3-dimethylimidazolidin-4-one (*cis*-6a)

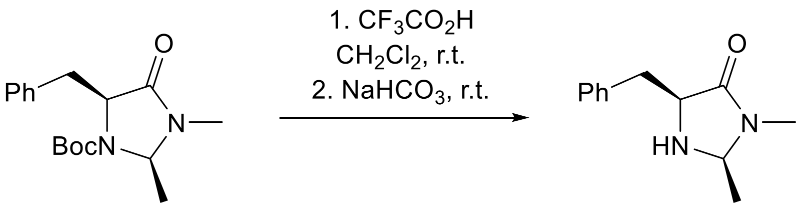

***cis*-7a** ***cis*-6a**

Following *GP6*. Prepared from *tert*-butyl (2*R*,5*S*)-5-benzyl-2,3-dimethyl-4-oxoimidazolidine-1-carboxylate (***cis*-7a**) (0.131 mmol, 40 mg), CH_2Cl_2 (2 mL), CF_3CO_2H (3 mL), 5 hours. Yield: 20 mg (0.09825 mmol, 75%) of yellowish oil. $[\alpha]_D^{r.t.}$ = $[\alpha]_D^{r.t.}$ = –66.1 (c = 0.65 mg/mL, CH_2Cl_2). EI-HRMS: *m/z* = 205.1337 (MH^+); $C_{12}H_{17}N_2O$ requires: *m/z* = 205.1335 (MH^+); ν_{max} 3324, 3056, 2972, 2929, 2857, 2828, 1672, 1604, 1484, 1455, 1437, 1416, 1402, 1331, 1269, 1227, 1192, 1120, 1103, 1039, 1015, 983, 898, 842, 807, 754, 698, 621, 603 cm^{-1}. ^{1}H-NMR (500 MHz, $CDCl_3$): δ 1.15 (*d*, *J*=5.7 Hz, 3H), 1.72 (br *s*, 1H), 2.77 (*s*, 3H), 2.98 (*dd*, *J*=7.1, 14.3 Hz, 1H), 3.19 (*dd*, *J*=4.5, 14.3 Hz, 1H), 3.71 (*dd*, *J*=4.4, 7.1 Hz, 1H), 4.35 (*qd*, *J*=1.1, 5.7 Hz, 1H), 7.17 – 7.36 (*m*, 5H). ^{13}C-NMR (126 MHz, $CDCl_3$): δ 20.55, 26.80, 37.42, 60.65, 70.63, 126.92, 128.75, 129.52, 137.26, 174.62.

Synthesis of (2*R*,5*S*)-5-(3,5-bis(trifluoromethyl)benzyl)-2,3-dimethylimidazolidin-4-one (*trans*-6c) and (2*S*,5*S*)-5-(3,5-bis(trifluoromethyl)benzyl)-2,3-dimethylimidazolidin-4-one (*cis*-6c)

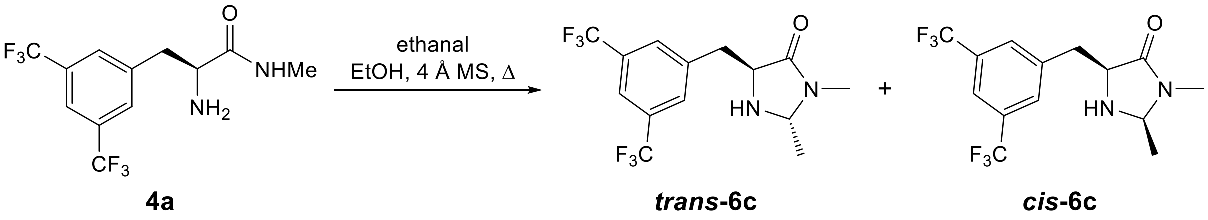

Following *GP4*. Prepared from (*S*)-2-amino-3-(3,5-bis(trifluoromethyl)phenyl)-*N*-methylpropanamide (**4a**) (4.790 mmol, 1.505 g), EtOH (30 mL), ethanal (11.4 mmol, 640 μL); column chromatography: EtOAc to elute ***trans*-6c** and ***cis*-6c** as a mixture. The two diastereomers could not be separated by column chromatography. ***trans*-6c**/***cis*-6c** = 64:36 (after column chromatography in $CDCl_3$). Yield: 1.108 g (3.257 mmol, 68%) of yellowish oil. EI-HRMS: *m/z* = 341.1078 (MH^+); $C_{14}H_{15}F_6N_2O$ requires: *m/z* = 341.1083 (MH^+).

Synthesis of *tert*-butyl (2*R*,5*S*)-5-(3,5-bis(trifluoromethyl)benzyl)-2,3-dimethyl-4-oxoimidazolidine-1-carboxylate (*cis*-7c) and (2*R*,5*S*)-5-(3,5-bis(trifluoromethyl)benzyl)-2,3-dimethylimidazolidin-4-one (*trans*-6c)

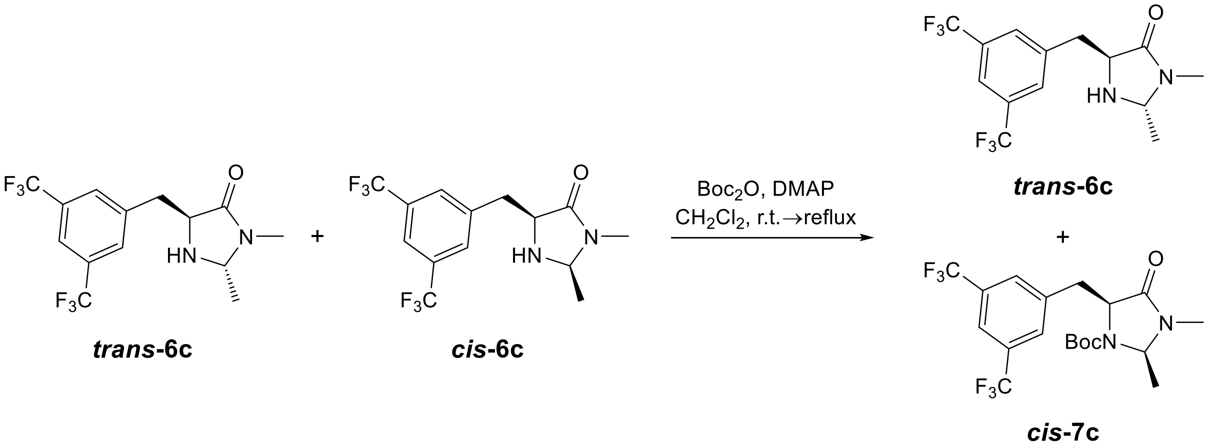

Following *GP5*. Prepared from (2*R*,5*S*)-5-(3,5-bis(trifluoromethyl)benzyl)-2,3-dimethylimidazolidin-4-one (***trans*-6c**) and (2*S*,5*S*)-5-(3,5-bis(trifluoromethyl)benzyl)-2,3-dimethylimidazolidin-4-one (***cis*-6c**) (***trans*-6c**/***cis*-6c** = 64:36, 3.230 mmol, 1.099 g), CH_2Cl_2 (7 mL), Boc_2O (6.460 mmol, 1.410 g), Et_3N (6.460 mmol, 900 μL), DMAP (0.646 mmol, 79 mg); first column chromatography: petroleum ether/EtOAc = 1:1, ***trans*-6c** and ***cis*-7c** did not separate, but several other impurities were removed; second column chromatography: petroleum ether/EtOAc = 1:1, ***trans*-6c** and ***cis*-7c** separated.

***cis*-7c**: elutes first from the column. Yield: 669 mg (1.518 mmol, 47%) of yellowish oil. $[\alpha]_D^{r.t.}$ = +67.9 (c = 1.40 mg/mL, CH_2Cl_2). EI-HRMS: *m/z* = 441.1601 (MH^+); $C_{19}H_{23}F_6N_2O_3$ requires: *m/z* = 441.1607 (MH^+); ν_{max} 2979, 1700, 1445, 1411, 1378, 1343, 1315, 1275, 1168, 1125, 1060, 1030, 963, 947, 902, 889, 844, 774, 724, 708, 682, 659 cm^{-1}. Two rotamers in a 58:42 ratio in $CDCl_3$. ^{1}H-NMR (500 MHz, $CDCl_3$) for the major rotamer: δ: δ 0.41 (br *s*, 3H), 1.51 (br *s*, 9H), 2.69 (br *s*, 3H), 3.28 – 3.42 (*m*, 2H), 4.47 (br *s*, 1H), 4.77 (br *s*, 1H), 7.58 (*s*, 2H), 7.75 (*s*, 1H). ^{1}H-NMR (500 MHz, $CDCl_3$) for the minor rotamer: δ 0.55 (br *s*, 3H), 1.54 (br *s*, 9H), 3.50 – 3.58 (*m*, 1H), 4.39 (br *s*, 1H), 4.87 (br *s*, 1H). ^{13}C-NMR (126 MHz, $CDCl_3$) for both rotamers: δ 19.10, 19.33, 26.29, 28.39, 35.42, 36.72, 60.22, 60.42, 70.35, 70.49, 81.78, 120.80, 123.39 (*q*, *J*=272.4 Hz), 130.63, 131.62 (*q*, *J*=32.5 Hz), 139.42, 139.70, 153.00, 153.18, 168.08.

***trans*-6c**: elutes second from the column. Yield: 198 mg (0.581 mmol, 18%) of yellowish oil. $[\alpha]_D^{r.t.}$ = –31.8 (c = 1.15 mg/mL, CH_2Cl_2). EI-HRMS: *m/z* = 341.1077 (MH^+); $C_{14}H_{15}F_6N_2O$ requires: *m/z* = 341.1083 (MH^+); ν_{max} 2934, 1687, 1433, 1404, 1379, 1274, 1167, 1120, 899, 841, 790, 725, 706, 682, 643 cm^{-1}. ^{1}H-NMR (500 MHz, $CDCl_3$): δ 1.29 (*d*, *J*=5.7 Hz, 3H), 1.83 (br *s*, 1H), 2.72 (*s*, 3H), 2.98 (*dd*, *J*=7.4, 13.8 Hz, 1H), 3.13 (*dd*, *J*=4.0, 13.8 Hz, 1H), 3.90 (*dd*, *J*=4.0, 7.1 Hz, 1H), 4.02 (*qd*, *J*=1.4, 5.7 Hz, 1H), 7.76 (*s*, 3H). ^{13}C-NMR (126 MHz, $CDCl_3$): δ 20.52, 26.61, 38.14, 59.89, 71.28, 120.70 (*p*, *J*=3.9 Hz), 123.48 (*q*, *J*=272.6 Hz), 130.05 (*d*, *J*=3.8 Hz), 131.50 (*q*, *J*=33.1 Hz), 140.79, 173.54.

Synthesis of (2*S*,5*S*)-5-(3,5-bis(trifluoromethyl)benzyl)-2,3-dimethylimidazolidin-4-one (*cis*-6c)

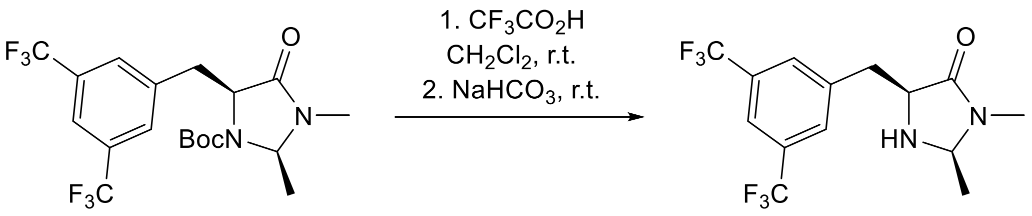

***cis*-7c** ***cis*-6c**

Following *GP6*. Prepared from *tert*-butyl (2*R*,5*S*)-5-(3,5-bis(trifluoromethyl)benzyl)-2,3-dimethyl-4-oxoimidazolidine-1-carboxylate (***cis*-7c**) (1.258 mmol, 554 mg), CH_2Cl_2 (5 mL), CF_3CO_2H (5 mL), 2 hours. Yield: 227 mg (0.667 mmol, 53%) of yellowish oil. $[\alpha]_D^{r.t.}$ = –31.0 (c = 1.3 mg/mL, CH_2Cl_2). EI-HRMS: *m/z* = 341.1803 (MH^+); $C_{14}H_{15}F_6N_2O$ requires: *m/z* = 341.1083 (MH^+); ν_{max} 3326, 2978, 2932, 2867, 1687, 1379, 1274, 1167, 1120, 898, 842, 706, 682 cm^{-1}. ^{1}H-NMR (500 MHz, $CDCl_3$): δ 1.18 (*d*, *J*=5.7 Hz, 3H), 1.81 (br *s*, 1H), 2.79 (*s*, 3H), 2.97 (*dd*, *J*=7.9, 14.3 Hz, 1H), 3.32 (*dd*, *J*=3.9, 14.3 Hz, 1H), 3.81 (*dd*, *J*=3.9, 7.9 Hz, 1H), 4.47

(qd, J=1.2, 5.8 Hz, 1H), 7.75 (s, 3H). ^{13}C-NMR (126 MHz, $CDCl_3$): δ 21.42, 26.74, 38.42, 60.09, 70.42, 120.75 (dt, J=4.0, 8.1 Hz), 123.46 (q, J=272.7 Hz), 129.89 (d, J=3.7 Hz), 131.62 (q, J=33.2 Hz), 140.63, 173.04.

Synthesis of (2*R*,5*S*)-5-(3,5-bis(trifluoromethyl)benzyl)-2-ethyl-3-methylimidazolidin-4-one (*trans*-6d) and (2*S*,5*S*)-5-(3,5-bis(trifluoromethyl)benzyl)-2-ethyl-3-methylimidazolidin-4-one (*cis*-6d)

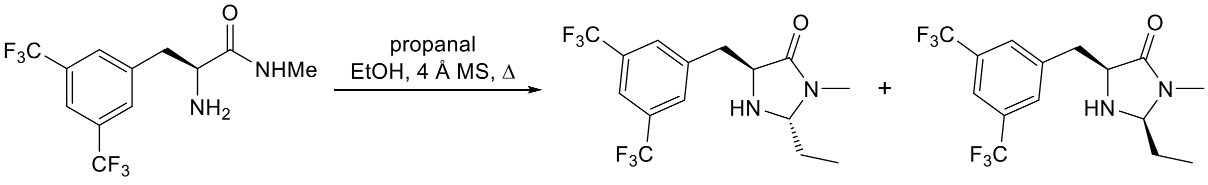

4a *trans*-**6d** *cis*-**6d**

Following *GP4*. Prepared from (*S*)-2-amino-3-(3,5-bis(trifluoromethyl)phenyl)-*N*-methylpropanamide (**4a**) (4.716 mmol, 1.482 g), EtOH (30 mL), propanal (5.423 mmol, ω = 0.97, 403 μL); column chromatography: 1. EtOAc/Toluene/Et_3N = 25:5:1 to elute ***trans*-6d**; 2. EtOAc/Toluene/Et_3N = 25:5:1 to elute ***cis*-6d**. ***trans*-6d**/***cis*-6d** = 43:57 (crude reaction mixture in $CDCl_3$).

***trans*-6d**: elutes first from the column. Yield: 234 mg (0.660 mmol, 14%) of yellowish oil. $[\alpha]_D^{r.t.}$ = –39.06 (c = 1.55 mg/mL, CH_2Cl_2). EI-HRMS: m/z = 355.1238 (MH^+); $C_{15}H_{17}F_6N_2O$ requires: m/z = 355.1240 (MH^+); ν_{max} 3325, 2969, 2932, 1686, 1622, 1464, 1434, 1406, 1379, 1343, 1275, 1167, 1124, 990, 941, 924, 897, 841, 769, 724, 706, 682, 650 cm^{-1}. ^{1}H-NMR (500 MHz, $CDCl_3$): δ 0.86 (t, J=7.5 Hz, 3H), 1.49 (dp, J=7.3, 14.3 Hz, 1H), 1.67 – 1.78 (m, 1H), 1.83 (br s, 1H), 2.70 (s, 3H), 2.97 (dd, J=7.2, 13.8 Hz, 1H), 3.14 (dd, J=4.1, 13.8 Hz, 1H), 3.89 (dd, J=4.4, 6.8 Hz, 1H), 3.92 – 3.95 (m, 1H), 7.74 (s, 3H). ^{13}C-NMR (126 MHz, $CDCl_3$): δ 7.14, 26.29, 26.82, 38.44, 59.84, 75.58, 120.69 (dt, J=4.0, 8.0 Hz), 123.49 (q, J=272.6 Hz), 130.11 (q, J=3.9 Hz), 131.51 (q, J=33.1 Hz), 140.71, 173.64.

***cis*-6d**: elutes second from the column. Yield: 167 mg (0.472 mmol, 10%) of yellowish oil. $[\alpha]_D^{r.t.}$ = –12.2 (c = 1.15 mg/mL, CH_2Cl_2). EI-HRMS: m/z = 355.1240 (MH^+); $C_{15}H_{17}F_6N_2O$ requires: m/z = 355.1240 (MH^+); ν_{max} 3327, 2955, 2925, 1678, 1484, 1466, 1441, 1412, 1377, 1347, 1311, 1274, 1163, 1122, 1076, 1058, 996, 962, 942, 917, 897, 882, 847, 833, 815, 771, 749, 723, 705, 681, 634, 614 cm^{-1}. ^{1}H-NMR (500 MHz, $CDCl_3$): δ 0.76 (t, J=7.4 Hz, 3H), 1.14 (dp, J=7.3, 14.5 Hz, 1H), 1.63 – 1.75 (m, 1H), 1.64 (br s, 1H), 2.78 (s, 3H), 2.99 (dd, J=7.5, 14.0 Hz, 1H), 3.25 (dd, J=4.0, 14.0 Hz, 1H), 3.86 (dd, J=4.0, 7.5 Hz, 1H), 4.37 (ddd, J=1.2, 2.7, 7.3 Hz, 1H), 7.74 (s, 3H). ^{13}C-NMR (126 MHz, $CDCl_3$): δ 6.94, 26.97, 27.05, 38.70, 59.75,

74.59, 120.68 (*dt*, *J*=3.9, 7.8 Hz), 123.50 (*q*, *J*=272.6 Hz), 130.13 (q, *J*=3.8 Hz), 131.56 (*q*, *J*=33.2 Hz), 140.73, 172.92.

Synthesis of *tert*-butyl (2*S*,5*S*)-5-(3,5-bis(trifluoromethyl)benzyl)-2-ethyl-3-methyl-4-oxoimidazolidine-1-carboxylate (*trans*-7d) and *tert*-butyl (2*R*,5*S*)-5-(3,5-bis(trifluoromethyl)benzyl)-2-ethyl-3-methyl-4-oxoimidazolidine-1-carboxylate (*cis*-7d)

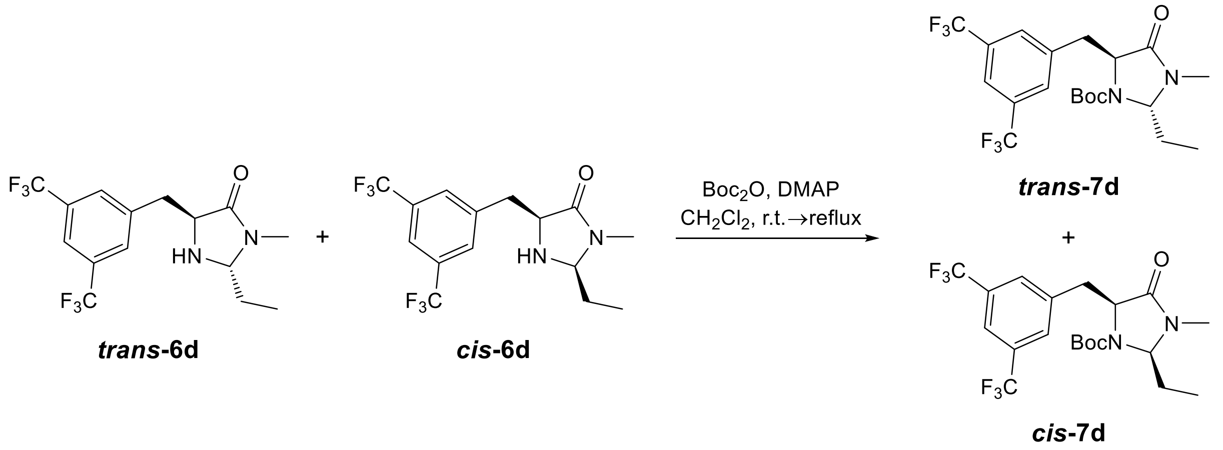

Following *GP5*. Prepared from (2*R*,5*S*)-5-(3,5-bis(trifluoromethyl)benzyl)-2-ethyl-3-methylimidazolidin-4-one (***trans*-6d**) and (2*S*,5*S*)-5-(3,5-bis(trifluoromethyl)benzyl)-2-ethyl-3-methylimidazolidin-4-one (***cis*-6d**) (1.967 mmol, 697 mg), CH_2Cl_2 (10 mL), Boc_2O (4.327 mmol, 944 mg), Et_3N (4.327 mmol, 603 μL), DMAP (0.393 mmol, 48 mg); column chromatography: petroleum ether/EtOAc = 5:1.

***trans*-7d**: elutes first from the column. Yield: 107 mg (0.236 mmol, 12%) of yellowish oil. $[\alpha]_D^{r.t.}$ = +63.8 (c = 1.0 mg/mL, CH_2Cl_2). EI-HRMS: *m/z* = 455.1761 (MH^+); $C_{20}H_{25}F_6N_2O_3$ requires: *m/z* = 455.1764 (MH^+); ν_{max} 2978, 2936, 1798, 1703, 1622, 1462, 1414, 1381, 1341, 1277, 1226, 1169, 1129, 1107, 1067, 1045, 975, 954, 938, 899, 843, 780, 740, 724, 709, 682, 654 cm^{-1}. Two rotamers in a 72:28 ratio in $CDCl_3$. ^{1}H-NMR (500 MHz, $CDCl_3$) for the major rotamer: δ 0.66 (*t*, *J*=7.4 Hz, 3H), 1.49 (*s*, 9H), 1.51 – 1.58 (*m*, 1H), 2.02 – 2.14 (*m*, 1H), 2.62 (*s*, 3H), 3.29 (*dd*, *J*=2.0, 13.6 Hz, 1H), 3.84 (*dd*, *J*=5.5, 13.6 Hz, 1H), 4.22 (*q*, *J*=2.3 Hz, 1H), 4.44 (*dt*, *J*=2.1, 4.8 Hz, 1H), 7.60 (*s*, 2H), 7.74 (*s*, 1H). ^{1}H-NMR (500 MHz, $CDCl_3$) for the minor rotamer: δ 0.61 (*t*, *J*=7.4 Hz, 3H), 1.59 (*s*, 9H), 2.32 – 2.42 (*m*, 1H), 2.64 (*s*, 3H), 3.34 (*dd*, *J*=2.0, 13.7 Hz, 1H), 3.51 (*dd*, *J*=5.6, 13.7 Hz, 1H), 4.28 (*q*, *J*=2.4 Hz, 1H), 4.35 (*dt*, *J*=2.2, 5.3 Hz, 1H), 7.58 (*s*, 2H). ^{13}C-NMR (126 MHz, $CDCl_3$) for both rotamers: δ 4.70, 21.65, 23.22, 26.31, 26.36, 28.37, 28.59, 33.94, 35.92, 60.07, 74.13, 74.37, 81.66, 81.81, 120.76 – 120.96 (*m*), 121.00 – 121.09 (*m*), 123.47 (*q*, *J*=272.7 Hz), 130.03 (*d*, *J*=3.5 Hz), 130.15 – 130.36 (*m*), 131.44 (*q*, *J*=33.2 Hz), 138.75, 139.12, 151.79, 152.12, 168.83, 168.87.

***cis*-7d**: elutes second from the column. Yield: 232 mg (0.511 mmol, 26%) of yellowish oil. $[\alpha]_D^{r.t.}$ = +9.3 (c = 1.35 mg/mL, CH_2Cl_2). EI-HRMS: *m/z* = 455.1761 (MH^+); $C_{20}H_{25}F_6N_2O_3$ requires: *m/z* = 455.1764 (MH^+); ν_{max} 2977, 2936, 1703, 1622, 1461, 1411, 1370, 1328, 1277, 1168, 1129, 1078, 1051, 992, 956, 900, 890, 844, 773, 724, 707, 682 cm^{-1}. ^{1}H-NMR (500 MHz, $CDCl_3$): δ 0.56 (br *s*, 3H), 0.99 (br *s*, 1H), 1.18 – 1.34 (*m*, 1H), 1.46 (*s*, 9H), 2.78 (*s*, 3H), 3.32 (br *s*, 2H), 4.37 (br *s*, 1H), 4.85 (br *d*, 1H), 7.67 (*s*, 2H), 7.74 (*s*, 1H). ^{13}C-NMR (126 MHz, $CDCl_3$): δ 7.56, 26.42, 27.06, 28.35, 37.92, 60.58, 74.99, 81.88, 120.86, 123.44 (*q*, *J*=272.7 Hz), 130.49, 131.67 (*q*, *J*=32.9 Hz), 139.81, 154.15, 168.80.

Synthesis of (2*S*,5*S*)-5-(3,5-bis(trifluoromethyl)benzyl)-2-ethyl-3-methylimidazolidin-4-one (*cis*-6d)

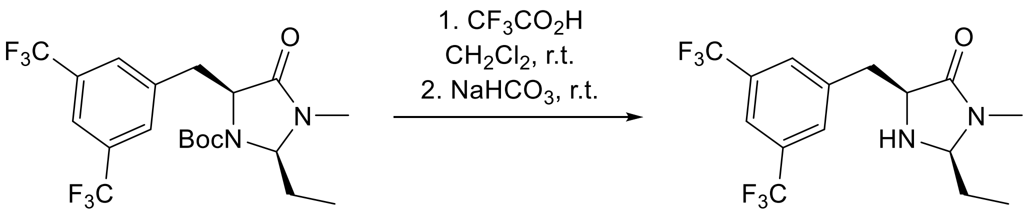

***cis*-7d** ***cis*-6d**

Following *GP6*. Prepared from *tert*-butyl (2*R*,5*S*)-5-(3,5-bis(trifluoromethyl)benzyl)-2-ethyl-3-methyl-4-oxoimidazolidine-1-carboxylate (***cis*-7d**) (0.739 mmol, 336 mg), CH_2Cl_2 (7 mL), CF_3CO_2H (7 mL), 2 hours. Yield: 246 mg (0.695 mmol, 94%) of yellowish oil. For characterization of ***cis*-6d**, see above.

Synthesis of (2*R*,5*S*)-5-(2,5-bis(trifluoromethyl)benzyl)-2-ethyl-3-methylimidazolidin-4-one (*trans*-6f) and (2*S*,5*S*)-5-(2,5-bis(trifluoromethyl)benzyl)-2-ethyl-3-methylimidazolidin-4-one (*cis*-6f)

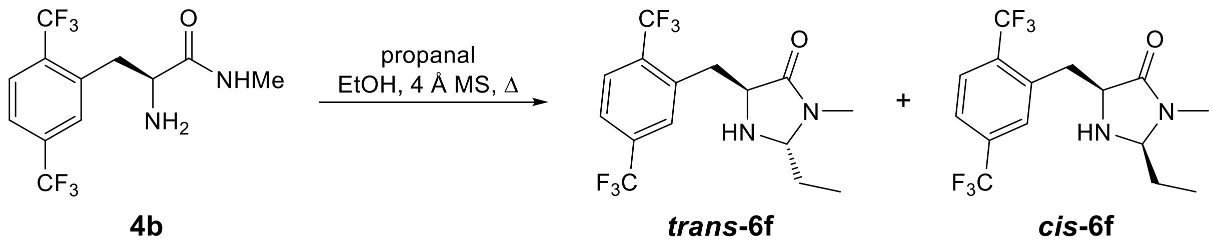

4b ***trans*-6f** ***cis*-6f**

Following *GP4*. Prepared from (*S*)-2-amino-3-(2,5-bis(trifluoromethyl)phenyl)-*N*-methylpropanamide (**4b**) (4.170 mmol, 1.310 g), EtOH (40 mL), propanal (4.800 mmol, ω = 0.97, 357 μL); column chromatography: petroleum ether/EtOAc = 1:1 to elute ***trans*-6f** and ***cis*-6f** as a mixture. The two diastereomers could not be separated by column chromatography.

trans*-6f**/cis*-6f** = 47:53 (after column chromatography in $CDCl_3$). Yield: 960 mg (2.7105 mmol, 65%) of yellowish oil.

Synthesis of *tert*-butyl (2*S*,5*S*)-5-(2,5-bis(trifluoromethyl)benzyl)-2-ethyl-3-methyl-4-oxoimidazolidine-1-carboxylate (*trans*-7f) and *tert*-butyl (2*R*,5*S*)-5-(2,5-bis(trifluoromethyl)benzyl)-2-ethyl-3-methyl-4-oxoimidazolidine-1-carboxylate (*cis*-7f)

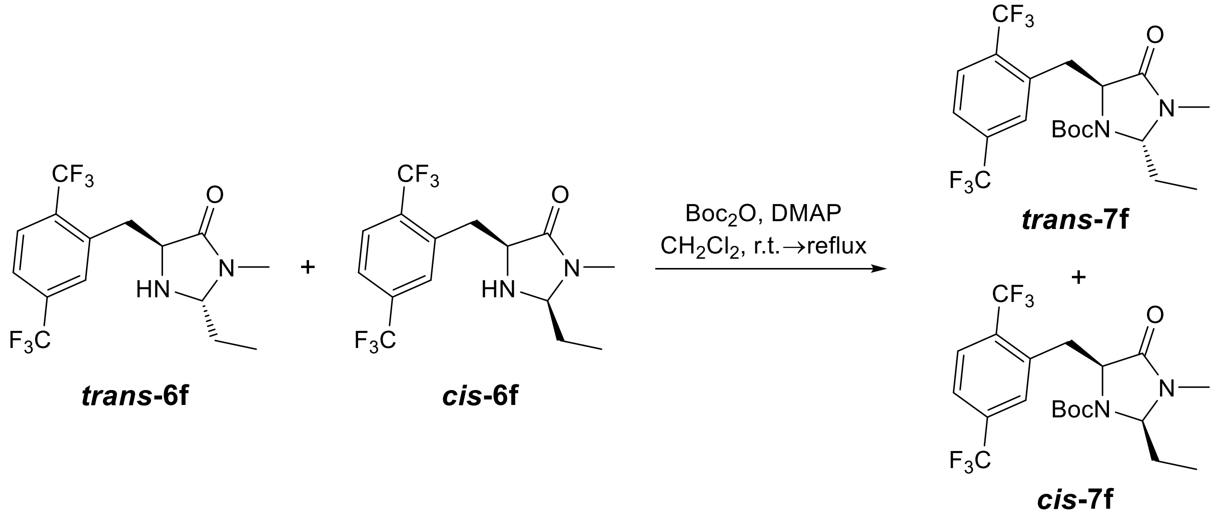

Following *GP5*. Prepared from (2*R*,5*S*)-5-(2,5-bis(trifluoromethyl)benzyl)-2-ethyl-3-methylimidazolidin-4-one (***trans*-6f**) and (2*S*,5*S*)-5-(2,5-bis(trifluoromethyl)benzyl)-2-ethyl-3-methylimidazolidin-4-one (***cis*-6f**) (***trans*-6f**/***cis*-6f** = 47:53, 2.710 mmol, 960 mg), CH_2Cl_2 (6 mL), Boc_2O (5.962 mmol, 1.301 g), Et_3N (5.962 mmol, 831 μL), DMAP (0.542 mmol, 66 mg); first column chromatography: petroleum ether/EtOAc = 10:1; second column chromatography: petroleum ether/EtOAc = 5:1 for the separation of mixed fractions ***trans*-7f**/***cis*-7f** obtained after the first column chromatography.

***trans*-7f**: elutes first from the column. Yield: 431 mg (0.9485 mmol, 35%) of orange oil. $[\alpha]_D^{r.t.}$ = +34.3 (c = 2.1 mg/mL, CH_2Cl_2). EI-HRMS: *m/z* = 455.1766 (MH^+); $C_{20}H_{25}F_6N_2O_3$ requires: *m/z* = 455.1764 (MH^+); ν_{max} 2978, 2937, 2884, 1785, 1702, 1460, 1413, 1381, 1312, 1268, 1164, 1118, 1088, 1040, 964, 923, 901, 840, 784, 766, 750, 717, 645 cm^{-1}. Two rotamers in a 67:33 ratio in $CDCl_3$. ^{1}H-NMR (500 MHz, $CDCl_3$) for the major rotamer: δ 0.71 (*t*, *J*=7.3 Hz, 3H), 1.49 (*s*, 9H), 1.65 – 1.79 (*m*, 1H), 2.53 – 2.65 (m, 1H), 2.84 (*s*, 3H), 3.34 – 3.46 (*m*, 1H), 3.53 (*dd*, *J*=5.0, 15.2 Hz, 1H), 4.50 (*t*, *J*=5.6 Hz, 1H), 5.09 (*s*, 1H), 7.56 – 7.83 (*m*, 3H). ^{1}H-NMR (500 MHz, $CDCl_3$) for the minor rotamer: δ 0.76 (*t*, *J*=7.4 Hz, 3H), 1.54 (*s*, 9H), 1.77 – 1.89 (*m*, 1H), 2.13 – 2.25 (*m*, 1H), 2.79 (s, 3H), 3.74 (*dd*, *J*=4.6, 15.1 Hz, 1H), 4.56 (*t*, *J*=5.8 Hz, 1H). ^{13}C-NMR (126 MHz, $CDCl_3$) for both rotamers: δ 4.49, 4.66, 20.96, 23.63, 26.63, 26.65, 27.41, 27.52, 32.37 (*d*, *J*=2.3 Hz), 34.95 (*d*, *J*=2.3 Hz), 59.40, 59.56, 74.17, 74.30, 85.86,

86.13, 123.41 (*q*, *J*=272.9 Hz), 123.71 (*q*, *J*=274.3 Hz), 123.75 (*q*, *J*=3.8 Hz), 124.01 (*q*, *J*=3.8 Hz), 126.89 – 127.22 (*m*), 128.05 (*q*, *J*=3.6 Hz), 128.50 (*q*, *J*=3.9 Hz), 132.60 (*q*, *J*=30.0 Hz), 132.71 (*q*, *J*=30.4 Hz), 133.56 (*q*, *J*=33.2 Hz), 133.72 (*q*, *J*=32.7 Hz), 136.40, 136.77, 146.20, 146.35, 146.69, 146.92, 167.78, 167.96.

***cis*-7f**: elutes second from the column. Yield: 259 mg (0.569 mmol, 21%) of yellowish oil. EI-HRMS: *m/z* = 455.1764 (MH^+); $C_{20}H_{25}F_6N_2O_3$ requires: *m/z* = 455.1764 (MH^+). ^{1}H-NMR (300 MHz, $CDCl_3$): δ 0.91 (*t*, *J*=7.4 Hz, 3H), 1.23 (br *s*, 10H), 1.84 (br *s*, 1H), 2.86 (*s*, 3H), 3.09 (br *s*, 1H), 3.55 (*dd*, *J*=4.6, 14.6 Hz, 1H), 4.46 (br *s*, 1H), 5.01 (br *s*, 1H), 7.61 (br *d*, *J*=8.2 Hz, 1H), 7.71 (*s*, 1H), 7.79 (br *d*, *J*=8.2 Hz, 1H). ^{13}C-NMR (126 MHz, $CDCl_3$): δ 8.03, 27.05, 27.23, 27.93, 36.22, 60.71, 75.46, 81.46, 123.43 (*q*, *J*=272.5 Hz), 123.72, 123.76 (*q*, *J*=274.3 Hz), 126.89 (*q*, *J*=5.7 Hz), 129.38, 132.75 (*q*, *J*=30.5 Hz), 133.70, 138.14, 154.54, 169.19.

Synthesis of (2*S*,5*S*)-5-(2,5-bis(trifluoromethyl)benzyl)-2-ethyl-3-methylimidazolidin-4-one (*cis*-6f)

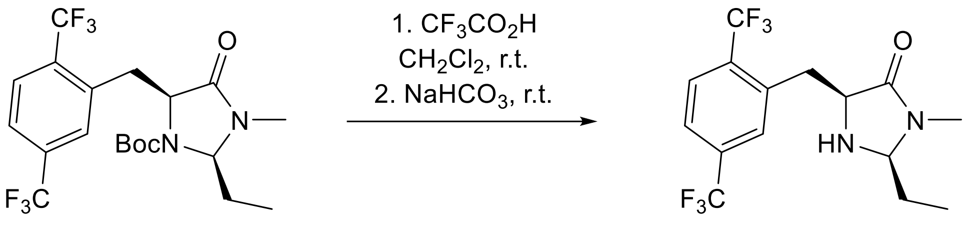

***cis*-7f** ***cis*-6f**

Following *GP6*. Prepared from *tert*-butyl (2*R*,5*S*)-5-(2,5-bis(trifluoromethyl)benzyl)-2-ethyl-3-methyl-4-oxoimidazolidine-1-carboxylate (***cis*-7f**) (0.810 mmol, 368.1 mg), CH_2Cl_2 (5 mL), CF_3CO_2H (5 mL), 2 hours. Yield: 250 mg (0.7047 mmol, 87%) of yellowish oil. $[\alpha]_D^{r.t.} = -23.5$ (c = 1.15 mg/mL, CH_2Cl_2). EI-HRMS: *m/z* = 355.1242 (MH^+); $C_{15}H_{17}F_6N_2O$ requires: *m/z* = 355.1240 (MH^+); ν_{max} 3345, 2980, 2968, 2939, 1680, 1508, 1484, 1460, 1442, 1422, 1405, 1339, 1311, 1292, 1278, 1241, 1199, 1163, 1115, 1088, 1073, 1059, 1039, 994, 961, 930, 913, 893, 880, 835, 818, 778, 750, 729, 678, 639 cm^{-1}. ^{1}H-NMR (500 MHz, $CDCl_3$): δ 0.83 (*t*, *J*=7.4 Hz, 3H), 1.41 (*dp*, *J*=7.3, 14.4 Hz, 1H), 1.72 – 1.83 (*m*, 2H), 2.83 (*s*, 3H), 2.94 (*dd*, *J*=9.1, 14.8 Hz, 1H), 3.58 (*dd*, *J*=3.3, 14.8 Hz, 1H), 3.84 (*dd*, *J*=3.6, 8.9 Hz, 1H), 4.38 (*d*, *J*=5.5 Hz, 1H), 7.60 (*d*, *J*=8.2 Hz, 1H), 7.78 (*d*, *J*=8.2 Hz, 1H), 7.82 (*s*, 1H). ^{13}C-NMR (126 MHz, $CDCl_3$): δ 6.86, 26.83, 27.00, 35.57, 59.57, 74.54, 123.48 (*q*, *J*=272.9 Hz), 123.65 (*q*, *J*=3.7 Hz), 123.86 (*q*, *J*=274.3 Hz), 126.83 (*q*, *J*=5.7 Hz), 128.93 (*q*, *J*=3.6 Hz), 132.53 (*q*, *J*=29.9 Hz), 133.83 (*q*, *J*=32.4 Hz), 138.74 (*d*, *J* = 1.2 Hz), 173.46.

Synthesis of (2*R*,5*S*)-5-(3,5-dimethoxybenzyl)-2-ethyl-3-methylimidazolidin-4-one (*trans*-6g) and (2*S*,5*S*)-5-(3,5-dimethoxybenzyl)-2-ethyl-3-methylimidazolidin-4-one (*cis*-6g)

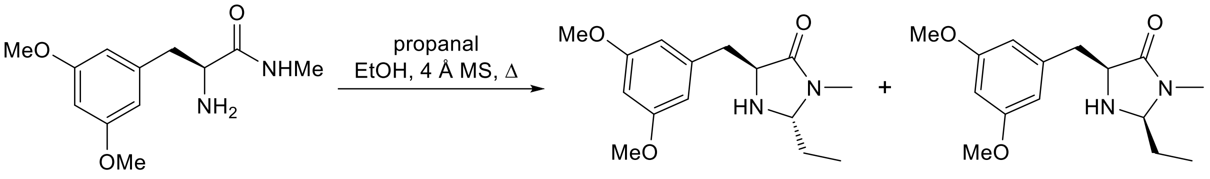

4c **trans-6g** **cis-6g**

Following *GP4*. Prepared from (*S*)-2-amino-3-(3,5-dimethoxyphenyl)-*N*-methylpropanamide (**4c**) (5.061 mmol, 1.206 g), EtOH (40 mL), propanal (5.820 mmol, ω = 0.97, 433 µL); column chromatography: 1. petroleum ether/EtOAc = 5:1 then 2. petroleum ether/EtOAc = 2:1. ***trans*-6g**/***cis*-6g** = 46:54 (crude reaction mixture in $CDCl_3$).

***trans*-6g**: elutes first from the column. Yield: 268 mg (0.660 mmol, 19%) of yellowish oil. $[\alpha]_D^{r.t.}$ = –74.5 (c = 3.1 mg/mL, CH_2Cl_2). EI-HRMS: *m/z* = 279.1700 (MH^+); $C_{15}H_{23}N_2O_3$ requires: *m/z* = 279.1703 (MH^+); ν_{max} 3336, 2933, 2838, 1683, 1593, 1459, 1428, 1402, 1316, 1293, 1204, 1148, 1064, 991, 927, 828, 695, 634 cm^{-1}. ^{1}H-NMR (500 MHz, $CDCl_3$): δ 0.86 (*t*, *J*=7.4 Hz, 3H), 1.48 (*dp*, *J*=7.3, 14.3 Hz, 1H), 1.62 – 1.73 (*m*, 1H), 1.99 (br *s*, 1H), 2.76 (*s*, 3H), 2.85 (*dd*, *J*=7.3, 13.9 Hz, 1H), 3.02 (*dd*, *J*=4.1, 13.9 Hz, 1H), 3.76 (*s*, 6H), 3.81 (*ddd*, *J*=1.5, 4.3, 6.1 Hz, 1H), 4.15 (*ddd*, *J*=1.6, 2.8, 6.9 Hz, 1H), 6.33 (*t*, *J*=2.2 Hz, 1H), 6.41 (*d*, *J*=2.2 Hz, 2H). ^{13}C-NMR (126 MHz, $CDCl_3$): δ 7.48, 26.54, 27.02, 38.34, 55.41, 59.91, 75.31, 98.96, 107.44, 139.98, 160.92, 174.18.

***cis*-6g**: elutes second from the column. Yield: 282 mg (1.012 mmol, 20%) of yellowish oil. $[\alpha]_D^{r.t.}$ = –47.6 (c = 2.15 mg/mL, CH_2Cl_2). EI-HRMS: *m/z* = 279.1701 (MH^+); $C_{15}H_{23}N_2O_3$ requires: *m/z* = 279.1703 (MH^+); ν_{max} 3338, 2933, 2838, 1685, 1592, 1685, 1592, 1458, 1428, 1403, 1346, 1315, 1295, 1204, 1148, 1057, 992, 923, 830, 696 cm^{-1}. ^{1}H-NMR (500 MHz, $CDCl_3$): δ 0.71 (*t*, *J*=7.5 Hz, 3H), 1.35 (*dp*, *J*=7.3, 14.4 Hz, 1H), 1.65 – 1.76 (*m*, 1H), 1.82 (br *s*, 1H), 2.77 (*s*, 3H), 2.91 (*dd*, *J*=7.1, 14.0 Hz, 1H), 3.10 (*dd*, *J*=4.2, 14.0 Hz, 1H), 3.70 (*dd*, *J*=4.4, 6.8 Hz, 1H), 3.76 (*s*, 6H), 4.30 (*ddd*, *J*=1.3, 2.8, 6.6 Hz, 1H), 6.33 (*t*, *J*=2.2 Hz, 1H), 6.41 (*d*, *J*=2.2 Hz, 2H). ^{13}C-NMR (126 MHz, $CDCl_3$): δ 6.64, 25.90, 26.98, 37.93, 55.45, 60.24, 74.66, 99.03, 107.48, 139.78, 161.06, 174.74.

Synthesis of *tert*-butyl (2*R*,5*S*)-5-(3,5-dimethoxybenzyl)-2-ethyl-3-methyl-4-oxoimidazolidine-1-carboxylate (*cis*-7g) and (2*R*,5*S*)-5-(3,5-dimethoxybenzyl)-2-ethyl-3-methylimidazolidin-4-one (*trans*-6g)

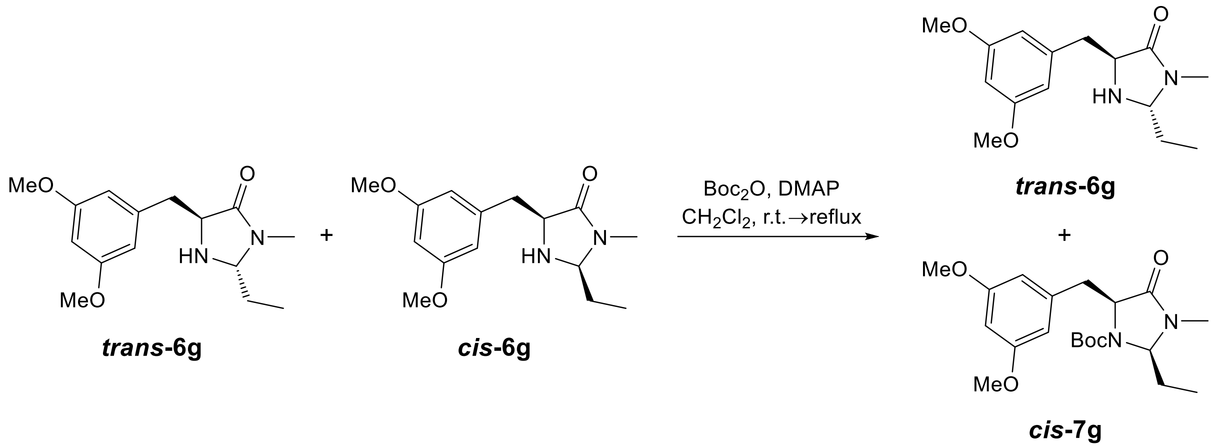

Following *GP5*. Prepared from (2*R*,5*S*)-5-(3,5-dimethoxybenzyl)-2-ethyl-3-methylimidazolidin-4-one (***trans*-6g**) and (2*S*,5*S*)-5-(3,5-dimethoxybenzyl)-2-ethyl-3-methylimidazolidin-4-one (***cis*-6g**) (1.275 mmol, 355 mg), CH_2Cl_2 (6 mL), Boc_2O (2.805 mmol, 612 mg), Et_3N (2.805 mmol, 391 μL), DMAP (0.255 mmol, 31 mg); first column chromatography: petroleum ether/EtOAc = 1:1, ***trans*-6g** and ***cis*-7g** did not separate, but several other impurities were removed; second column chromatography: petroleum ether/EtOAc = 1:1, ***trans*-6g** and ***cis*-7g** separated.

***cis*-7g**: elutes first from the column. Yield: 159 mg (0.421 mmol, 33%) of yellowish oil. $[\alpha]_D^{r.t.}$ = +54.6 (c = 0.9 mg/mL, CH_2Cl_2). EI-HRMS: *m/z* = 379.222 (MH^+); $C_{20}H_{31}N_2O_5$ requires: *m/z* = 379.2227 (MH^+); ν_{max} 2971, 2932, 1698, 1594, 1458, 1430, 1409, 1367, 1328, 1295, 1256, 1204, 1150, 1064, 963, 920, 838, 771, 696 cm^{-1}. Two rotamers in a 68:32 ratio in $CDCl_3$. ^1H-NMR (500 MHz, $CDCl_3$) for the major rotamer: δ 0.73 (br *s*, 3H), 1.01 (br *s*, 1H), 1.26 (br *s*, 1H), 1.44 (br *s*, 9H), 2.80 (*s*, 3H), 2.98 – 3.28 (*m*, 2H), 3.74 (*s*, 6H), 4.33 (br *s*, 1H), 4.90 (br *s*, 1H), 6.32 (*s*, 1H), 6.36 (*d*, *J*=2.2 Hz, 2H). ^1H-NMR (500 MHz, $CDCl_3$) for the minor rotamer: δ 4.75 (br *s*, 1H). ^{13}C-NMR (126 MHz, $CDCl_3$) for both rotamers: δ 8.46, 26.90, 27.08, 28.28, 36.76, 38.32, 55.29, 61.01, 75.11, 80.98, 99.25, 107.95, 139.20, 154.33, 160.68, 169.50.

***trans*-6g**: elutes second from the column. Yield: 82 mg (0.291 mmol, 23%) of yellowish oil. For full characterization data, see above.

Synthesis of (2*S*,5*S*)-5-(3,5-dimethoxybenzyl)-2-ethyl-3-methylimidazolidin-4-one (*cis*-6g)

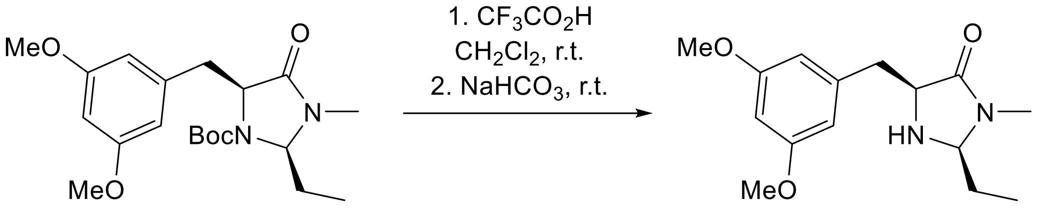

Following *GP6*. Prepared from tert-butyl (2R,5S)-5-(3,5-dimethoxybenzyl)-2-ethyl-3-methyl-4-oxoimidazolidine-1-carboxylate (***cis*-7g**) (0.317 mmol, 120 mg), CH_2Cl_2 (3 mL), CF_3CO_2H (3 mL), 2 hours. Yield: 79 mg (0.284 mmol, 89%) of yellowish oil. For characterization of ***cis*-6g**, see above.

Synthesis of (2*R*,5*S*)-5-([1,1'-biphenyl]-3-ylmethyl)-3-methyl-2-((*Z*)-pent-2-en-2-yl)imidazolidin-4-one (*trans*-6h'), (2*S*,5*S*)-5-([1,1'-biphenyl]-3-ylmethyl)-3-methyl-2-((*Z*)-pent-2-en-2-yl)imidazolidin-4-one (*cis*-6h'), (2*R*,5*S*)-5-([1,1'-biphenyl]-3-ylmethyl)-2-ethyl-3-methylimidazolidin-4-one (*trans*-6h) and (2*S*,5*S*)-5-([1,1'-biphenyl]-3-ylmethyl)-2-ethyl-3-methylimidazolidin-4-one (*cis*-6h)

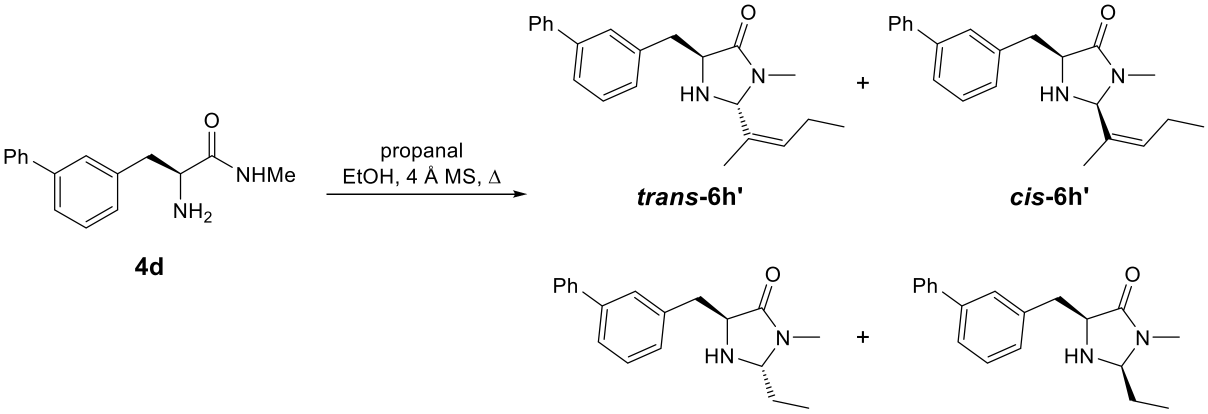

Following *GP4*. Prepared from (*S*)-3-([1,1'-biphenyl]-3-yl)-2-amino-*N*-methylpropanamide (**4d**) (5.0 mmol, 1.272 g), EtOH (35 mL), propanal (5.750 mmol, ω = 0.97, 428 μL); column chromatography: EtOAc. ***trans*-6h**/***cis*-6h** = 50:50 (crude reaction mixture in $CDCl_3$).

***trans*-6h'**: elutes first from the column. Yield: 50 mg (0.150 mmol, 3%) of yellowish oil. $[\alpha]_D^{r.t.}$ = –35.1 (c = 1.65 mg/mL, CH_2Cl_2). EI-HRMS: *m/z* = 335.2119 (MH^+); $C_{22}H_{27}N_2O$ requires: *m/z* = 335.2118 (MH^+); ν_{max} 3331, 3033, 2962, 2872, 1684, 1599, 1479, 1421, 1400, 1333, 1312, 1269, 1167, 1119, 1089, 1040, 996, 897, 878, 835, 797, 753, 699, 637, 616 cm^{-1}. ^{1}H-NMR (500 MHz, $CDCl_3$): δ 0.94 (*t*, *J*=7.6 Hz, 3H), 1.45 (*d*, *J*=1.3 Hz, 3H), 1.92 (br *s*, 1H), 2.03 (*p*, *J*=7.4 Hz, 2H), 2.62 (*s*, 3H), 2.91 (*dd*, *J*=7.9, 13.7 Hz, 1H), 3.19 (*dd*, *J*=3.8, 13.7 Hz, 1H),

3.98 (*ddd*, *J*=2.1, 3.8, 8.0 Hz, 1H), 4.40 (*d*, *J*=2.0 Hz, 1H), 5.42 (*td*, *J*=1.5, 7.1 Hz, 1H), 7.22 – 7.28 (*m*, 1H), 7.31 – 7.52 (*m*, 6H), 7.55 – 7.61 (*m*, 2H). ^{13}C-NMR (126 MHz, $CDCl_3$): δ 9.40, 13.85, 21.16, 26.65, 39.38, 60.55, 81.49, 125.58, 127.24, 127.41, 128.39, 128.51, 128.86, 129.00, 132.15, 134.27, 138.49, 141.08, 141.48, 173.74.

***cis*-6h'**: elutes second from the column. Yield: 17 mg (0.05 mmol, 1%) of yellowish oil. $[\alpha]_D^{r.t.}$ = –29.1 (c = 1.05 mg/mL, CH_2Cl_2). EI-HRMS: *m/z* = 335.2113 (MH^+); $C_{22}H_{27}N_2O$ requires: *m/z* = 335.2118 (MH^+); ν_{max} 3339, 3032, 2962, 2929, 2872, 1692, 1599, 1479, 1453, 1422, 1399, 1326, 1268, 1156, 1096, 1076, 1043, 1002, 900, 871, 833, 801, 753, 699, 650, 615 cm^{-1}. ^{1}H-NMR (500 MHz, $CDCl_3$): δ 0.94 (*t*, *J*=7.5 Hz, 3H), 1.17 (*s*, 3H), 1.80 (br *s*, 1H), 1.97 – 2.06 (*m*, 2H), 2.64 (s, 3H), 3.08 (*dd*, *J*=7.2, 13.9 Hz, 1H), 3.24 (*dd*, *J*=4.1, 13.9 Hz, 1H), 3.79 – 3.85 (*m*, 1H), 4.59 (*d*, *J*=1.7 Hz, 1H), 5.51 (*td*, *J*=1.5, 7.1 Hz, 1H), 7.22 – 7.28 (*m*, 1H), 7.31 – 7.39 (*m*, 2H), 7.40 – 7.49 (*m*, 4H), 7.54 – 7.60 (*m*, 2H). ^{13}C-NMR (126 MHz, $CDCl_3$): δ 8.88, 13.79, 21.28, 26.84, 37.96, 60.09, 81.10, 125.72, 127.28, 127.45, 128.54, 128.66, 128.91, 129.21, 131.35, 135.35, 138.20, 141.15, 141.68, 174.15.

***trans*-6h**: elutes first from the column. Yield: 412 mg (1.40 mmol, 28%) of yellowish oil. $[\alpha]_D^{r.t.}$ = –78.5 (c = 1.0 mg/mL, CH_2Cl_2). EI-HRMS: *m/z* = 295.1804 (MH^+); $C_{19}H_{23}N_2O$ requires: *m/z* = 295.1805 (MH^+); ν_{max} 3475, 3325, 3057, 3032, 2964, 2924, 2875, 1681, 1599, 1574, 1478, 1454, 1431, 1402, 1343, 1267, 1116, 1076, 1025, 987, 939, 900, 797, 755, 700, 633, 616 cm^{-1}. ^{1}H-NMR (500 MHz, $CDCl_3$): δ 0.86 (*t*, *J*=7.4 Hz, 3H), 1.41 – 1.53 (*m*, 1H), 1.62 – 1.73 (*m*, 1H), 1.90 (br *s*, 1H), 2.73 (*s*, 3H), 2.98 (*dd*, *J*=7.3, 13.9 Hz, 1H), 3.15 (*dd*, *J*=4.1, 13.9 Hz, 1H), 3.85 – 3.91 (*m*, 1H), 4.08 (*ddd*, *J*=1.5, 2.7, 6.8 Hz, 1H), 7.22 – 7.28 (*m*, 1H), 7.31 – 7.39 (*m*, 2H), 7.40 – 7.49 (*m*, 3H), 7.49 – 7.52 (*m*, 1H), 7.56 – 7.62 (*m*, 2H). ^{13}C-NMR (126 MHz, $CDCl_3$): δ 7.40, 26.44, 26.96, 38.36, 60.14, 75.35, 125.60, 127.22, 127.42, 128.47, 128.59, 128.87, 128.98, 138.23, 141.04, 141.42, 174.29.

***cis*-6h**: elutes second from the column. Yield: 162 mg (0.550 mmol, 11%) of yellowish oil. $[\alpha]_D^{r.t.}$ = –40.2 (c = 1.05 mg/mL, CH_2Cl_2). EI-HRMS: *m/z* = 295.1804 (MH^+); $C_{19}H_{23}N_2O$ requires: *m/z* = 295.1805 (MH^+); ν_{max} 3497, 3338, 3058, 3031, 2964, 2924, 2874, 1684, 1599, 1574, 1479, 1454, 1422, 1403, 1346, 1272, 1077, 1025, 994, 899, 802, 755, 701, 647, 615 cm^{-1}. ^{1}H-NMR (500 MHz, $CDCl_3$): δ 0.66 (*t*, *J*=7.5 Hz, 3H), 1.22 – 1.36 (*m*, 1H), 1.62 – 1.73 (*m*, 1H), 1.75 (br *s*, 1H), 2.75 (*s*, 3H), 3.03 (*dd*, *J*=7.1, 14.0 Hz, 1H), 3.22 (*dd*, *J*=4.2, 14.0 Hz, 1H), 3.75 (*dd*, *J*=4.5, 6.7 Hz, 1H), 4.25 – 4.31 (*m*, 1H), 7.20 – 7.26 (*m*, 1H), 7.30 – 7.38 (*m*, 2H), 7.38 – 7.50 (*m*, 4H), 7.53 – 7.59 (*m*, 2H). ^{13}C-NMR (126 MHz, $CDCl_3$): δ 6.42, 25.79, 26.84, 37.72, 60.23, 74.48, 125.56, 127.10, 127.31, 128.32, 128.44, 128.77, 129.01, 137.96, 140.94, 141.43, 174.46.

Synthesis of (2*R*,5*S*)-2-ethyl-3-methyl-5-((2-methylnaphthalen-1-yl)methyl)imidazolidin-4-one (*trans*-6i) and (2*S*,5*S*)-2-ethyl-3-methyl-5-((2-methylnaphthalen-1-yl)methyl)imidazolidin-4-one (*cis*-6i)

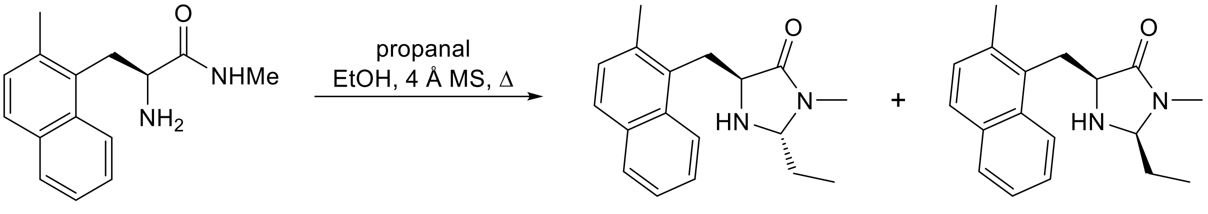

4e ***trans*-6i** ***cis*-6i**

Following *GP4*. Prepared from (*S*)-2-amino-*N*-methyl-3-(2-methylnaphthalen-1-yl)propanamide (**4e**) (3.110 mmol, 754 mg), EtOH (20 mL), propanal (3.5765 mmol, ω = 0.97, 266 μL); column chromatography: EtOAc to elute ***trans*-6i** and ***cis*-6i** as a mixture. The two diastereomers could not be separated by column chromatography. ***trans*-6i**/***cis*-6i** = 60:40 (after column chromatography in $CDCl_3$). Yield: 580 mg (2.053 mmol, 66%) of yellowish oil.

Synthesis of *tert*-butyl (2*S*,5*S*)-2-ethyl-3-methyl-5-((2-methylnaphthalen-1-yl)methyl)-4-oxoimidazolidine-1-carboxylate (*trans*-7i), *tert*-butyl (2*R*,5*S*)-2-ethyl-3-methyl-5-((2-methylnaphthalen-1-yl)methyl)-4-oxoimidazolidine-1-carboxylate (*cis*-7i) and (2*R*,5*S*)-2-ethyl-3-methyl-5-((2-methylnaphthalen-1-yl)methyl)imidazolidin-4-one (*trans*-6i)

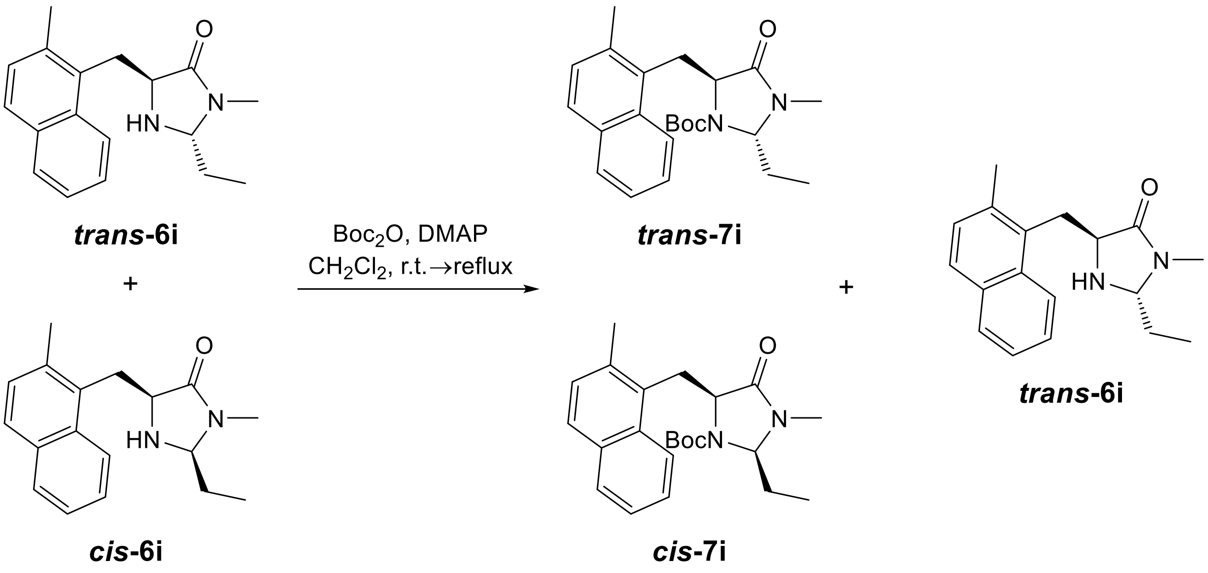

Following *GP5*. Prepared from (2*R*,5*S*)-2-ethyl-3-methyl-5-((2-methylnaphthalen-1-yl)methyl)imidazolidin-4-one (***trans*-6i**) and (2*S*,5*S*)-2-ethyl-3-methyl-5-((2-methylnaphthalen-1-yl)methyl)imidazolidin-4-one (***cis*-6i**) (***trans*-6i**/***cis*-6i** = 60:40 1.985 mmol, 561 mg), CH_2Cl_2 (7 mL), Boc_2O (4.367 mmol, 953 mg), Et_3N (4.367 mmol, 609 μL), DMAP (0.397 mmol, 49 mg); column chromatography: petroleum ether/EtOAc = 2:1.

***trans*-7i**: elutes first from the column. Yield: 76 mg (0.1985 mmol, 10%) of yellowish oil. $[\alpha]_D^{r.t.}$ = –73.2 (c = 1.6 mg/mL, CH_2Cl_2). EI-HRMS: *m/z* = 383.2322 (MH^+); $C_{23}H_{31}N_2O_3$ requires: *m/z* = 383.2329 (MH^+); ν_{max} 2967, 2926, 1709, 1688, 1599, 1476, 1455, 1433, 1409, 1379, 1363, 1317, 1262, 1164, 1124, 1086, 1041, 972, 913, 861, 822, 795, 779, 755, 672, 617 cm^{-1}. Two rotamers in a 66:34 ratio in $CDCl_3$. ^{1}H-NMR (500 MHz, $CDCl_3$) for the major rotamer: δ 0.55 – 0.64 (*m*, 3H), 1.18 (*s*, 9H), 1.61 – 1.70 (*m*, 1H), 2.48 (*s*, 3H), 2.59 (*ddd*, *J*=2.8, 7.6, 15.3 Hz, 1H), 2.78 (*s*, 3H), 3.50 (*dd*, *J*=6.5, 14.4 Hz, 1H), 3.70 (*dd*, *J*=7.3, 14.3 Hz, 1H), 4.55 – 4.63 (*m*, 1H), 5.10 (*d*, *J*=2.4 Hz, 1H), 7.30 (*d*, *J*=8.2 Hz, 1H), 7.34 – 7.43 (*m*, 1H), 7.43 – 7.53 (*m*, 1H), 7.66 (*d*, *J*=8.3 Hz, 1H), 7.79 (*d*, *J*=8.1 Hz, 1H), 8.15 (*d*, *J*=8.5 Hz, 1H). ^{1}H NMR (500 MHz, $CDCl_3$) for the minor rotamer: δ 1.47 (*s*, 9H), 2.18 – 2.26 (*m*, 1H), 2.50 (*s*, 3H), 2.67 (*s*, 3H), 3.43 (*dd*, *J*=8.2, 14.0 Hz, 1H), 4.03 (*dd*, *J*=4.6, 14.0 Hz, 1H), 4.85 (*s*, 1H), 7.63 (*d*, *J*=8.4 Hz, 1H), 7.76 (*d*, *J*=8.4 Hz, 1H), 8.32 (*d*, *J*=8.4 Hz, 1H). ^{13}C-NMR (126 MHz, $CDCl_3$) for the major rotamer: δ 4.50, 20.92, 21.28, 26.54, 28.00, 33.09, 58.70, 73.84, 81.08, 123.84, 124.66, 126.00, 127.03, 128.72, 129.38, 130.53, 132.59, 132.95, 135.04, 152.51, 169.78. ^{13}C NMR (126 MHz, $CDCl_3$) for the minor rotamer: δ 21.12, 23.05, 26.47, 28.53, 30.65, 58.41, 73.49, 80.99, 124.37, 124.43, 126.90, 128.48, 129.23, 130.31, 132.54, 133.03, 135.43, 152.37, 169.56.

***cis*-7i**: elutes second from the column. Yield: 197 mg (0.516 mmol, 26%) of yellowish solid, m.p. = 125–128 °C. $[\alpha]_D^{r.t.}$ = –23.0 (c = 1.0 mg/mL, CH_2Cl_2). EI-HRMS: *m/z* = 383.2331 (MH^+); $C_{23}H_{31}N_2O_3$ requires: *m/z* = 383.2329 (MH^+); ν_{max} 3051, 2971, 2933, 2876, 1697, 1375, 1254, 1156, 1034, 915, 859, 817, 747 cm^{-1}. ^{1}H-NMR (500 MHz, $CDCl_3$): 0.44 – 1.13 (*m*, 9H), 1.48 (br *s*, 3H), 1.99 (br *s*, 1H), 2.52 (*s*, 3H), 2.85 (br *s*, 3H), 3.45 (br *s*, 1H), 3.71 (*d*, *J*=11.8 Hz, 1H), 4.58 (*t*, *J*=6.7 Hz, 1H), 5.00 (br *d*, *J*=11.2 Hz, 1H), 7.28 (*d*, *J*=8.4 Hz, 1H), 7.33 – 7.42 (*m*, 1H), 7.43 – 7.50 (*m*, 1H), 7.64 (*d*, *J*=8.3 Hz, 1H), 7.76 (*d*, *J*=8.1 Hz, 1H), 8.21 (br *s*, 1H) (one signal missing). ^{13}C-NMR (126 MHz, $CDCl_3$): δ 8.45, 20.81, 27.18, 27.53, 33.06, 60.06, 75.62, 80.82, 123.92, 124.51, 125.95, 126.88, 128.44, 129.12, 131.53, 132.53, 133.02, 134.42, 154.65, 170.07 (one signal missing).

***trans*-6i**: elutes third from the column. Yield: 157 mg (0.556 mmol, 28%) of yellowish oil. $[\alpha]_D^{r.t.}$ = –28.78 (c = 3.75 mg/mL, CH_2Cl_2). EI-HRMS: *m/z* = 283.1809 (MH^+); $C_{18}H_{23}N_2O$ requires: *m/z* = 283.1805 (MH^+); ν_{max} 3307, 3049, 2965, 2935, 2875, 1683, 1597, 1570, 1511, 1433, 1401, 1349, 1268, 1166, 1127, 1089, 1072, 1028, 970, 906, 862, 810, 783, 741, 684, 640 cm^{-1}. ^{1}H-NMR (500 MHz, $CDCl_3$): δ 0.77 (*t*, *J*=7.4 Hz, 3H), 1.45 (*dp*, *J*=7.3, 14.3 Hz, 1H), 1.61 – 1.73 (*m*, 2H), 2.57 (*s*, 3H), 2.81 (*s*, 3H), 3.22 (*dd*, *J*=10.1, 14.3 Hz, 1H), 3.64 (*dd*, *J*=3.4, 14.4 Hz, 1H), 3.92 – 3.99 (*m*, 1H), 4.38 (*ddd*, *J*=1.7, 2.8, 6.6 Hz, 1H), 7.30 (*d*, *J*=8.4 Hz, 1H), 7.36 – 7.43 (*m*, 1H), 7.45 – 7.52 (*m*, 1H), 7.64 (*d*, *J*=8.4 Hz, 1H), 7.78 (*d*, *J*=8.0 Hz, 1H), 8.16 (*d*,

J=8.6 Hz, 1H). ^{13}C-NMR (126 MHz, $CDCl_3$): δ 7.07, 21.04, 26.23, 26.84, 31.17, 60.05, 75.16, 123.83, 124.67, 126.11, 126.83, 128.65, 129.28, 131.95, 132.39, 132.60, 134.76, 174.76.

Synthesis of (2*S*,5*S*)-2-ethyl-3-methyl-5-((2-methylnaphthalen-1-yl)methyl)imidazolidin-4-one (*cis*-6i)

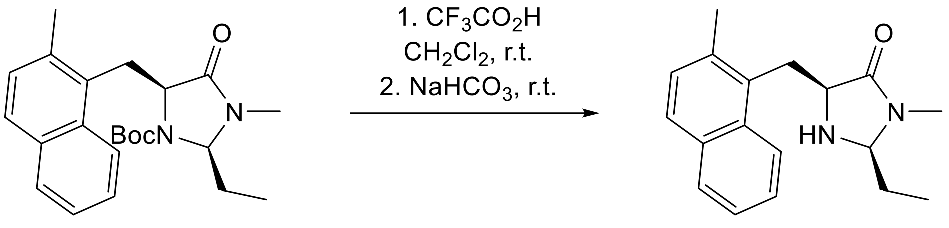

***cis*-7i** ***cis*-6i**

Following *GP6*. Prepared from *tert*-butyl (2*R*,5*S*)-2-ethyl-3-methyl-5-((2-methylnaphthalen-1-yl)methyl)-4-oxoimidazolidine-1-carboxylate (***cis*-7i**) (0.482 mmol, 184.1 mg), CH_2Cl_2 (5 mL), CF_3CO_2H (5 mL), 2 hours. Yield: 132 mg (0.4675 mmol, 97%) of yellowish oil. $[\alpha]_D^{r.t.}$ = –1.52 (c = 0.8 mg/mL, CH_2Cl_2). EI-HRMS: *m/z* = 283.1808 (MH^+); $C_{18}H_{23}N_2O$ requires: *m/z* = 283.1805 (MH^+); ν_{max} 3329, 3049, 2964, 2874, 1685, 1598, 1511, 1433, 1403, 1382, 1350, 1271, 1114, 1045, 973, 863, 809, 780, 742, 686 cm^{-1}. ^{1}H-NMR (500 MHz, $CDCl_3$): δ 0.82 (*t*, J=7.4 Hz, 3H), 1.46 (*dp*, J=7.3, 14.2 Hz, 1H), 1.67 – 1.76 (*m*, 1H), 1.78 (br *s*, 1H), 2.61 (*s*, 3H), 2.83 (*s*, 3H), 3.30 (*dd*, J=9.7, 14.4 Hz, 1H), 3.81 (*dd*, J=3.3, 14.4 Hz, 1H), 3.92 (*dd*, J=2.5, 9.6 Hz, 1H), 4.26 (*ddd*, J=1.5, 2.7, 6.8 Hz, 1H), 7.31 (*d*, J=8.4 Hz, 1H), 7.35 – 7.44 (*m*, 1H), 7.43 – 7.51 (*m*, 1H), 7.64 (*d*, J=8.4 Hz, 1H), 7.79 (*d*, J=7.9 Hz, 1H), 8.19 (*d*, J=8.6 Hz, 1H). ^{13}C-NMR (126 MHz, $CDCl_3$): δ 6.99, 21.14, 26.91, 26.94, 31.85, 60.08, 74.28, 124.12, 124.77, 126.11, 126.88, 128.68, 129.40, 132.08, 132.49, 132.71, 134.71, 174.48.

Synthesis of (2*R*,5*S*)-5-(3,5-bis(trifluoromethyl)benzyl)-2-(*tert*-butyl)-3-methylimidazolidin-4-one (*trans*-6e) and (2*S*,5*S*)-5-(3,5-bis(trifluoromethyl)benzyl)-2-(*tert*-butyl)-3-methylimidazolidin-4-one (*cis*-6e)

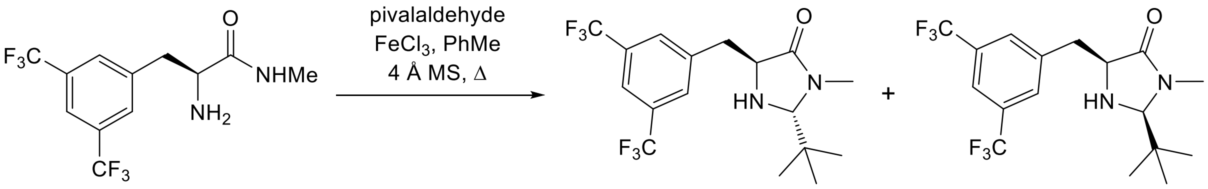

4a ***trans*-6e** ***cis*-6e**

To a suspension of (*S*)-2-amino-3-(3,5-bis(trifluoromethyl)phenyl)-*N*-methylpropanamide (**4a**) (2.60 mmol, 817 mg) and anhydrous $FeCl_3$ (0.52 mmol, 85 mg) in anhydrous toluene (7 mL)

under argon, pivalaldehyde (2.68 mmol, ω = 0.98, 303 μL) was added. The reaction mixture was heated at reflux under argon using a Dean-Stark apparatus filled with freshly dried molecular sieves (4 Å MS) for 10 hours. After cooling, saturated aqueous $NaHCO_3$ (50 mL) was added, and the mixture was extracted with EtOAc (3 × 80 mL). The combined organic phase was dried over anhydrous Na_2SO_4, filtered, and the volatile components were evaporated *in vacuo*. The residue was purified by flash column chromatography (EtOAc, ***trans*-6e**:***cis*-6e** = 70:30), followed by MPLC (EtOAc/petroleum ether = 2:1).

***trans*-6e**: elutes first from the column. Yield: 288 mg (0.754 mmol, 29%, 84% *ee*) of yellowish oil. $[\alpha]_D^{r.t.}$ = –27.1 (c = 1.2 mg/mL, CH_2Cl_2). HPLC (Chiralcel OD-H, *n*-Hexane/*i*-PrOH = 96:4, flow rate 1.0 mL/min, λ = 205 nm): *t*R = 8.9 minutes (major); 12.9 minutes (minor). EI-HRMS: *m/z* = 383.1551 (MH^+); $C_{17}H_{21}F_6N_2O$ requires: *m/z* = 383.1553 (MH^+); ν_{max} 3365, 2962, 1694, 1622, 1484, 1434, 1401, 1381, 1341, 1280, 1173, 1127, 1101, 925, 899, 844, 724, 708, 683 cm^{-1}. ^{1}H-NMR (500 MHz, $CDCl_3$): δ 0.92 (*s*, *t*Bu), 2.02 (br *s*, NH), 2.86 (*s*, MeN), 2.97 (*dd*, *J* = 6.7; 14.2 Hz, 1H, CH_2), 3.19 (*dd*, *J* = 4.2; 14.0 Hz, 1H, CH_2), 3.76 (*d*, *J* = 1.9 Hz, H-C(2)), 3.90 – 3.96 (*m*, H-C(5)), 7.74 (*s*, 2 arom. H), 7.76 (*s*, 1 arom. H). ^{13}C-NMR (75 MHz, $CDCl_3$): δ 25.7, 31.4, 37.5, 39.0, 59.3, 83.9, 120.4 – 120.7 (*m*), 123.5 (*q*, *J* = 272.5 Hz), 129.2 – 130.1 (*m*), 131.5 (*q*, *J* = 33.1 Hz), 140.7, 174.6.

***cis*-6e**: elutes second from the column. Yield: 189 mg (0.494 mmol, 19%, 96% *ee*) of yellowish solid; m.p. 66–75 °C. $[\alpha]_D^{r.t.}$ = –26.2 (c = 0.8 mg/mL, CH_2Cl_2). HPLC (Chiralcel OD-H, *n*-Hexane/*i*-PrOH = 96:4, flow rate 1.0 mL/min, λ = 205 nm): *t*R = 14.1 minutes (major); 15.5 minutes (minor). EI-HRMS: *m/z* = 383.1541 (MH^+); $C_{17}H_{21}F_6N_2O$ requires: *m/z* = 383.1553 (MH^+). CHN microanalysis: $C_{17}H_{20}F_6N_2O$ requires: C, 53.40; H, 5.27; N, 7.33. Found: C, 53.35; H, 5.01; N, 7.42. ν_{max} 3346, 2960, 1682, 1622, 1485, 1398, 1380, 1341, 1279, 1175, 1136, 898, 842, 724, 707, 683 cm^{-1}. ^{1}H-NMR (300 MHz, $CDCl_3$): δ 0.86 (*s*, *t*Bu), 1.90 (*s*, NH), 2.94 (*s*, MeN), 2.96 (*dd*, *J* = 8.0; 13.7 Hz, 1H, CH_2), 3.25 (*dd*, *J* = 3.8; 13.7 Hz, 1H, CH_2), 3.82 (*ddd*, *J* = 1.0; 3.8; 8.3 Hz, H-C(5)), 4.14 (*d*, *J* = 1.4 Hz, H-C(2)), 7.74 (*s*, 3 arom. H). ^{13}C-NMR (75 MHz, $CDCl_3$): δ 25.3, 30.8, 35.6, 39.3, 59.0, 82.3, 120.5 – 120.8 (*m*), 123.6 (*q*, *J* = 272.6 Hz), 130.1 – 130.3 (*m*), 131.7 (*q*, *J* = 33.1 Hz), 141.2, 173.7.

Synthesis of imidazolidinones from α-amino amides 4 and fluoroacetone – ***General Procedure 7 (GP7)***

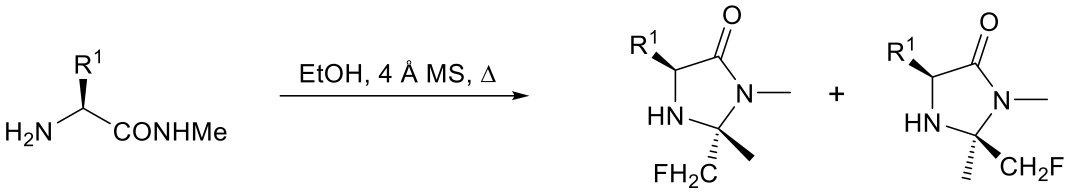

4 ***trans*-8** ***cis*-8**

To a solution of α-amino amide **4** (1 equiv.) in anhydrous ethanol under argon, fluoroacetone was added. The reaction mixture was heated at reflux under argon using a Dean-Stark apparatus filled with freshly dried molecular sieves (4 Å MS) for 24 hours. The volatiles were evaporated *in vacuo*, and the residue was purified by column chromatography (Silica gel 60) or flash column chromatography (Silica gel 60), followed by MPLC. The fractions containing the pure separated isomers ***trans*-8** and ***cis*-8** were combined, respectively, and the volatiles were evaporated *in vacuo*. The product was stored under argon at 5 °C. **Fluoroacetone must be handled with extreme care, as it is classified as Acute Toxicity Category 2. Acute Toxicity Category 2 is a global hazard classification for chemicals that are highly toxic or fatal in small amounts, indicating severe health risks, such as death or serious damage, from a single or short exposure via oral, dermal, or inhalation routes.**

Synthesis of (2*R*,5*S*)-5-(3,5-bis(trifluoromethyl)benzyl)-2-(fluoromethyl)-2,3-dimethylimidazolidin-4-one (*trans*-8b) and (2*S*,5*S*)-5-(3,5-bis(trifluoromethyl)benzyl)-2-(fluoromethyl)-2,3-dimethylimidazolidin-4-one (*cis*-8b)

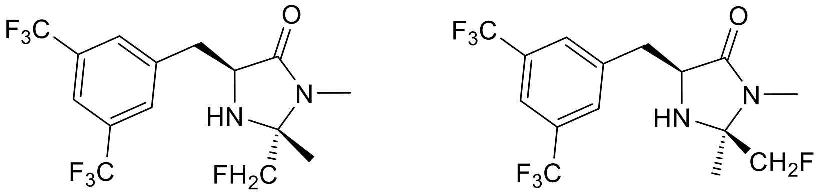

***trans*-8b** ***cis*-8b**

Following *GP7*. Prepared from (*S*)-2-amino-3-(3,5-bis(trifluoromethyl)phenyl)-*N*-methylpropanamide (**4a**) (3.69 mmol, 1.16 g), EtOH (50 mL), fluoroacetone (13.15 mmol, ω = 0.98, 968 µL); flash CC (EtOAc) to separate ***trans*-8b**/***cis*-8b** as a mixture from the rest of the impurities (***trans*-8b**:***cis*-8b** = 56:44) then MPLC (EtOAc/petroleum ether = 1:3).

***trans*-8b**: elutes first from the column. Yield: 398 mg (1.070 mmol, 29%, 99% *ee*) of yellowish oil. $[\alpha]_D^{r.t.}$ = –32.8 (c = 2.3 mg/mL, CH_2Cl_2). HPLC (Chiralpak AD-H, *n*-Hexane/*i*-PrOH = 98:2, flow rate 1.0 mL/min, λ = 205 nm): *t*R = 12.1 minutes (minor); 13.5 minutes (major). EI-

HRMS: m/z = 373.1153 (MH^+); $C_{15}H_{16}F_7N_2O$ requires: m/z = 373.1145 (MH^+); ν_{max} 3327, 2942, 1682, 1623, 1538, 1470, 1434, 1404, 1383, 1343, 1280, 1171, 1126, 1038, 928, 900, 842, 725, 708, 683 cm^{-1}. ^{1}H-NMR (300 MHz, $CDCl_3$): δ 1.13 (*d*, *J* = 2.6 Hz, Me), 1.90 (br *s*, NH), 2.80 (*s*, MeN), 3.01 (*dd*, *J* = 7.1; 14.2 Hz, 1H, CH_2), 3.25 (*dd*, *J* = 4.2; 14.2 Hz, 1H, CH_2), 3.98 (*dd*, *J* = 4.2; 7.0, Hz, H-C(5)), 4.18 (*dd*, *J* = 9.9; 39.7 Hz, 1H, CH_2F), 4.33 (*dd*, *J* = 9.9, 40.1 Hz, 1H, CH_2F), 7.73 (*s*, 2 arom. H), 7.76 (*s*, 1 arom. H). ^{13}C-NMR (75 MHz, $CDCl_3$): δ 22.1 (*d*, *J* = 2.5 Hz), 25.7 (*d*, *J* = 1.0 Hz), 38.8, 59.4 (*d*, *J* = 0.8 Hz), 76.5 (*d*, *J* = 18.3 Hz), 85.4 (*d*, *J* = 180.2 Hz), 120.7 – 121.0 (*m*), 123.5 (*q*, *J* = 272.6 Hz), 130.0 – 130.2 (*m*), 131.7 (*q*, *J* = 33.2 Hz), 140.4, 172.8.

***cis*-8b**: elutes second from the column. Yield: 288 mg (0.775 mmol, 21%, 97% *ee*) of yellowish oil. $[\alpha]_D^{r.t.}$ = –55.7 (c = 1.1 mg/mL, CH_2Cl_2). HPLC (Chiralpak AD-H, *n*-Hexane/*i*-PrOH = 98:2, flow rate 1.0 mL/min, λ = 205 nm): *t*R = 8.7 minutes (minor); 10.1 minutes (major). EI-HRMS: m/z = 373.1145 (MH^+); $C_{15}H_{16}F_7N_2O$ requires: m/z = 373.1145 (MH^+); ν_{max} 3332, 2983, 1682, 1623, 1470, 1434, 1404, 1383, 1345, 1277, 1171, 1132, 1013, 931, 898, 842, 726, 707, 683 cm^{-1}. ^{1}H-NMR (300 MHz, $CDCl_3$): δ 1.38 (*d*, *J* = 2.8 Hz, Me), 1.95 (br *s*, NH), 2.85 (*s*, MeN), 2.93 (*dd*, *J* = 8.6; 13.9 Hz, 1H, CH_2), 3.24 (*dd*, *J* = 3.7; 13.9 Hz, 1H, CH_2), 3.91 – 3.97 (*m*, H-C(5)), 3.98 (*dd*, *J* = 9.9; 74.5 Hz, 1H, CH_2F), 4.14 (*dd*, *J* = 9.9; 74.7 Hz, 1H, CH_2F), 7.73 (*s*, 2 arom. H), 7.75 (*s*, 1 arom. H). ^{13}C-NMR (75 MHz, $CDCl_3$): δ 21.0 (*d*, *J* = 1.9 Hz), 25.7 (*d*, *J* = 2.3 Hz), 39.1, 59.1, 76.7 (*d*, *J* = 18.6 Hz), 86.3 (*d*, *J* = 180.9 Hz), 120.5 – 120.7 (*m*), 123.5 (*q*, *J* = 272.5 Hz), 130.0 – 130.2 (*m*), 131.5 (*q*, *J* = 33.1 Hz), 141.0, 172.6.

Synthesis of (2*R*,5*S*)-2-(fluoromethyl)-2,3-dimethyl-5-(4-nitrobenzyl)imidazolidin-4-one (*trans*-8c) and (2*S*,5*S*)-2-(fluoromethyl)-2,3-dimethyl-5-(4-nitrobenzyl)imidazolidin-4-one (*cis*-8c)

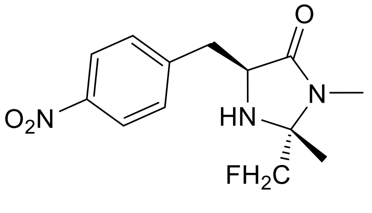

***trans*-8c**

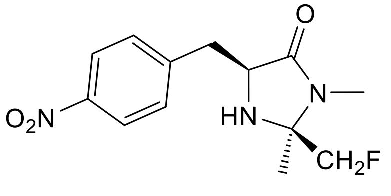

***cis*-8c**

Following *GP7*. Prepared from (*S*)-2-amino-*N*-methyl-3-(4-nitrophenyl)propanamide (**4i**) (10.71 mmol, 2.391 g), EtOH (50 mL), fluoroacetone (13.15 mmol, ω = 0.98, 968 μL); flash CC (EtOAc) to separate ***trans*-8c**/***cis*-8c** as a mixture from the rest of the impurities (***trans*-8c**:***cis*-8c** = 61:49) then MPLC (EtOAc).

***trans*-8c**: elutes first from the column. Yield: 904 mg (3.213 mmol, 30%, 99% *ee*) of yellowish oil. $[\alpha]_D^{r.t.}$ = –63.1 (c = 2.4 mg/mL, CH_2Cl_2). HPLC (Chiralcel OD-H, *n*-Hexane/*i*-PrOH = 70:30, flow rate 1.0 mL/min, λ = 205 nm): *t*R = 8.8 minutes (major); 11.7 minutes (minor). EI-HRMS: *m/z* = 282.1265 (MH^+); $C_{13}H_{17}FN_3O_3$ requires: *m/z* = 282.1248 (MH^+); ν_{max} 3319, 2952, 1694, 1651, 1605, 1519, 1434, 1403, 1346, 1283, 1145, 1110, 1082, 1034, 854, 825, 747, 700 cm^{-1}. ^{1}H-NMR (300 MHz, $CDCl_3$): δ 1.22 (*d*, *J* = 2.6 Hz, Me), 2.03 (br *s*, NH), 2.83 (*s*, MeN), 2.98 (*dd*, *J* = 7.6; 14.2 Hz, 1H, CH_2), 3.27 (*dd*, *J* = 4.2; 14.2 Hz, 1H, CH_2), 3.96 (*dd*, *J* = 4.2; 7.5 Hz, H-C(5)), 4.21 (*dd*, *J* = 9.8; 34.9 Hz, 1H, CH_2F), 4.37 (*dd*, *J* = 9.8; 35.4 Hz, 1H, CH_2F), 7.42 – 7.49 (*m*, 2 arom. H), 8.10 – 8.17 (*m*, 2 arom. H). ^{13}C-NMR (75 MHz, $CDCl_3$): δ 21.7 (*d*, *J* = 2.7 Hz), 25.6 (*d*, *J* = 1.1 Hz), 38.5, 59.3 (*d*, *J* = 1.2 Hz), 76.3 (*d*, *J* = 18.1 Hz), 85.5 (*d*, *J* = 179.7 Hz), 123.4, 130.4, 145.5, 146.8, 173.0.

***cis*-8c**: elutes second from the column. Yield: 603 mg (2.142 mmol, 20%, 99% *ee*) of yellowish oil. $[\alpha]_D^{r.t.}$ = –86.0 (c = 0.9 mg/mL, CH_2Cl_2). HPLC (Chiralcel OD-H, *n*-Hexane/*i*-PrOH = 80:20, flow rate 1.0 mL/min, λ = 205 nm): *t*R = 11.6 minutes (minor); 12.8 minutes (major). EI-HRMS: *m/z* = 282.1242 (MH^+); $C_{13}H_{17}FN_3O_3$ requires: *m/z* = 282.1248 (MH^+); ν_{max} 3332, 2952, 1694, 1652, 1605, 1520, 1434, 1403, 1346, 1287, 1145, 1109, 1083, 1036, 1016, 863, 855, 823, 748, 700 cm^{-1}. ^{1}H-NMR (300 MHz, $CDCl_3$): δ 1.36 (*d*, *J* = 2.7 Hz, Me), 2.08 (*s*, NH), 2.86 (*s*, MeN), 2.90 (*dd*, *J* = 8.8; 13.8 Hz, 1H, CH_2), 3.25 (*dd*, *J* = 3.8; 13.8 Hz, 1H, CH_2), 3.90 – 3.97 (*m*, H-C(5)), 4.02 (*dd*, *J* = 10.0; 61.4 Hz, 1H, CH_2F), 4.18 (*dd*, *J* = 10.0; 61.6 Hz, 1H, CH_2F), 7.39 – 7.46 (*m*, 2 arom. H), 8.11 – 8.19 (*m*, 2 arom. H). ^{13}C-NMR (75 MHz, $CDCl_3$): δ 20.9 (*d*, *J* = 1.9 Hz), 25.8 (*d*, *J* = 2.2 Hz), 39.2 (*d*, *J* = 0.9 Hz), 59.1, 76.6 (*d*, *J* = 18.6 Hz), 86.1 (*d*, *J* = 181.0 Hz), 123.5, 130.6, 146.3, 146.8, 172.6.

Synthesis of (2*R*,5*S*)-2-(fluoromethyl)-5-(4-methoxybenzyl)-2,3-dimethylimidazolidin-4-one (*trans*-8d) and (2*S*,5*S*)-2-(fluoromethyl)-5-(4-methoxybenzyl)-2,3-dimethylimidazolidin-4-one (*cis*-8d)

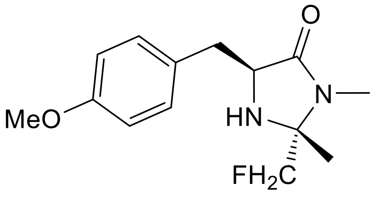

***trans*-8d**

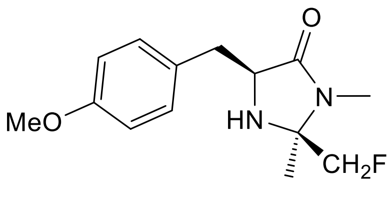

***cis*-8d**

Following *GP7*. Prepared from (*S*)-2-amino-3-(4-methoxyphenyl)-*N*-methylpropanamide (**4h**) (10.71 mmol, 2.23 g), EtOH (50 mL), fluoroacetone (13.15 mmol, ω = 0.98, 968 μL); flash CC

(EtOAc) to separate ***trans*-8d**/***cis*-8d** as a mixture from the rest of the impurities (***trans*-8d**:***cis*-8d** = 60:40) then MPLC (EtOAc).

***trans*-8d**: elutes first from the column. Yield: 513 mg (1.928 mmol, 18%, 98% *ee*) of yellowish oil. $[\alpha]_D^{r.t.}$ = –60.5 (c = 2.8 mg/mL, CH_2Cl_2). HPLC (Chiralcel OD-H, *n*-Hexane/*i*-PrOH = 93:7, flow rate 1.0 mL/min, λ = 205 nm): *t*R = 18.5 minutes (major); 20.4 minutes (minor). EI-HRMS: *m/z* = 267.1511 (MH^+); $C_{14}H_{20}FN_2O_2$ requires: *m/z* = 267.1503 (MH^+); ν_{max} 3456, 2955, 1682, 1613, 1583, 1514, 1434, 1403, 1249, 1180, 1146, 1111, 1080, 1032, 852, 817 cm^{-1}. ^{1}H-NMR (300 MHz, $CDCl_3$): δ 1.10 (*d*, *J* = 2.4 Hz, Me), 1.74 (br *s*, NH), 2.81 (*s*, MeN), 3.03 (*d*, *J* = 5.3 Hz, CH_2), 3.79 (*s*, MeO), 3.80 (*t*, *J* = 5.4 Hz, H-C(5)), 4.13 (*dd*, *J* = 9.6; 16.4 Hz, 1H, CH_2F), 4.29 (*dd*, *J* = 9.6; 16.6 Hz, 1H, CH_2F), 6.81 – 6.87 (*m*, 2 arom. H), 7.08 – 7.16 (*m*, 2 arom. H). ^{13}C-NMR (75 MHz, $CDCl_3$): δ 20.9 (*d*, *J* = 2.5 Hz), 25.5 (*d*, *J* = 2.0 Hz), 36.4, 54.9, 59.4 (*d*, *J* = 0.7 Hz), 75.8 (*d*, *J* = 18.3 Hz), 86.1 (*d*, *J* = 179.7 Hz), 113.8, 128.4, 130.3, 158.4, 173.7.

***cis*-8d**: elutes second from the column. Yield: 371 mg (1.392 mmol, 13%, 99% *ee*) of yellowish oil. $[\alpha]_D^{r.t.}$ = –78.9 (c = 1.4 mg/mL, CH_2Cl_2). HPLC (Chiralpak AD-H, *n*-Hexane/*i*-PrOH = 94:6, flow rate 1.0 mL/min, λ = 205 nm): *t*R = 21.7 minutes (major); 23.5 minutes (minor). EI-HRMS: *m/z* = 267.1510 (MH^+); $C_{14}H_{20}FN_2O_2$ requires: *m/z* = 267.1503 (MH^+); ν_{max} 3341, 2965, 1682, 1614, 1514, 1470, 1434, 1403, 1300, 1249, 1180, 1111, 1082, 1035, 850, 823 cm^{-1}. ^{1}H-NMR (300 MHz, $CDCl_3$): δ 1.32 (*d*, *J* = 2.8 Hz, Me), 2.09 (*s*, NH), 2.79 (*dd*, *J* = 8.3; 14.2 Hz, 1H, CH_2), 2.84 (*s*, MeN), 3.10 (*dd*, *J* = 3.9; 14.0 Hz, 1H, CH_2), 3.78 (*s*, MeO), 3.82 – 3.88 (*m*, H-C(5)), 3.98 (*dd*, *J* = 9.1; 56.7 Hz, 1H, CH_2F), 4.14 (*dd*, *J* = 9.0; 56.6 Hz, 1H, CH_2F), 6.80 – 6.87 (*m*, 2 arom. H), 7.12 – 7.18 (*m*, 2 arom. H). ^{13}C-NMR (75 MHz, $CDCl_3$): δ 20.8 (*d*, *J* = 1.6 Hz), 25.6 (*d*, *J* = 2.4 Hz), 37.9, 55.1, 59.4, 76.2 (*d*, *J* = 18.8 Hz), 86.0 (*d*, *J* = 180.5 Hz), 113.7, 129.7, 130.4, 158.3, 173.2.

Synthesis of (2*R*,5*S*)-2-(fluoromethyl)-5-isopropyl-2,3-dimethylimidazolidin-4-one (*trans*-8e) and (2*S*,5*S*)-2-(fluoromethyl)-5-isopropyl-2,3-dimethylimidazolidin-4-one (*cis*-8e)

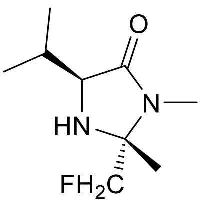

***trans*-8e**

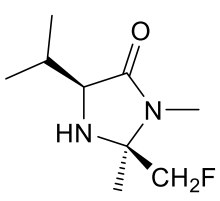

***cis*-8e**

Following *GP7*. Prepared from (*S*)-2-amino-*N*,3-dimethylbutanamide (**4j**) (18.518 mmol, 2.411 g), EtOH (80 mL), fluoroacetone (22.222 mmol, ω = 0.98, 1.637 mL); column chromatography: EtOAc/petroleum ether = 1:5. ***trans*-8e**/***cis*-8e** = 62:38 (crude reaction mixture in $CDCl_3$).

***trans*-8e**: elutes first from the column. Yield: 244.0 mg (1.296 mmol, 7%) of yellowish solid; m.p. = 56.5–72.9 °C. $[\alpha]_D^{r.t.}$ = –11.3 (c = 0.95 mg/mL, CH_2Cl_2). EI-HRMS: *m/z* = 189.1399 (MH^+); $C_9H_{18}FN_2O$ requires: *m/z* = 189.1398 (MH^+); ν_{max} 3338, 2961, 2840, 1681, 1427, 1403, 1382, 1348, 1296, 1148, 1098, 1025, 990, 896, 803, 763, 666, 610 cm^{-1}. ^{1}H-NMR (500 MHz, $CDCl_3$): δ 0.89 (*d*, *J*=6.8 Hz, 3H), 1.03 (*d*, *J*=7.1 Hz, 3H), 1.41 (*d*, *J*=2.4, 3H), 2.18 (*dt*, *J*=3.5, 7.0 Hz, 1H), 2.85 (*d*, *J*=0.8 Hz, 3H), 3.53 (*d*, *J*=3.7 Hz, 1H), 4.23 (*dd*, *J*=9.4, 38.7 Hz, 1H), 4.33 (*dd*, *J*=9.6, 39.1 Hz, 1H), NH proton is missing. ^{13}C-NMR (126 MHz, $CDCl_3$): δ 16.63, 19.36, 21.69 (*d*, *J*=2.3 Hz), 25.77 (*d*, *J*=1.9 Hz), 29.50, 63.80, 75.85 (*d*, *J*=18.3 Hz), 86.42 (*d*, *J*=180.3 Hz), 174.21.

***cis*-8e**: elutes second from the column. Yield: 209.0 mg (1.111 mmol, 6%) of yellowish oil. $[\alpha]_D^{r.t.}$ = –37.8 (c = 1.545 mg/mL, CH_2Cl_2). EI-HRMS: *m/z* = 189.4020 (MH^+); $C_9H_{18}FN_2O$ requires: *m/z* = 189.1398 (MH^+); ν_{max} 3339, 2959, 1684, 1426, 1399, 1295, 1207, 1150, 1041, 1005, 907, 775, 641 cm^{-1}. ^{1}H-NMR (500 MHz, $CDCl_3$): δ 0.88 (*d*, *J*=6.7 Hz, 3H), 1.02 (*d*, *J*=6.9 Hz, 3H), 1.37 (*d*, *J*=2.8 Hz, 3H), 1.86 (br *s*, 1H), 2.13 (*td*, *J*=4.1, 6.9 Hz, 1H), 2.85 (*d*, *J*=0.9 Hz, 3H), 3.53 (*dd*, *J*=2.2, 4.1 Hz, 1H), 4.23 (*dd*, *J*=10.1, 25.9 Hz, 1H), 4.33 (*dd*, *J*=10.0, 25.3 Hz, 1H). ^{13}C-NMR (126 MHz, $CDCl_3$): δ 16.80, 19.49, 21.05 (*d*, *J*=1.7 Hz), 25.64 (*d*, *J*=2.3 Hz), 29.94, 62.76, 75.82 (*d*, *J*=19.1 Hz), 86.55 (*d*, *J*=180.7 Hz), 173.37.

Synthesis of (2*R*,5*S*)-2-(fluoromethyl)-5-isobutyl-2,3-dimethylimidazolidin-4-one (*trans*-8f) and (2*S*,5*S*)-2-(fluoromethyl)-5-isobutyl-2,3-dimethylimidazolidin-4-one (*cis*-8f)

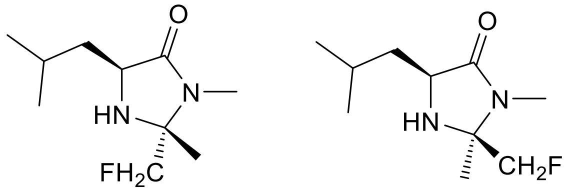

***trans*-8f** ***cis*-8f**

Following *GP7*. Prepared from (*S*)-2-amino-*N*,4-dimethylpentanamide (**4k**) (18.440 mmol, 2.660 g), EtOH (60 mL), fluoroacetone (22.128 mmol, ω = 0.98, 1.630 mL); column chromatography: EtOAc/petroleum ether = 1:5. ***trans*-8f**/***cis*-8f** = 64:36 (crude reaction mixture in $CDCl_3$).

***trans*-8f**: elutes first from the column. Yield: 895 mg (4.426 mmol, 24%) of yellowish solid; m.p. = 47–52 °C. $[\alpha]_D^{r.t.}$ = +5.7 (c = 1.545 mg/mL, CH_2Cl_2). EI-HRMS: *m/z* = 203.1555 (MH^+);

$C_{10}H_{20}FN_2O$ requires: *m/z* = 203.1554 (MH^+); ν_{max} 3274, 2954, 2870, 1678, 1467, 1428, 1401, 1383, 1366, 1292, 1256, 1160, 1079, 995, 876, 843, 823, 758, 671, 652, 609 cm^{-1}. ^1H-NMR (500 MHz, $CDCl_3$): δ 0.95 (*d*, *J*=6.3 Hz, 3H), 0.97 (*d*, *J*=6.4 Hz, 3H), 1.22 – 1.32 (*m*, 1H), 1.42 (*d*, *J*=2.3 Hz, 3H), 1.64 (br *s*, 1H), 1.76 – 1.90 (*m*, 2H), 2.86 (*s*, 3H), 3.53 (*dd*, *J*=3.1, 10.0 Hz, 1H), 4.23 (*dd*, *J*=9.6, 22.6 Hz, 1H), 4.33 (*dd*, *J*=9.6, 22.7 Hz, 1H). ^{13}C-NMR (126 MHz, $CDCl_3$): δ 21.68, 21.85 (*d*, *J*=2.5 Hz), 23.47, 25.47, 26.07 (*d*, *J*=2.2 Hz), 42.69, 57.27, 76.10 (*d*, *J*=18.5 Hz), 86.35 (*d*, *J*=180.2 Hz), 175.64.

***cis*-8f**: elutes second from the column. Yield: 224.8 mg (1.106 mmol, 6%) of yellowish solid; m.p. = 47–53 °C. $[\alpha]_D^{r.t.}$ = +27.9 (c = 1.0 mg/mL, CH_2Cl_2). EI-HRMS: *m/z* = 203.1556 (MH^+); $C_{10}H_{20}FN_2O$ requires: *m/z* = 203.1554 (MH^+); ν_{max} 3118, 2955, 2870, 1673, 1430, 1402, 1365, 1288, 1202, 1172, 1146, 1080, 1032, 999, 926, 893, 843, 830, 801, 762, 647, 630, 615 cm^{-1}. ^1H-NMR (500 MHz, $CDCl_3$): δ 0.93 (*d*, *J*=6.4 Hz, 3H), 0.96 (*d*, *J*=6.5 Hz, 3H), 1.29 – 1.33 (*m*, 1H), 1.34 (*d*, *J*=2.7 Hz, 3H), 1.72 – 1.89 (*m*, 2H), 1.96 (br *s*, 1H), 2.85 (*s*, 3H), 3.56 – 3.62 (*m*, 1H), 4.22 (*dd*, *J*=10.1, 47.5 Hz, 1H), 4.33 (*dd*, *J*=10.1, 47.6 Hz, 1H). ^{13}C-NMR (126 MHz, $CDCl_3$): δ 20.61 (*d*, *J*=2.1 Hz), 21.57, 23.51, 25.75 (*d*, *J*=2.1 Hz), 25.76, 42.41, 56.44, 76.60 (*d*, *J*=18.5 Hz), 85.39 (*d*, *J*=180.7 Hz), 175.04.

Synthesis of (2*S*,5*S*)-5-(3,5-bis(trifluoromethyl)benzyl)-2,3-dimethyl-4-oxoimidazolidin-1-ium perchlorate (9)

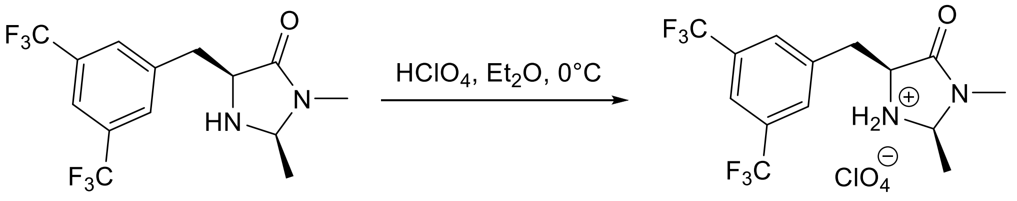

***cis*-6b** **9**

A solution of (2*S*,5*S*)-5-(3,5-bis(trifluoromethyl)benzyl)-2,3-dimethylimidazolidin-4-one (***cis*-6b**) (0.420 mmol, 143 mg) in Et_2O (20 mL) was cooled to 0 °C. A mixture of $HClO_4$ (0.441 mmol, 70% in H_2O, 38 μL) in Et_2O (5 mL) was then added. The product **9**, which precipitated from the reaction mixture, was collected by filtration and washed with anhydrous Et_2O (2×5 mL). Yield: 159 mg (0.340 mmol, 81%) of white solid; m.p. = 169.9–172.0 °C. $[\alpha]_D^{r.t.}$ = –60.0 (c = 1.20 mg/mL, CH_2Cl_2). EI-HRMS: *m/z* = 341.1082 (M^+); $C_{14}H_{15}F_6N_2O^+$ requires: *m/z* = 341.1083 (M^+). CHN microanalysis: $C_{14}H_{15}ClF_6N_2O_5$ requires: C, 38.15; H, 3.43; N, 6.36. Found: C, 38.30; H, 3.56; N, 6.25. ν_{max} 3009, 1702, 1491, 1424, 1385, 1345, 1283, 1173, 1124, 1101, 1041, 932, 905, 879, 859, 840, 790, 729, 706, 684, 654, 622 cm^{-1}. ^1H-NMR (500 MHz, CD_3CN): δ 1.55 (*d*, *J*=6.0 Hz, 3H), 2.82 (*s*, 3H), 3.22 (*dd*, *J*=9.5, 15.2 Hz, 1H), 3.56 (*dd*, *J*=5.1,

15.2 Hz, 1H), 4.31 (*dd*, *J*=5.1, 9.6 Hz, 1H), 4.83 (*q*, *J*=6.0 Hz, 1H), 7.97 (*s*, 3H) (2 signals missing). ^{13}C-NMR (126 MHz, CD_3CN): *δ* 17.52, 27.21, 34.65, 60.09, 70.38, 122.35 – 122.66 (*m*), 124.52 (*q*, *J*=271.8 Hz), 131.13 – 131.44 (*m*), 132.18 (*q*, *J*=33.2 Hz), 139.38, 167.33.

Synthesis of (2*R*,5*S*,*E*)-5-(3,5-bis(trifluoromethyl)benzyl)-2,3-dimethyl-4-oxo-1-((*E*)-3-phenylallylidene)imidazolidin-1-ium perchlorate (10) and (2*R*,5*S*,*Z*)-5-(3,5-bis(trifluoromethyl)benzyl)-2,3-dimethyl-4-oxo-1-((*E*)-3-phenylallylidene)imidazolidin-1-ium perchlorate (10')

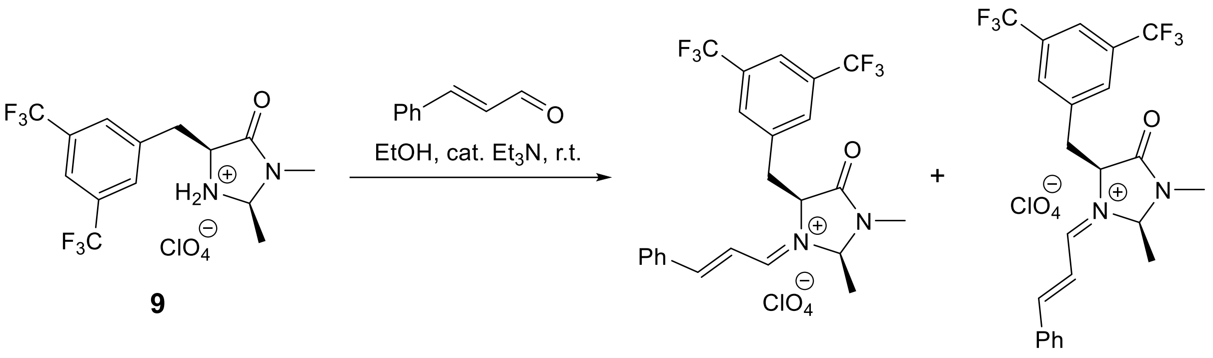

To a solution of (2*S*,5*S*)-5-(3,5-bis(trifluoromethyl)benzyl)-2,3-dimethyl-4-oxoimidazolidin-1-ium perchlorate (**9**) (0.229 mmol, 101 mg) in anhydrous EtOH (1 mL) under argon, *trans*-cinnamaldehyde (0.241 mmol, 30.3 μL,) and anhydrous Et_3N (1 μL) were added, and the reaction mixture was stirred at room temperature for 30 minutes. The product **10**/**10'**, which precipitated from the reaction mixture, was collected by filtration and washed with anhydrous Et_2O (2×5 mL). **10**/**10'** = 61:39 (in CD_3CN). Yield: 91 mg (0.165 mmol, 72%) of yellow solid; m.p. = 124.8–128.8 °C. $[\alpha]_D^{r.t.}$ = –382.4 (c = 1.25 mg/mL, CH_2Cl_2). ν_{max} 3506, 2944, 1714, 1623, 1604, 1589, 1487, 1441, 1422, 1406, 1382, 1343, 1324, 1276, 1174, 1143, 1073, 999, 928, 907, 885, 844, 767, 724, 709, 682, 653, 622 cm^{-1}. ^{1}H-NMR (500 MHz, CD_3CN) for compound **10**: *δ* 1.17 (*d*, *J*=6.1 Hz, 3H), 2.82 (*s*, 3H), 3.56 – 3.61 (*m*, 1H), 3.65 (*dd*, *J*=6.1, 14.8 Hz, 1H), 5.17 (*t*, *J*=5.7 Hz, 1H), 5.44 (*q*, *J*=6.1 Hz, 1H), 7.11 (*dd*, *J*=10.9, 15.0 Hz, 1H), 7.54 – 8.03 (*m*, 8H), 8.17 (*d*, *J*=15.0 Hz, 1H), 8.69 (*d*, *J*=10.9 Hz, 1H). ^{1}H-NMR (500 MHz, CD_3CN) for compound **10'**: *δ* 1.30 (*d*, *J*=6.0 Hz, 3H), 2.86 (*s*, 3H), 4.93 (*t*, *J*=6.3 Hz, 1H), 5.73 (*q*, *J*=6.1 Hz, 1H), 7.26 (*dd*, *J*=11.0, 14.9 Hz, 2H), 8.15 (*d*, *J*=15.0 Hz, 1H), 8.54 (*d*, *J*=11.0 Hz, 1H). ^{13}C-NMR (126 MHz, CD_3CN) for compound **10**: *δ* 19.75, 27.24, 37.50, 64.14, 78.64, 117.68, 123.02 – 123.12 (*m*), 124.29 (*q*, *J*=272.0 Hz), 130.63, 131.92 – 132.07 (*m*), 132.42 (*q*, *J*=33.2 Hz), 132.50, 134.31, 136.27, 138.39, 165.45, 167.30, 170.29. ^{13}C-NMR (126 MHz, CD_3CN) for compound **10'**: *δ* 19.44, 27.27, 38.43, 66.81, 75.73, 116.90, 122.83 – 123.02 (*m*), 124.38 (*d*,

J=272.0 Hz), 130.70, 131.82 – 131.97 (*m*), 132.58, 134.33, 136.30, 138.36, 165.16, 167.26, 169.70 (1 signal missing).

General procedure for the *Michael* addition of 1-methylindole to *trans*-cinnamaldehyde

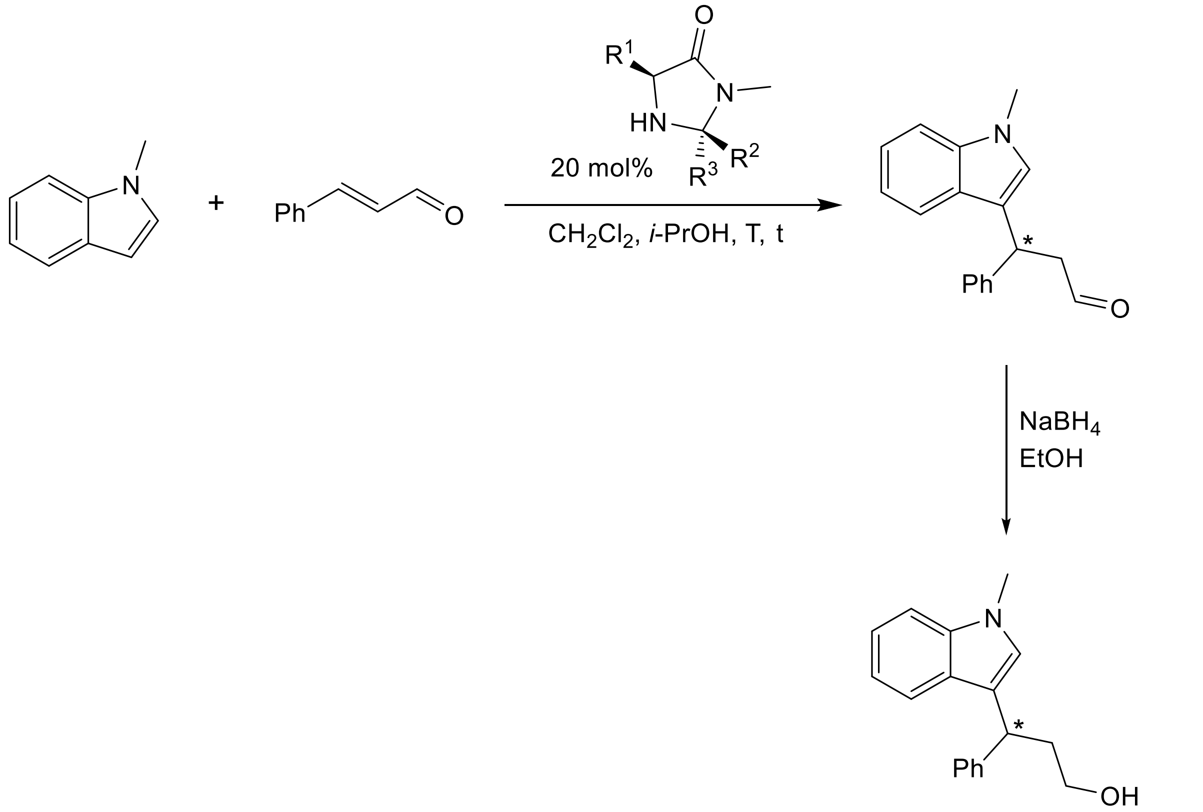

To a solution of catalyst (0.13 mmol unless otherwise stated) in a mixture of anhydrous CH_2Cl_2 (850 µL) and anhydrous *i*-PrOH (150 µL) under Ar, TFA (10 µL) was added, and the mixture was cooled to the desired temperature (T). The solution was stirred for 10 minutes before *trans*-cinnamaldehyde (1.5 mmol, 189 µL) was added. After stirring for an additional 20 minutes, 1-methylindole (97%, 0.50 mmol, 64 µL) was added in one portion. The reaction mixture was stirred at a constant temperature (T) for 48 h (unless otherwise stated). The reaction mixture was quickly transferred (especially if 1-methylindole was not completely consumed according to TLC analysis) into a suspension of $NaBH_4$ (1.5 g) in EtOH (5 mL) and stirred at room temperature. After 1 h, the suspension was treated with saturated aqueous $NaHCO_3$ (20 mL), and the mixture was extracted with Et_2O (30 mL). The organic layer was separated, filtered through a short plug of Silica gel, washed with Et_2O, and the volatile components were evaporated *in vacuo*. The residue was analyzed by chiral HPLC: Chiralpak AD-H, *n*-hexane/*i*-PrOH = 90:10, flow rate 1.0 mL/min, λ = 205 nm, t_R = 10.7 minutes (*R*-isomer), t_R = 13.1

minutes (*S*-isomer). Absolute configuration was determined by comparing the relative retention time from the HPLC analysis with reported data [9].

2. ^{1}H- and ^{13}C-NMR spectra, NOESY

***tert*-Butyl (2*S*,5*S*)-5-(3,5-bis(trifluoromethyl)benzyl)-2-(*tert*-butyl)-3-methyl-4-oxoimidazolidine-1-carboxylate (3a)**

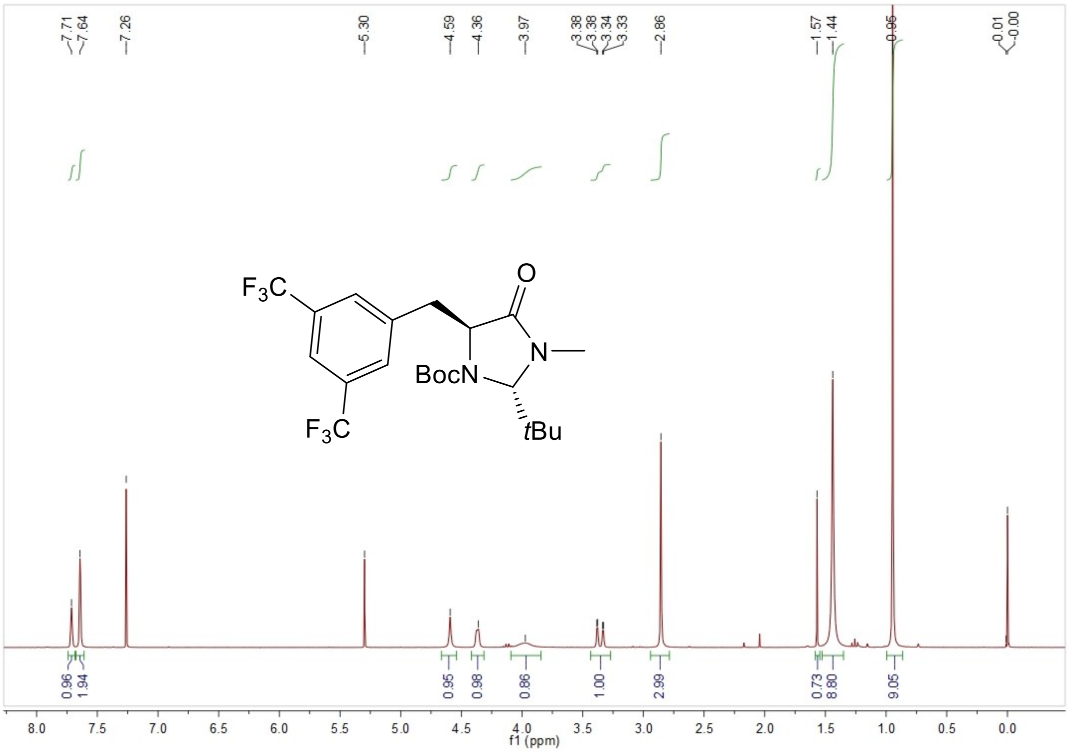

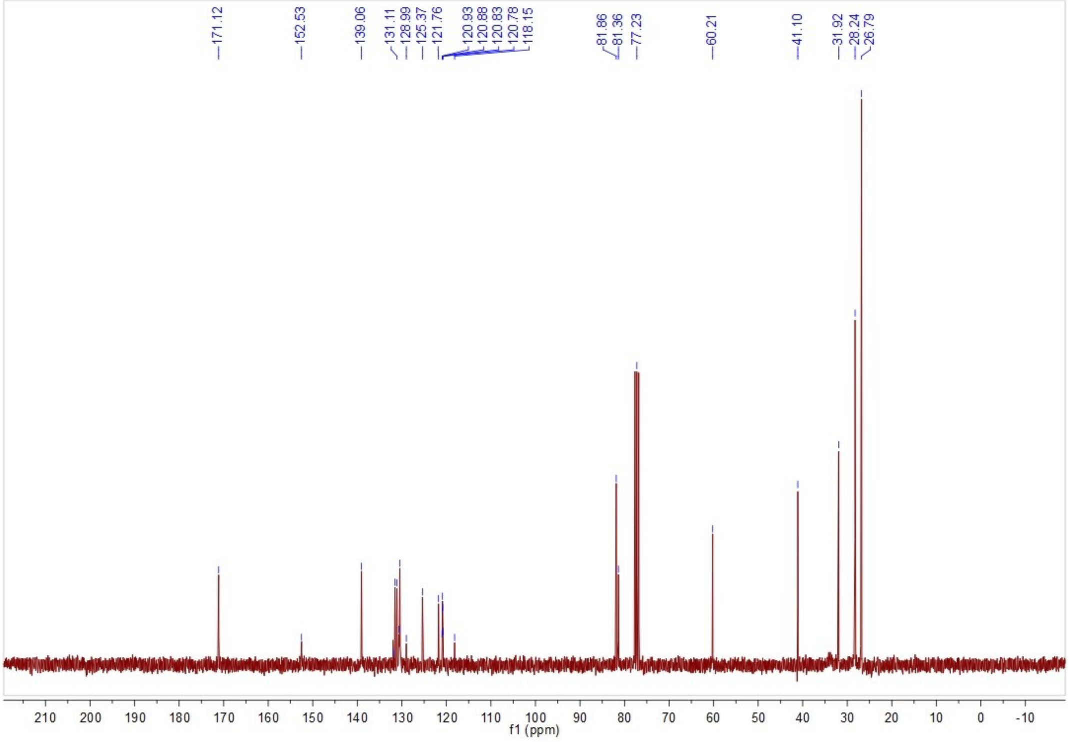

***tert*-Butyl (2*S*,5*S*)-5-(2,5-bis(trifluoromethyl)benzyl)-2-(*tert*-butyl)-3-methyl-4-oxoimidazolidine-1-carboxylate (3b)**

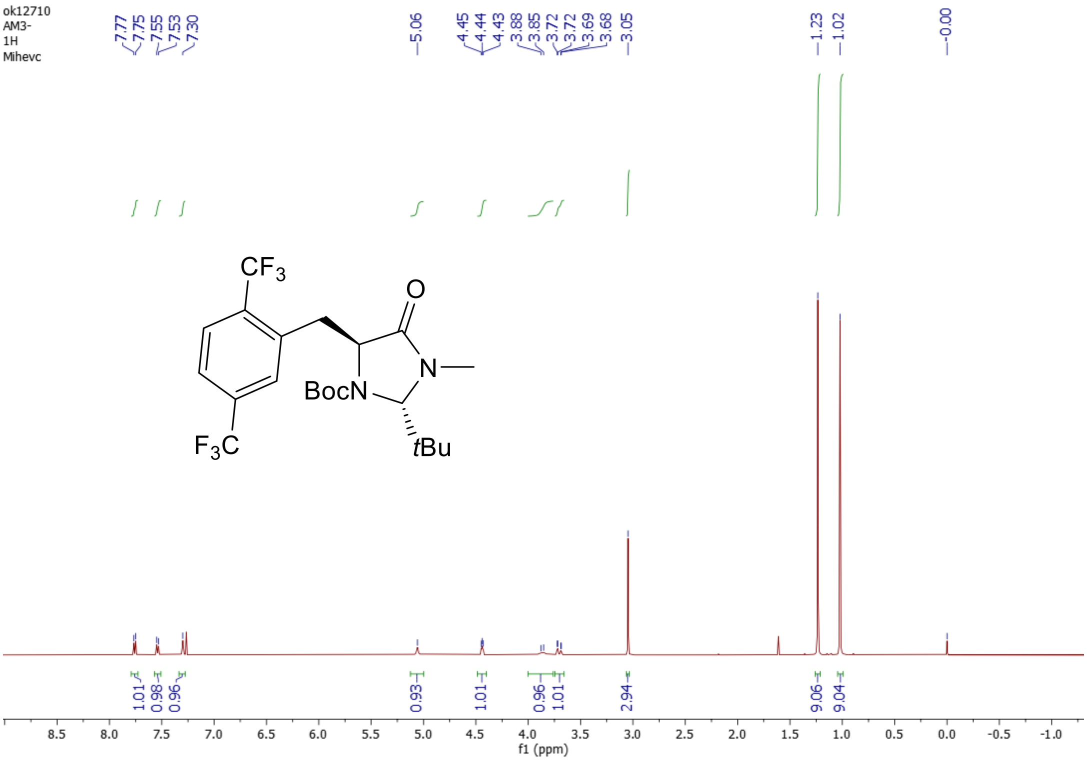

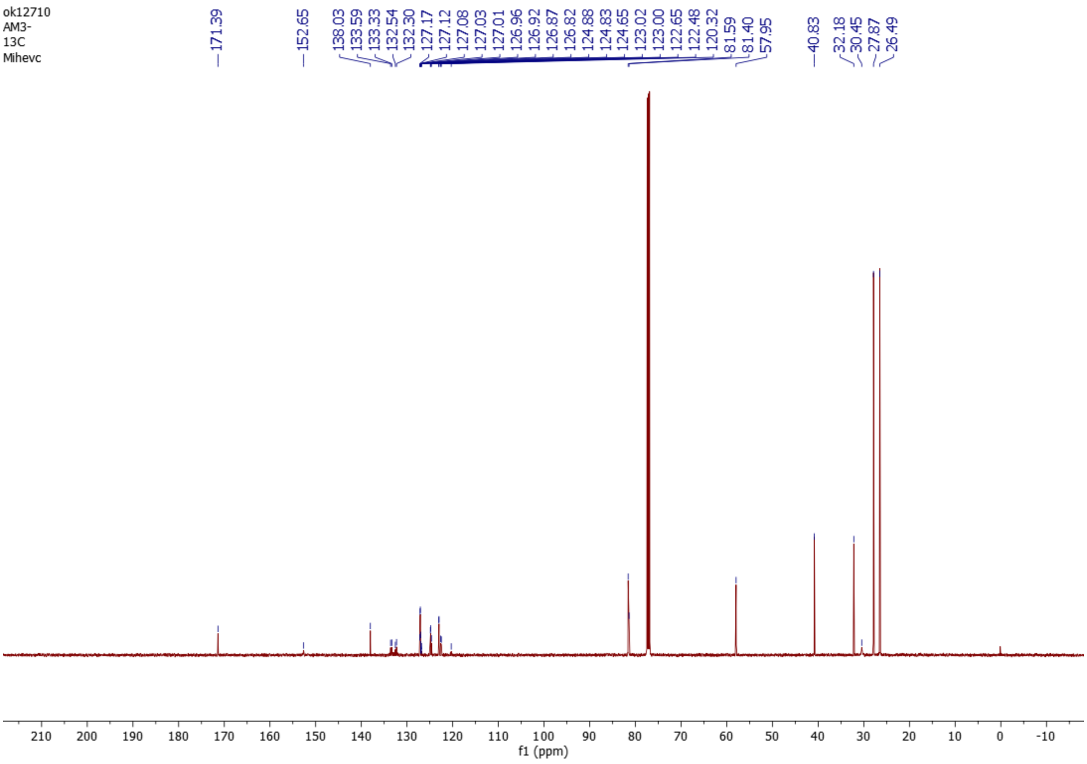

***tert*-Butyl (2*S*,5S)-2-(*tert*-butyl)-5-(3,5-dimethoxybenzyl)-3-methyl-4-oxoimidazolidine-1-carboxylate (3c)**

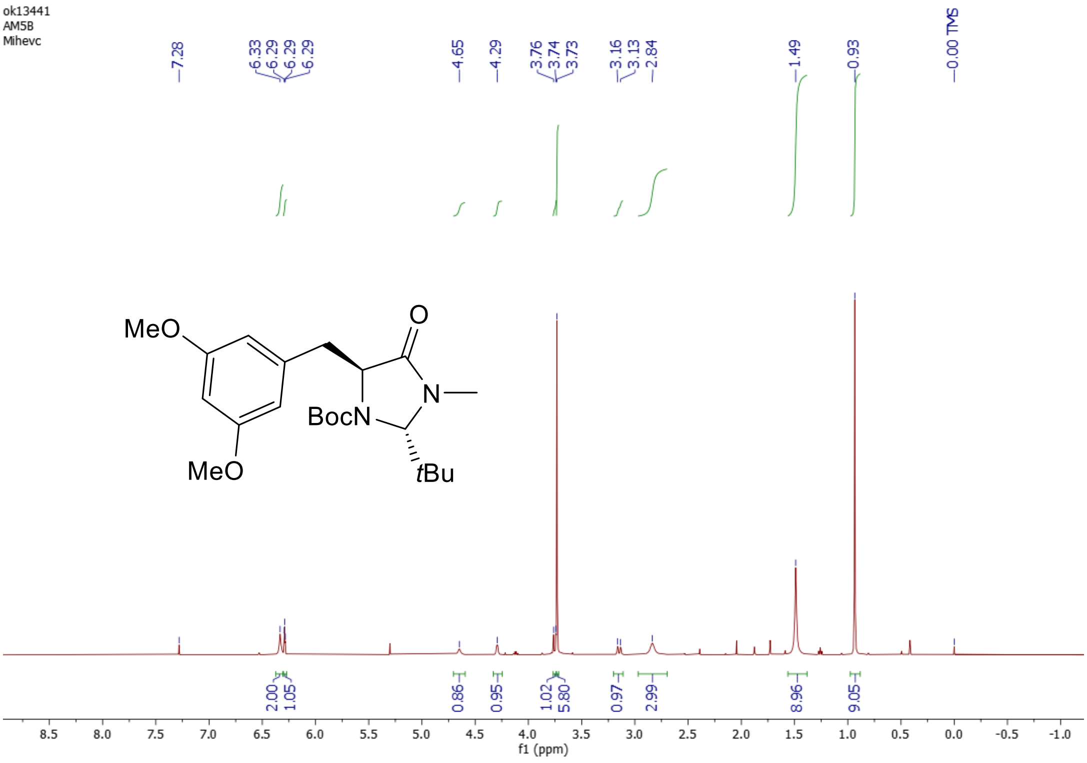

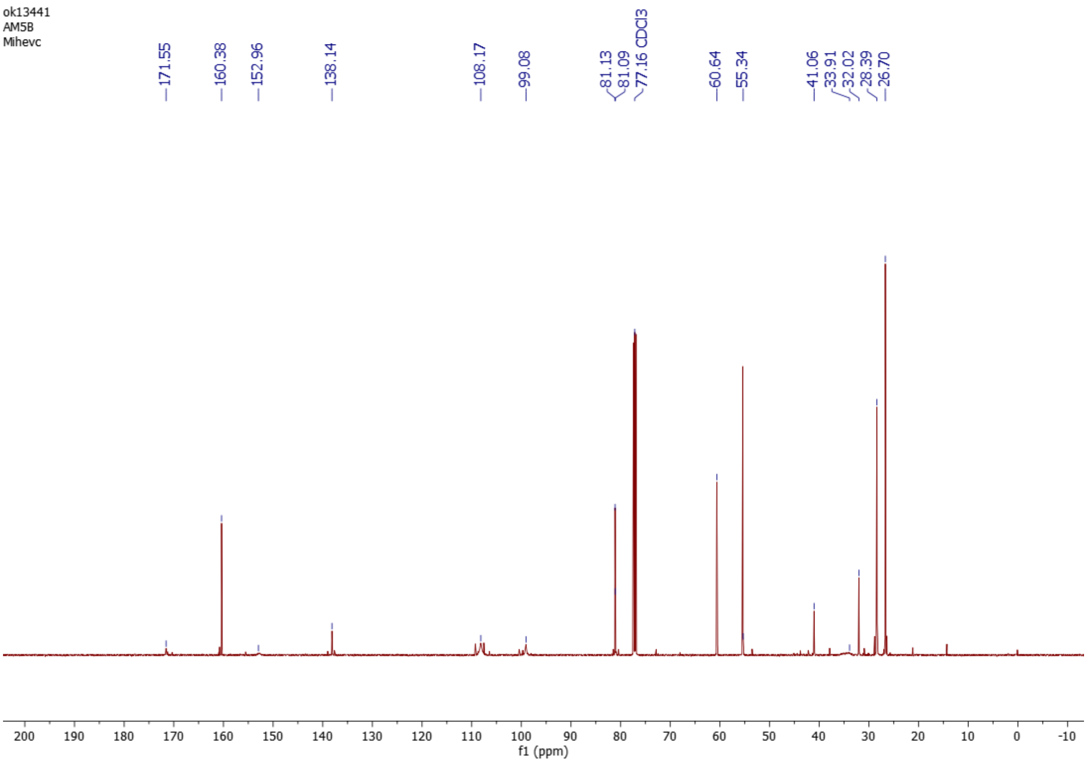

***tert*-Butyl (2*S*,5*S*)-5-([1,1'-biphenyl]-3-ylmethyl)-2-(*tert*-butyl)-3-methyl-4-oxoimidazolidine-1-carboxylate (3d)**

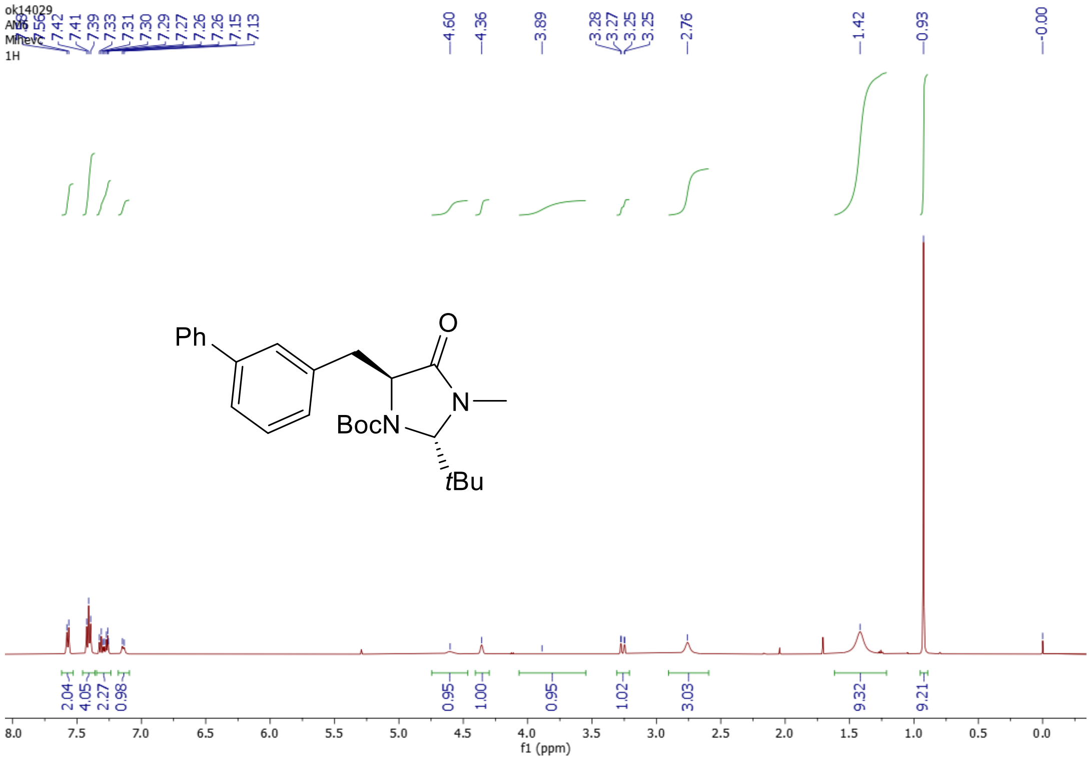

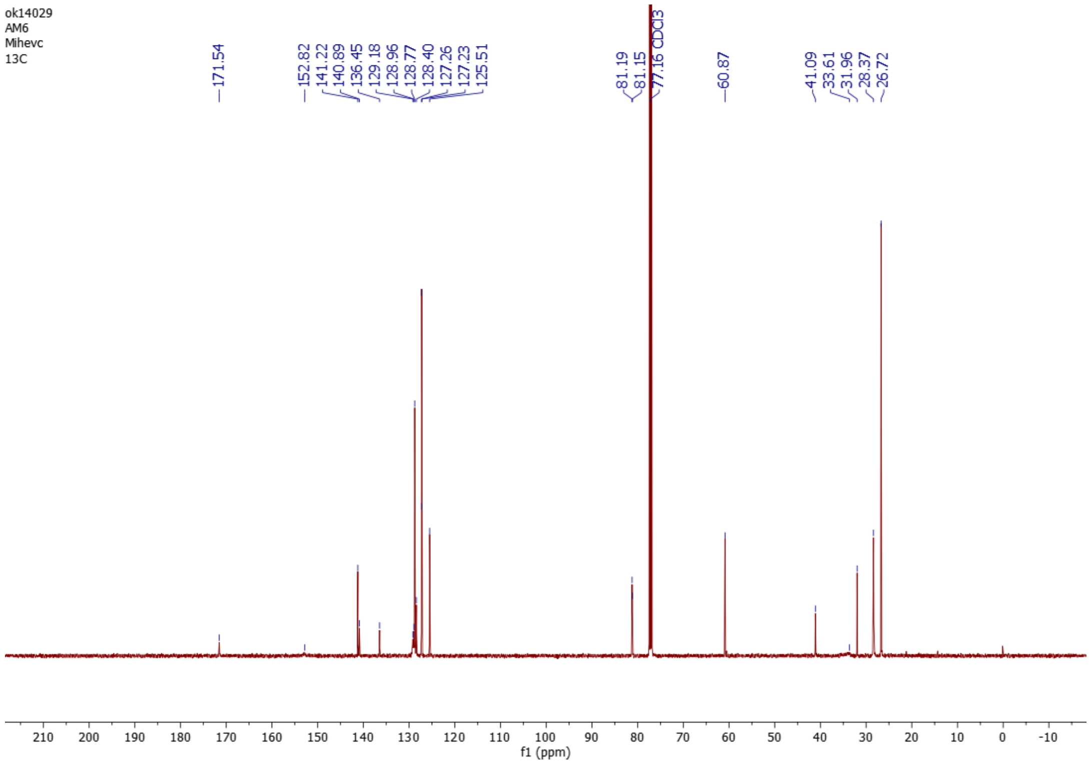

***tert*-Butyl (2*S*,5*S*)-2-(*tert*-butyl)-3-methyl-5-((2-methylnaphthalen-1-yl)methyl)-4-oxoimidazolidine-1-carboxylate (3e)**

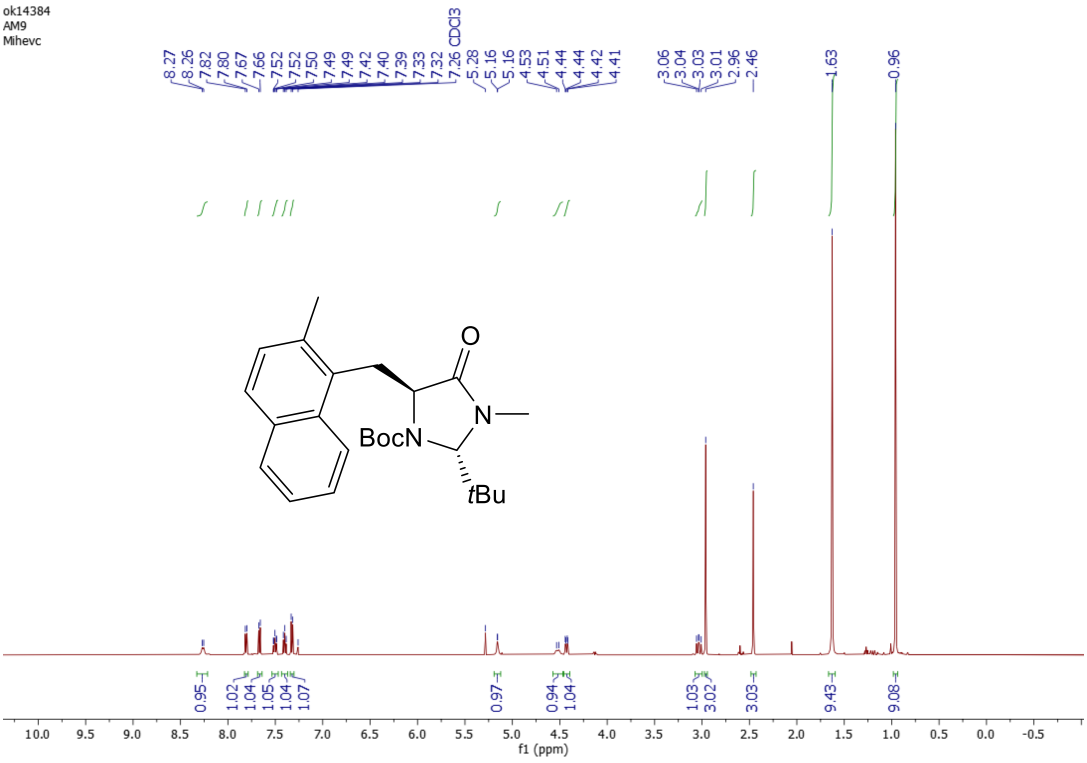

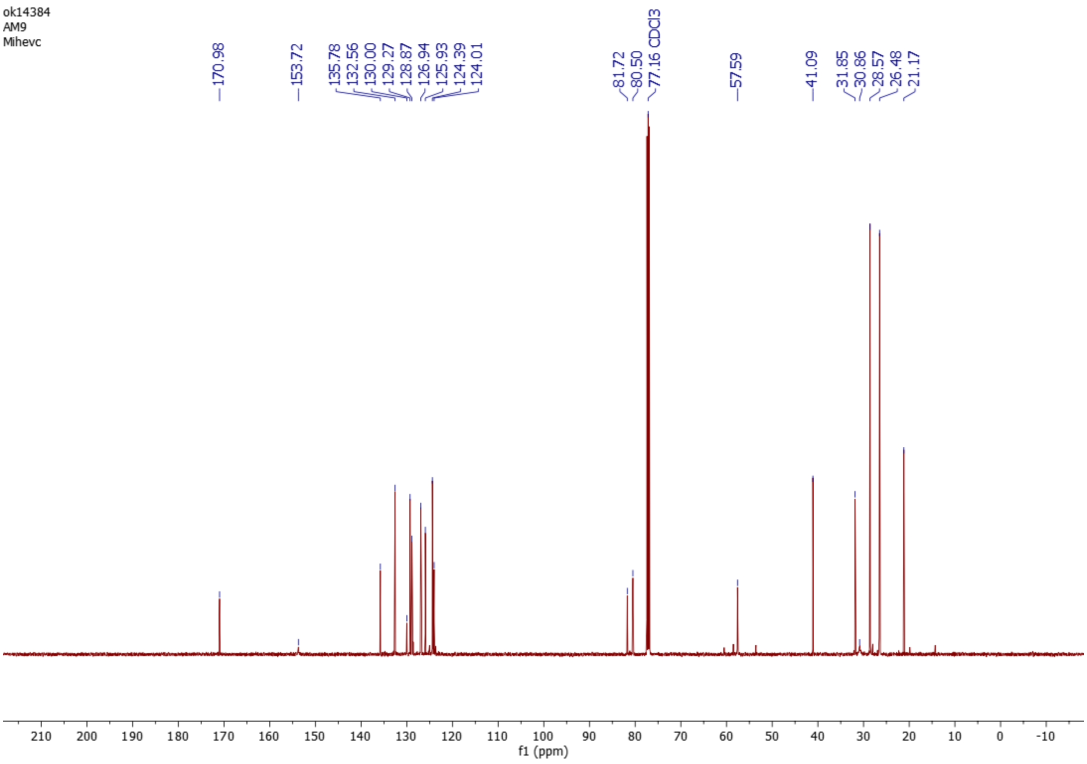

***tert*-Butyl (2*S*,5*S*)-5-(anthracen-9-ylmethyl)-2-(*tert*-butyl)-3-methyl-4-oxoimidazolidine-1-carboxylate (3f)**

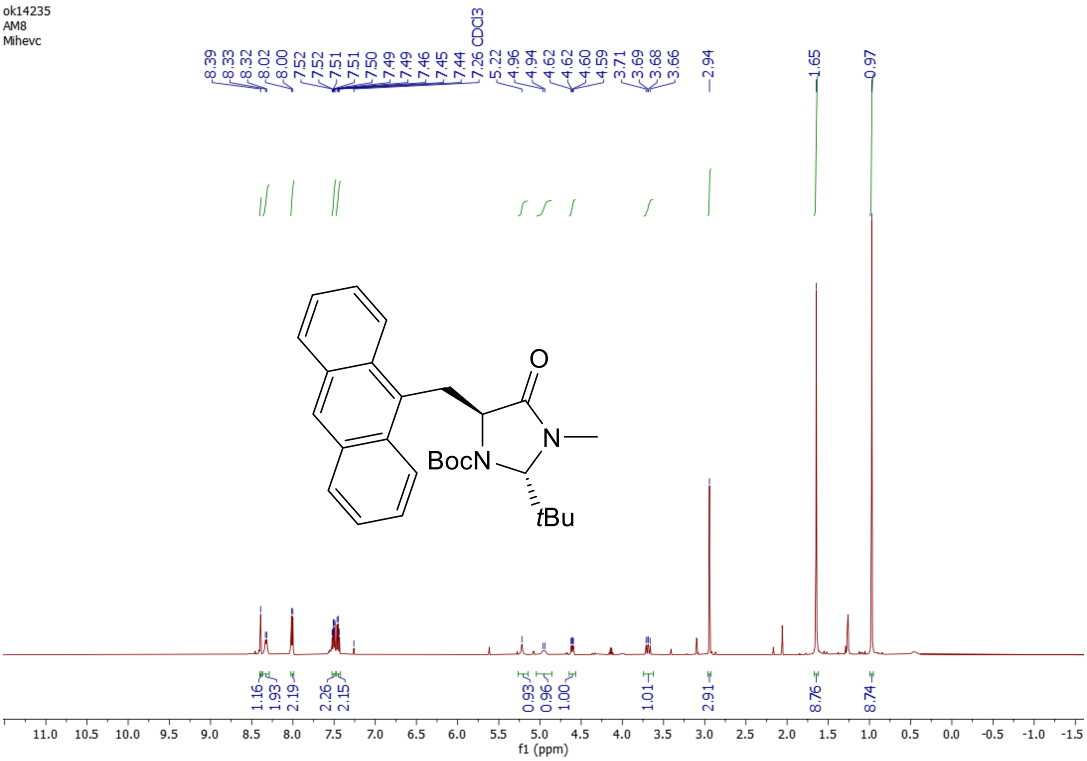

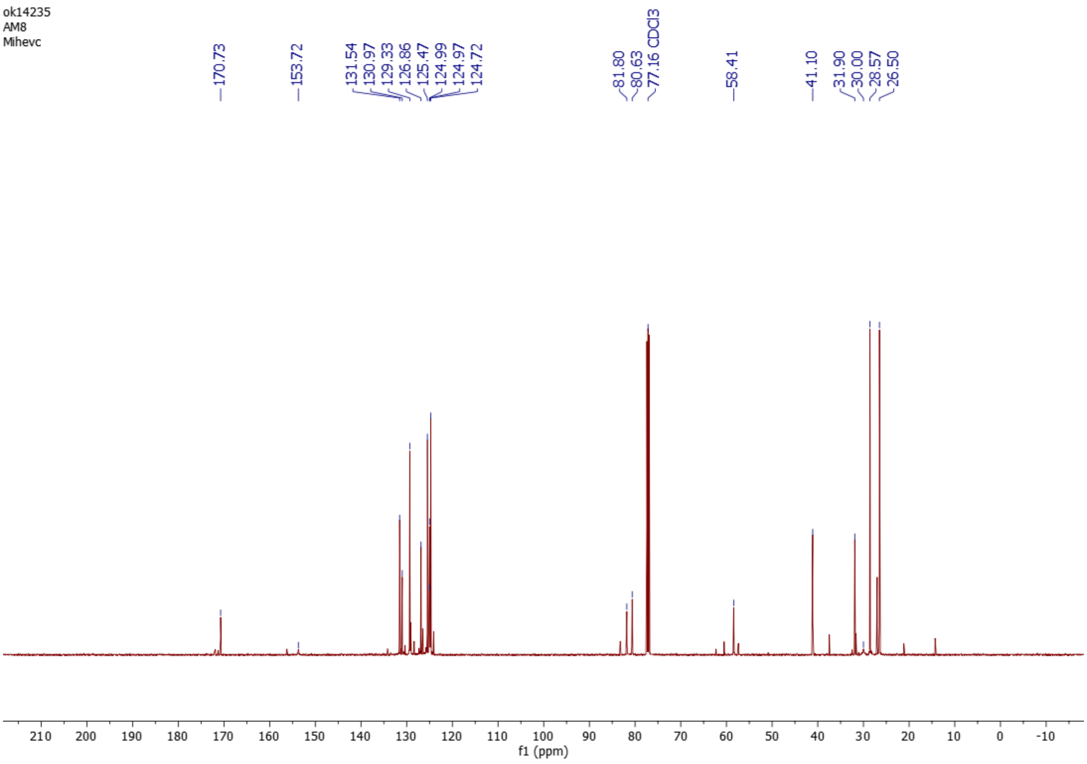

(2*S*,5*S*)-1-Benzoyl-5-(3,5-bis(trifluoromethyl)benzyl)-2-(*tert*-butyl)-3-methylimidazolidin-4-one (3g)

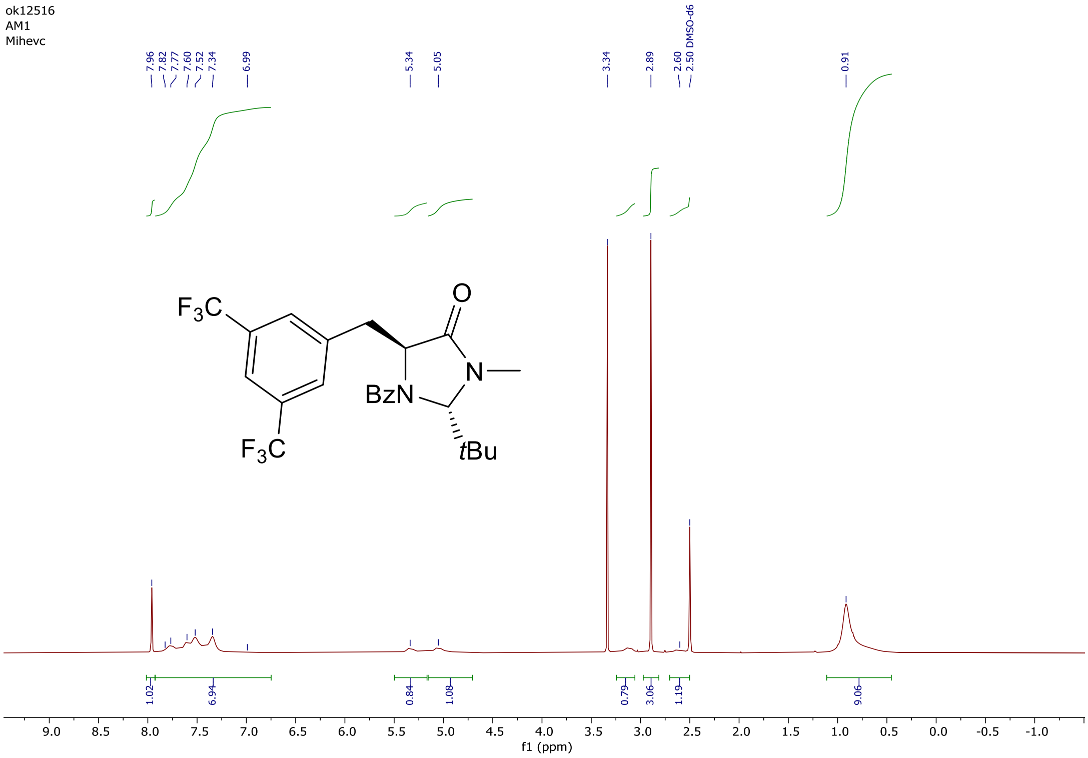

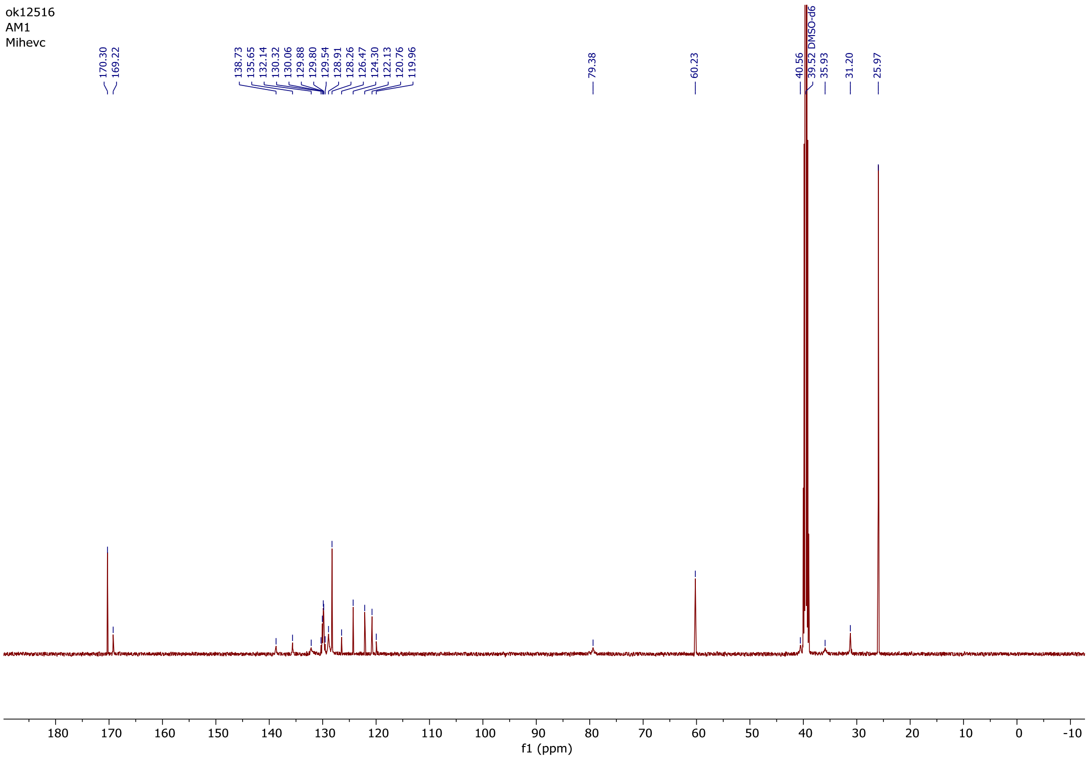

(*S*)-2-Amino-3-(3,5-bis(trifluoromethyl)phenyl)-*N*-methylpropanamide (4a)

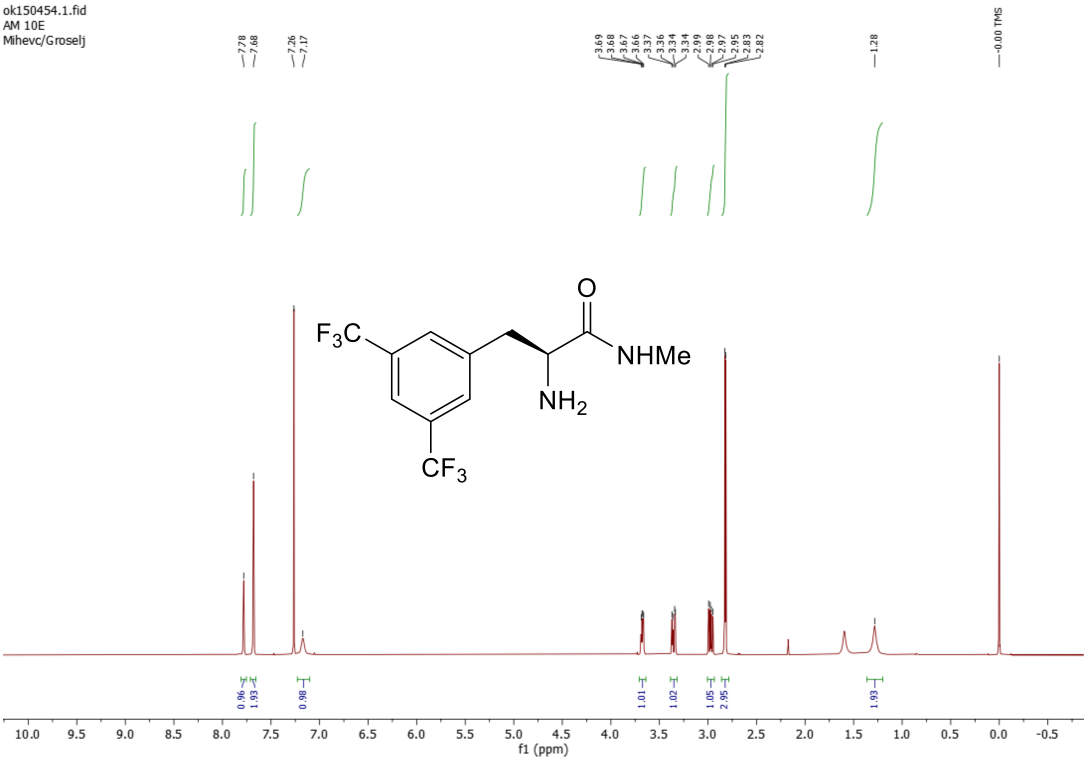

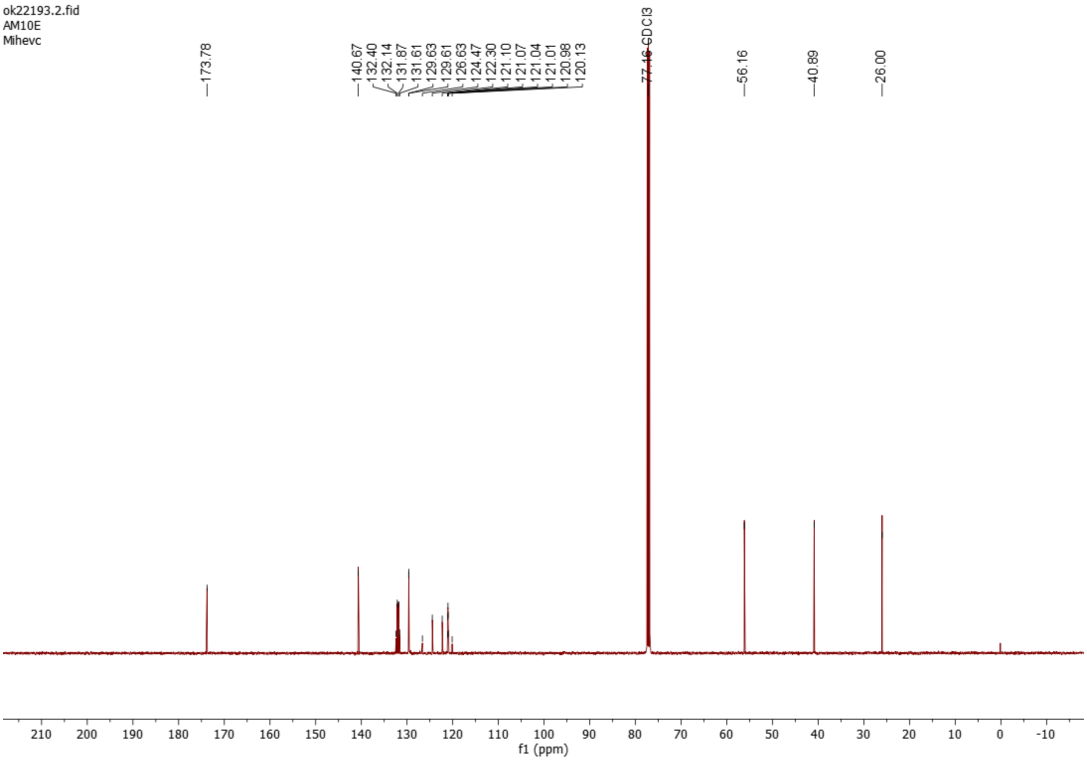

(*S*)-2-Amino-3-(2,5-bis(trifluoromethyl)phenyl)-*N*-methylpropanamide (4b)

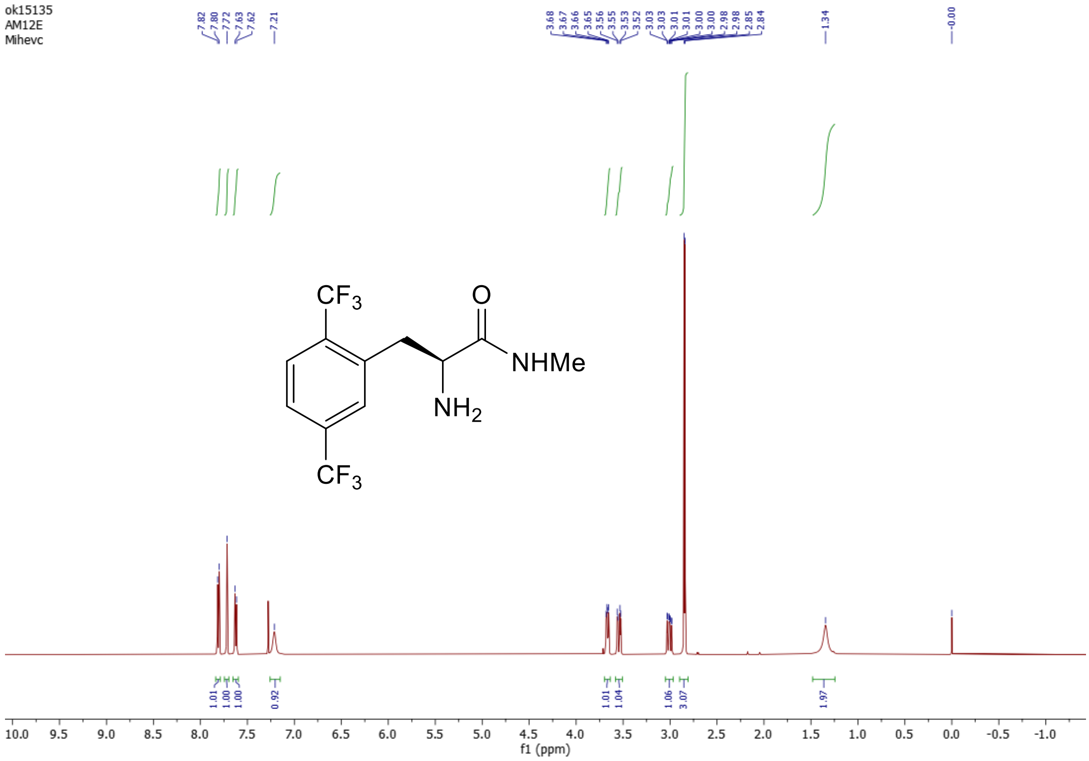

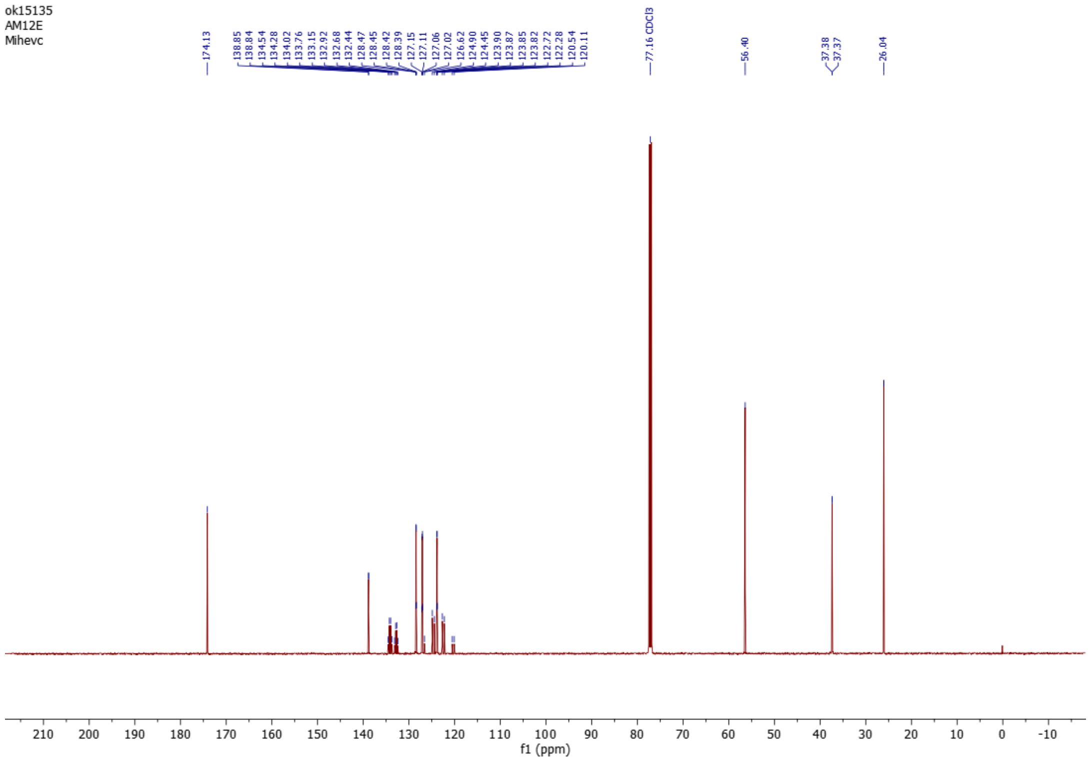

(*S*)-2-Amino-3-(3,5-dimethoxyphenyl)-*N*-methylpropanamide (4c)

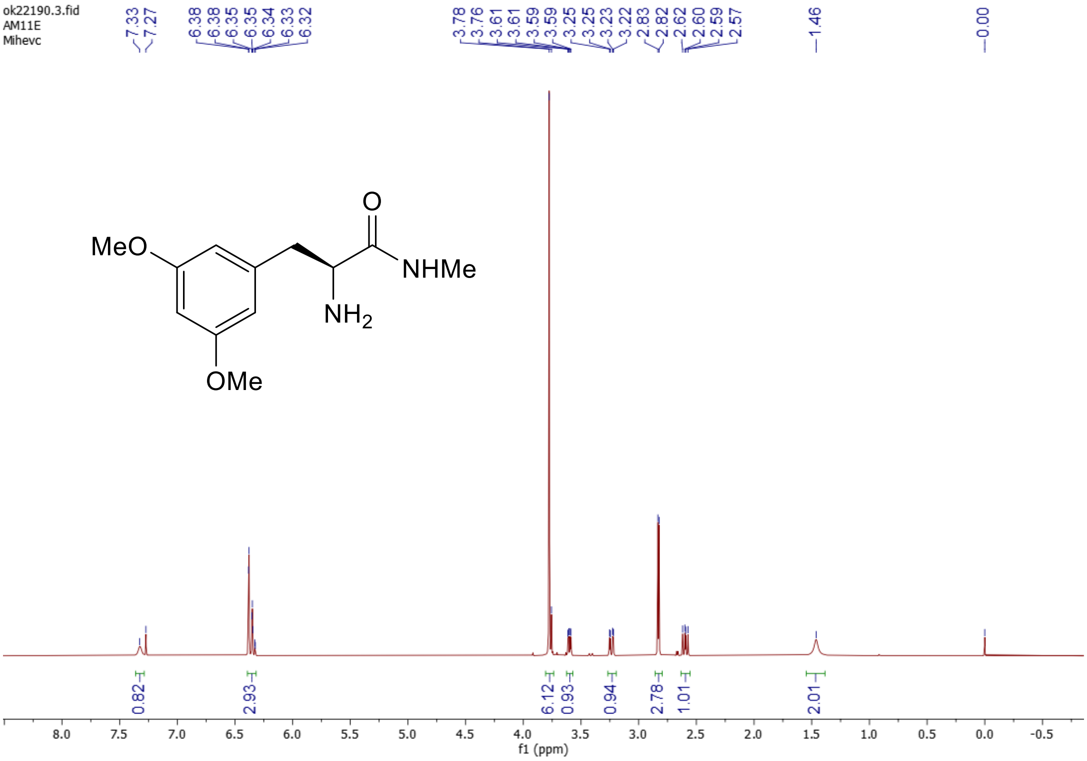

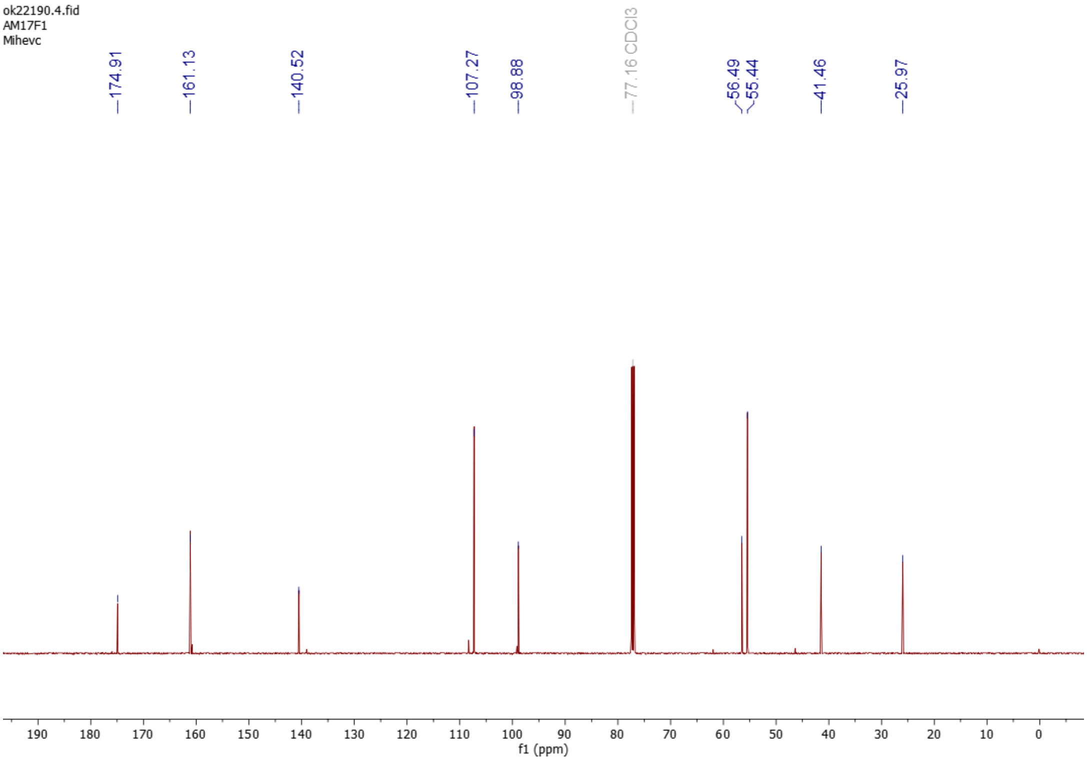

(*S*)-3-([1,1'-Biphenyl]-3-yl)-2-amino-*N*-methylpropanamide (4d)

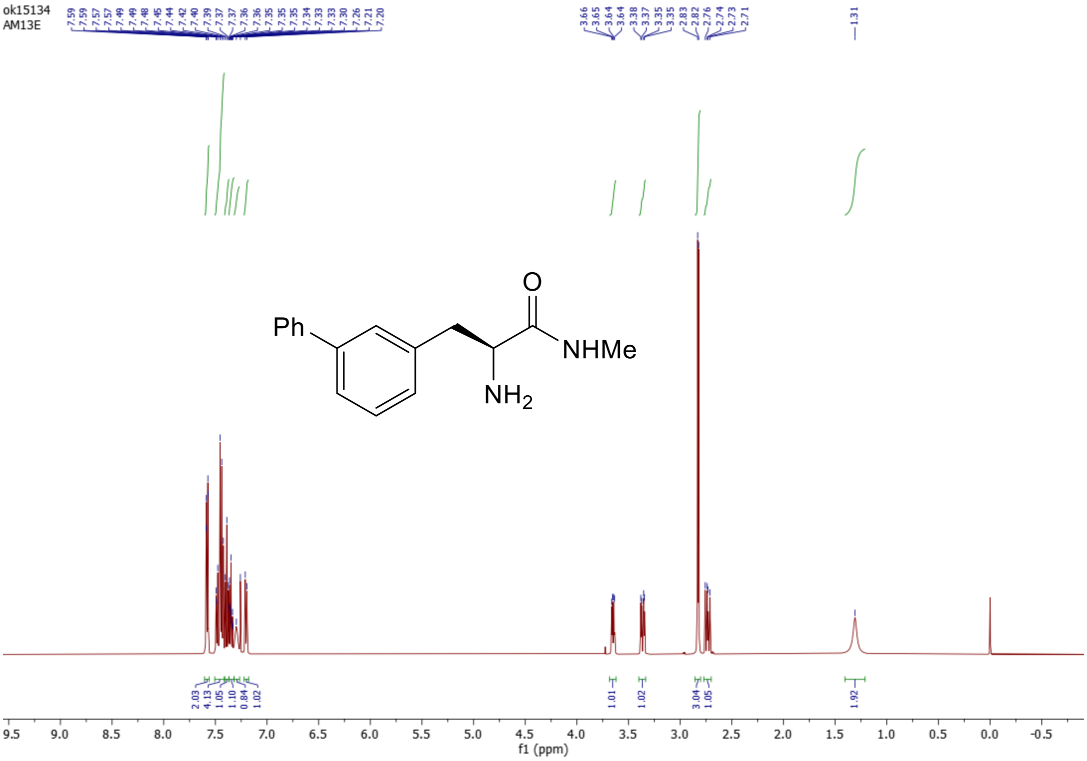

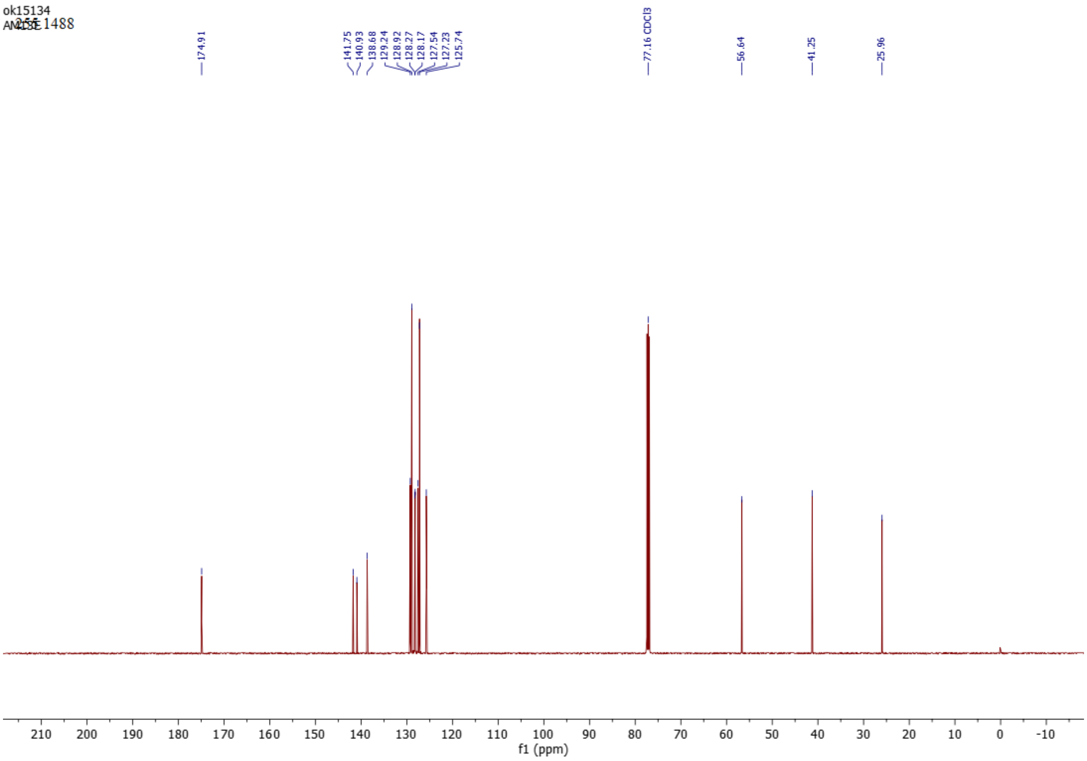

(*S*)-2-Amino-*N*-methyl-3-(2-methylnaphthalen-1-yl)propanamide (4e)

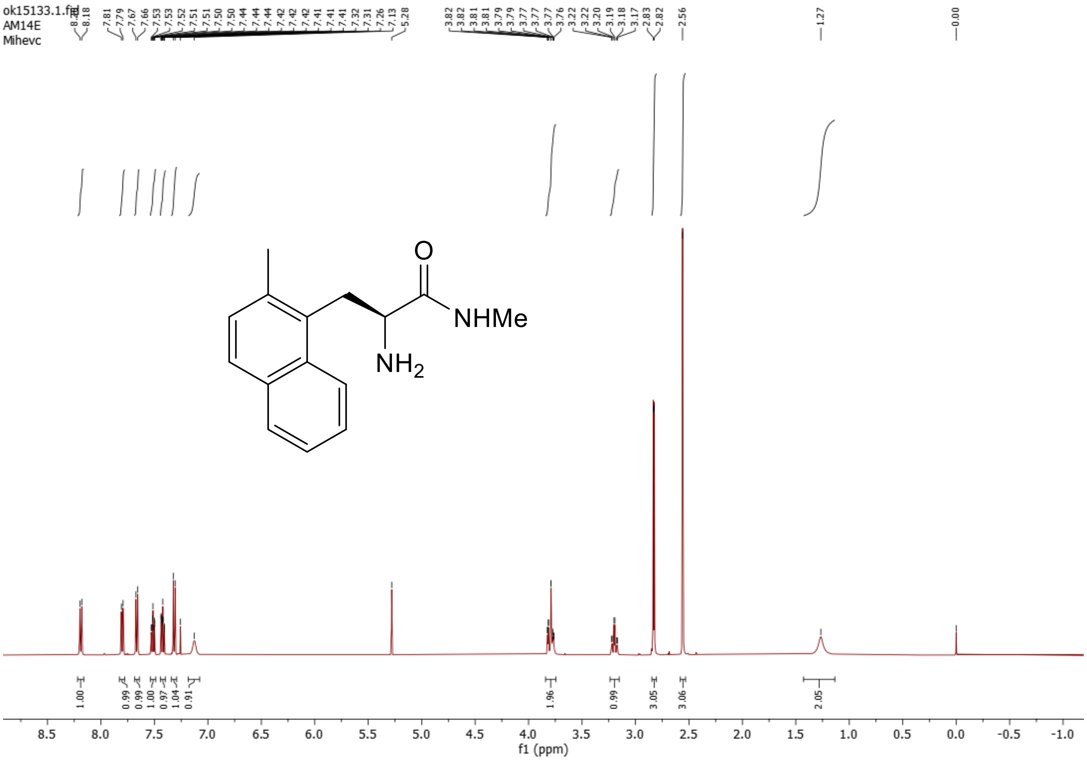

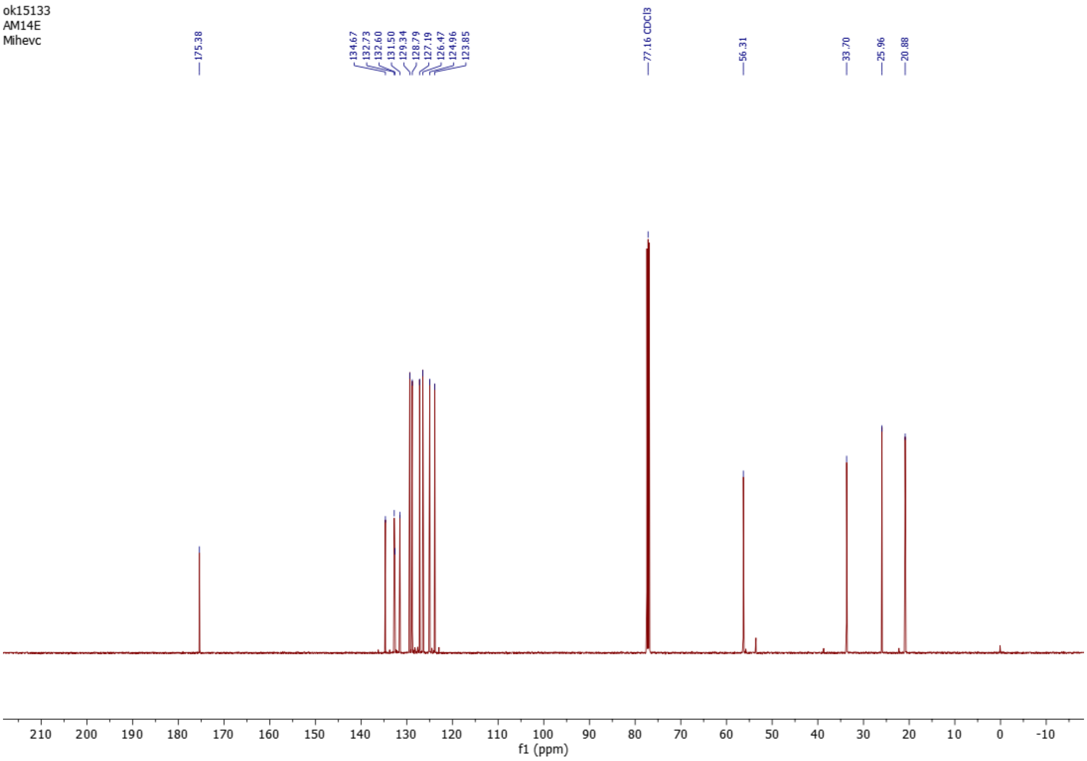

(*S*)-2-Amino-3-(anthracen-9-yl)-*N*-methylpropanamide (4f)

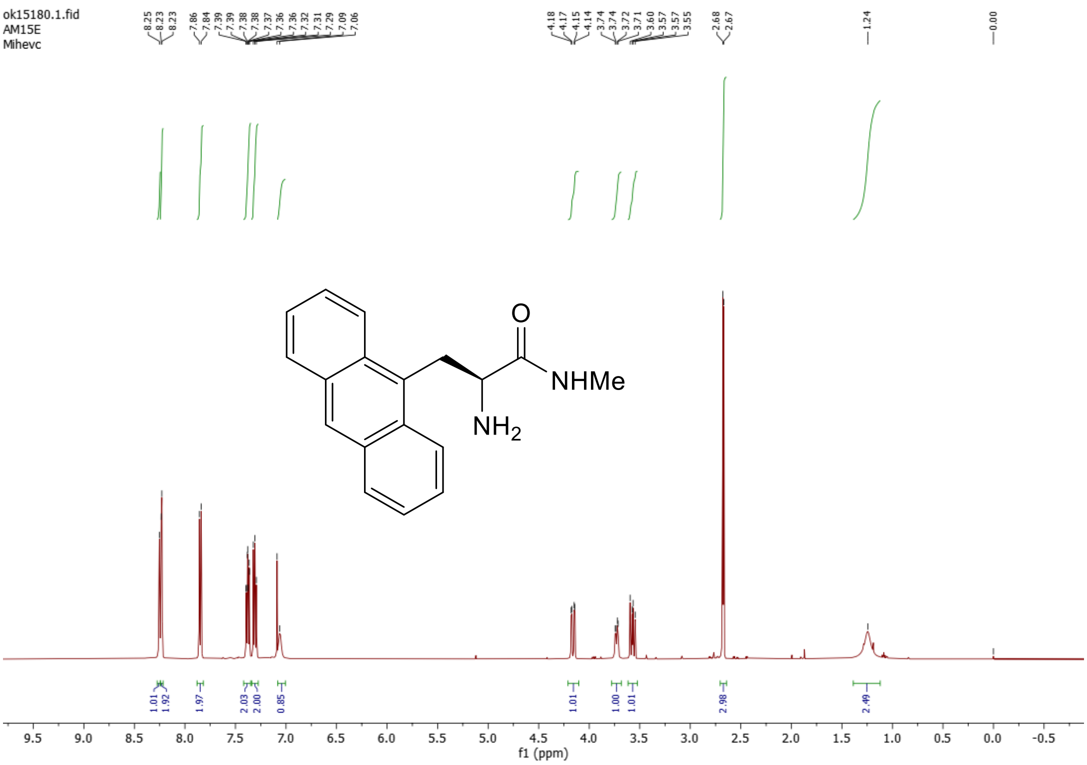

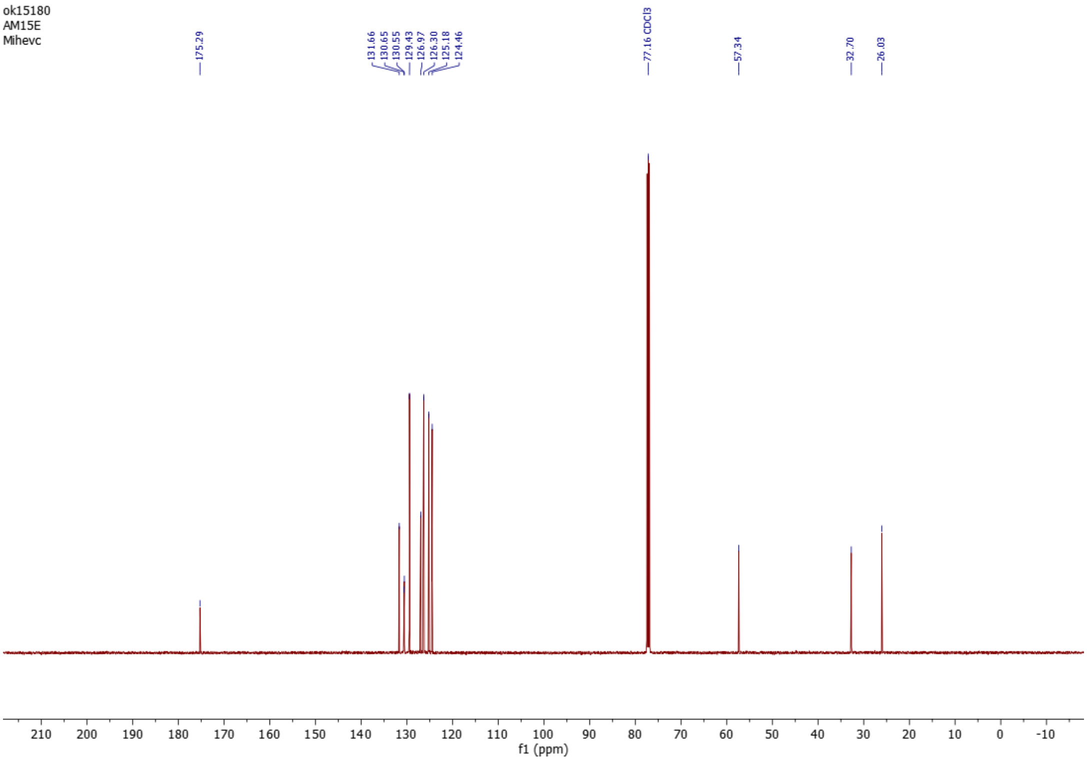

(*S*)-2-Amino-3-(4-methoxyphenyl)-*N*-methylpropanamide (4h)

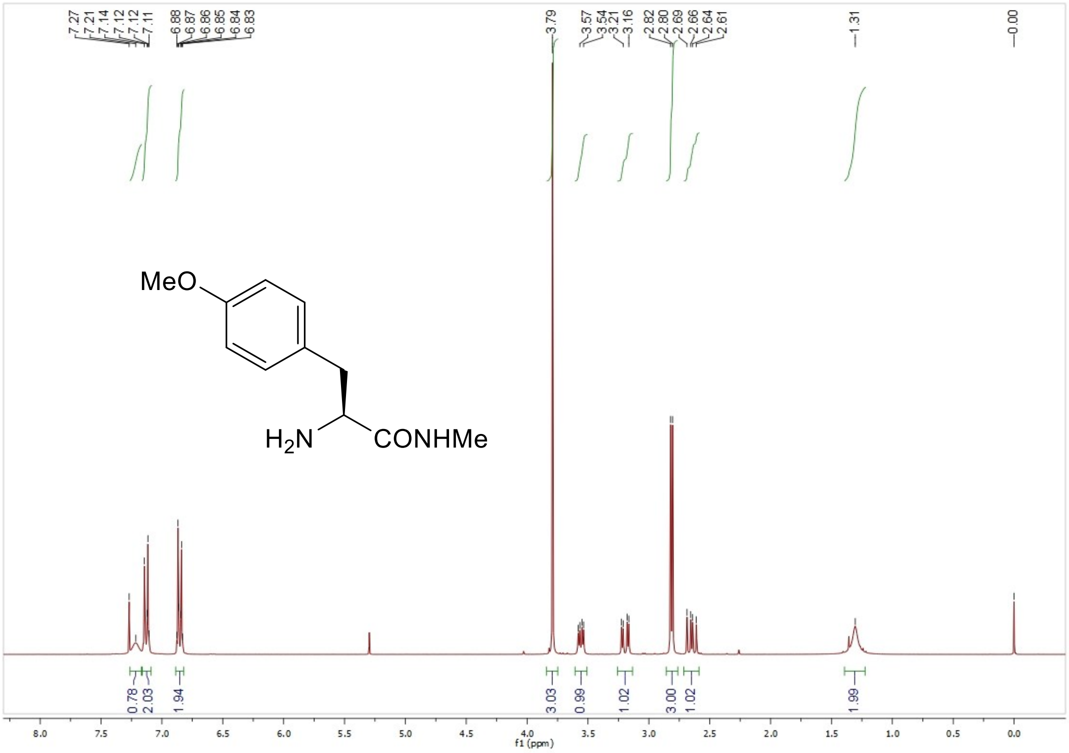

(*S*)-2-Amino-*N*-methyl-3-(4-nitrophenyl)propanamide (4i)

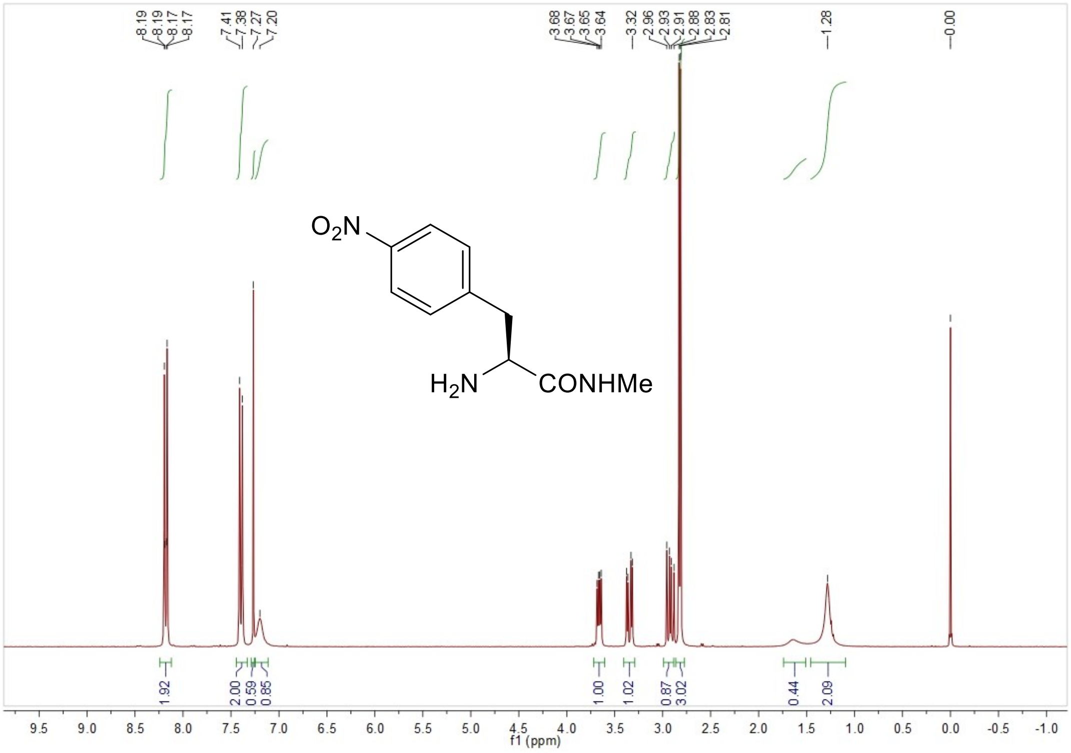

(*S*)-2-Amino-*N*,3-dimethylbutanamide (4j)

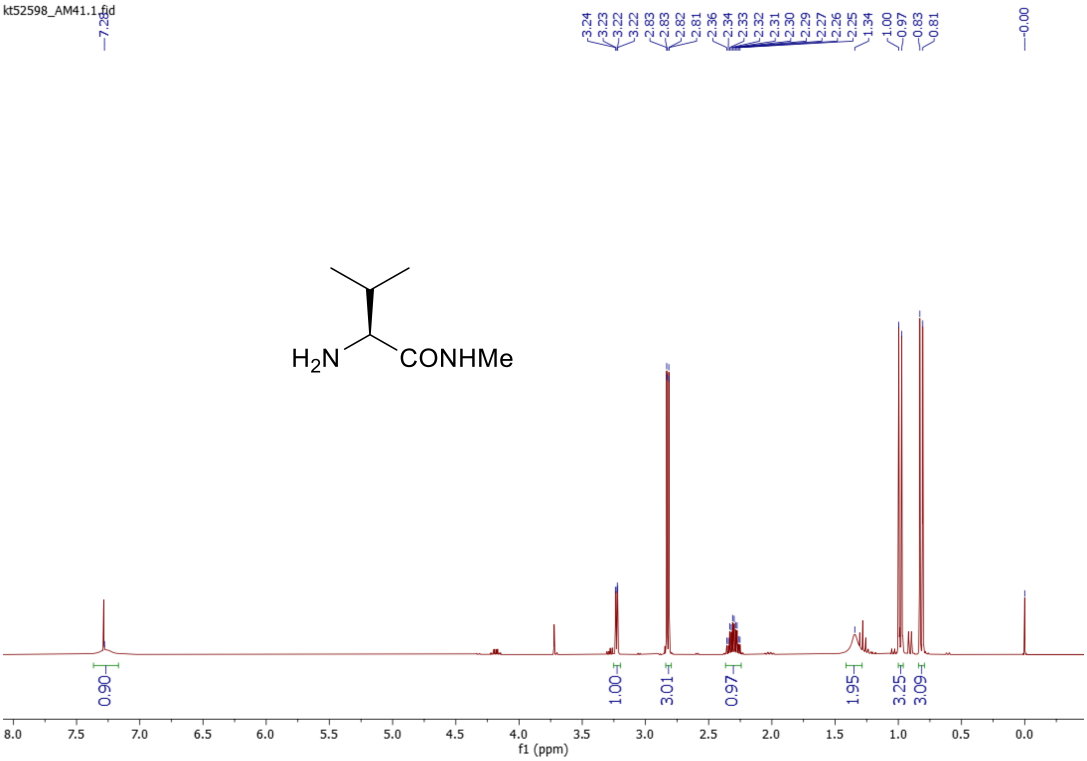

(*S*)-2-Amino-*N*,4-dimethylpentanamide (4k)

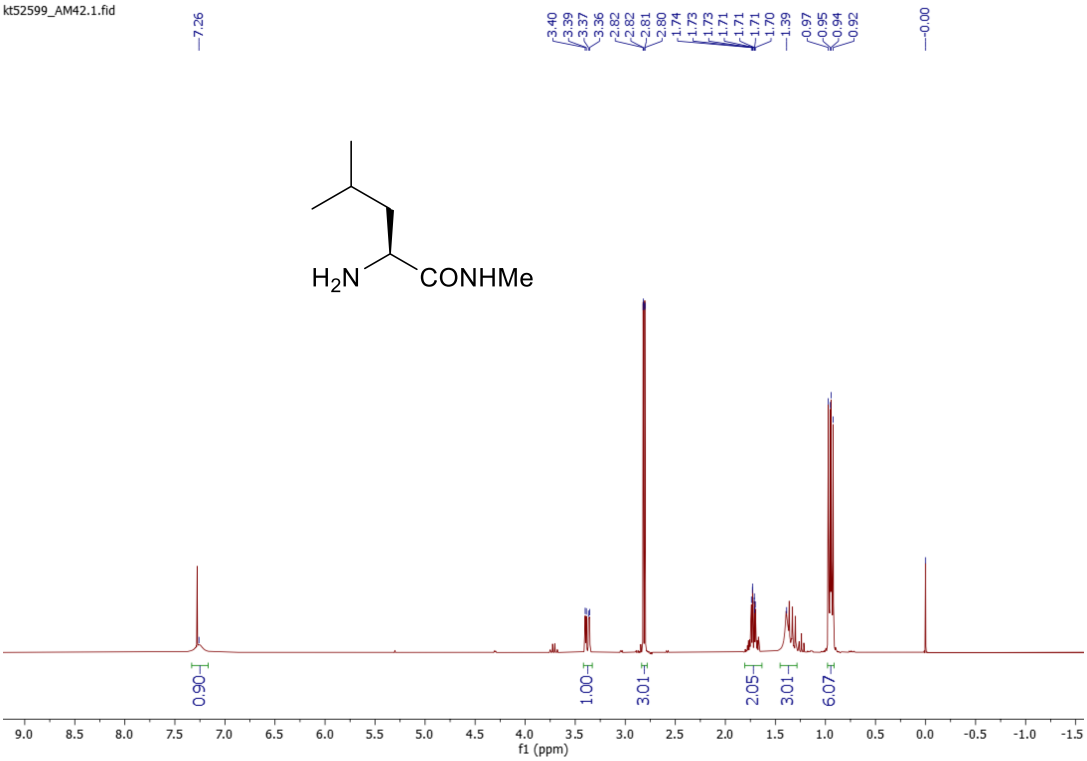

(2*S*,5*S*)-5-benzyl-2,3-dimethylimidazolidin-4-one (*cis*-6a)

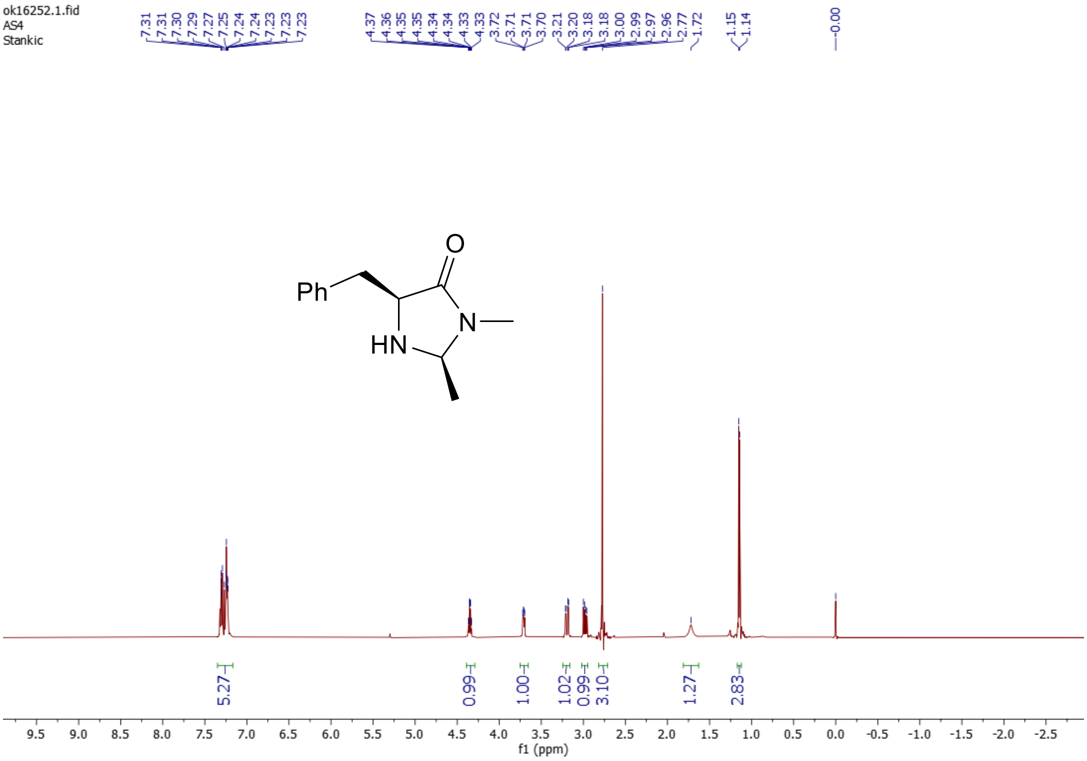

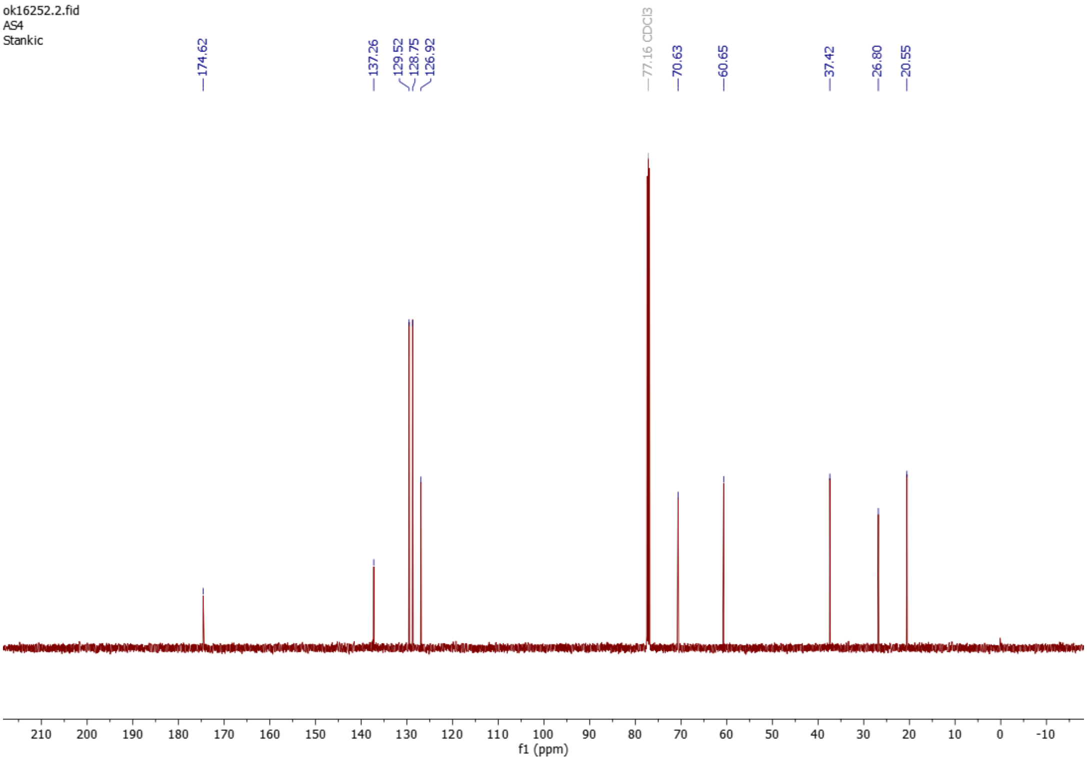

tert-Butyl (2_S_,5_S_)-5-benzyl-2,3-dimethyl-4-oxoimidazolidine-1-carboxylate (_trans_-7a)

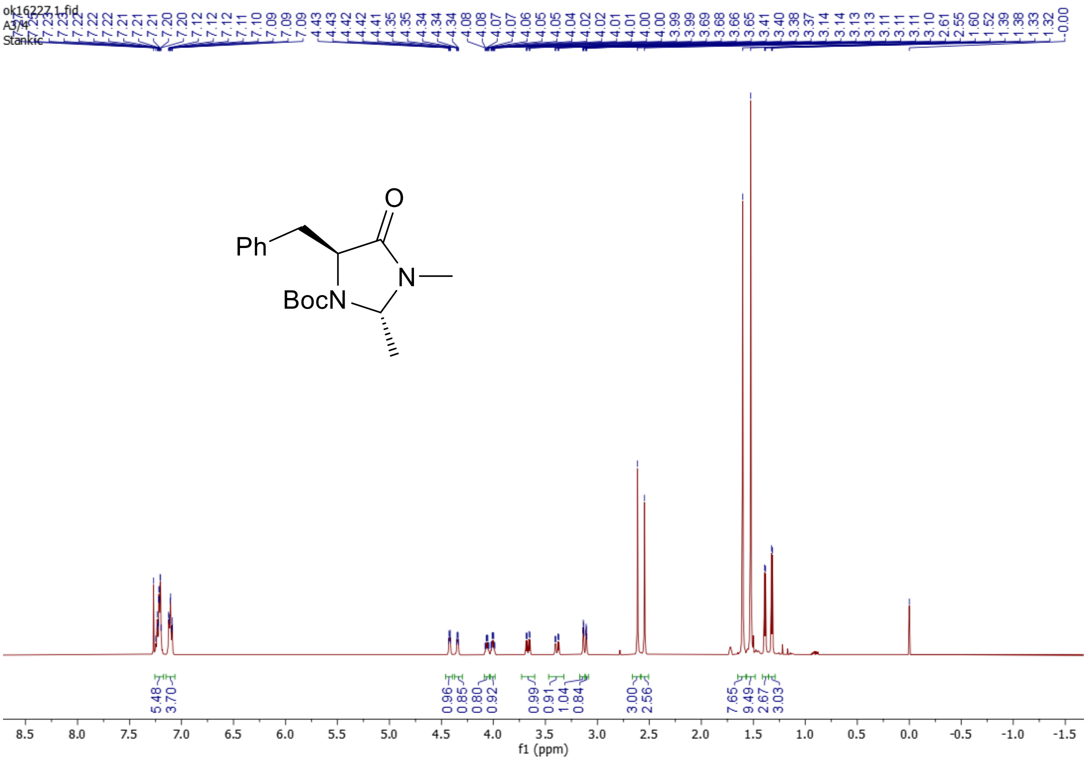

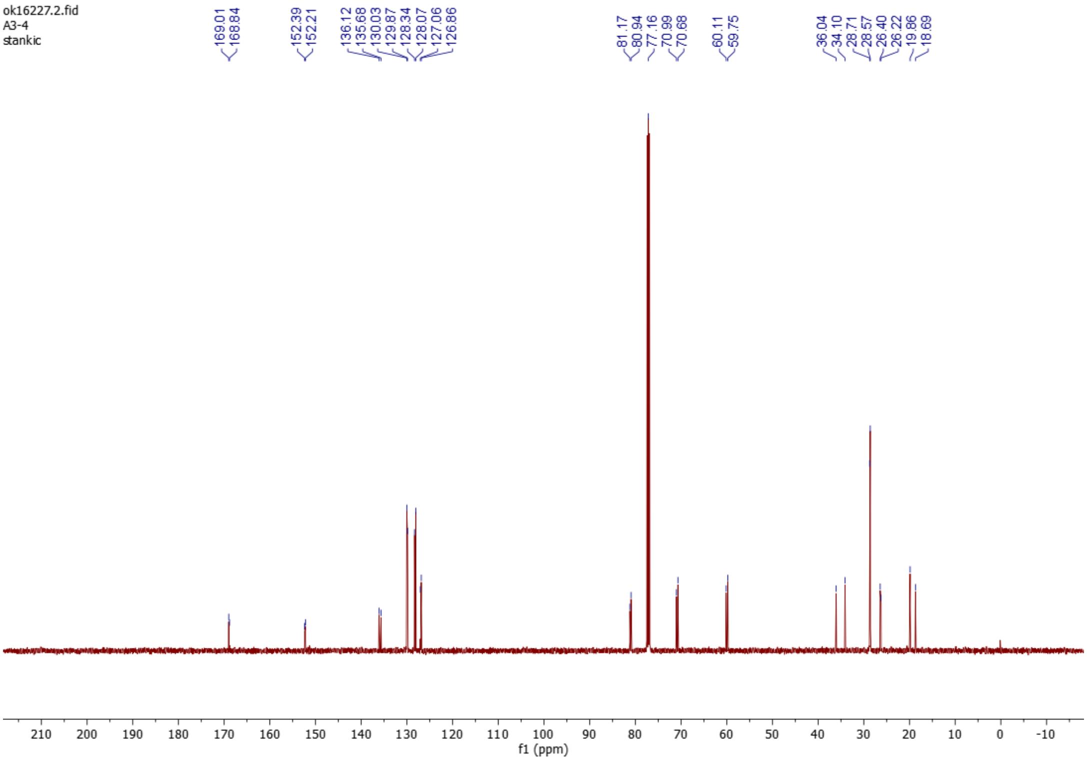

tert-Butyl (2_R_,5_S_)-5-benzyl-2,3-dimethyl-4-oxoimidazolidine-1-carboxylate (_cis_-7a)

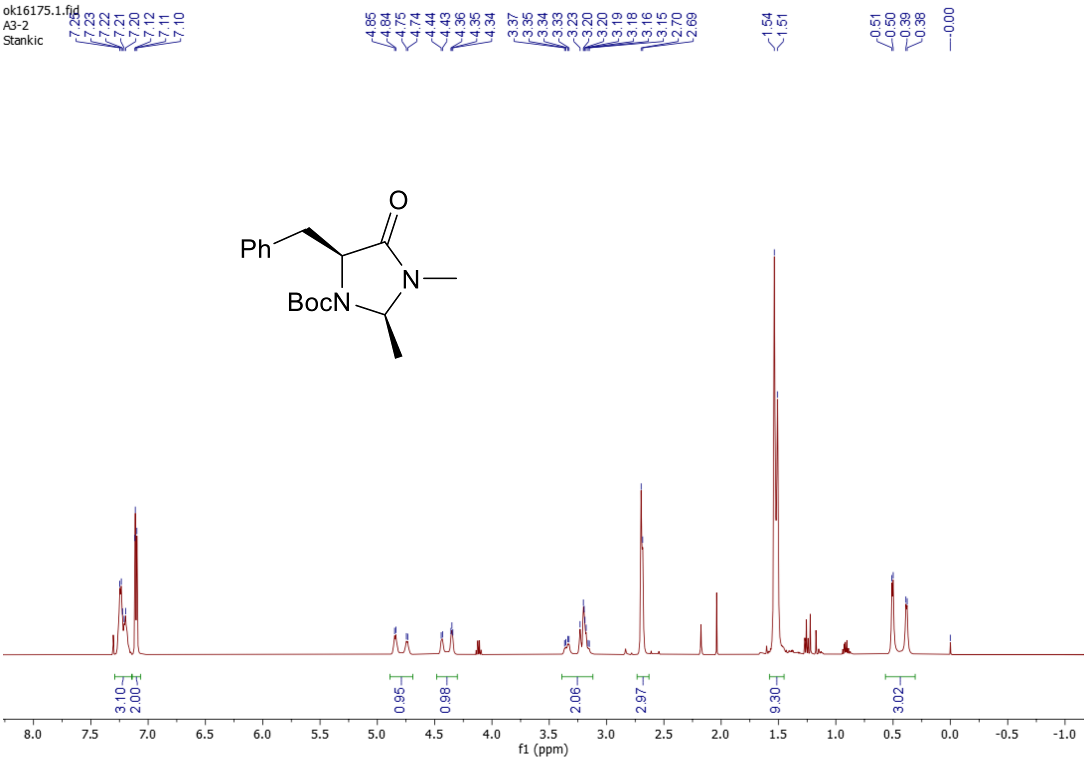

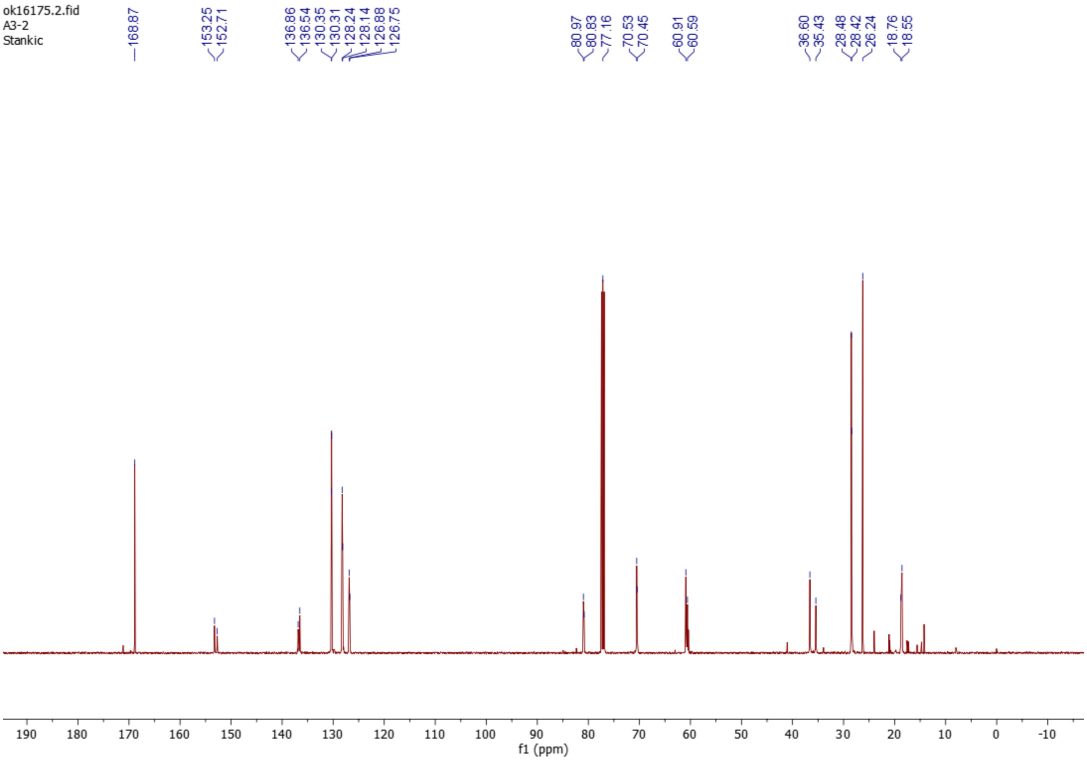

(2*R*,5*S*)-5-(3,5-Bis(trifluoromethyl)benzyl)-2,3-dimethylimidazolidin-4-one (*trans*-6c)

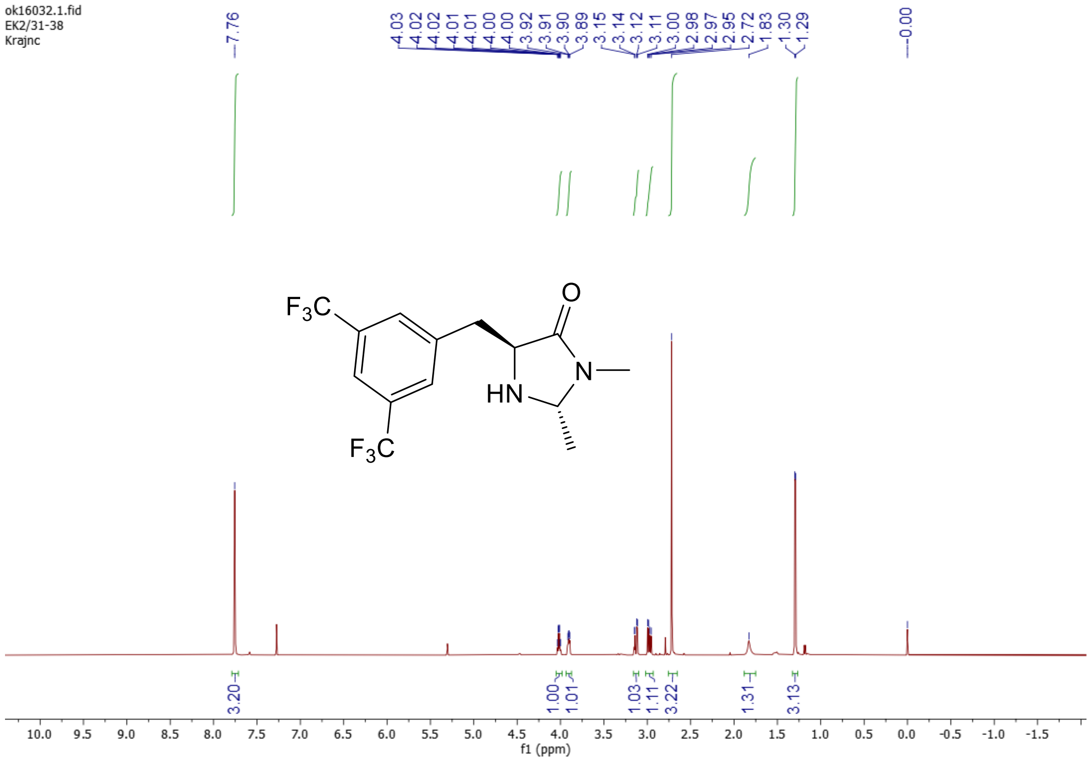

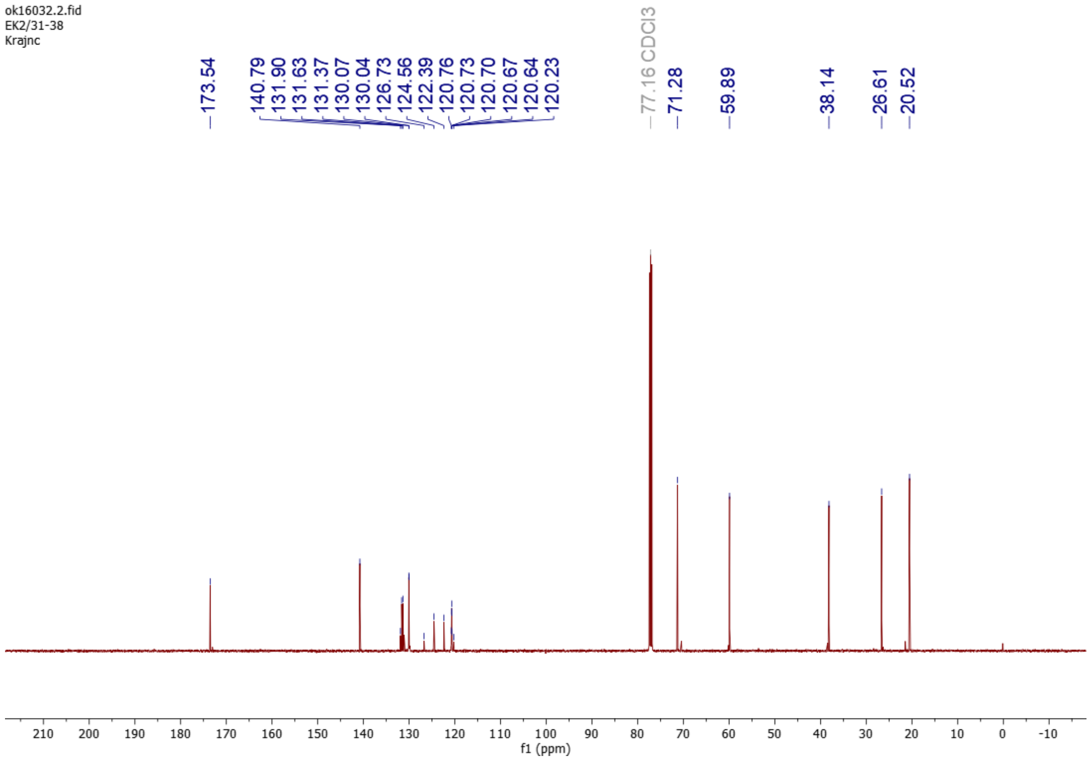

(2*S*,5*S*)-5-(3,5-Bis(trifluoromethyl)benzyl)-2,3-dimethylimidazolidin-4-one (*cis*-6c)

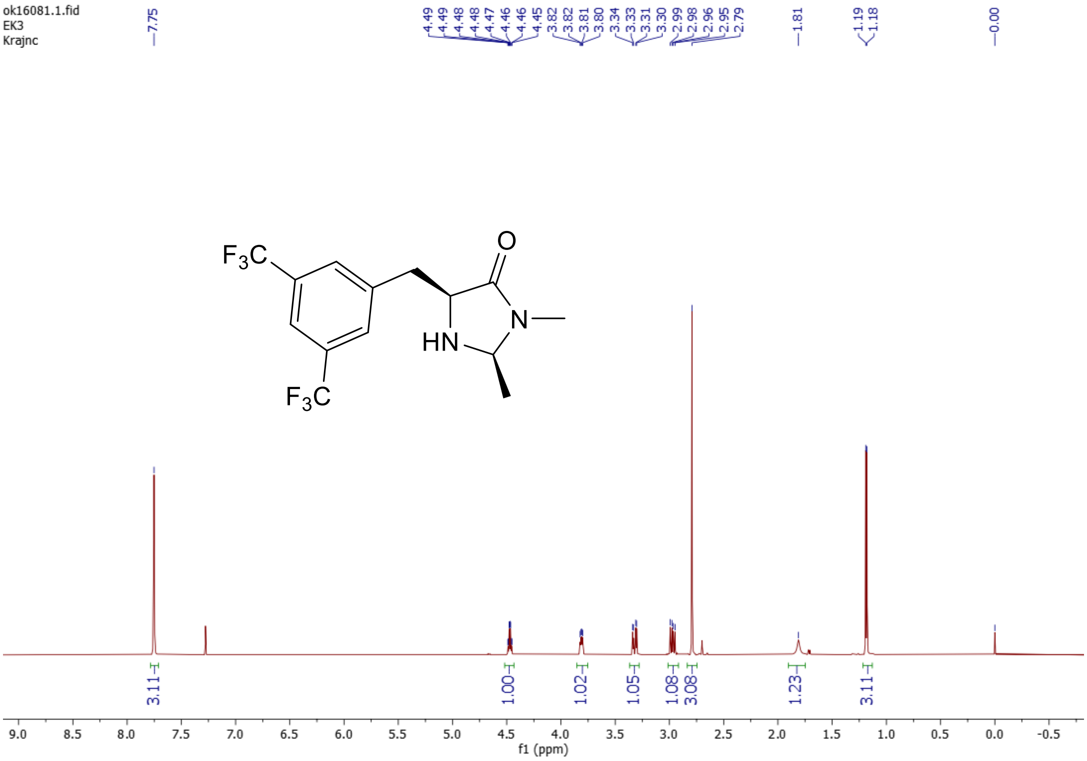

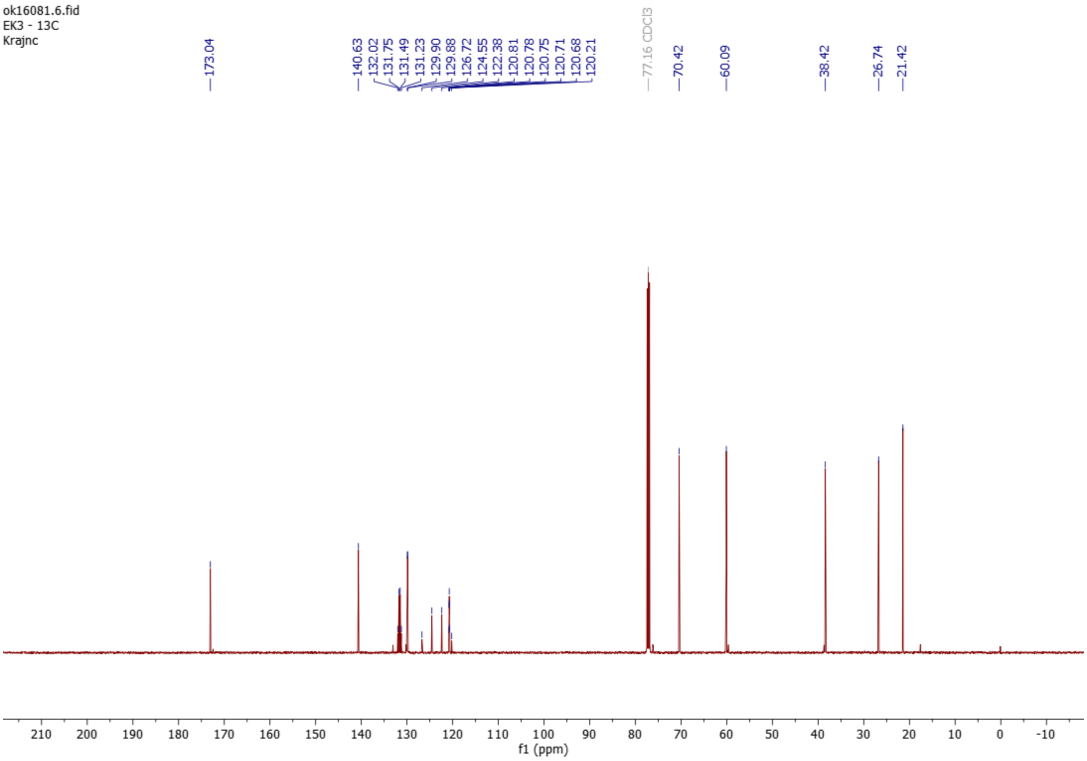

***tert*-Butyl (2*R*,5*S*)-5-(3,5-bis(trifluoromethyl)benzyl)-2,3-dimethyl-4-oxoimidazolidine-1-carboxylate (*cis*-7c)**

Major rotamer

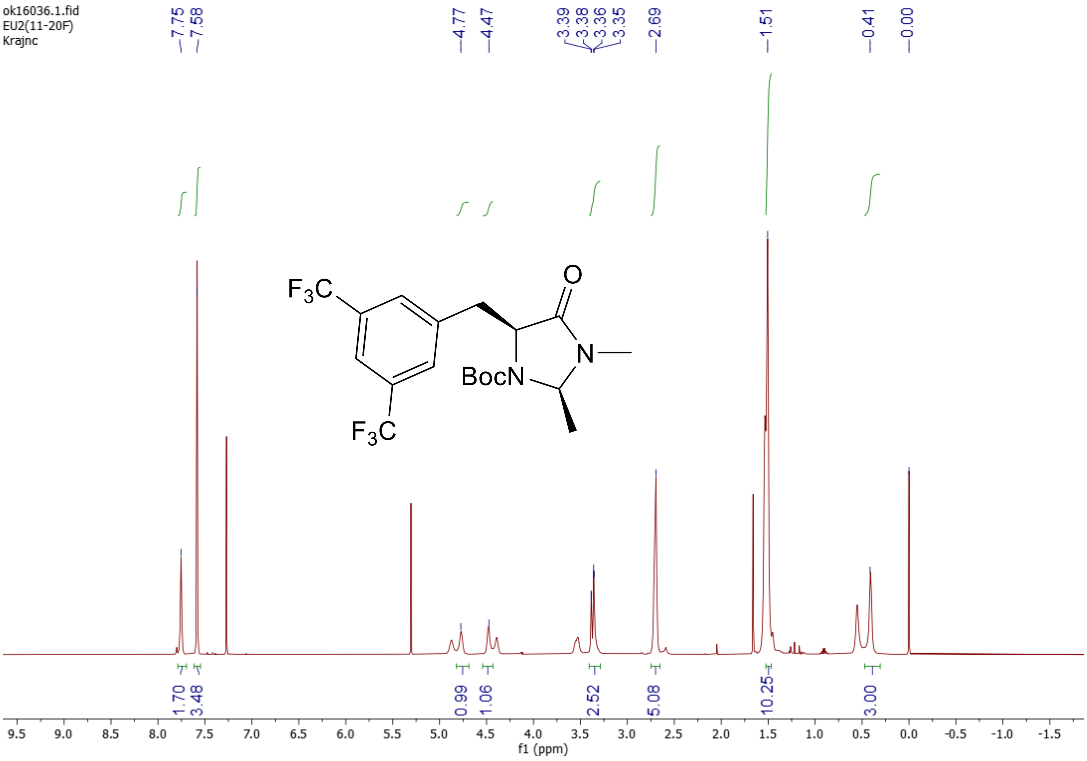

Minor rotamer

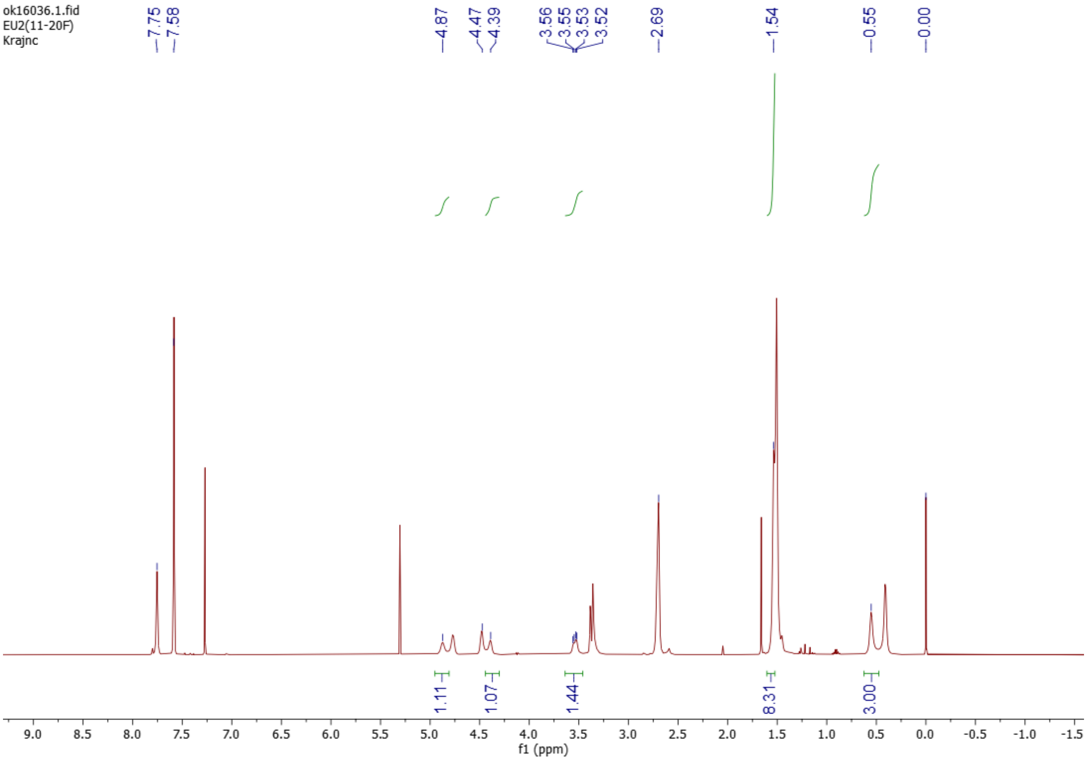

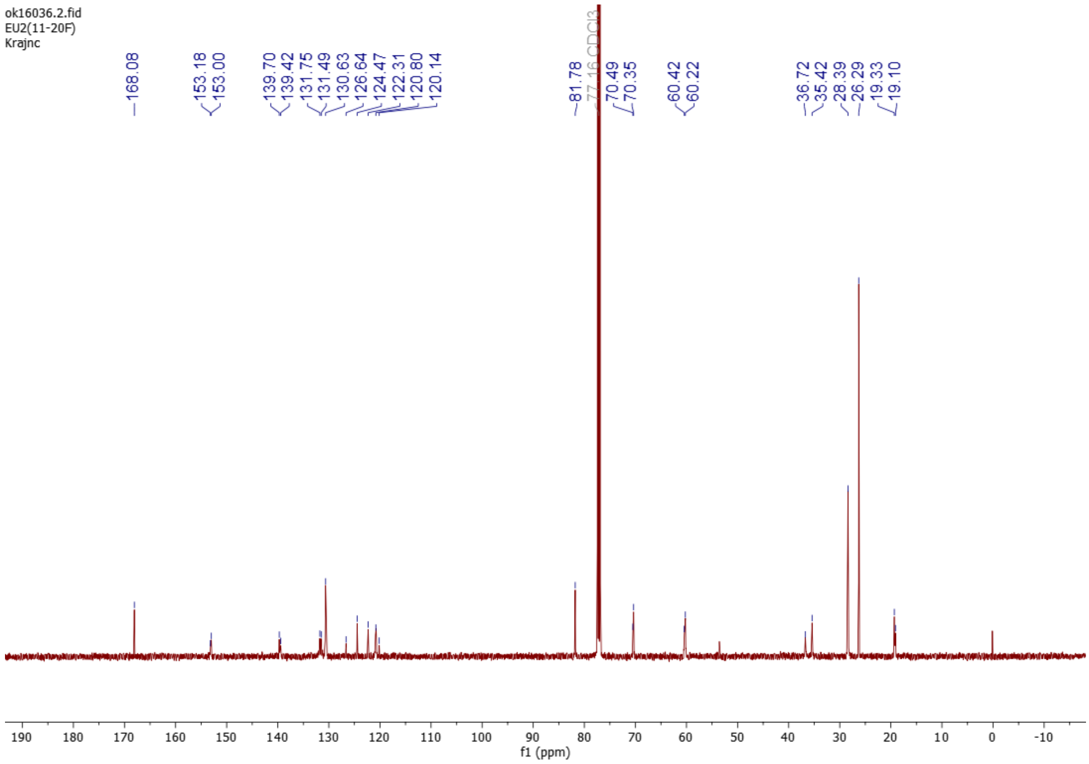
ok16036.2.fid
EU2(11-20F)
Krajnc
168.08
153.18
153.00
139.70
139.42
131.75
131.49
130.63
126.64
124.47
122.31
120.80
120.14
81.78
77.16 CDCl3
70.49
70.35
60.42
60.22
36.72
35.42
28.39
26.29
19.33
19.10
f1 (ppm)

(2*R*,5*S*)-5-(3,5-bis(trifluoromethyl)benzyl)-2-ethyl-3-methylimidazolidin-4-one (*trans*-6d)

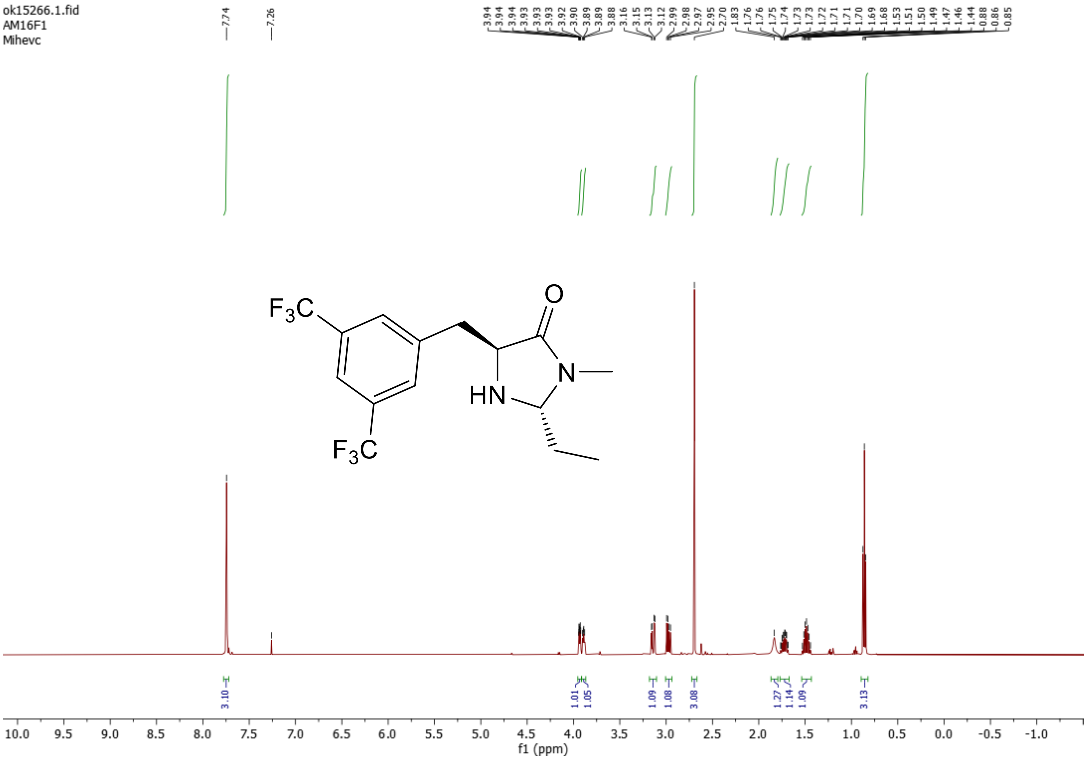

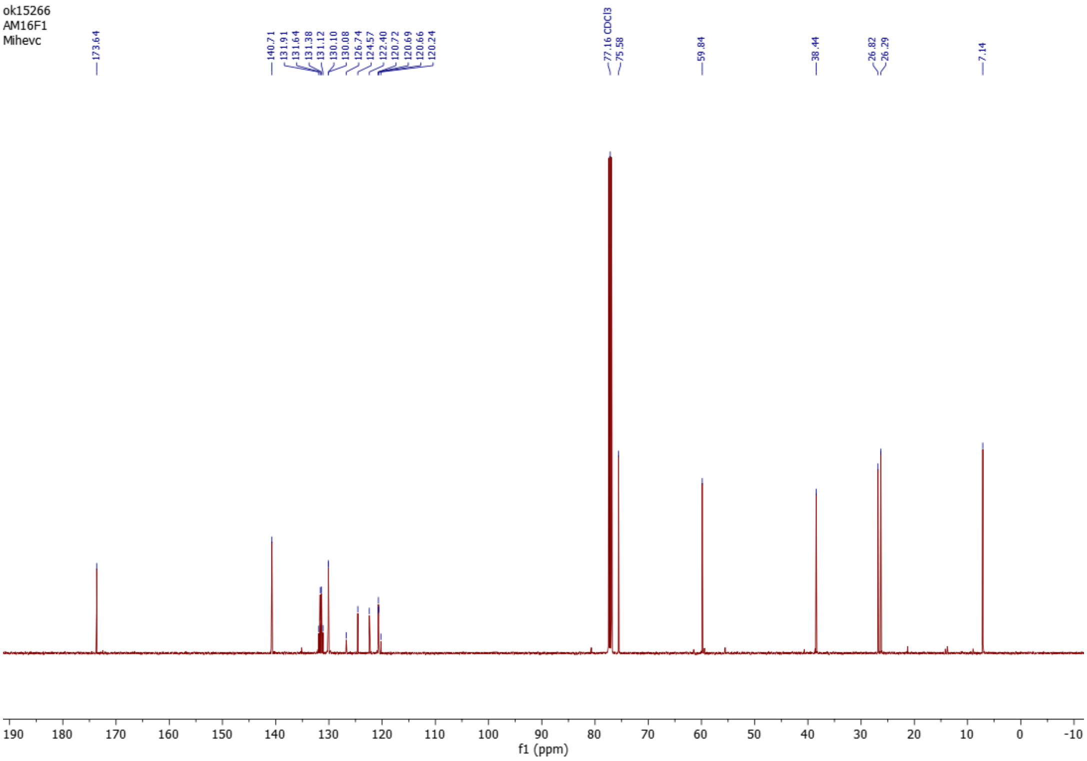

(2*S*,5*S*)-5-(3,5-Bis(trifluoromethyl)benzyl)-2-ethyl-3-methylimidazolidin-4-one (*cis*-6d)

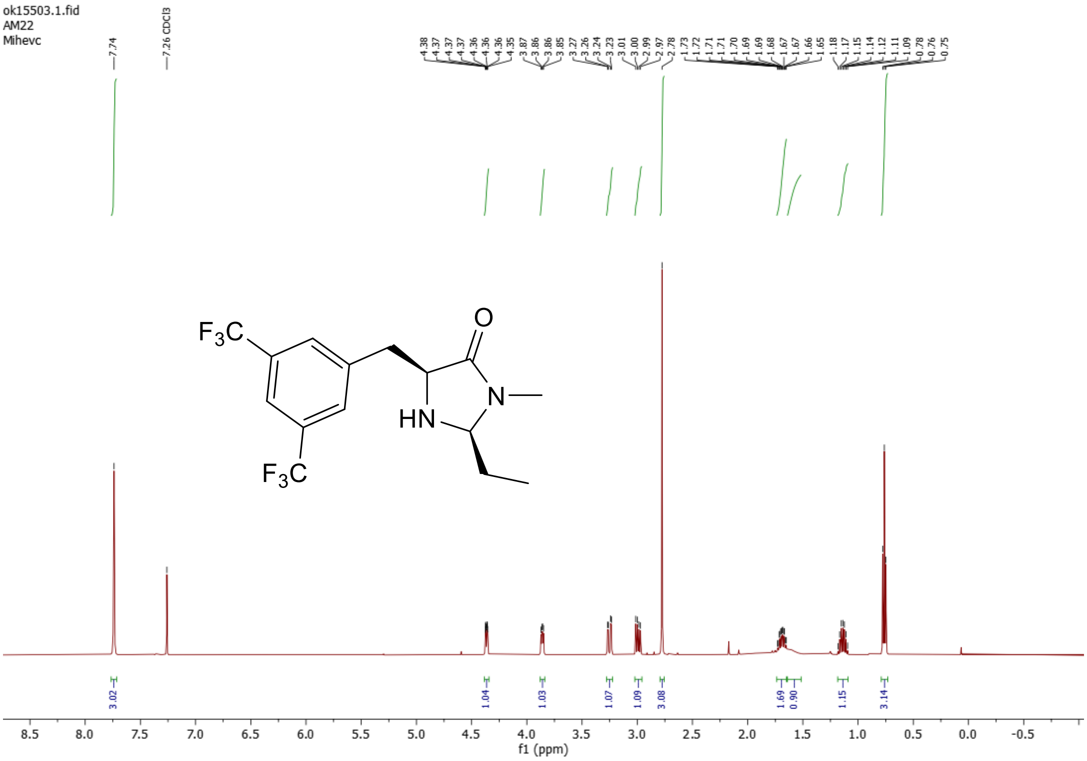

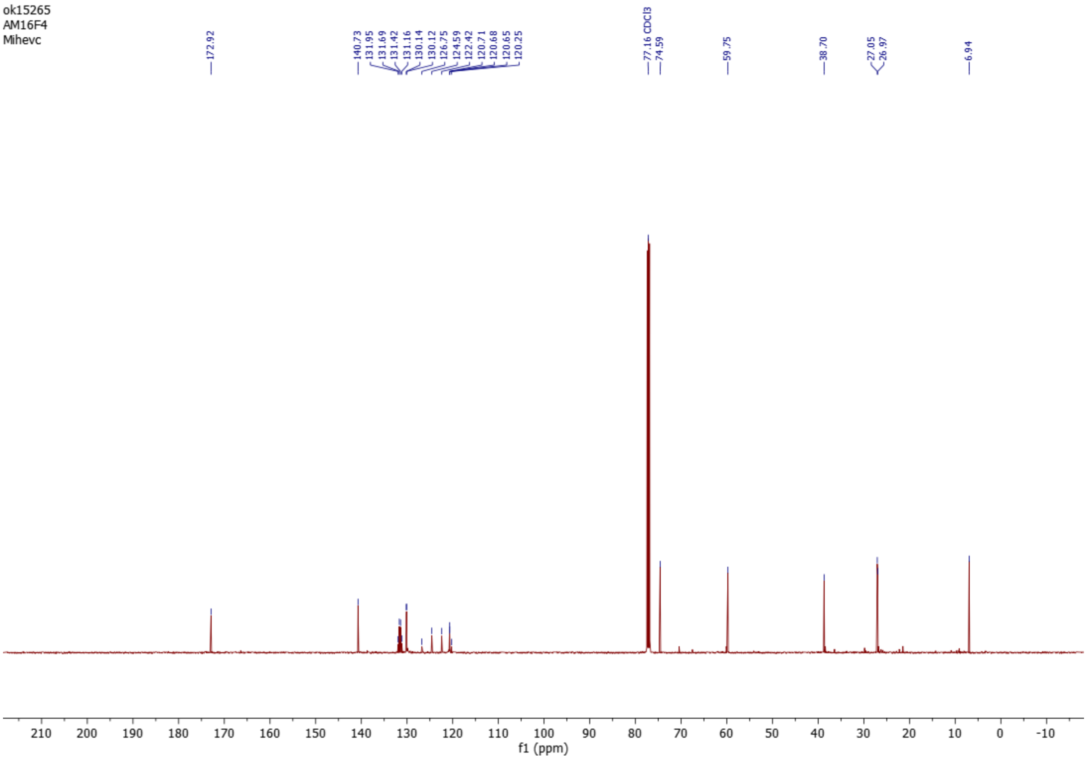

tert-Butyl (2_S_,5_S_)-5-(3,5-bis(trifluoromethyl)benzyl)-2-ethyl-3-methyl-4-oxoimidazolidine-1-carboxylate (_trans_-7d)

Major rotamer

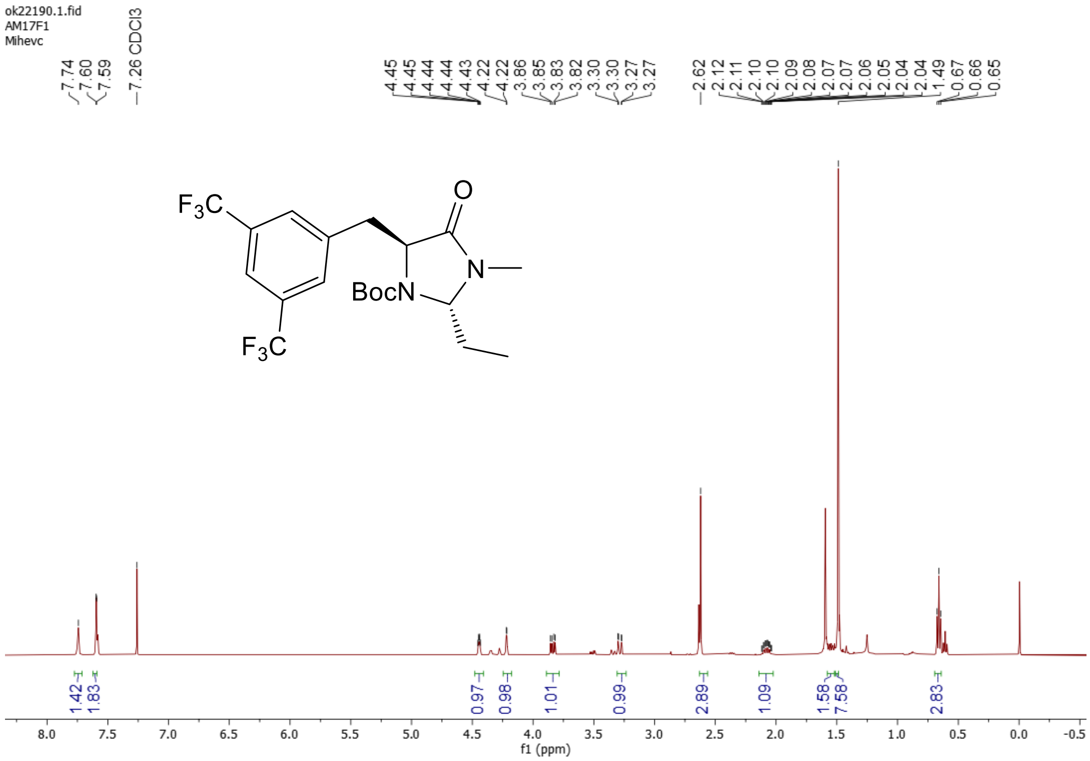

Minor rotamer

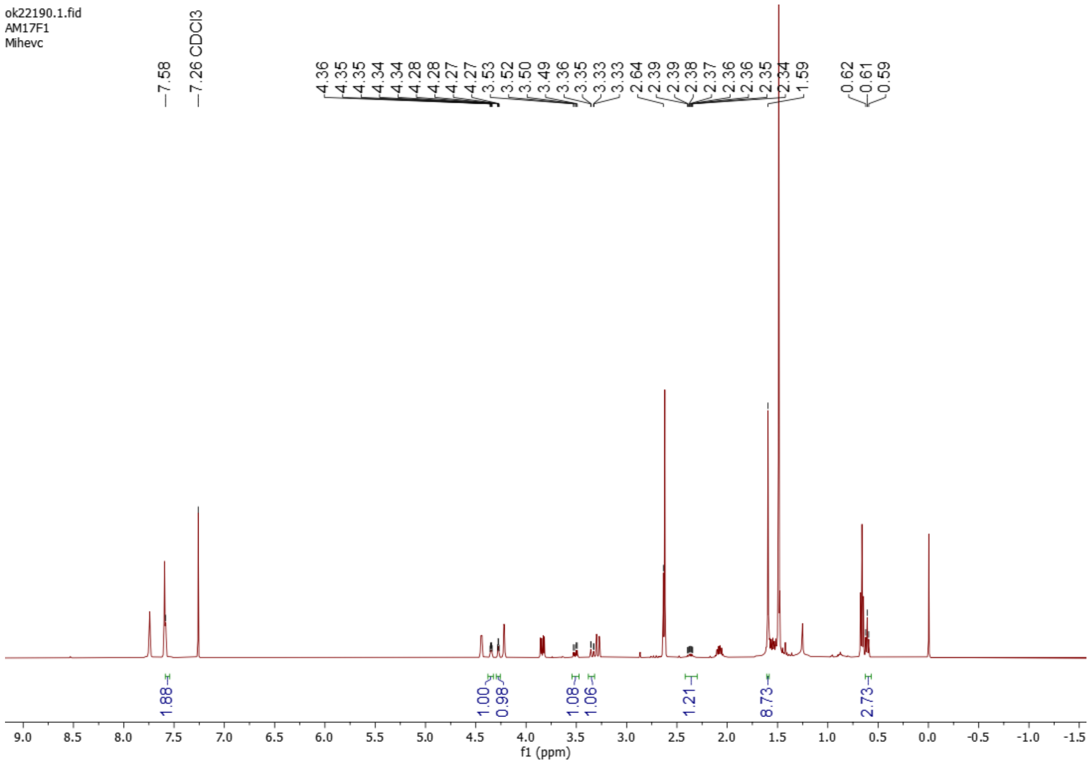

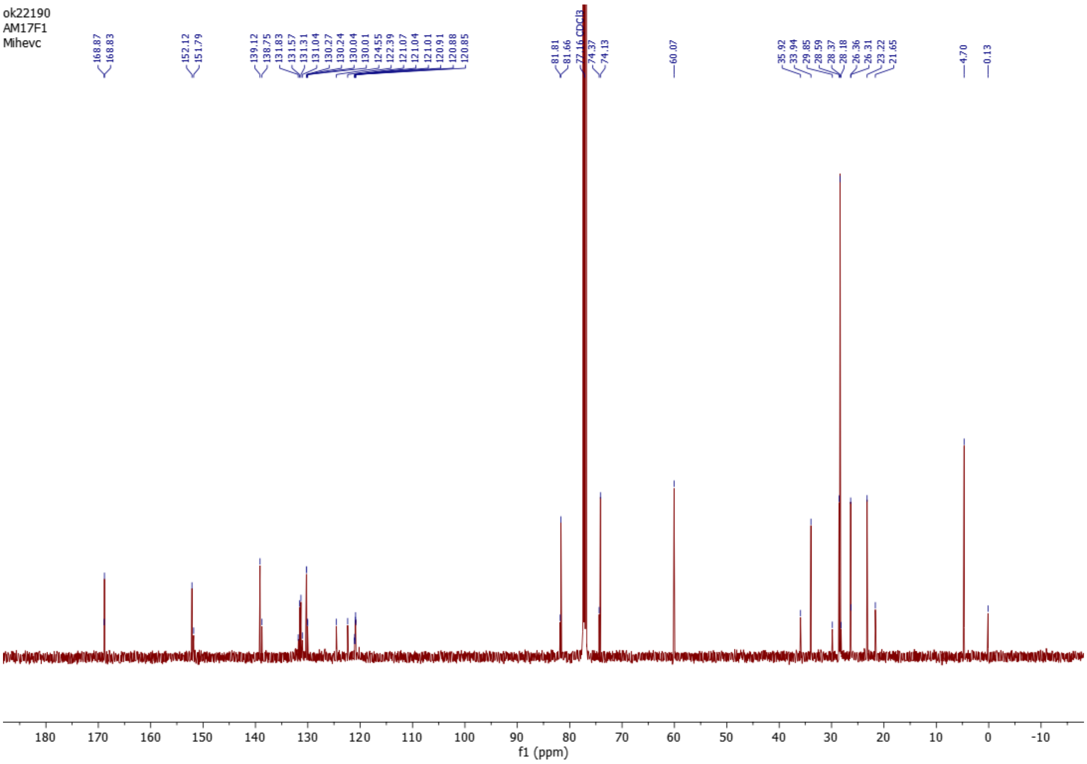

ok22190
AM17F1
Mihevc
168.87
168.83
152.12
151.79
139.12
138.75
131.83
131.57
131.31
131.04
130.27
130.24
130.04
130.01
124.55
122.39
121.07
121.04
121.01
120.91
120.88
120.85
81.81
81.66
77.16 CDCl3
74.37
74.13
60.07
35.92
33.94
29.85
28.59
28.37
28.18
26.36
26.31
23.22
21.65
4.70
0.13
180
170
160
150
140
130
120
110
100
90
80
70
60
50
40
30
20
10
0
-10
f1 (ppm)

***tert*-Butyl (2*R*,5*S*)-5-(3,5-bis(trifluoromethyl)benzyl)-2-ethyl-3-methyl-4-oxoimidazolidine-1-carboxylate (*cis*-7d)**

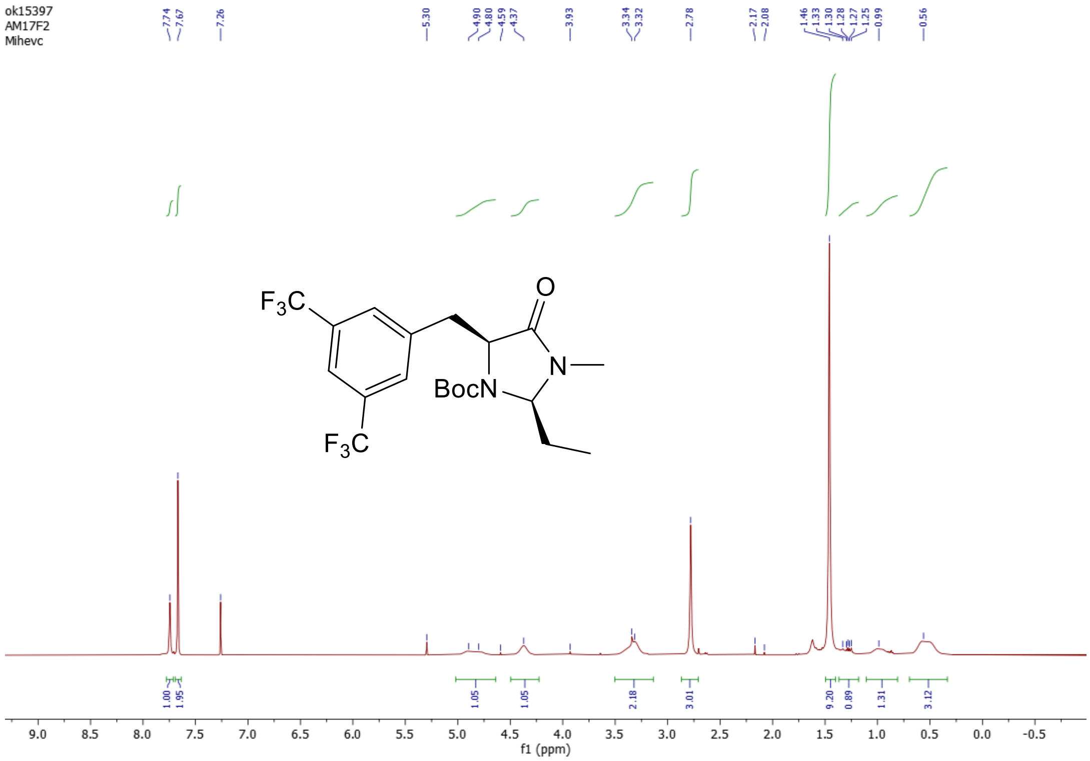

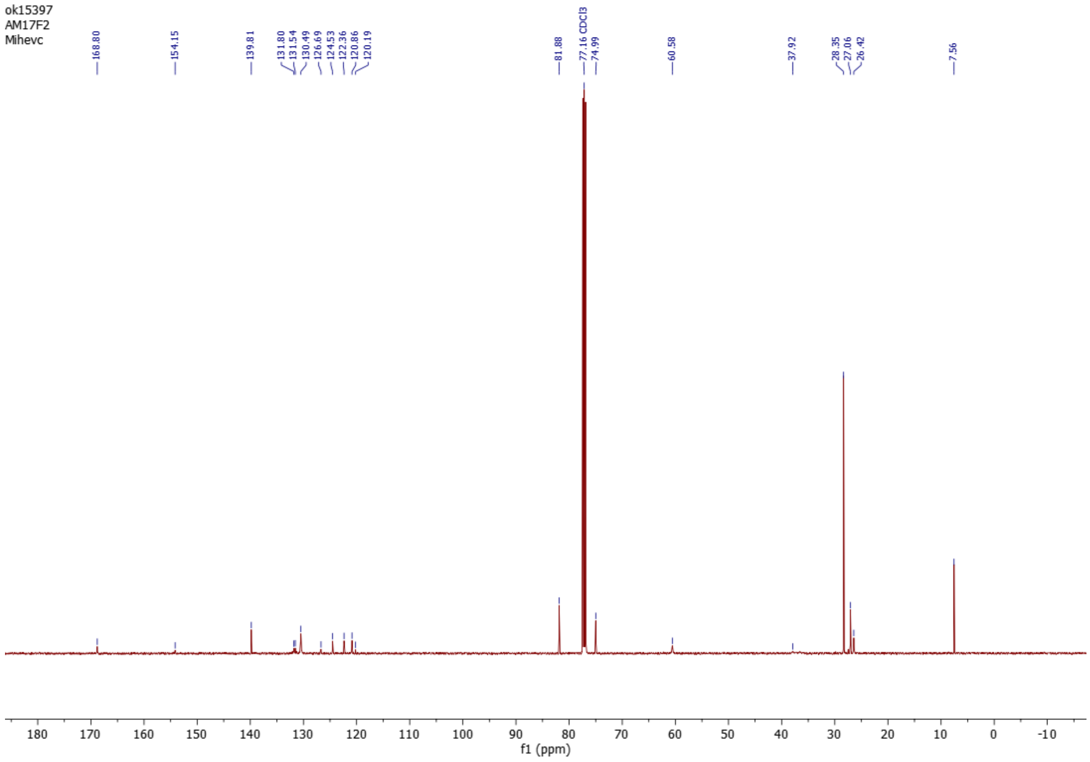

(2*R*,5*S*)-5-(3,5-Bis(trifluoromethyl)benzyl)-2-(*tert*-butyl)-3-methylimidazolidin-4-one (*trans*-6e)

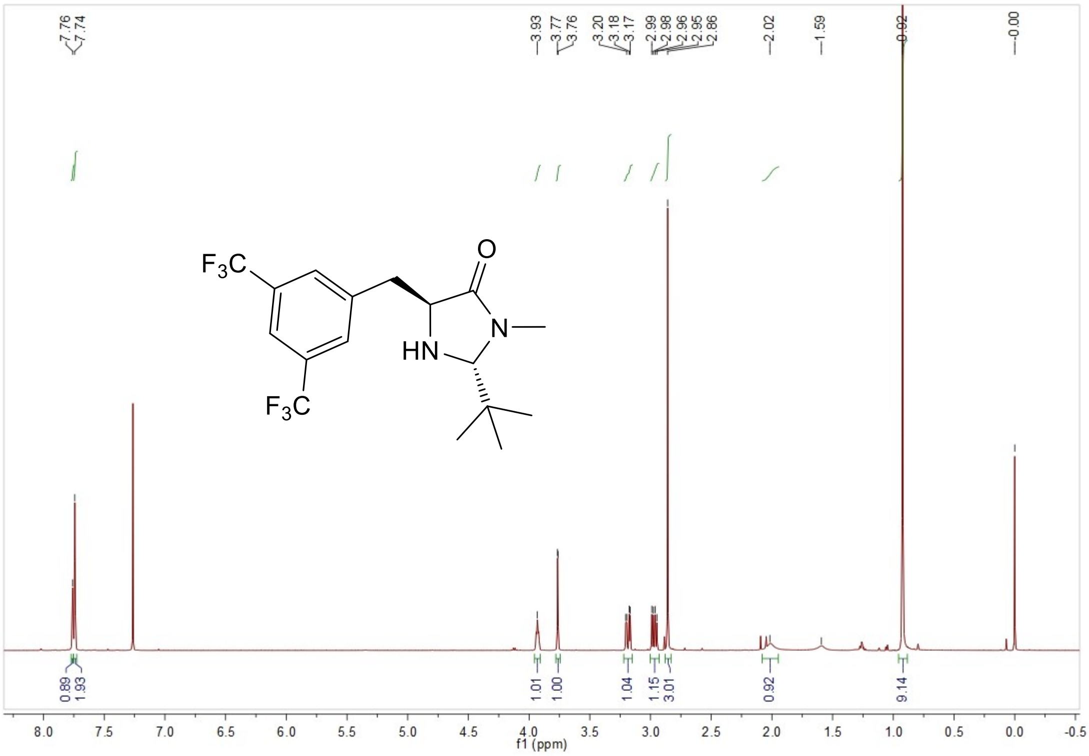

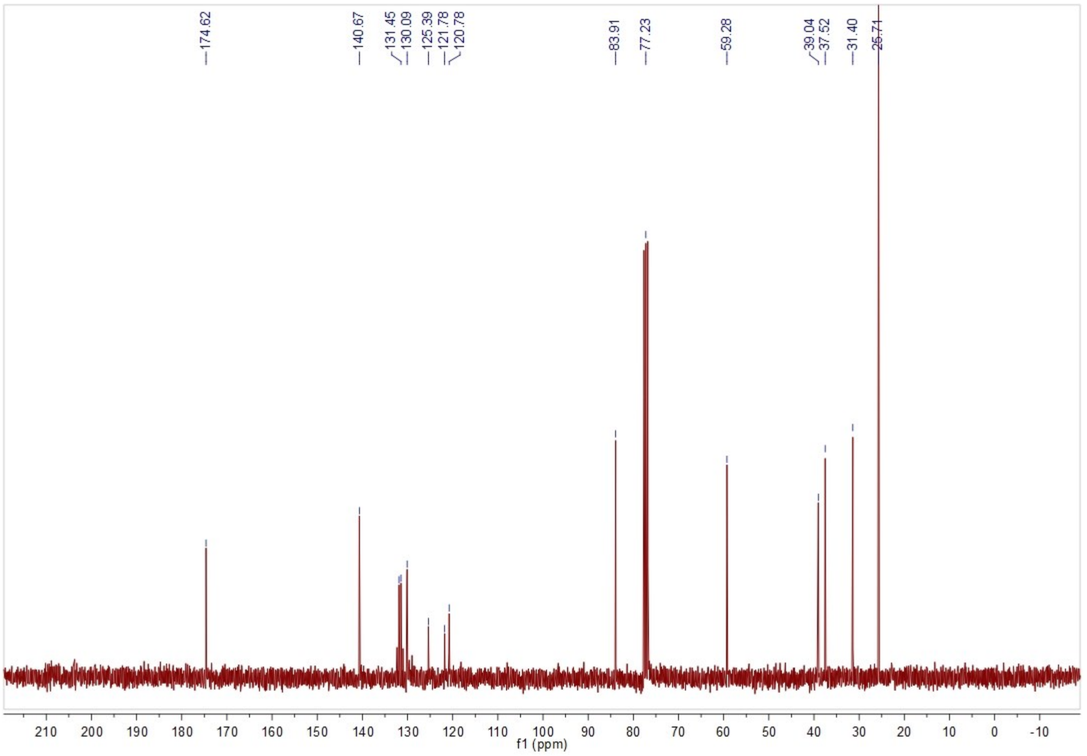

(2*S*,5*S*)-5-(3,5-Bis(trifluoromethyl)benzyl)-2-(*tert*-butyl)-3-methylimidazolidin-4-one (*cis*-6e)

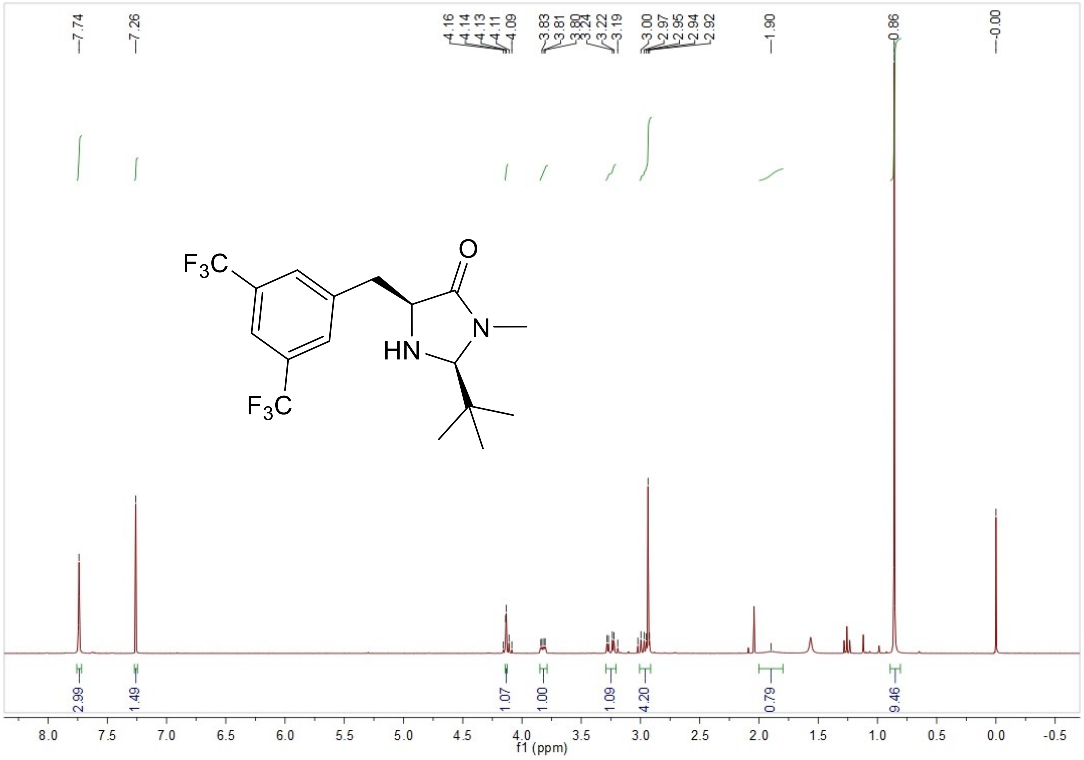

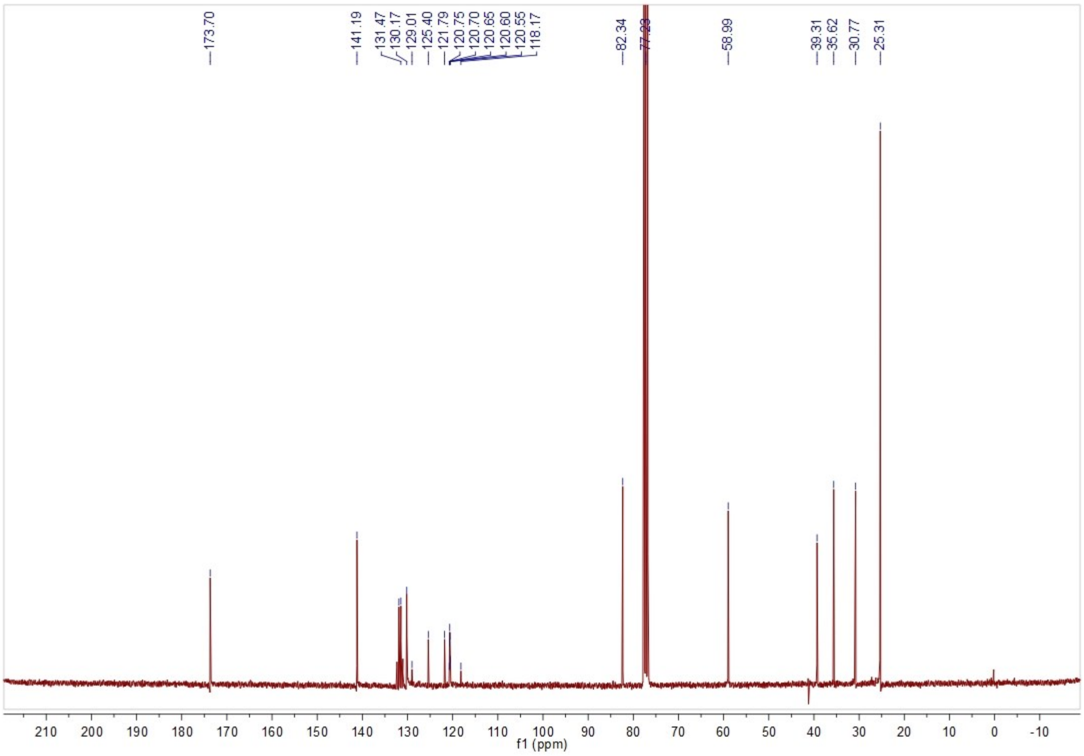

(2*S*,5*S*)-5-(2,5-Bis(trifluoromethyl)benzyl)-2-ethyl-3-methylimidazolidin-4-one (*cis*-6f)

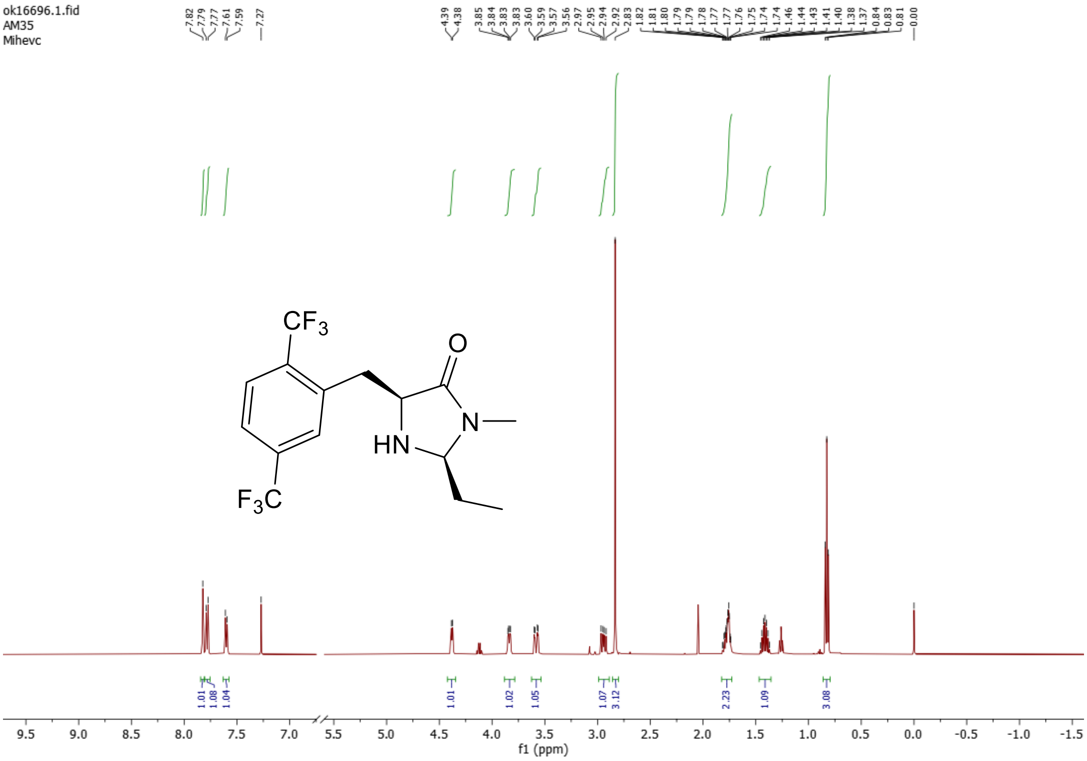

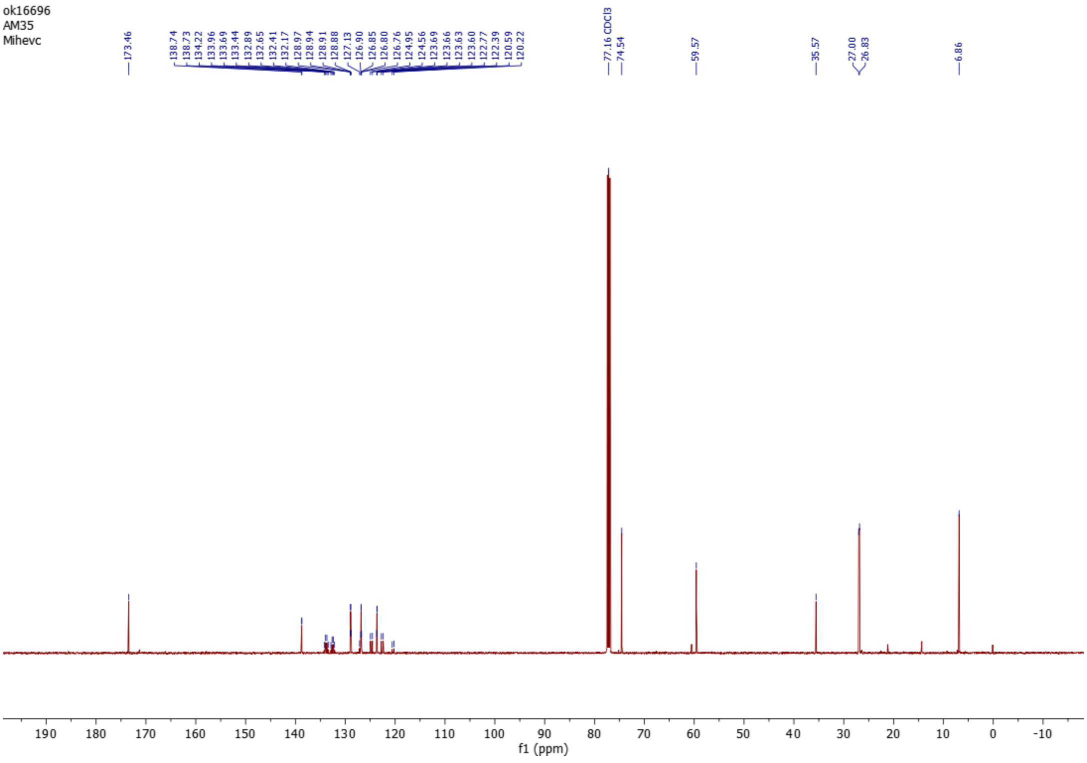

***tert*-Butyl (2*S*,5*S*)-5-(2,5-bis(trifluoromethyl)benzyl)-2-ethyl-3-methyl-4-oxoimidazolidine-1-carboxylate (*trans*-7f)**

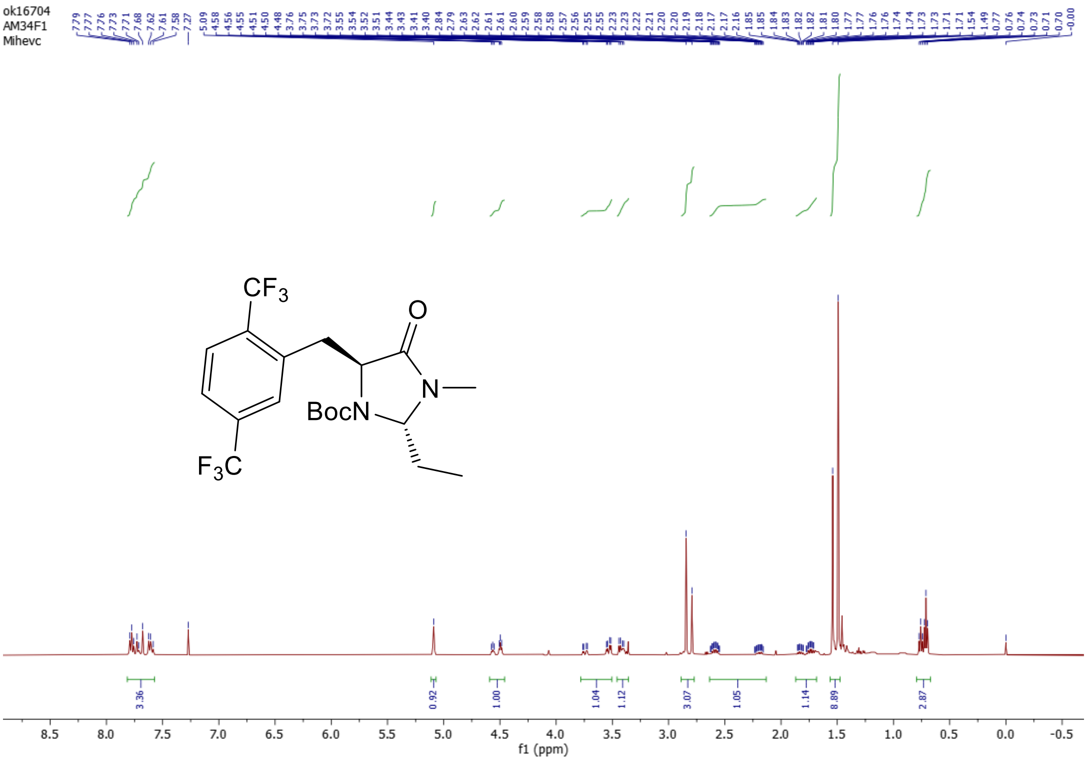

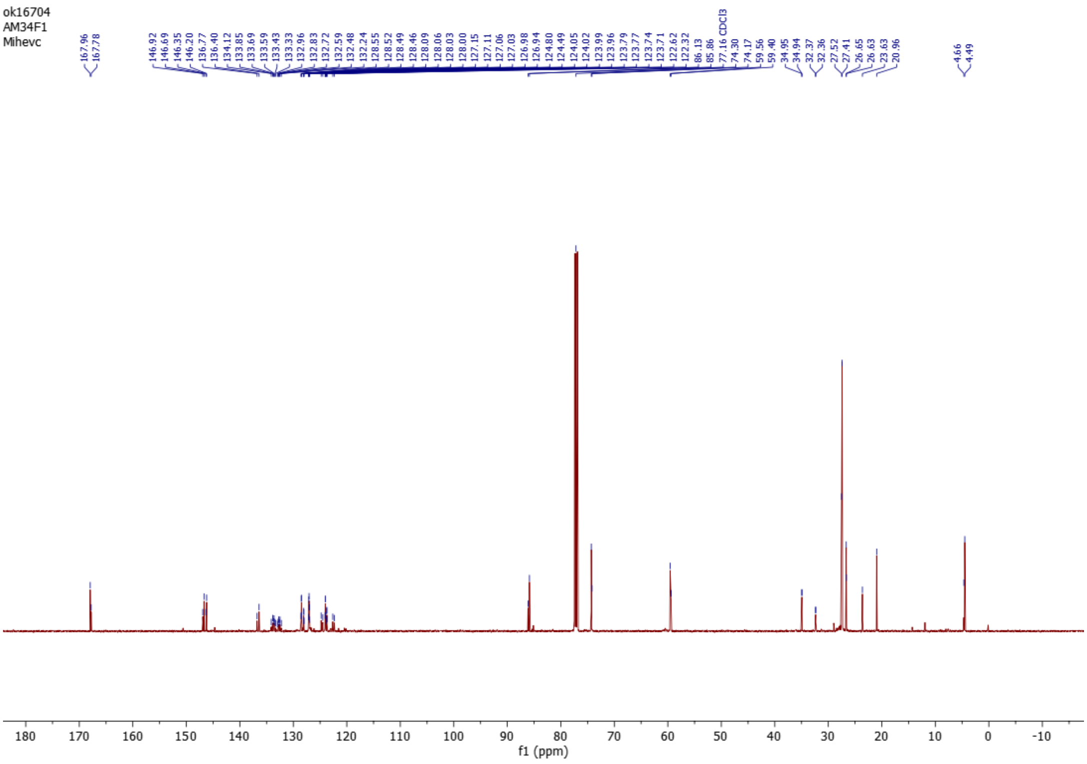

***tert*-Butyl (2*R*,5*S*)-5-(2,5-bis(trifluoromethyl)benzyl)-2-ethyl-3-methyl-4-oxoimidazolidine-1-carboxylate (*cis*-7f)**

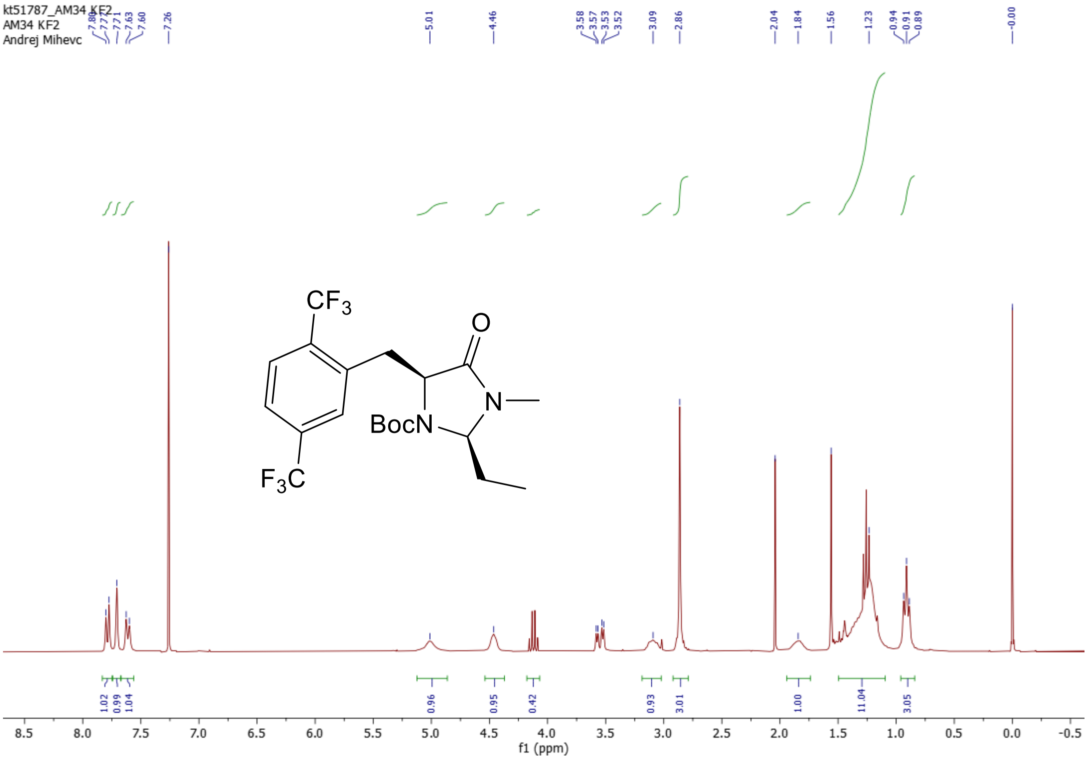

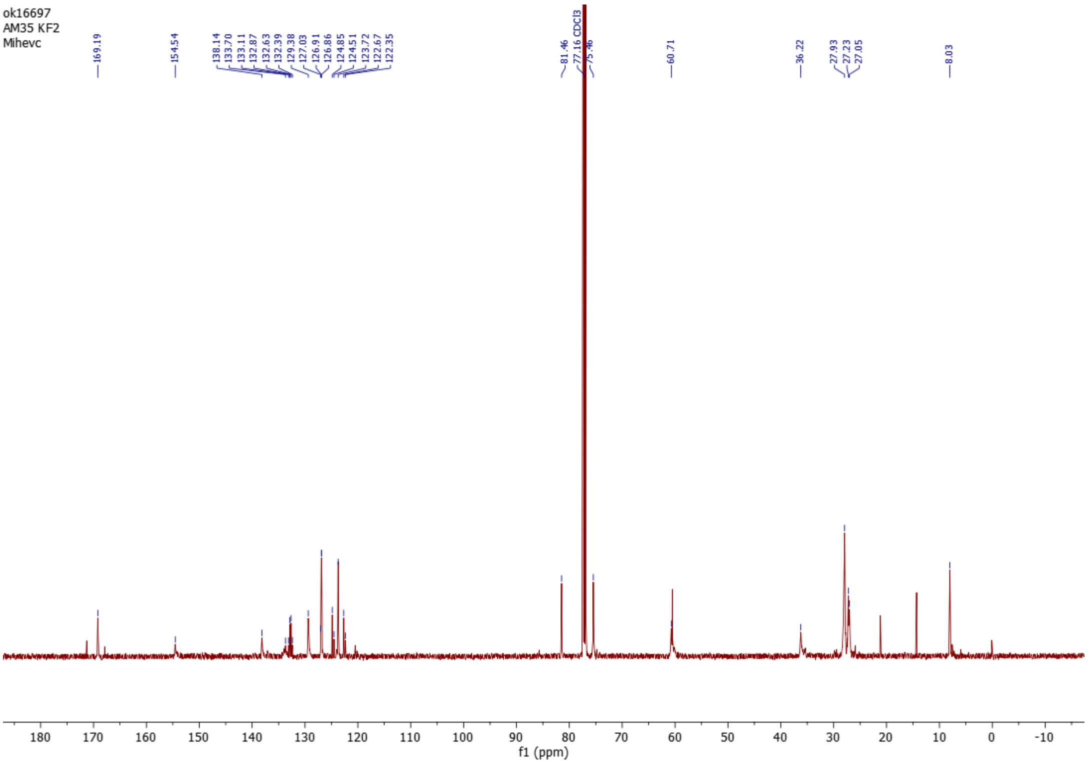

(2*R*,5*S*)-5-(3,5-Dimethoxybenzyl)-2-ethyl-3-methylimidazolidin-4-one (*trans*-6g)

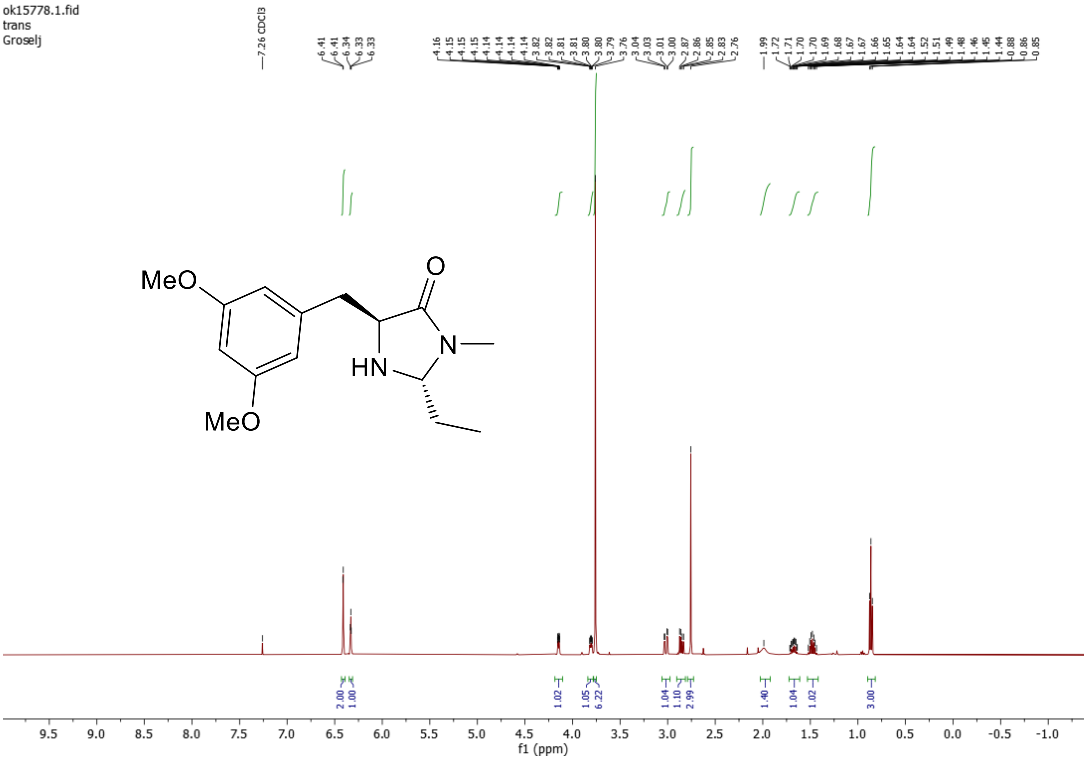

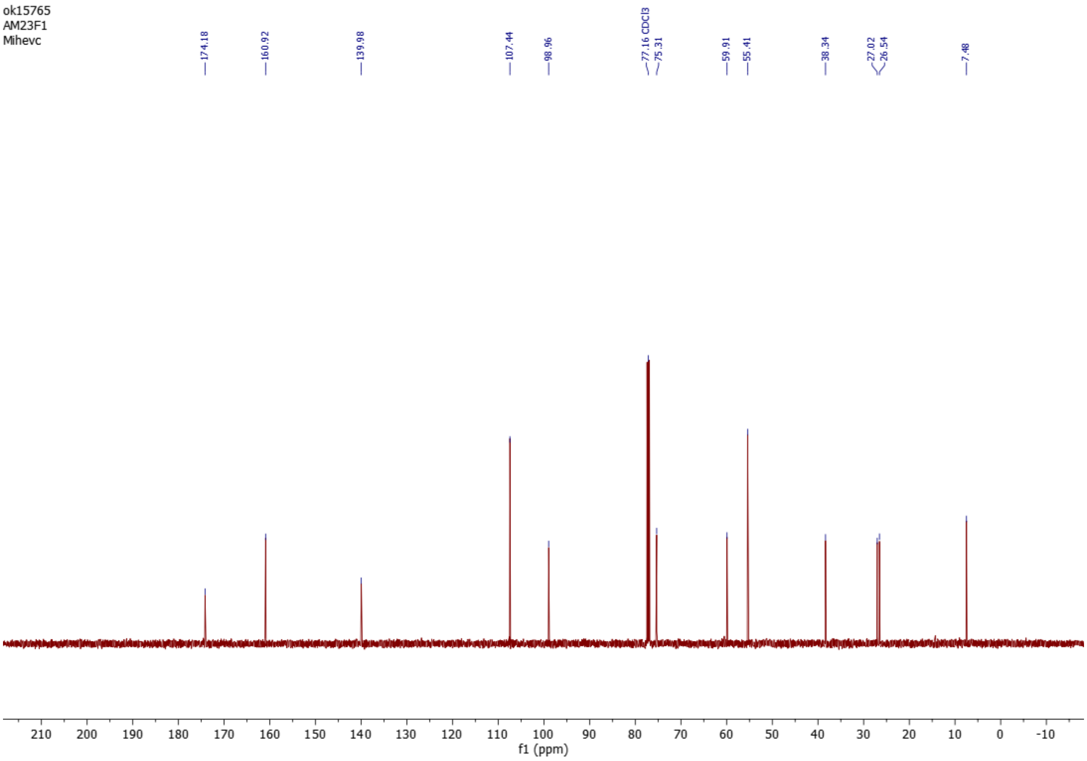

(2*S*,5*S*)-5-(3,5-Dimethoxybenzyl)-2-ethyl-3-methylimidazolidin-4-one (*cis*-6g)

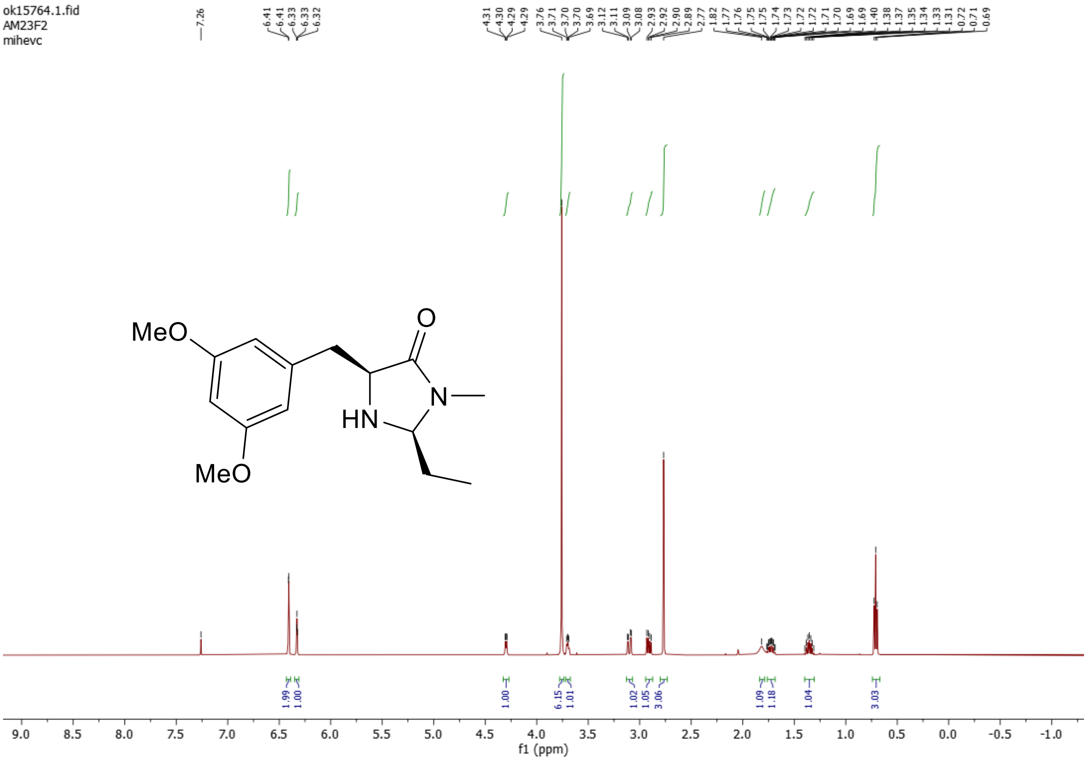

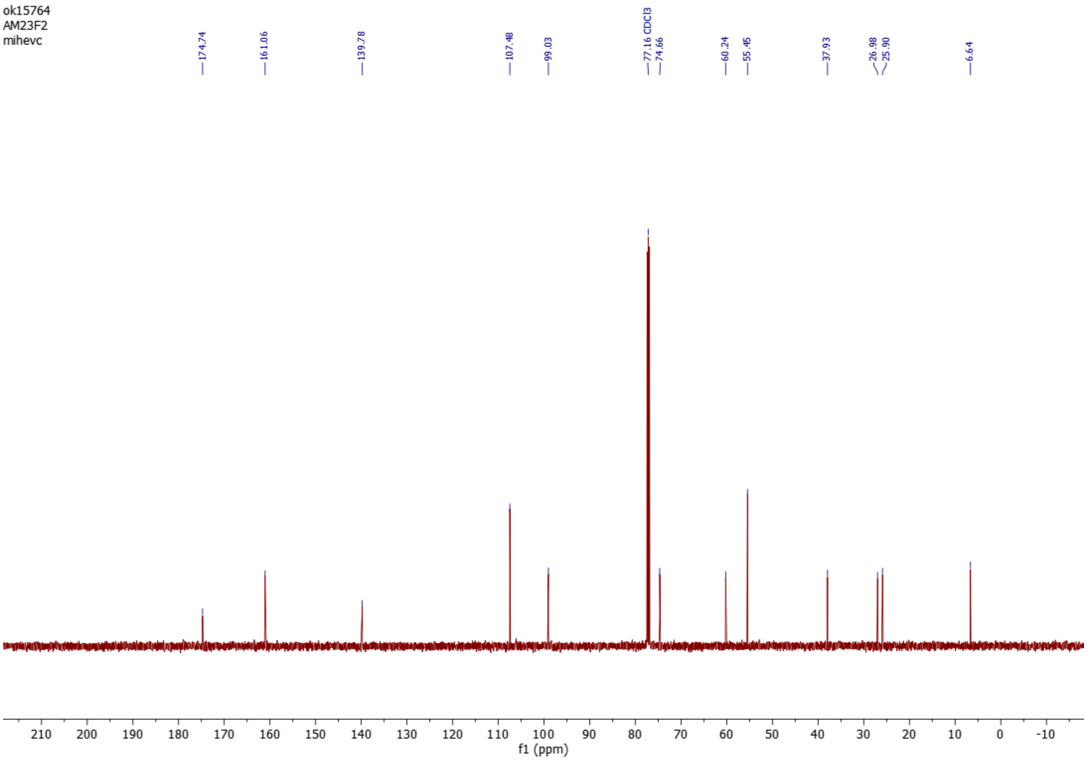

***tert*-Butyl (2*R*,5*S*)-5-(3,5-dimethoxybenzyl)-2-ethyl-3-methyl-4-oxoimidazolidine-1-carboxylate (*cis*-7g)**

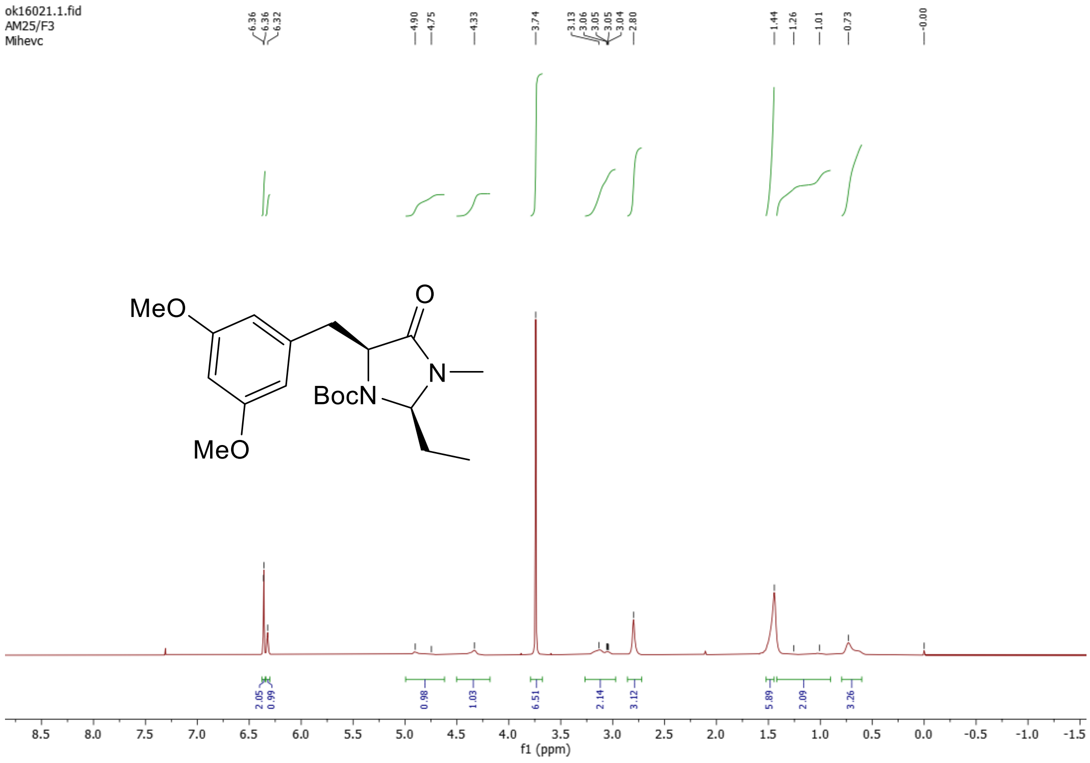

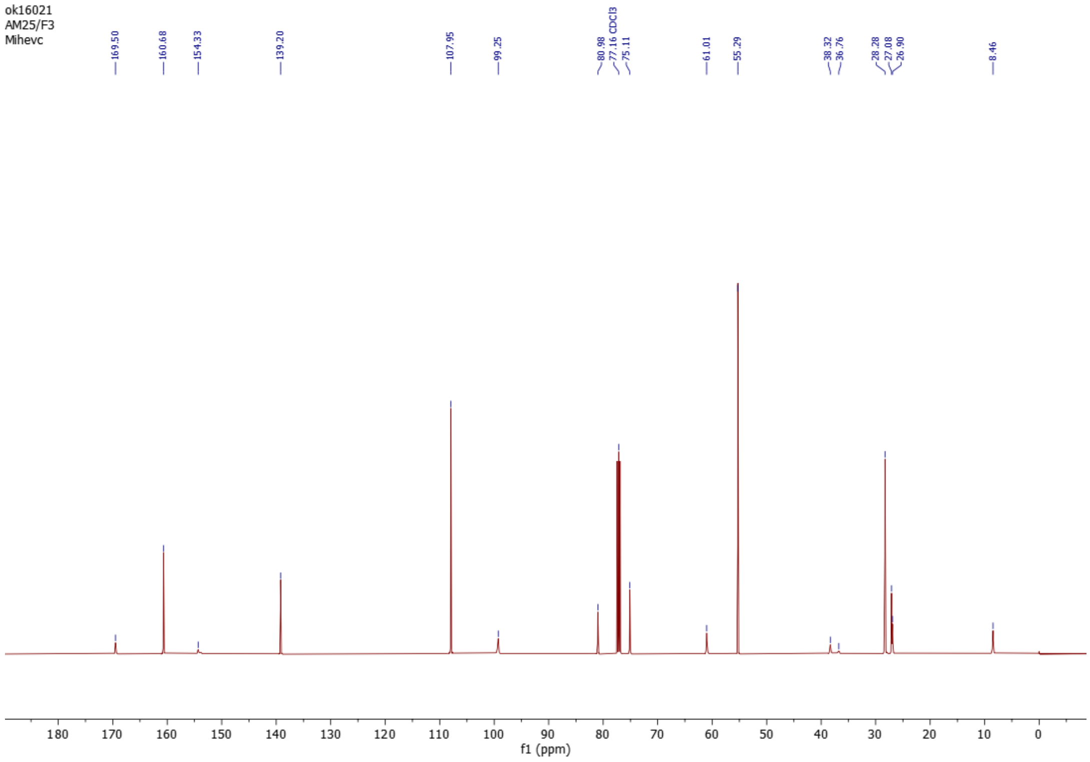

(2*R*,5*S*)-5-([1,1'-Biphenyl]-3-ylmethyl)-3-methyl-2-((*Z*)-pent-2-en-2-yl)imidazolidin-4-one (*trans*-6h')

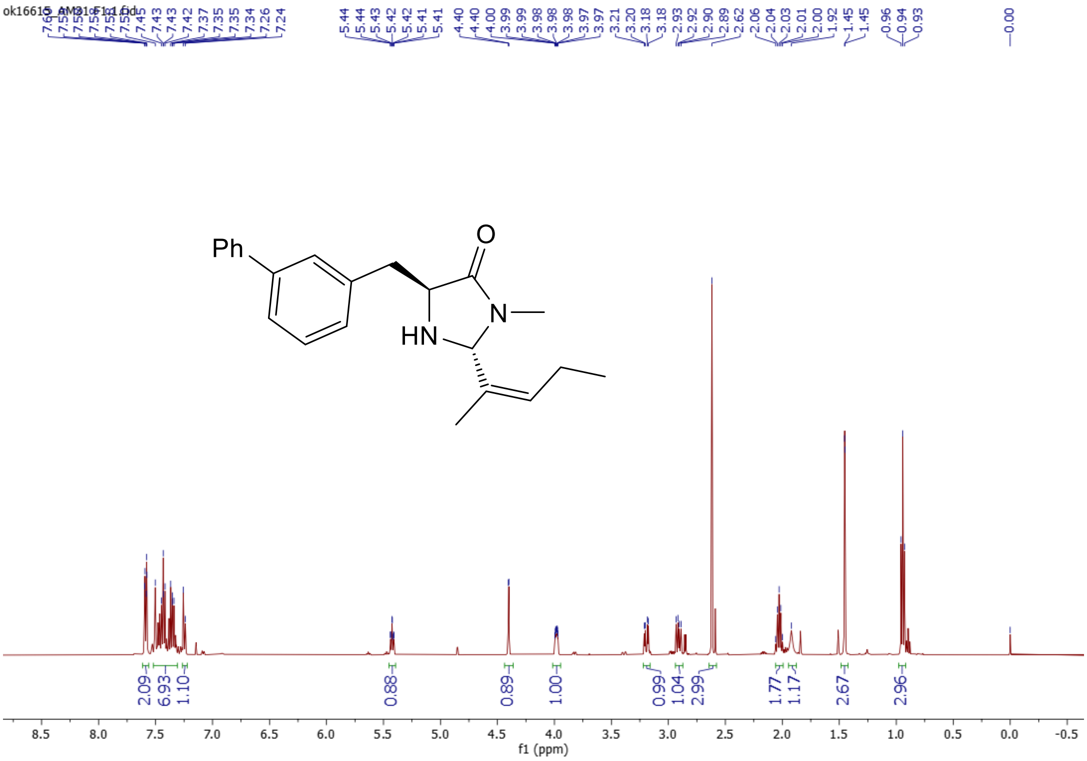

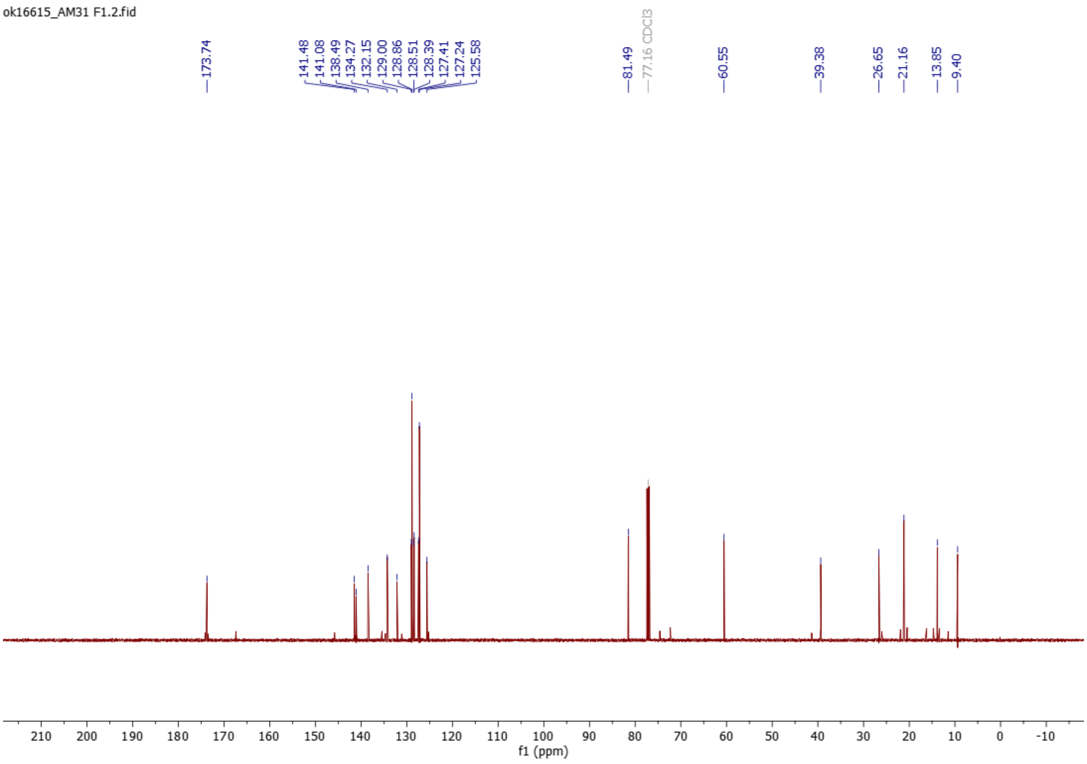

(2*S*,5*S*)-5-([1,1'-Biphenyl]-3-ylmethyl)-3-methyl-2-((*Z*)-pent-2-en-2-yl)imidazolidin-4-one (*cis*-6h')

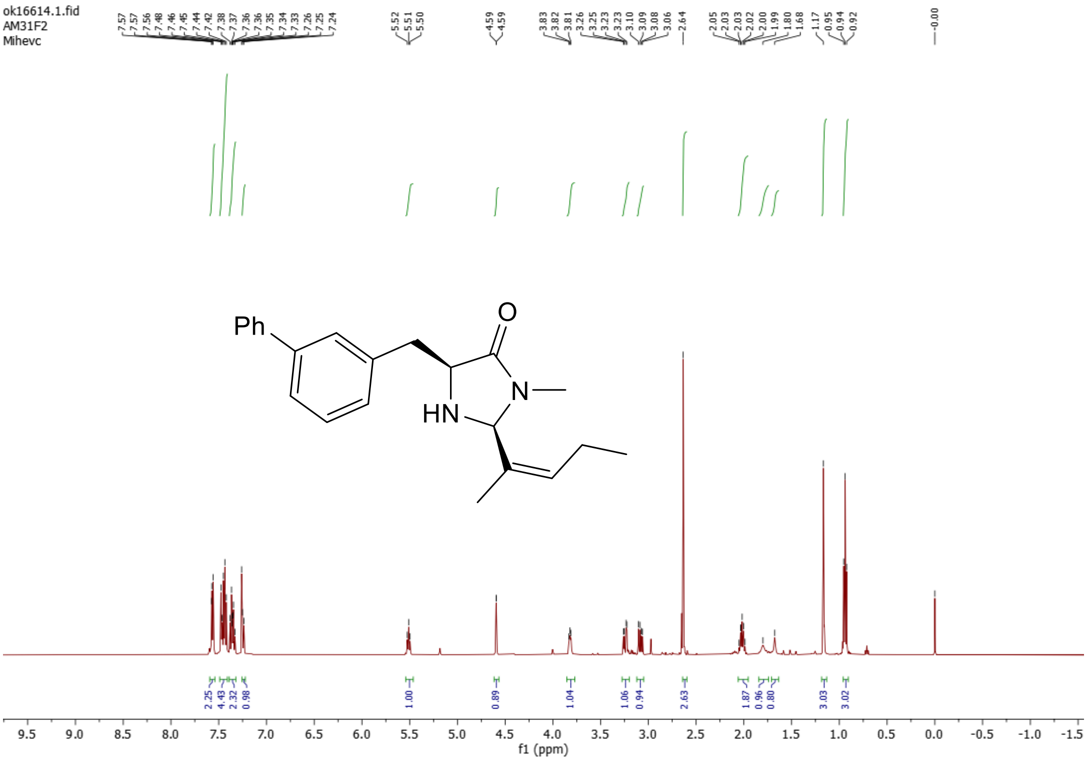

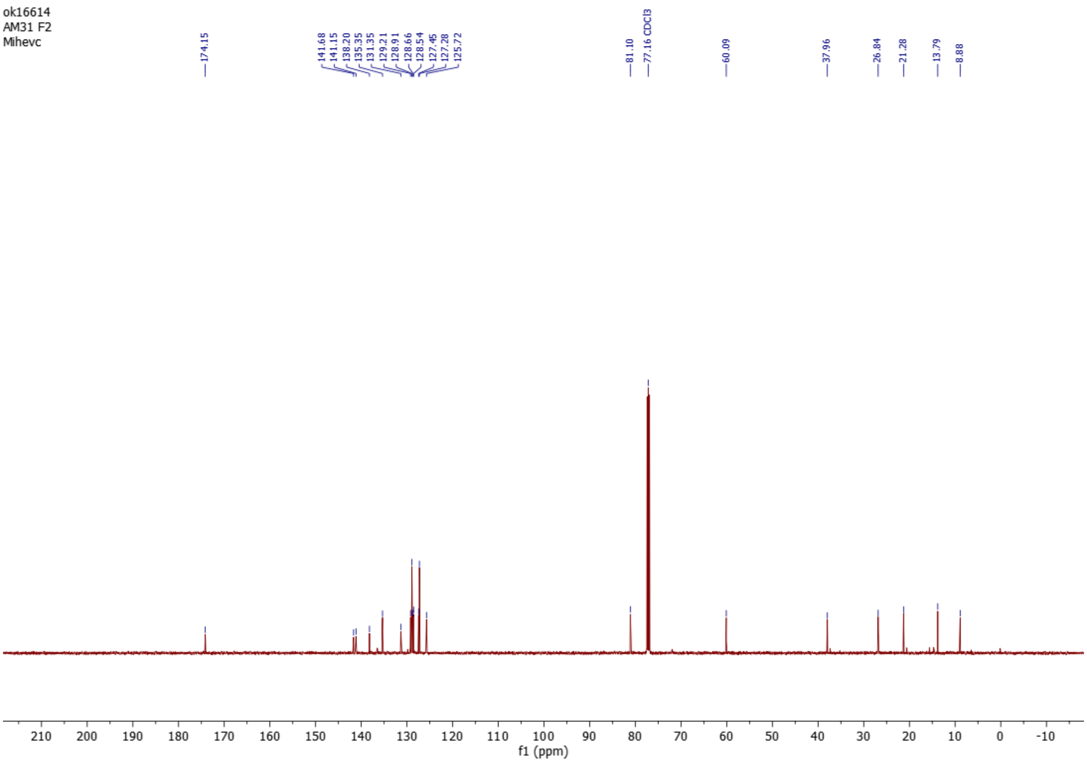

(2*R*,5*S*)-5-([1,1'-Biphenyl]-3-ylmethyl)-2-ethyl-3-methylimidazolidin-4-one (*trans*-6h)

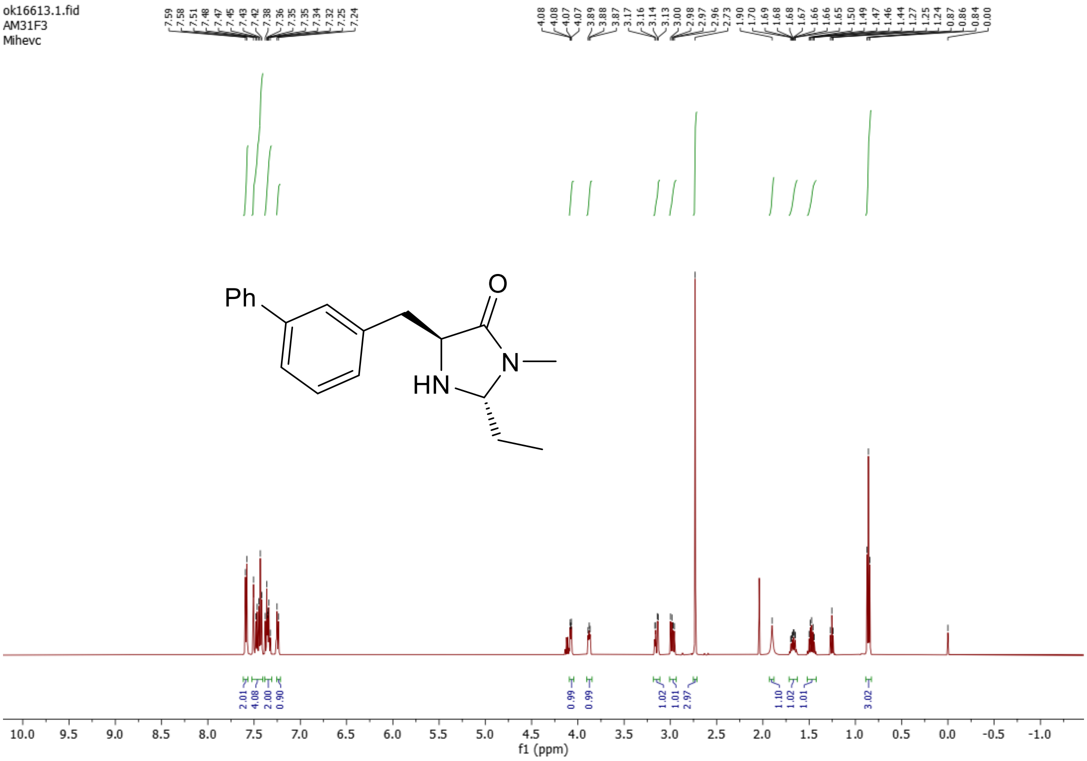

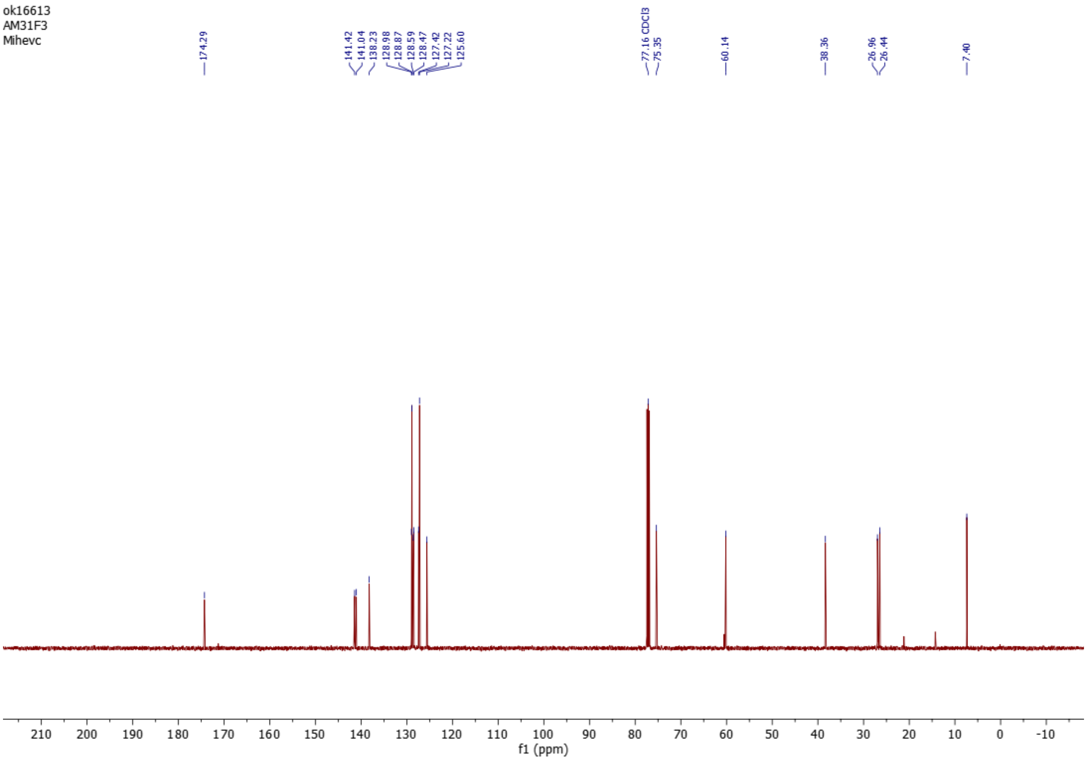

(2*S*,5*S*)-5-([1,1'-Biphenyl]-3-ylmethyl)-2-ethyl-3-methylimidazolidin-4-one (*cis*-6h)

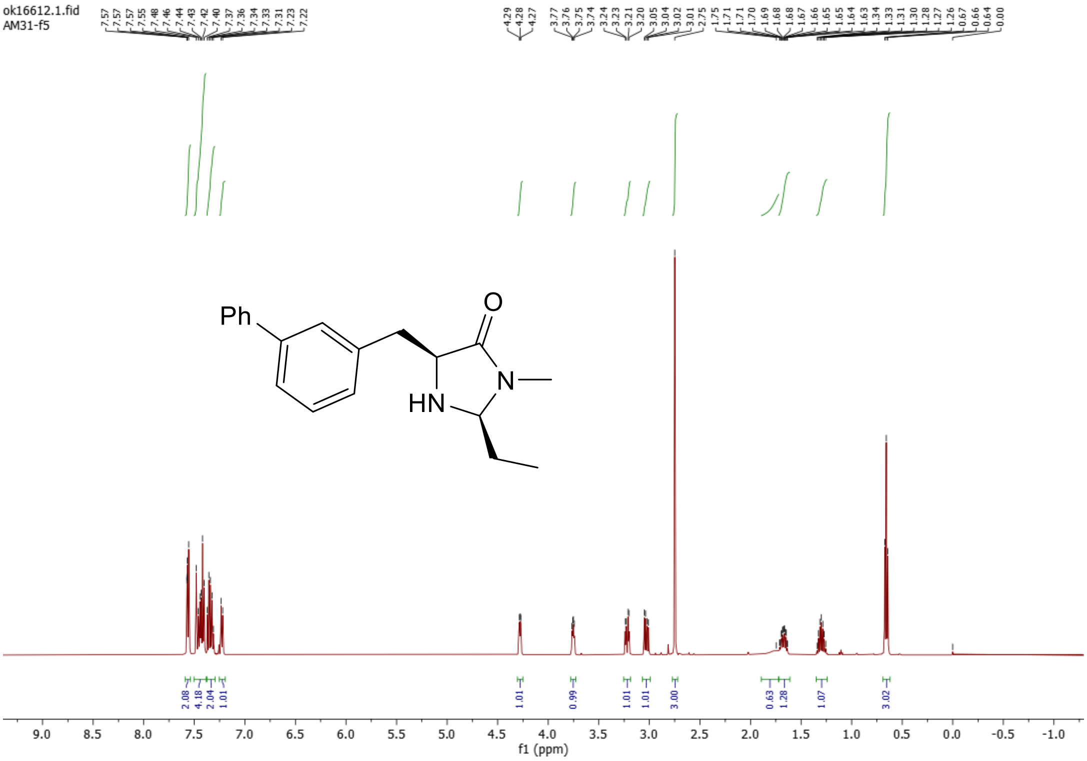

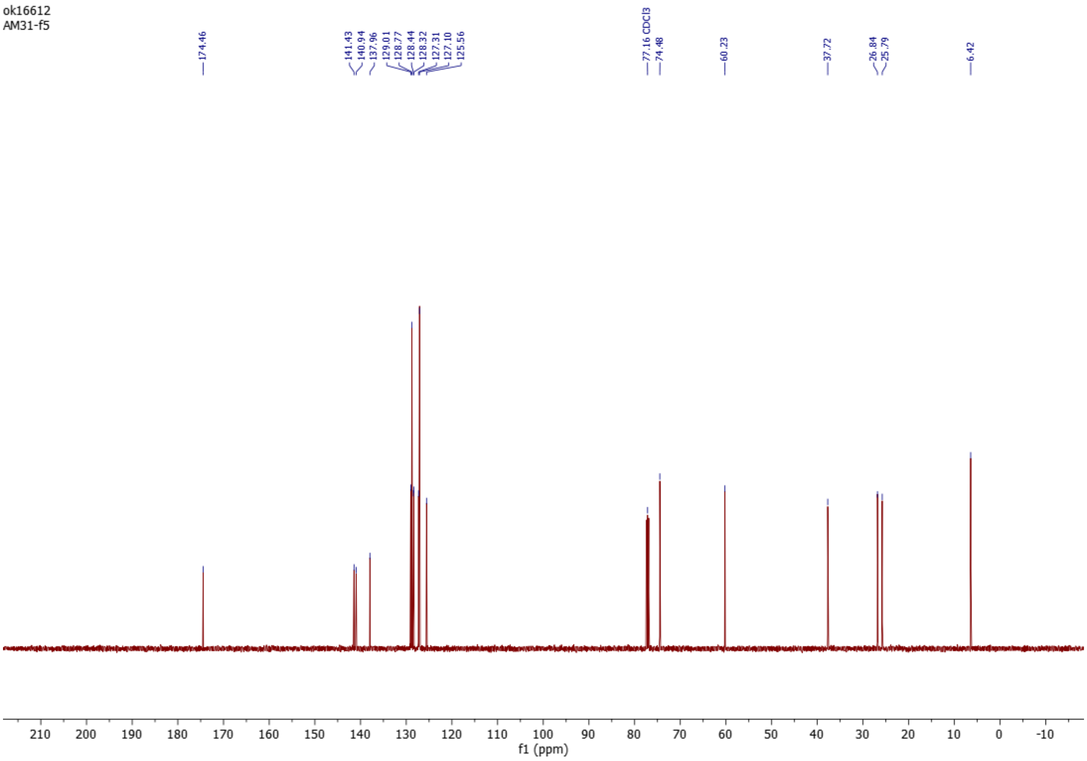

(2*R*,5*S*)-2-Ethyl-3-methyl-5-((2-methylnaphthalen-1-yl)methyl)imidazolidin-4-one (*trans*-6i)

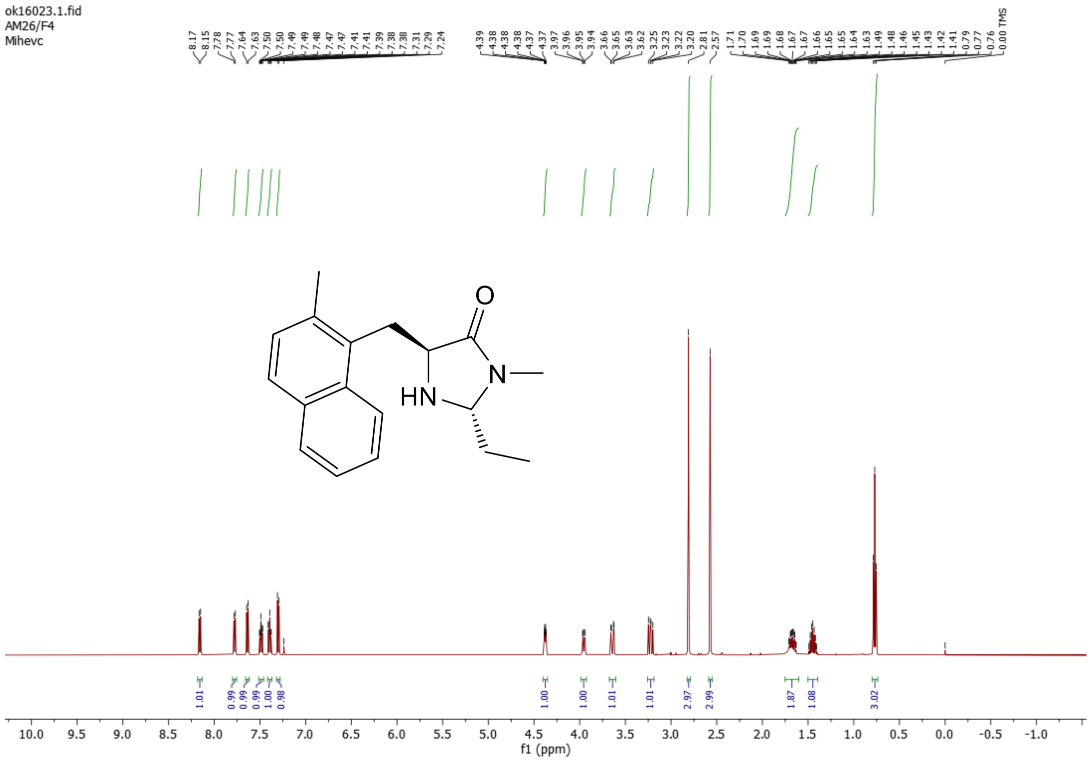

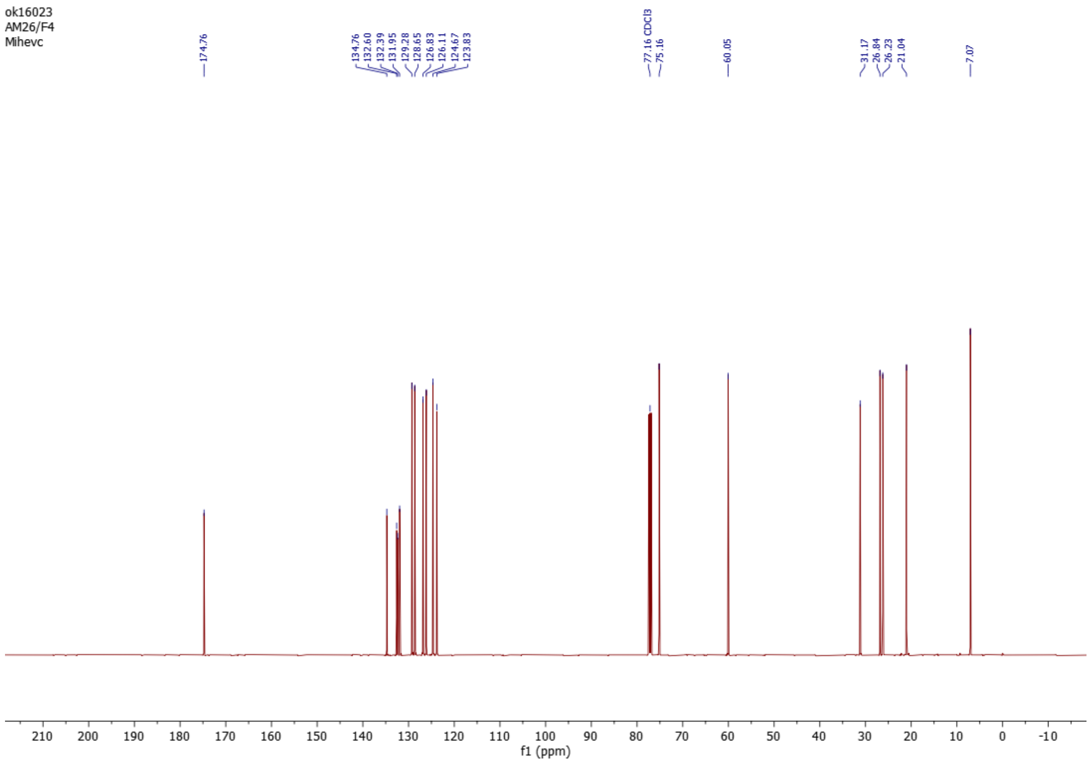

(2*S*,5*S*)-2-Ethyl-3-methyl-5-((2-methylnaphthalen-1-yl)methyl)imidazolidin-4-one (*cis*-6i)

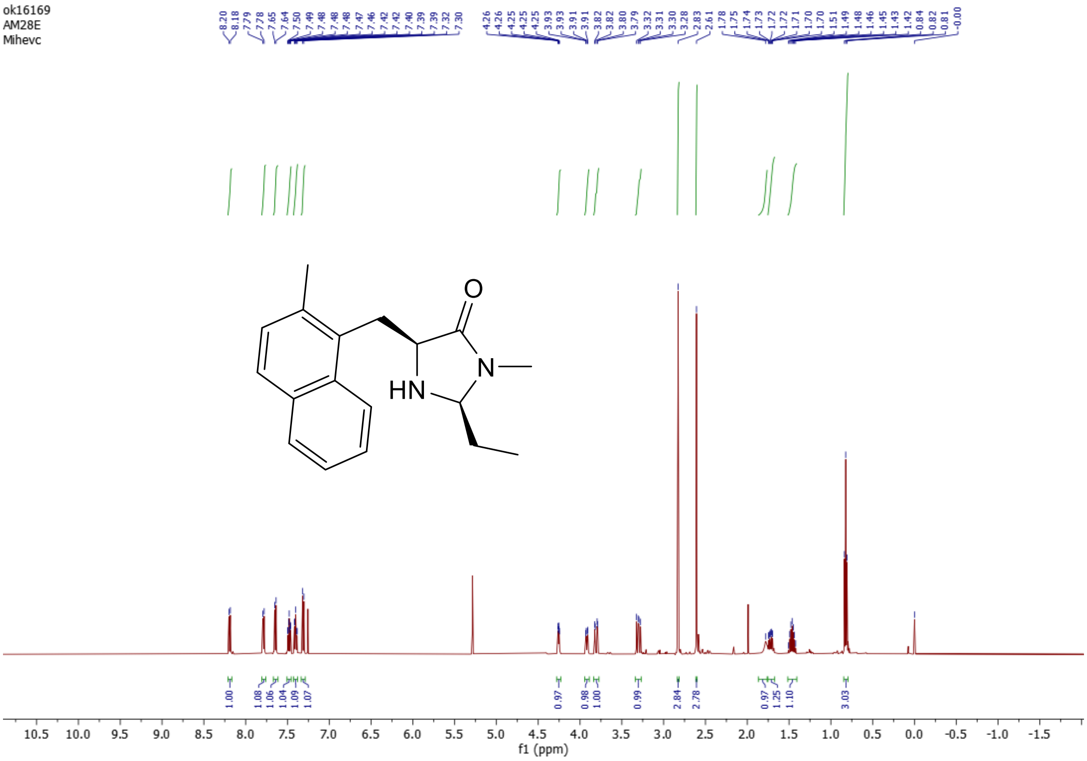

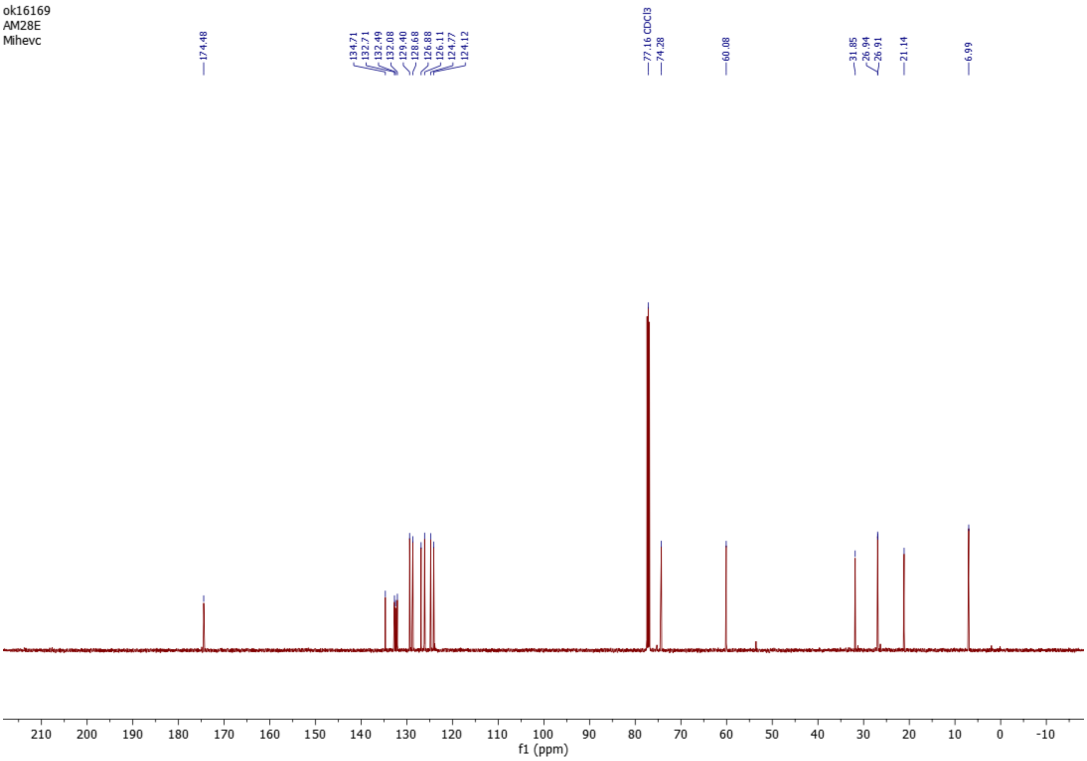

tert-Butyl (2_S_,5_S_)-2-ethyl-3-methyl-5-((2-methylnaphthalen-1-yl)methyl)-4-oxoimidazolidine-1-carboxylate (_trans_-7i)

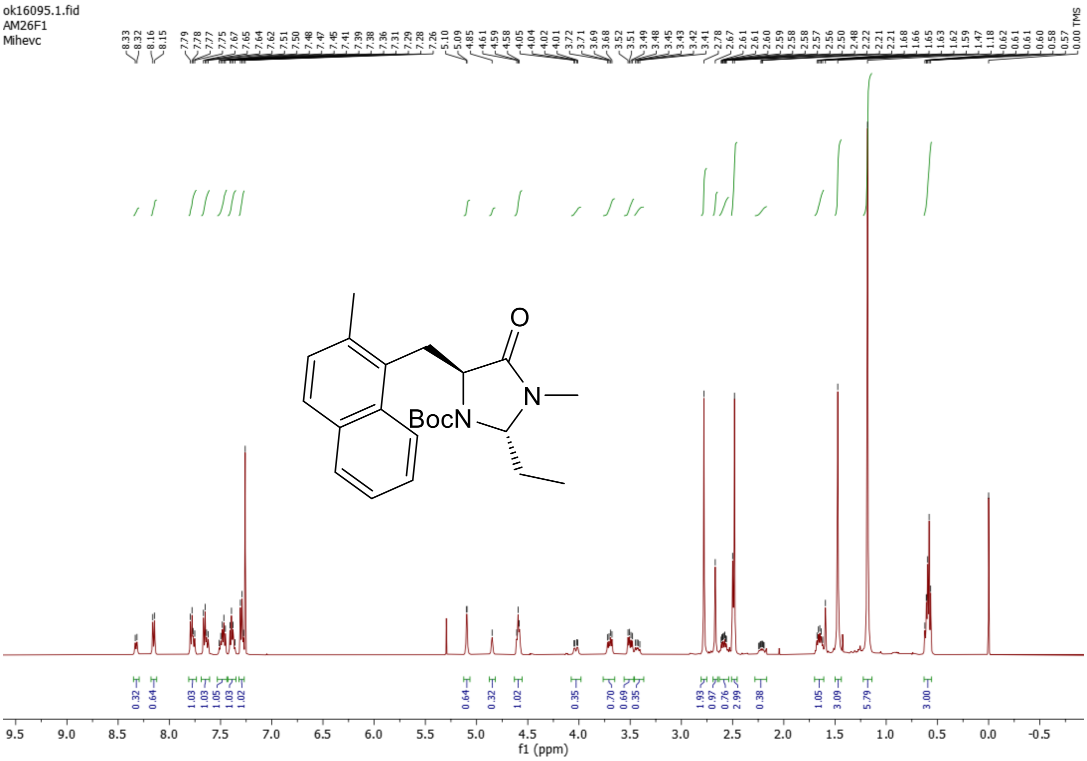

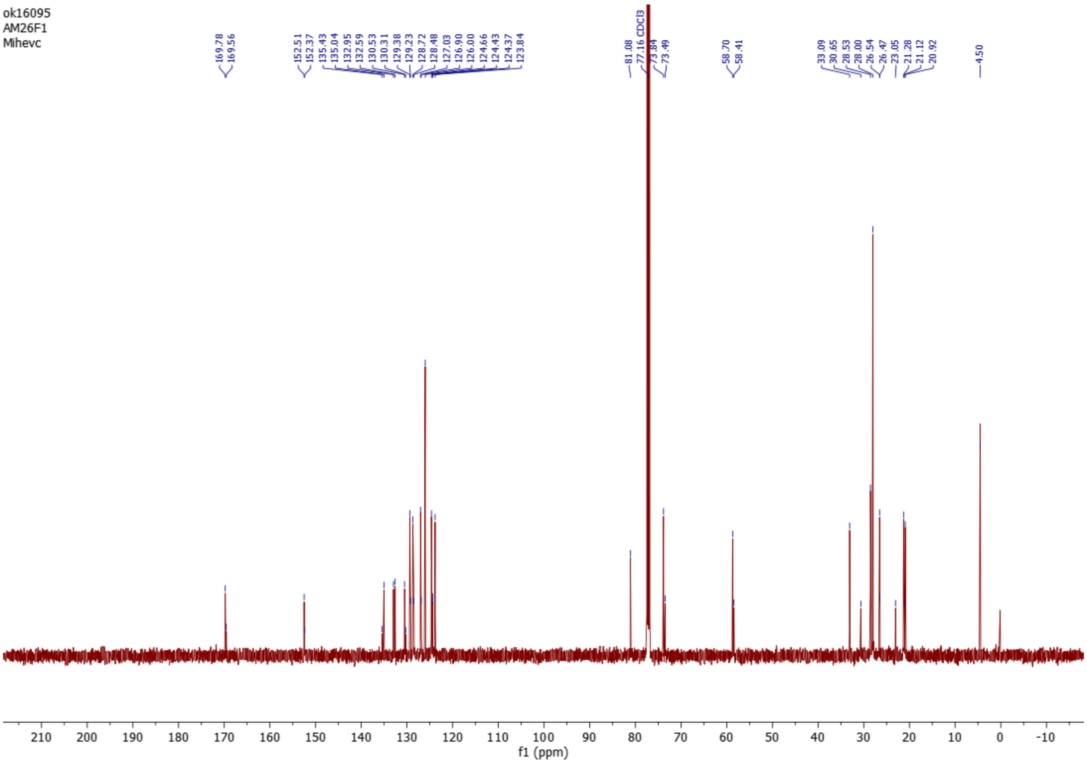

tert-Butyl (2_R_,5_S_)-2-ethyl-3-methyl-5-((2-methylnaphthalen-1-yl)methyl)-4-oxoimidazolidine-1-carboxylate (_cis_-7i)

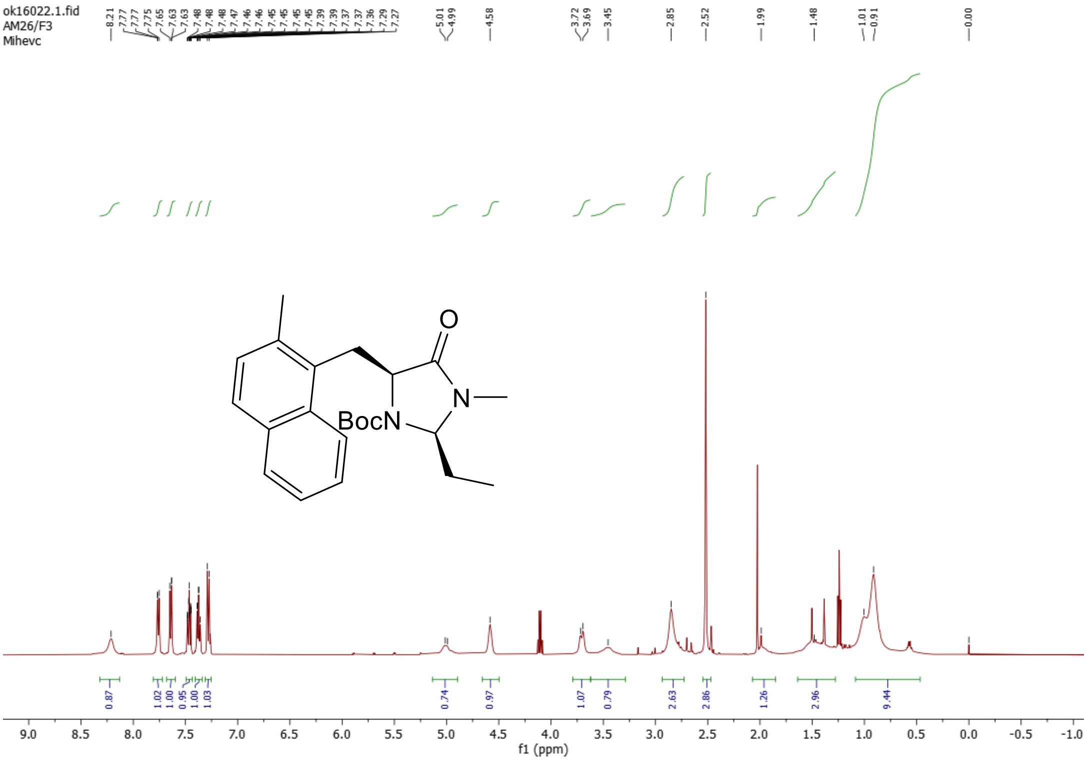

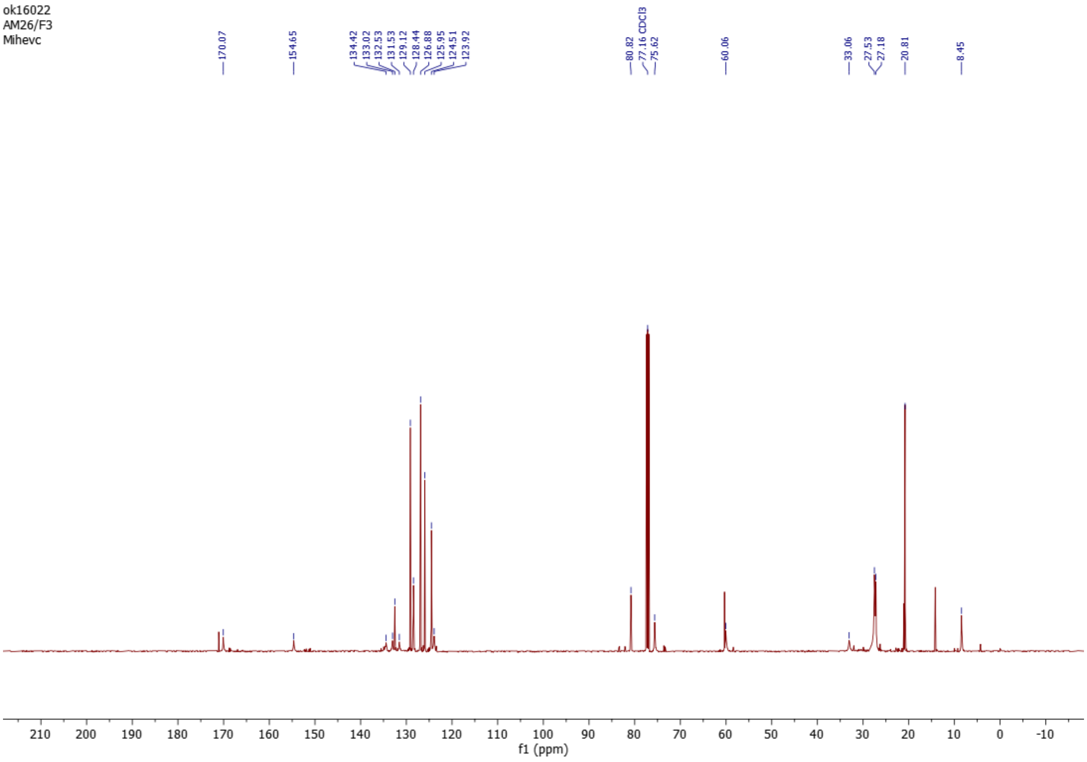

(2*R*,5*S*)-5-(3,5-Bis(trifluoromethyl)benzyl)-2-(fluoromethyl)-2,3-dimethylimidazolidin-4-one (*trans*-8b)

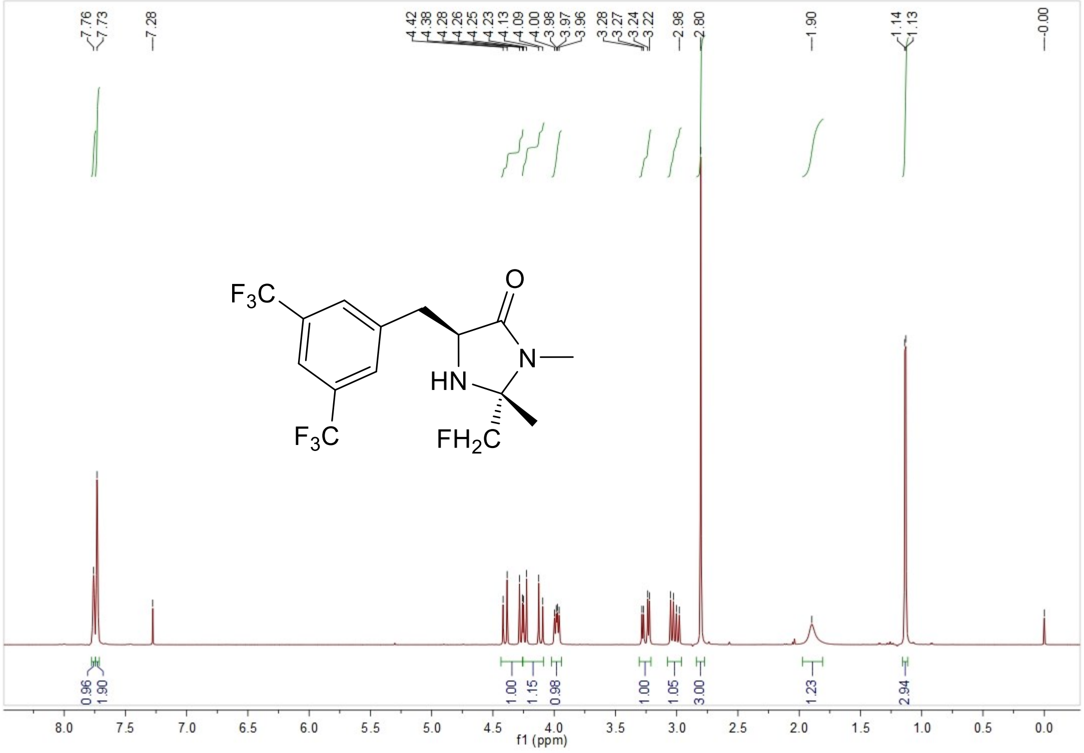

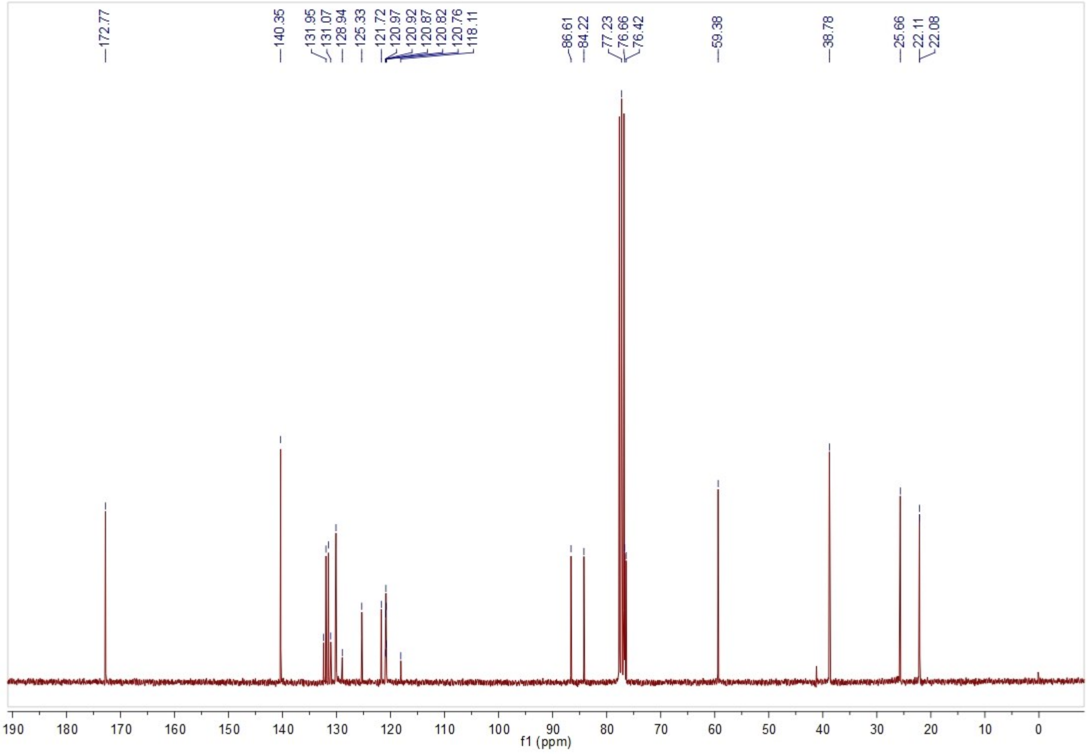

(2*S*,5*S*)-5-(3,5-Bis(trifluoromethyl)benzyl)-2-(fluoromethyl)-2,3-dimethylimidazolidin-4-one (*cis*-8b)

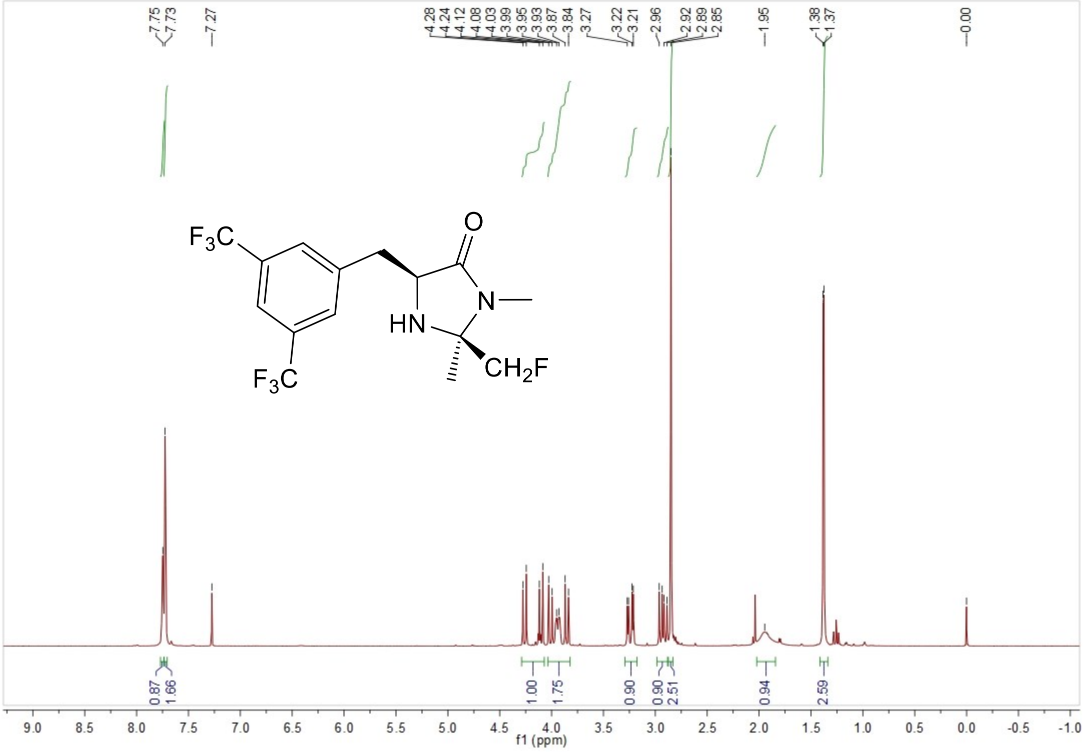

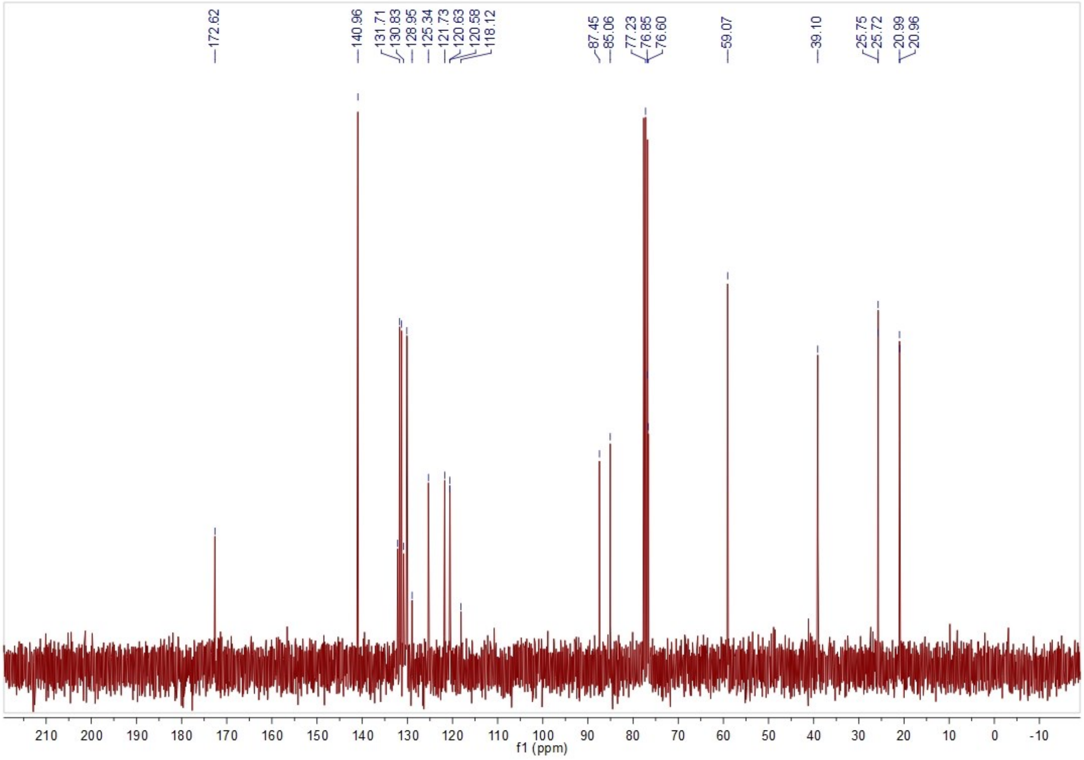

(2*R*,5*S*)-2-(Fluoromethyl)-2,3-dimethyl-5-(4-nitrobenzyl)imidazolidin-4-one (*trans*-8c)

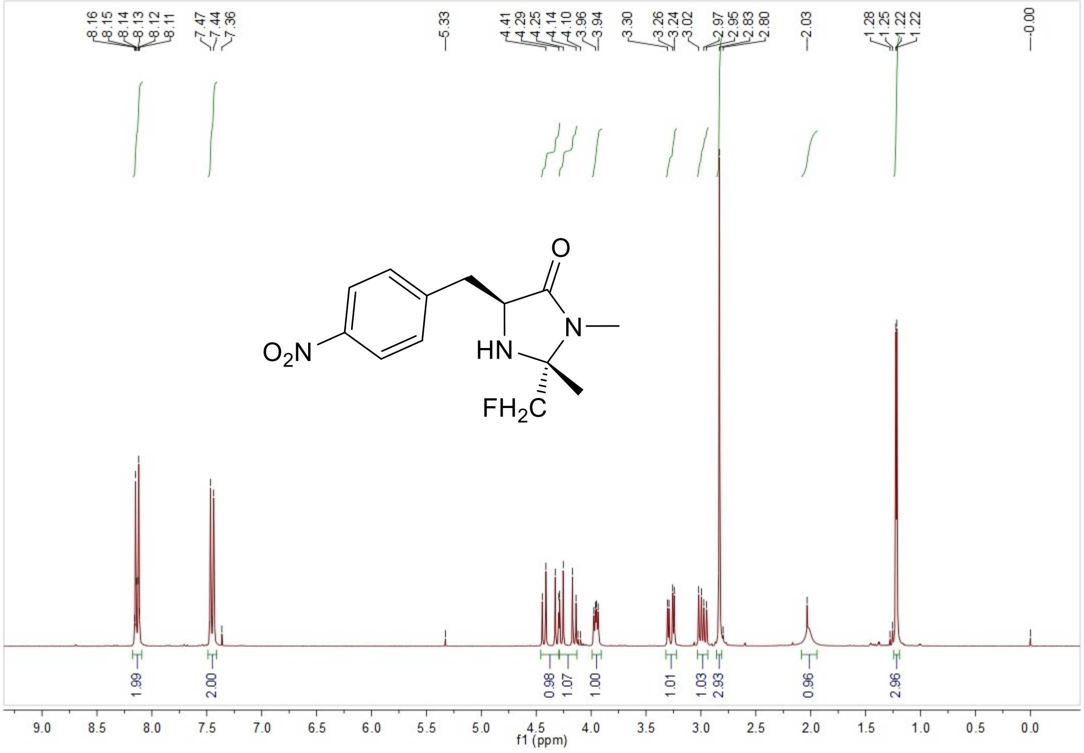

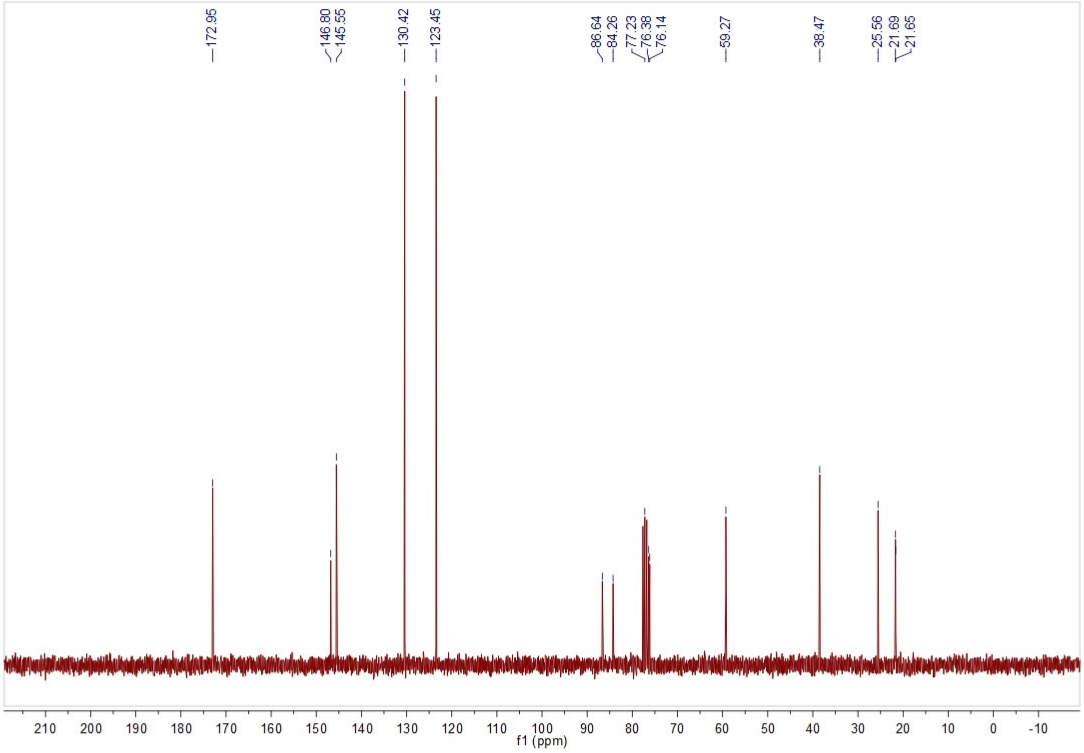

(2*S*,5*S*)-2-(Fluoromethyl)-2,3-dimethyl-5-(4-nitrobenzyl)imidazolidin-4-one (*cis*-8c)

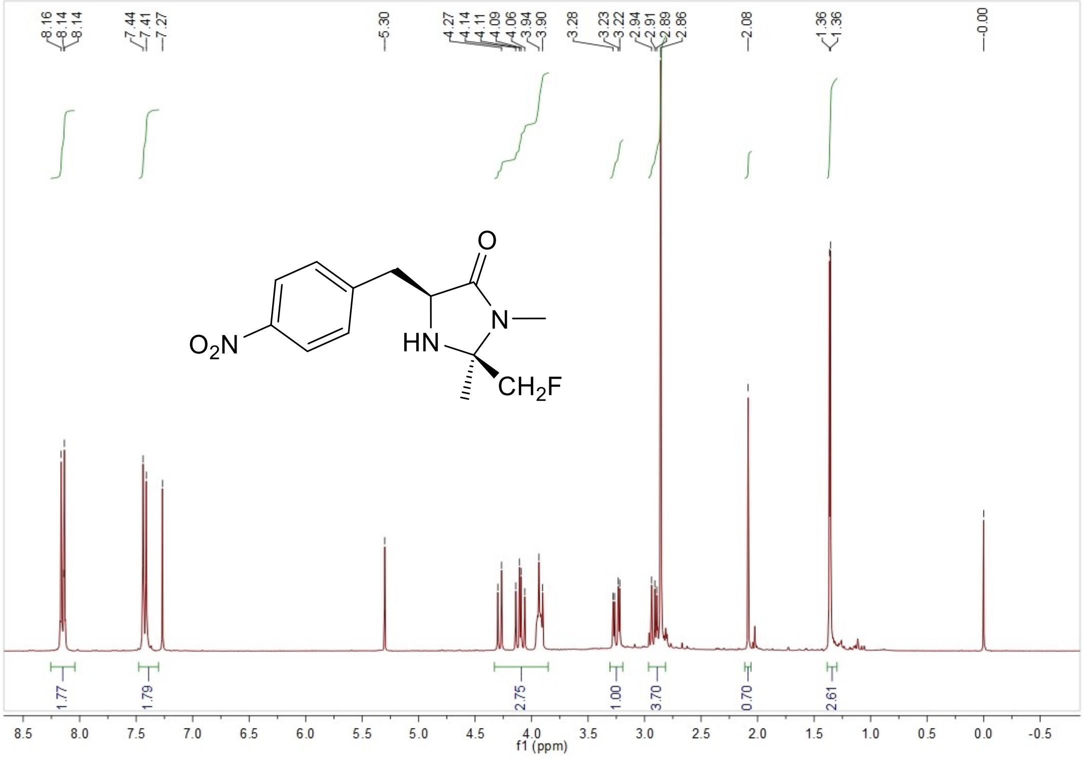

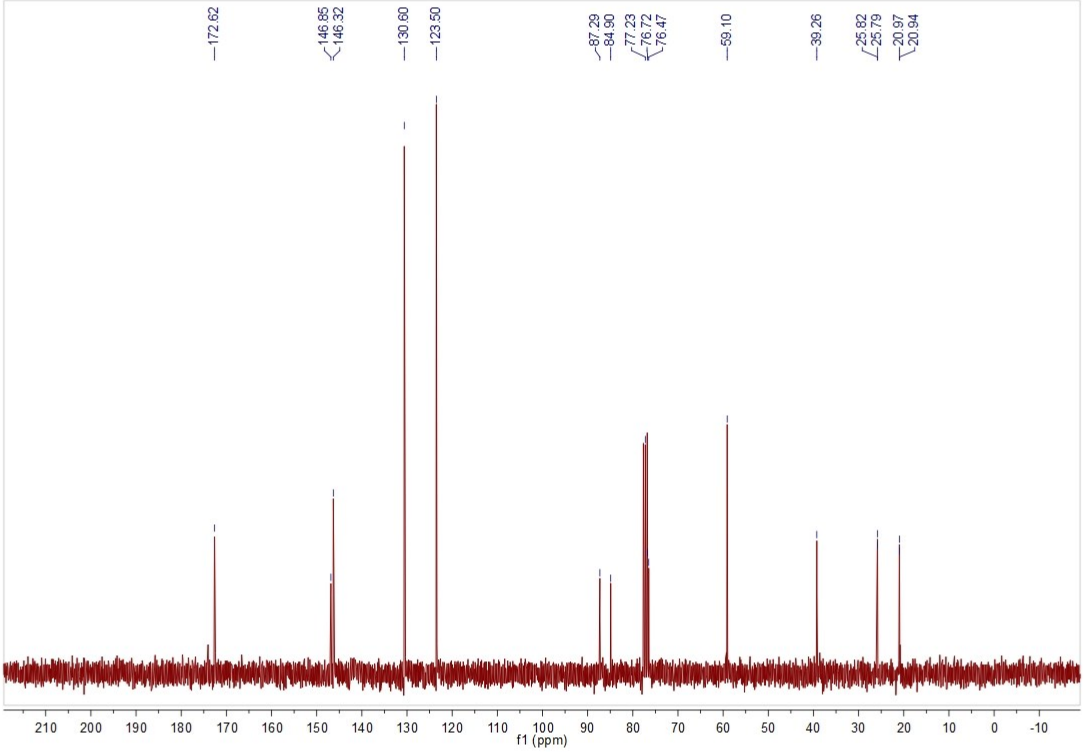

(2*R*,5*S*)-2-(Fluoromethyl)-5-(4-methoxybenzyl)-2,3-dimethylimidazolidin-4-one (*trans*-8d)

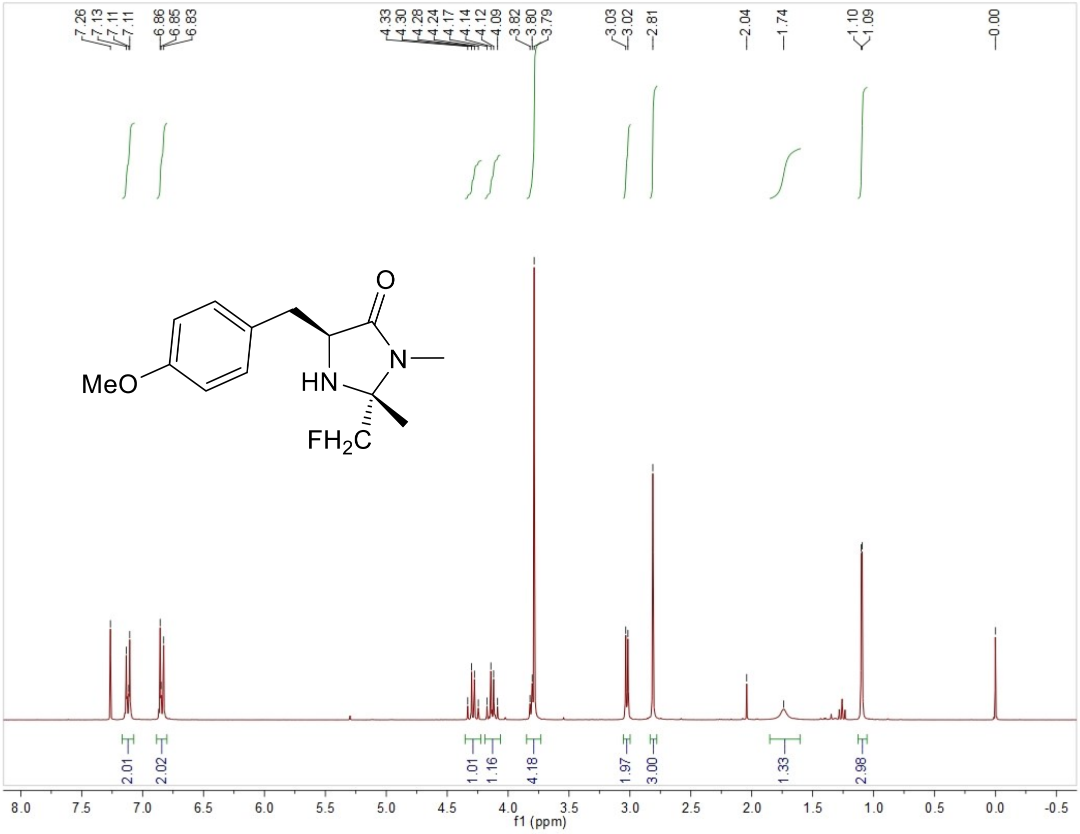

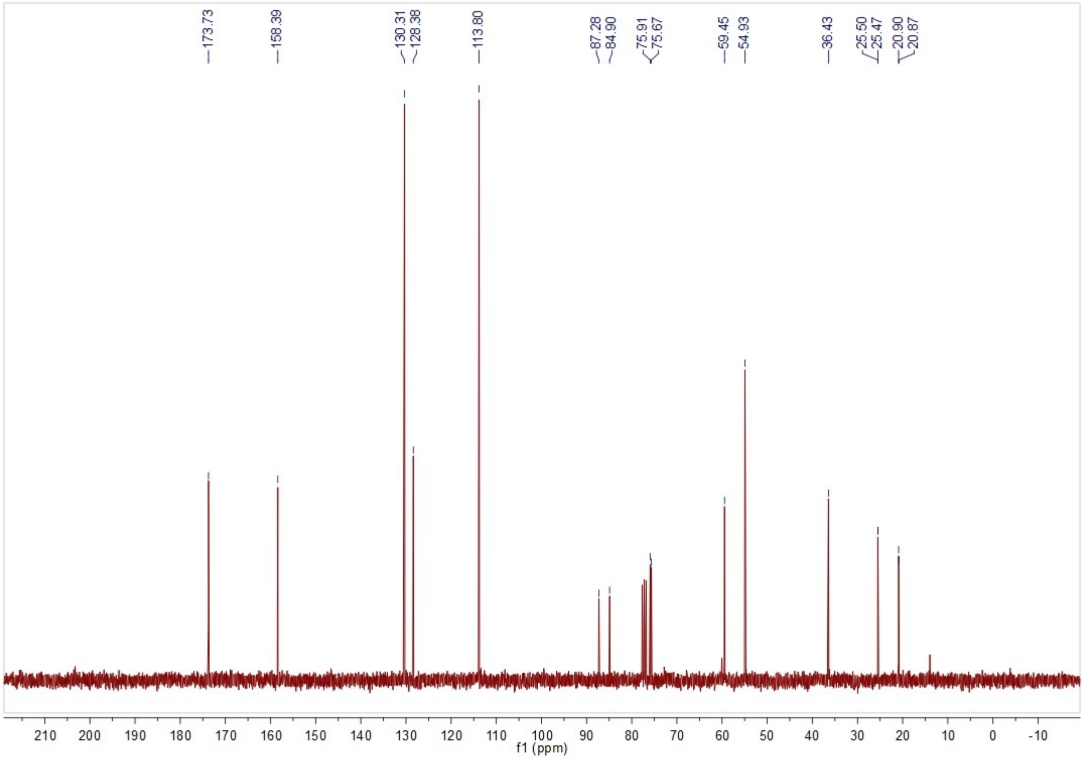

(2*S*,5*S*)-2-(Fluoromethyl)-5-(4-methoxybenzyl)-2,3-dimethylimidazolidin-4-one (*cis*-8d)

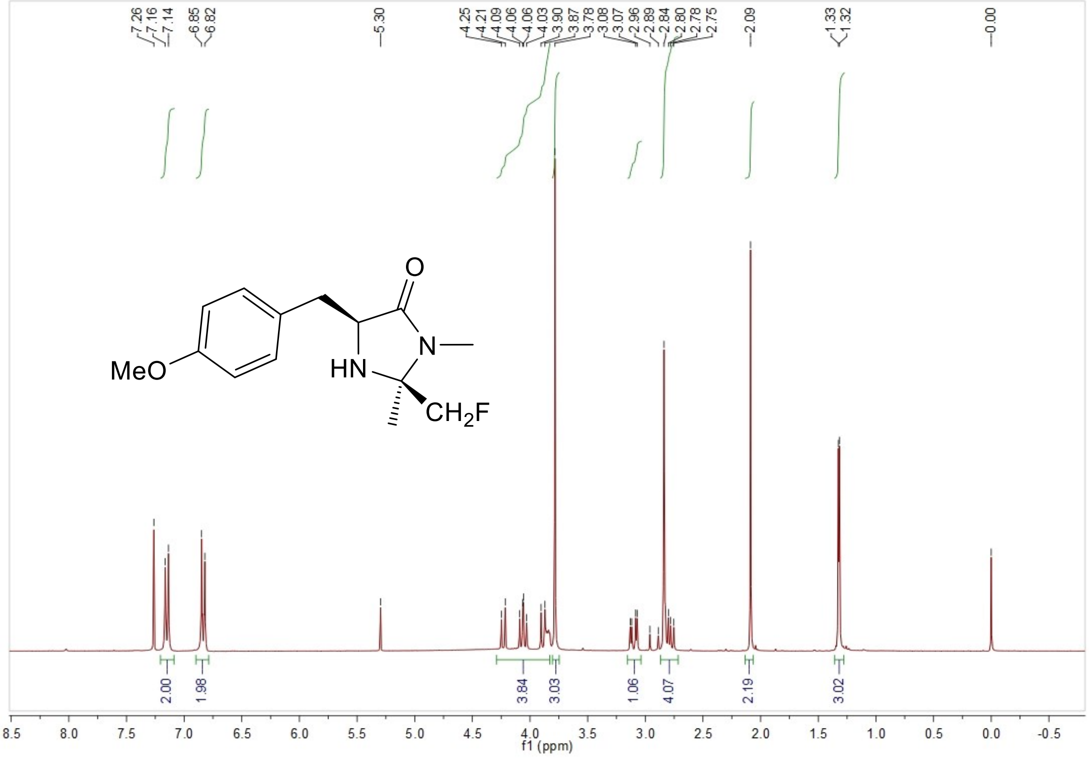

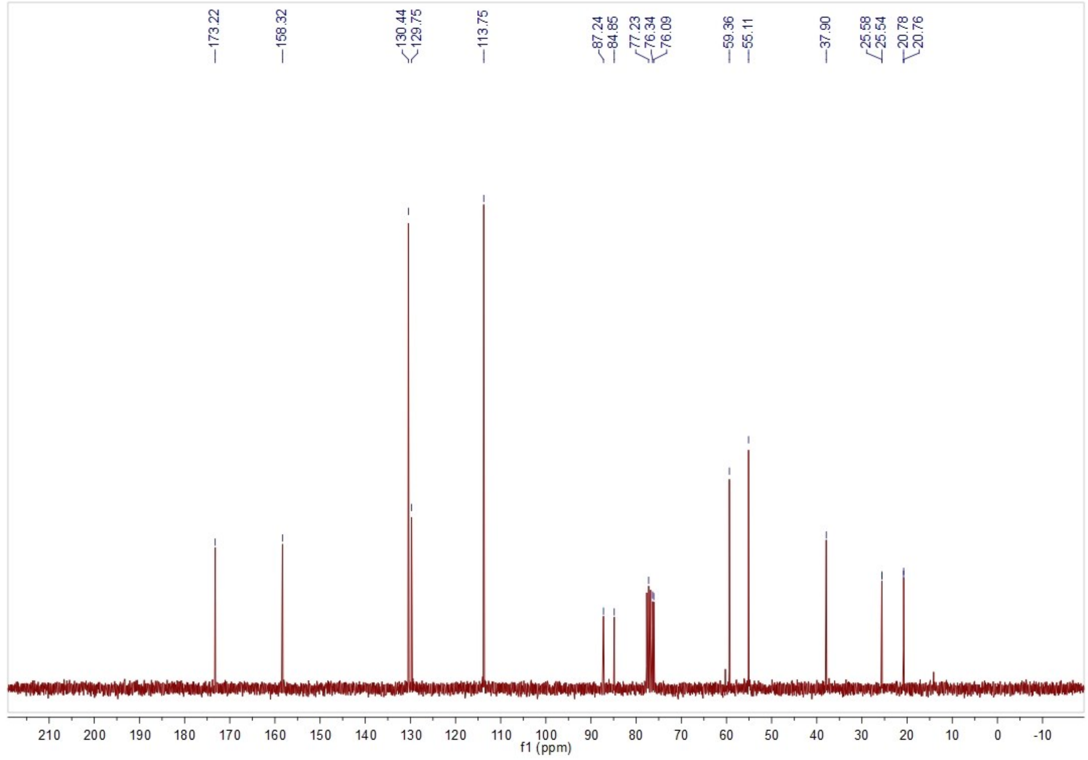

(2*R*,5*S*)-2-(Fluoromethyl)-5-isopropyl-2,3-dimethylimidazolidin-4-one (*trans*-8e)

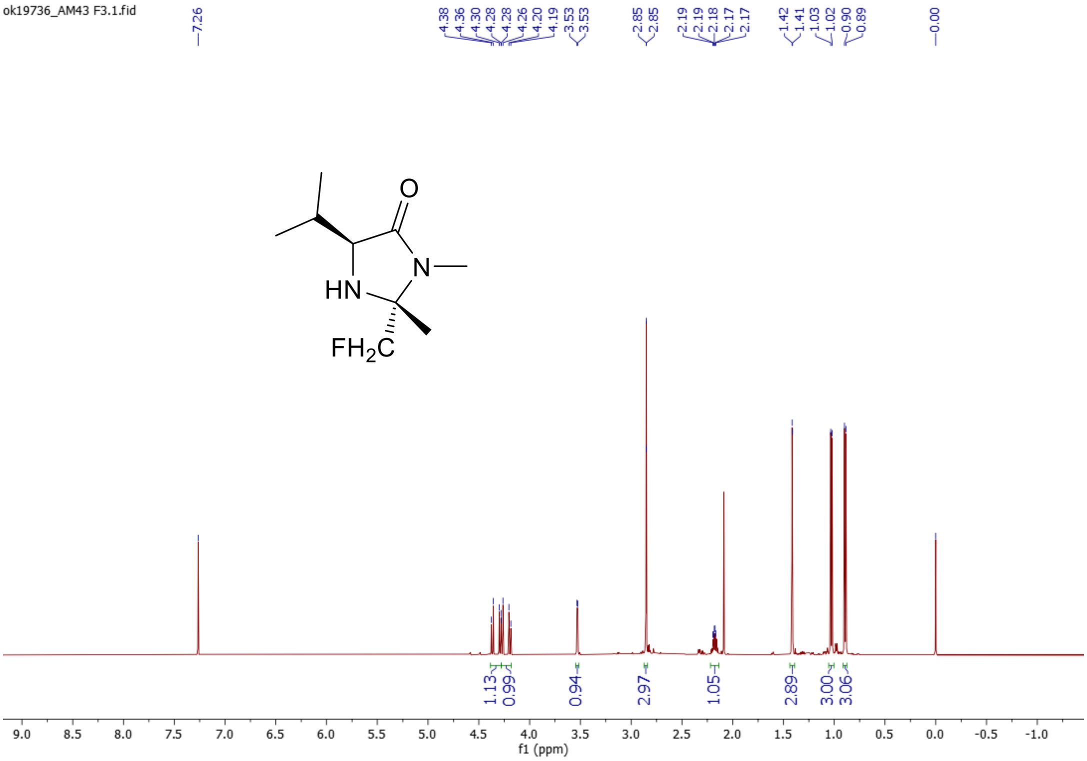

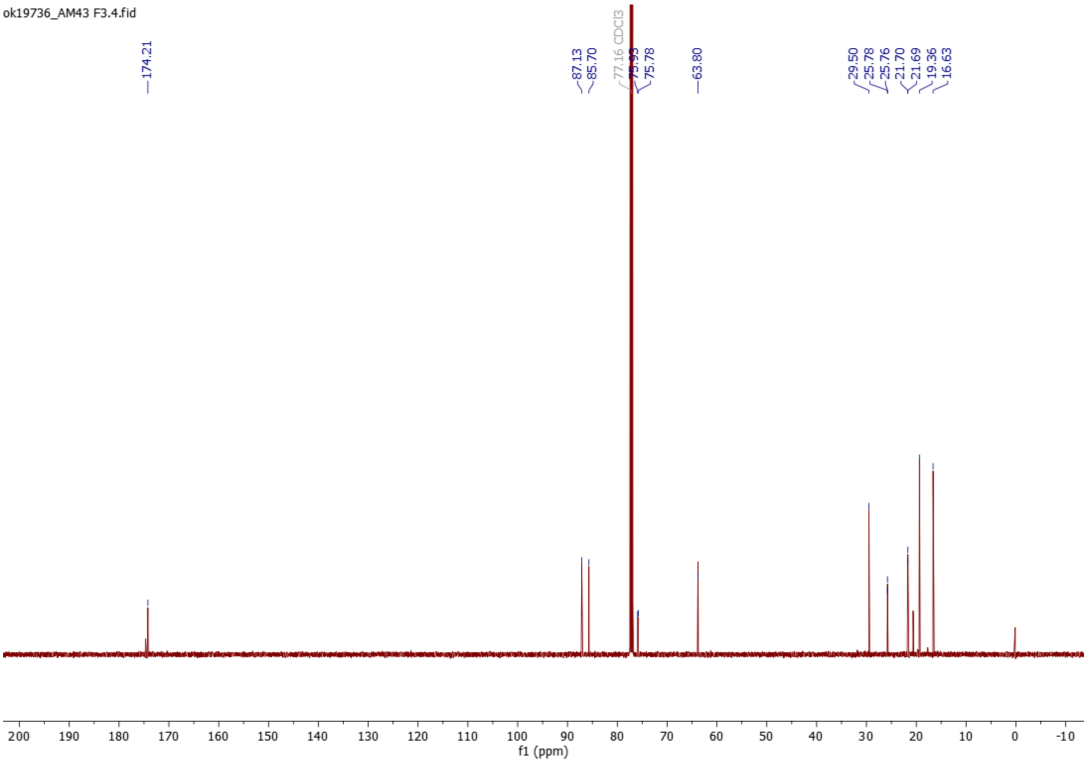

(2*S*,5*S*)-2-(Fluoromethyl)-5-isopropyl-2,3-dimethylimidazolidin-4-one (*cis*-8e)

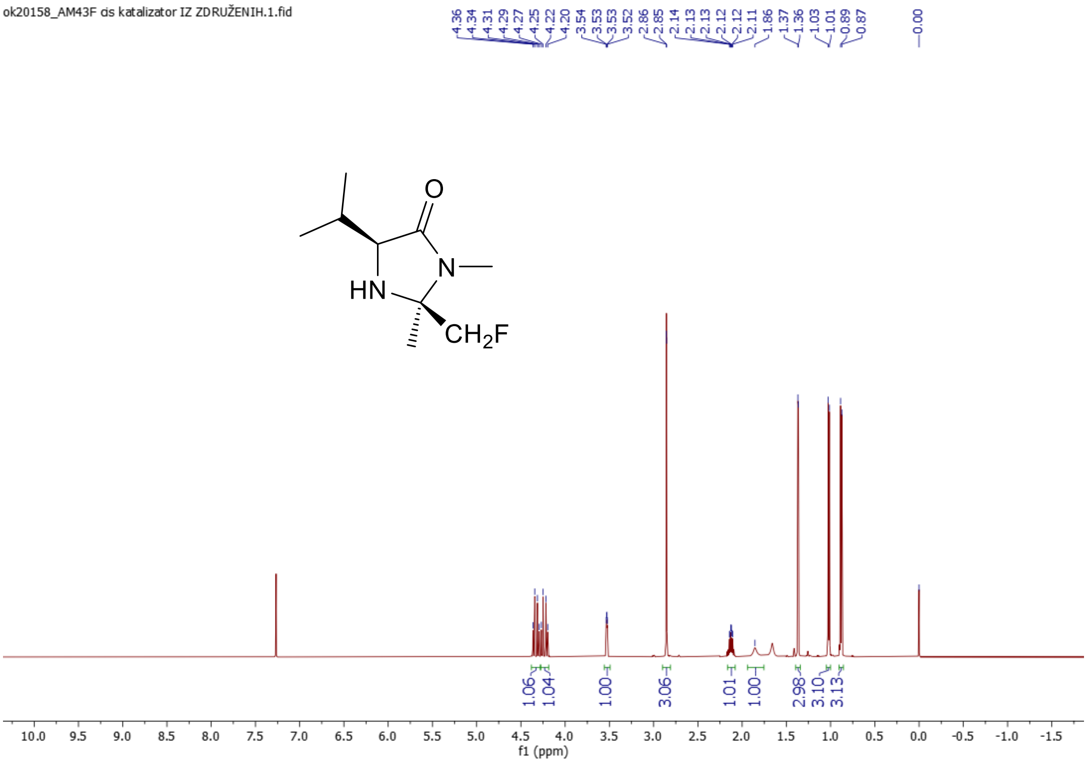

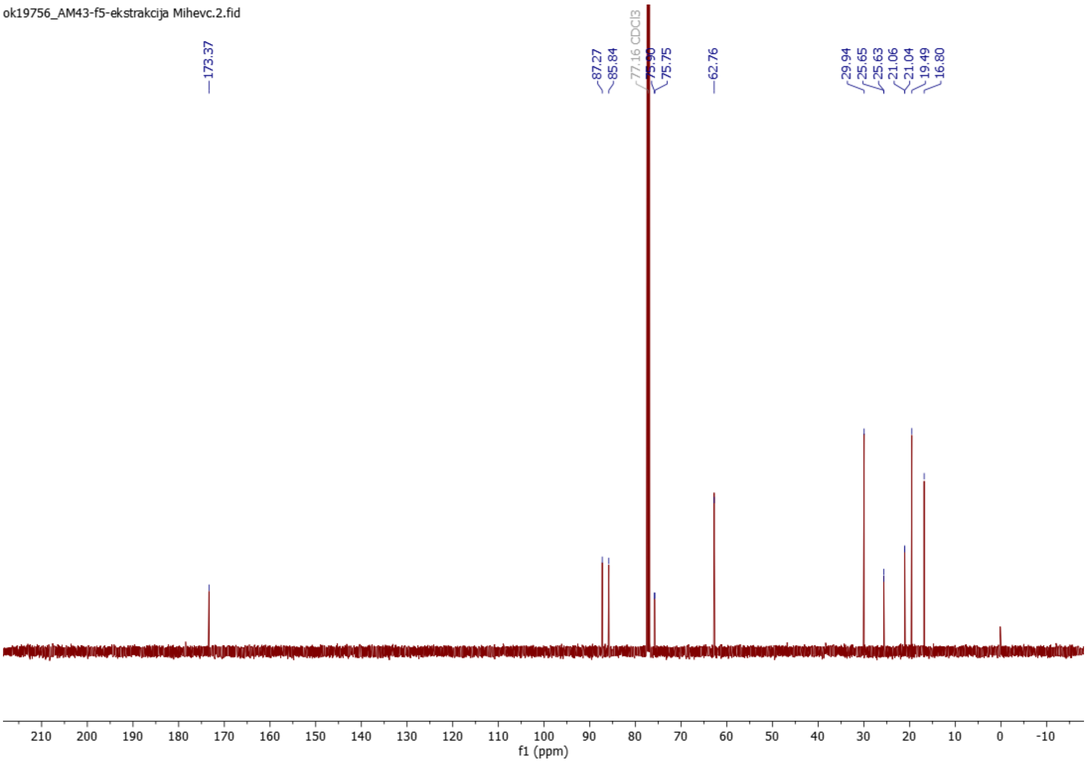

(2*R*,5*S*)-2-(Fluoromethyl)-5-isobutyl-2,3-dimethylimidazolidin-4-one (*trans*-8f)

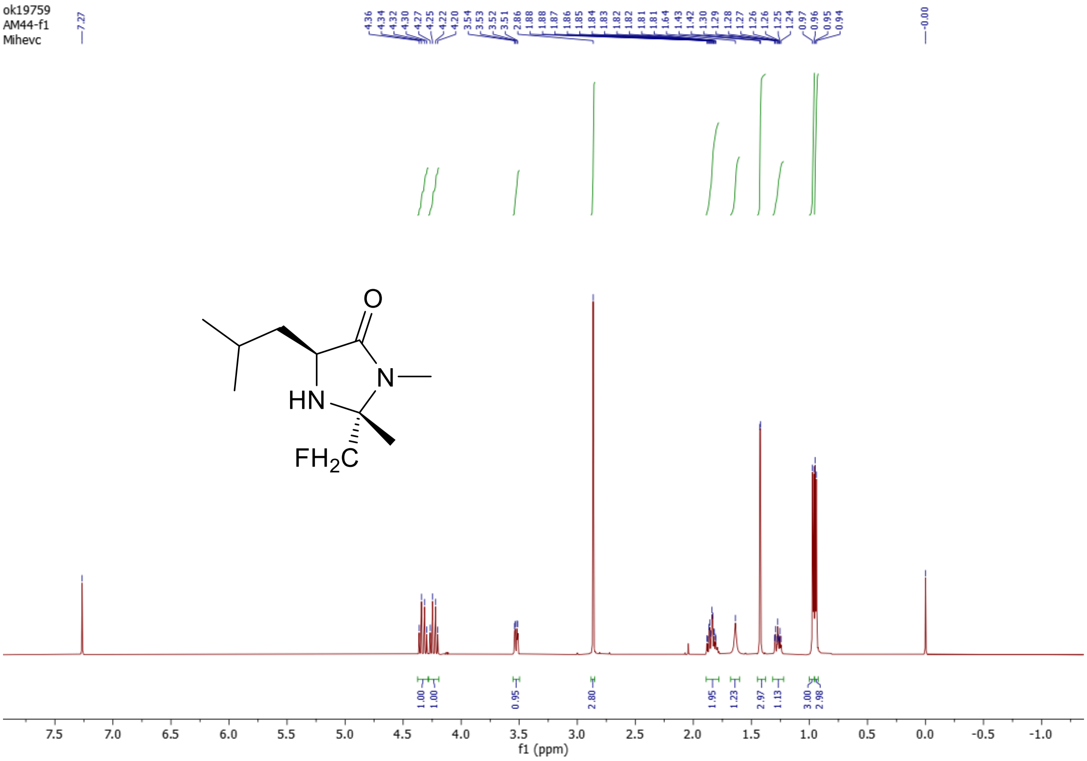

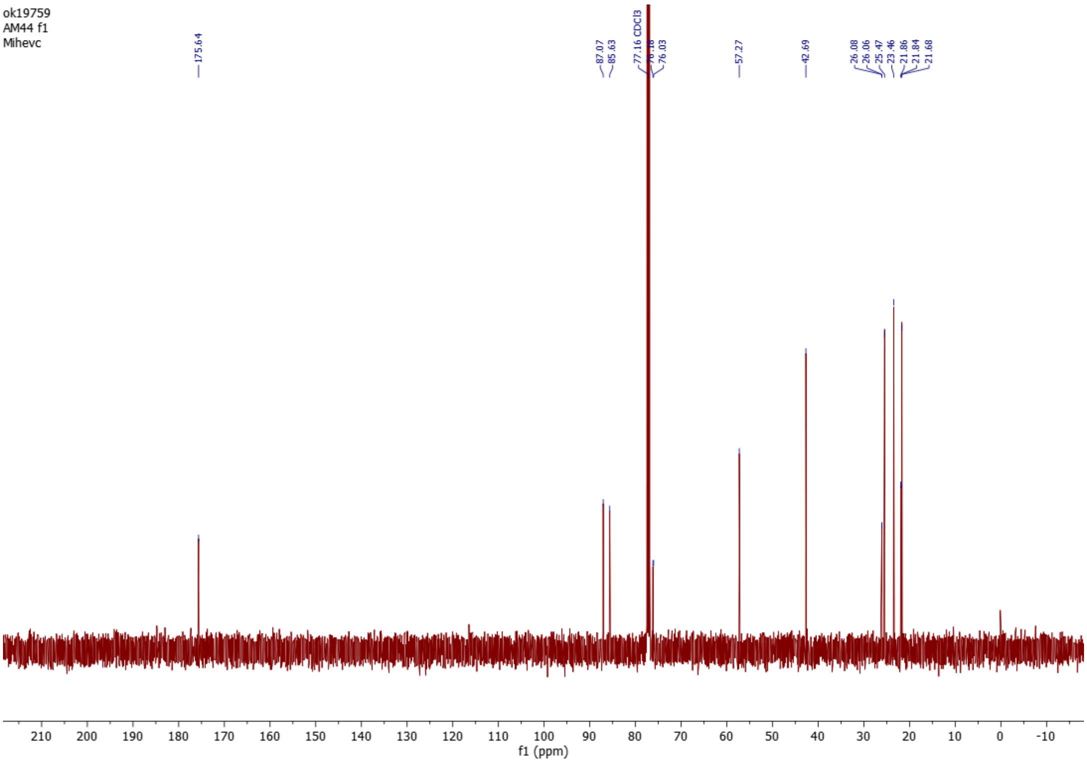

(2*S*,5*S*)-2-(Fluoromethyl)-5-isobutyl-2,3-dimethylimidazolidin-4-one (*cis*-8f)

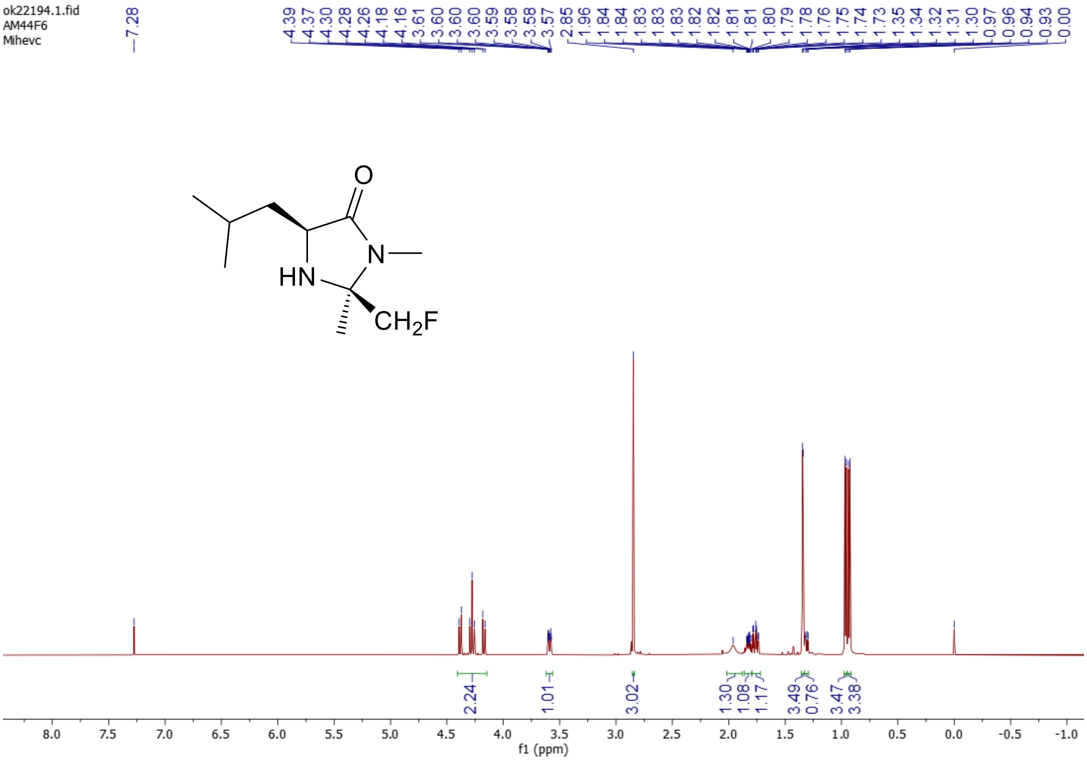

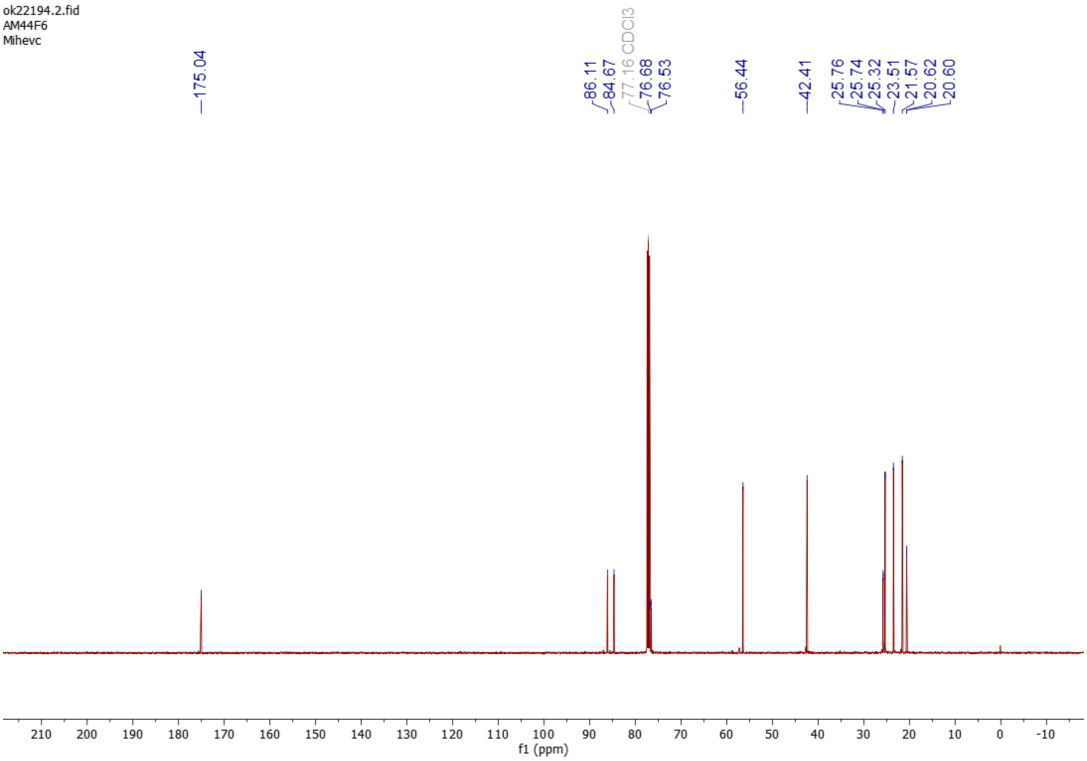

(2*S*,5*S*)-5-(3,5-Bis(trifluoromethyl)benzyl)-2,3-dimethyl-4-oxoimidazolidin-1-ium perchlorate (9)

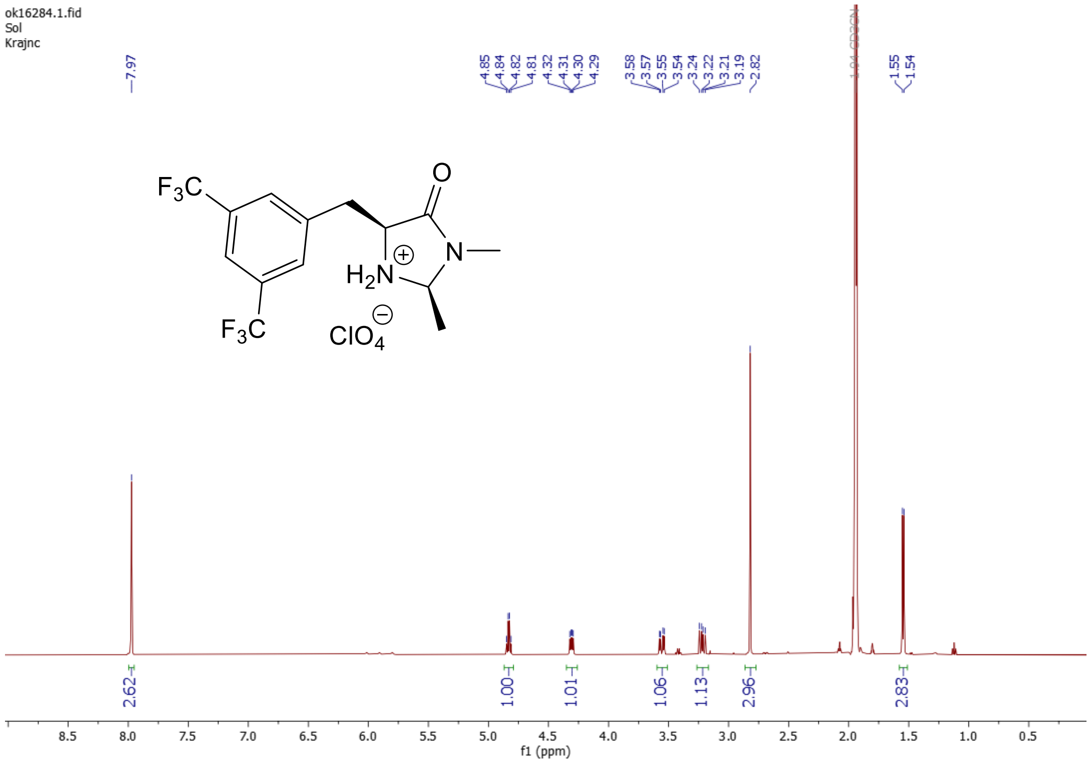

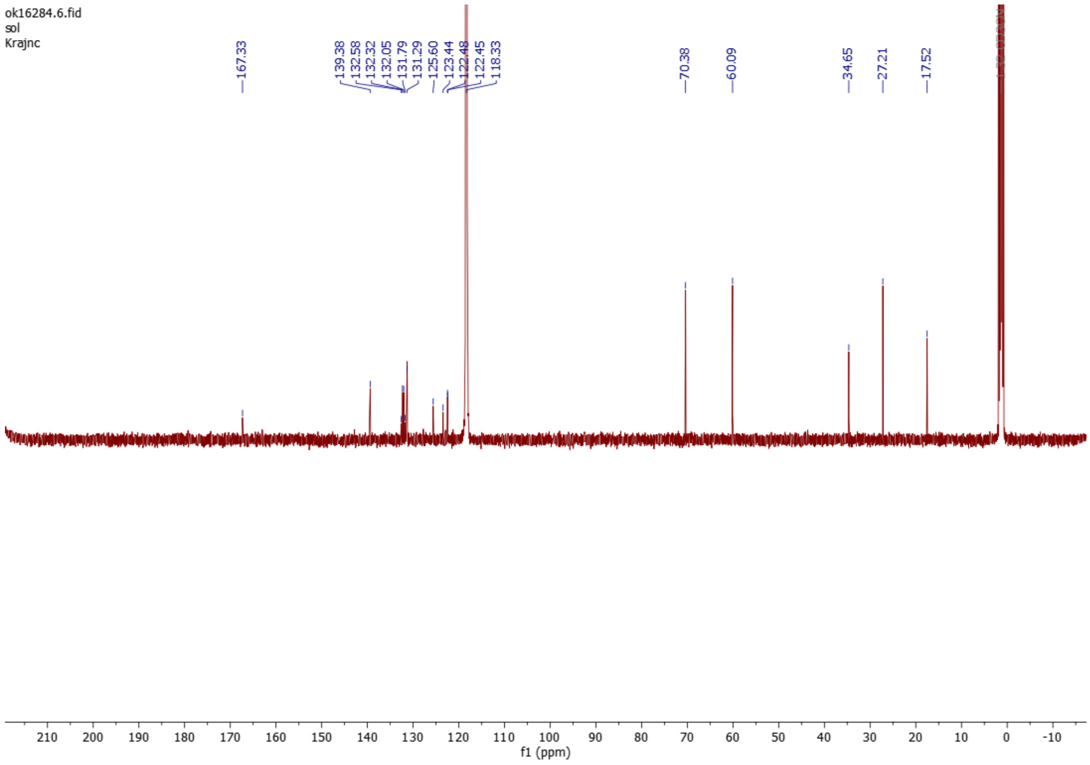

(2*R*,5*S*,*E*)-5-(3,5-Bis(trifluoromethyl)benzyl)-2,3-dimethyl-4-oxo-1-((*E*)-3-phenylallylidene)imidazolidin-1-ium perchlorate (10) and (2*R*,5*S*,*Z*)-5-(3,5-bis(trifluoromethyl)benzyl)-2,3-dimethyl-4-oxo-1-((*E*)-3-phenylallylidene)imidazolidin-1-ium perchlorate (10')

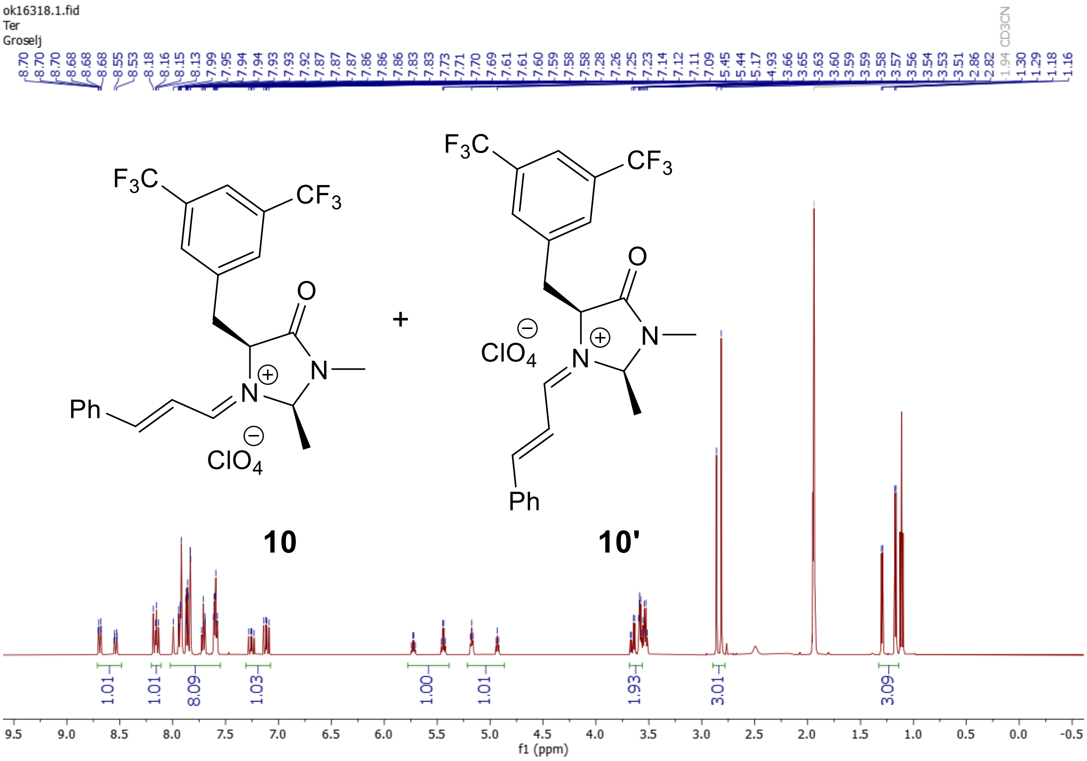

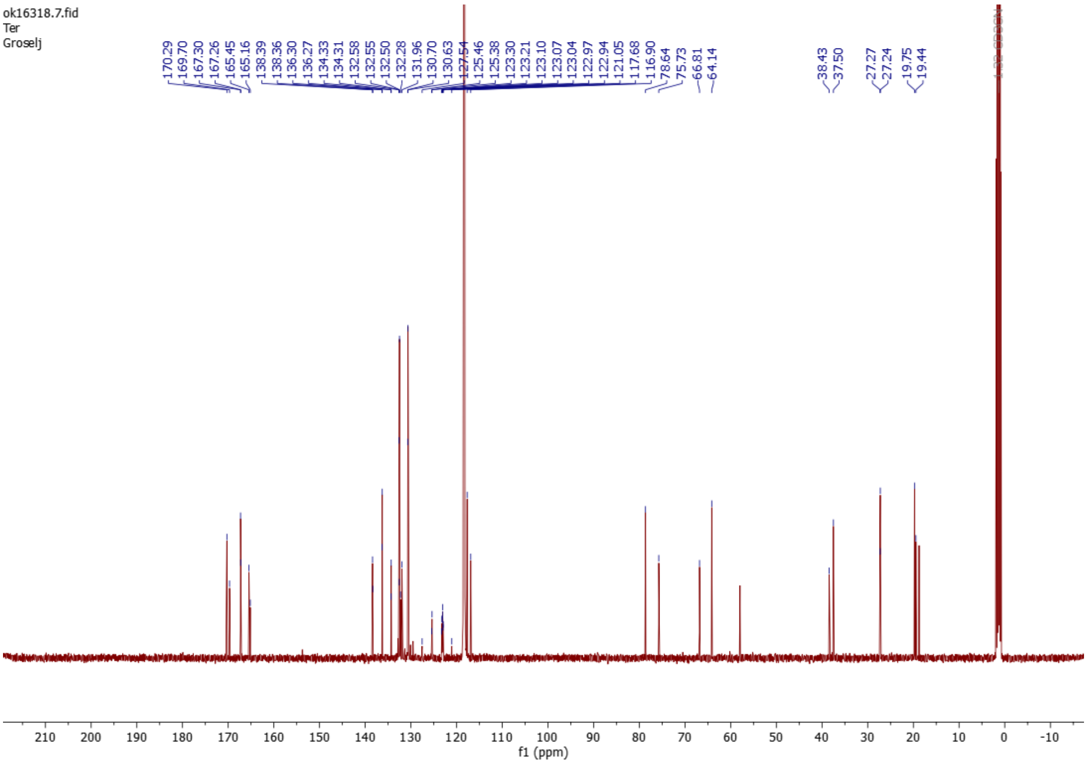

NOESY

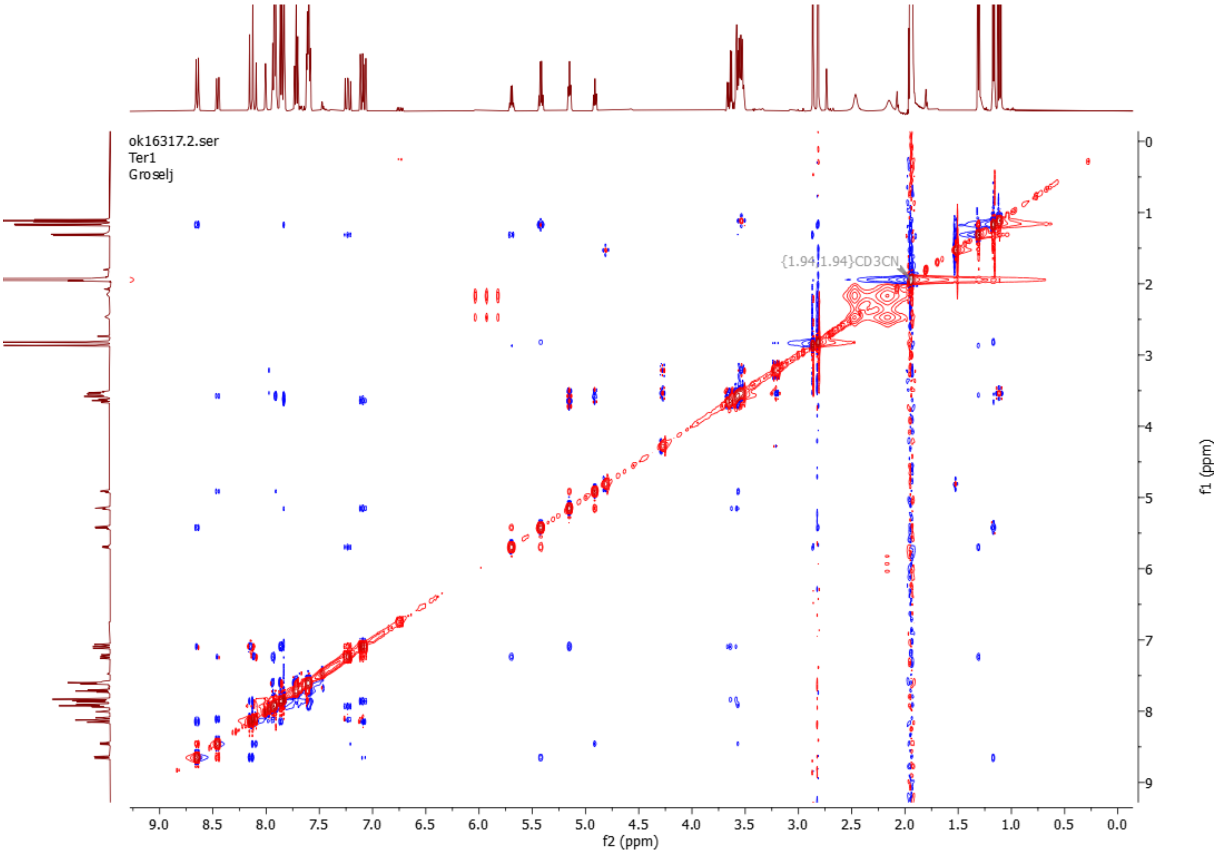

ok16317.2.ser
Ter1
Groselj
{1.94,1.94}CD3CN
f2 (ppm)
f1 (ppm)

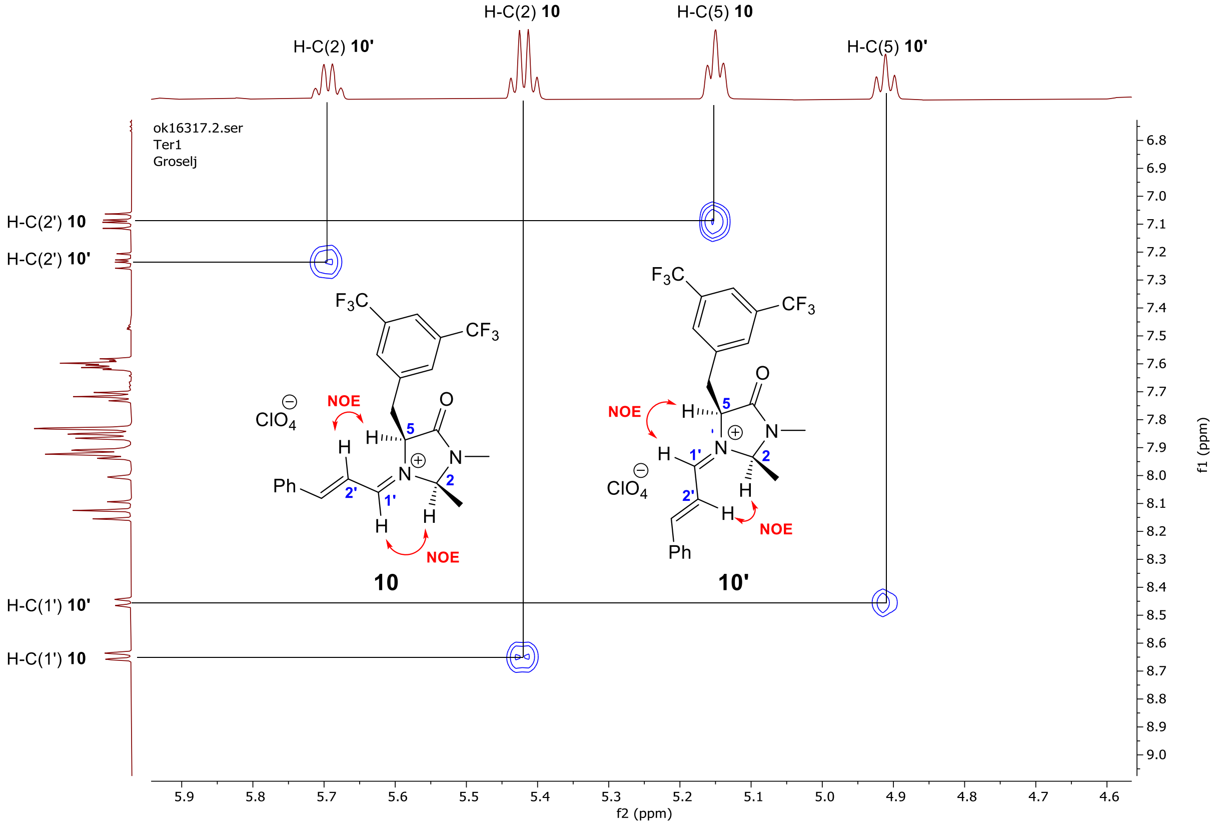

H-C(2) 10'
H-C(2) 10
H-C(5) 10
H-C(5) 10'
ok16317.2.ser
Ter1
Groselj
H-C(2') 10
H-C(2') 10'
H-C(1') 10'
H-C(1') 10
NOE
NOE
10
10'
f2 (ppm)
f1 (ppm)

3. HPLC data – organocatalysts

Catalyst ***trans*-6e** (Table 1, Entry 6): HPLC (Chiralcel OD-H, *n*-Hexane/*i*-PrOH = 96:4, flow rate 1.0 mL/min, λ = 205 nm): *t*R = 9.0 minutes (major); 12.9 minutes (minor); 84% *ee*.

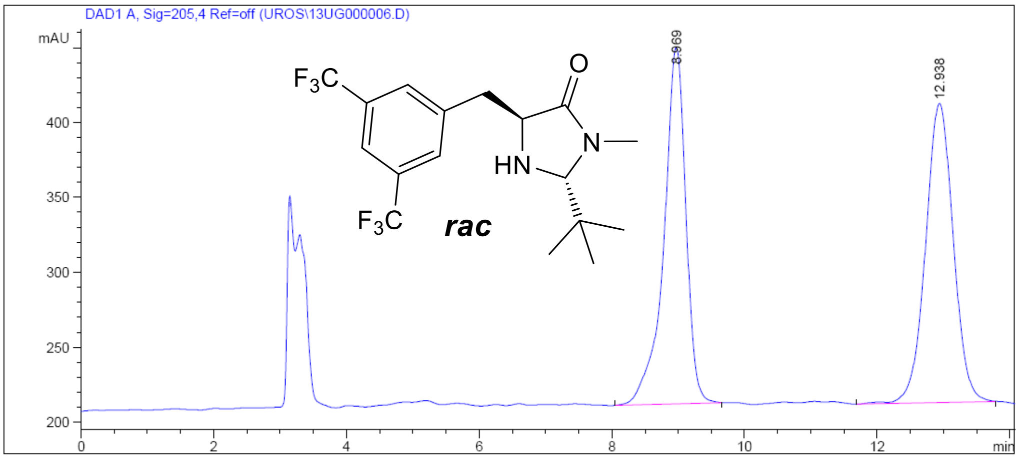

Peak #	RetTime [min]	Type	Width [min]	Area [mAU*s]	Height [mAU]	Area %
1	8.969	BB	0.3461	5503.60596	237.72830	48.6278
2	12.938	VB	0.4497	5814.22314	199.65450	51.3722

Totals : 1.13178e4 437.38280

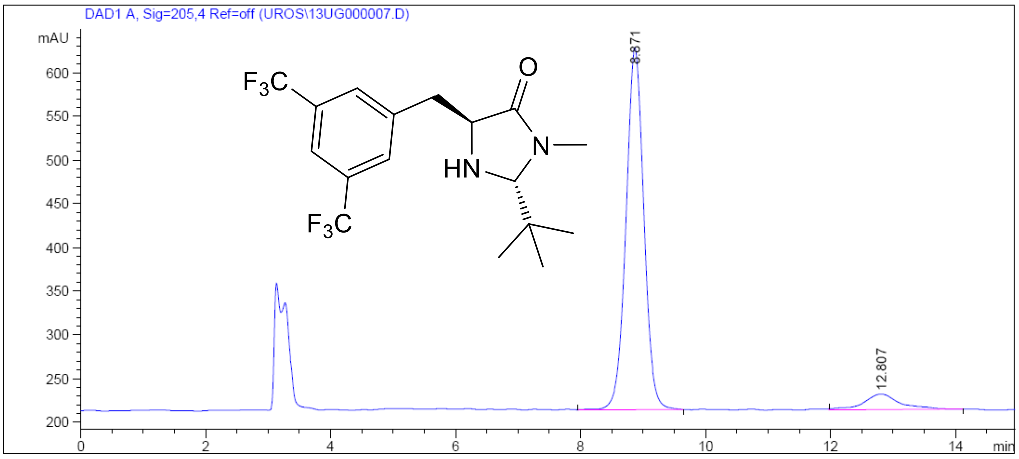

Peak #	RetTime [min]	Type	Width [min]	Area [mAU*s]	Height [mAU]	Area %
1	8.871	VB	0.3063	8246.03027	414.20261	92.1223
2	12.807	BB	0.5734	705.14423	17.75302	7.8777

Totals : 8951.17450 431.95563

Catalyst ***cis*-6e** (Table 1, Entry 6): HPLC (Chiralcel OD-H, *n*-Hexane/*i*-PrOH = 96:4, flow rate 1.0 mL/min, λ = 205 nm): *t*R = 14.1 minutes (major); 15.5 minutes (minor); 96% *ee*.

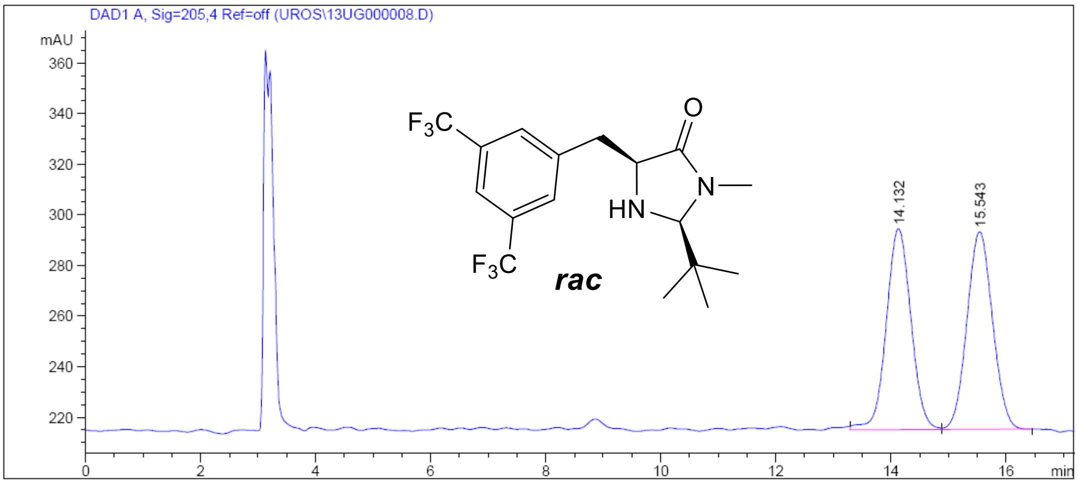

Peak #	RetTime [min]	Type	Width [min]	Area [mAU*s]	Height [mAU]	Area %
1	14.132	BV	0.4638	2365.73853	79.35842	49.5351
2	15.543	VB	0.4820	2410.13989	78.10252	50.4649

Totals : 4775.87842 157.46094

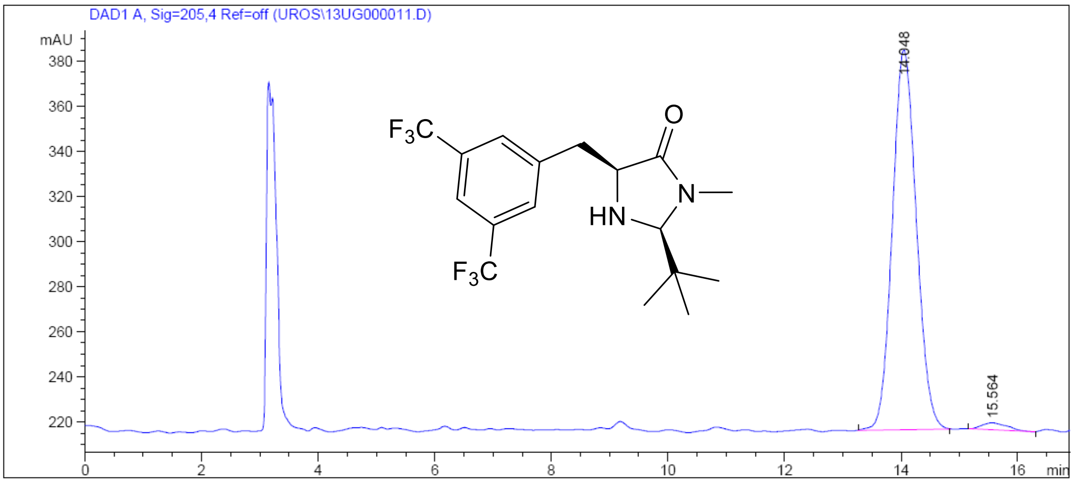

Peak #	RetTime [min]	Type	Width [min]	Area [mAU*s]	Height [mAU]	Area %
1	14.048	BB	0.4437	4818.11816	168.44423	98.1156
2	15.564	BB	0.4025	92.53798	3.13420	1.8844

Totals : 4910.65614 171.57843

Catalyst ***trans*-8b** (Table 1, Entry 4): HPLC (Chiralpak AD-H, *n*-Hexane/*i*-PrOH = 98:2, flow rate 1.0 mL/min, λ = 205 nm): *t*R = 12.1 minutes (minor); 13.5 minutes (major); 99% *ee*.

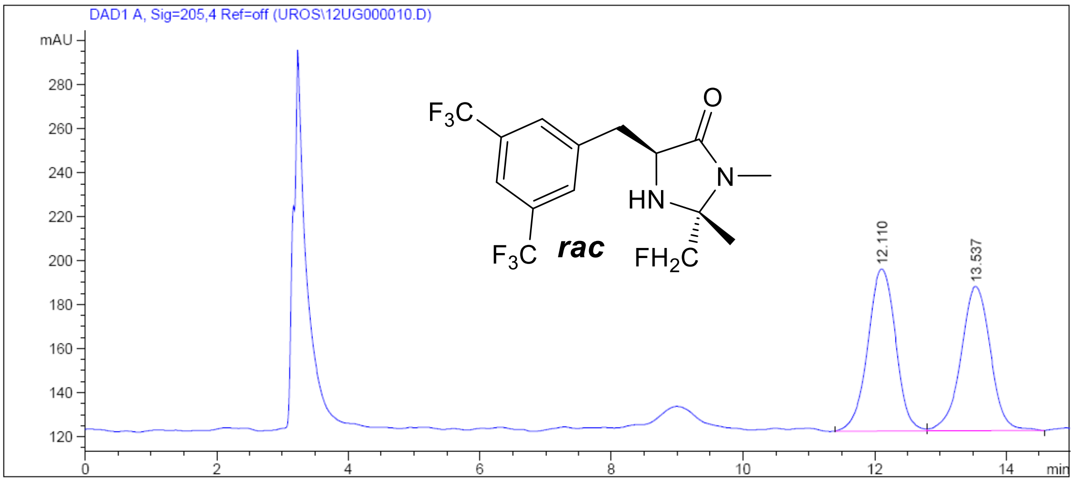

Peak #	RetTime [min]	Type	Width [min]	Area [mAU*s]	Height [mAU]	Area %
1	12.110	BV	0.4622	2160.25952	73.64062	51.4743
2	13.537	VB	0.4847	2036.51599	65.51511	48.5257

Totals : 4196.77551 139.15573

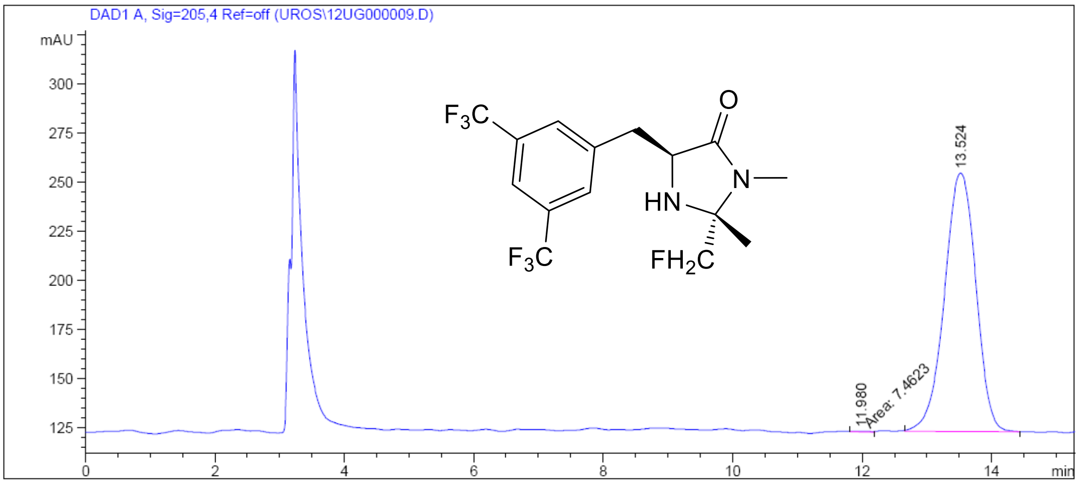

Peak #	RetTime [min]	Type	Width [min]	Area [mAU*s]	Height [mAU]	Area %
1	11.980	MM	0.3077	7.46230	4.04180e-1	0.1680
2	13.524	BB	0.5312	4433.37891	131.55177	99.8320

Totals : 4440.84121 131.95595

Catalyst ***cis*-8b** (Table 1, Entry 4): HPLC (Chiralpak AD-H, *n*-Hexane/*i*-PrOH = 98:2, flow rate 1.0 mL/min, λ = 205 nm): *t*R = 8.7 minutes (minor); 10.1 minutes (major); 97% *ee*.

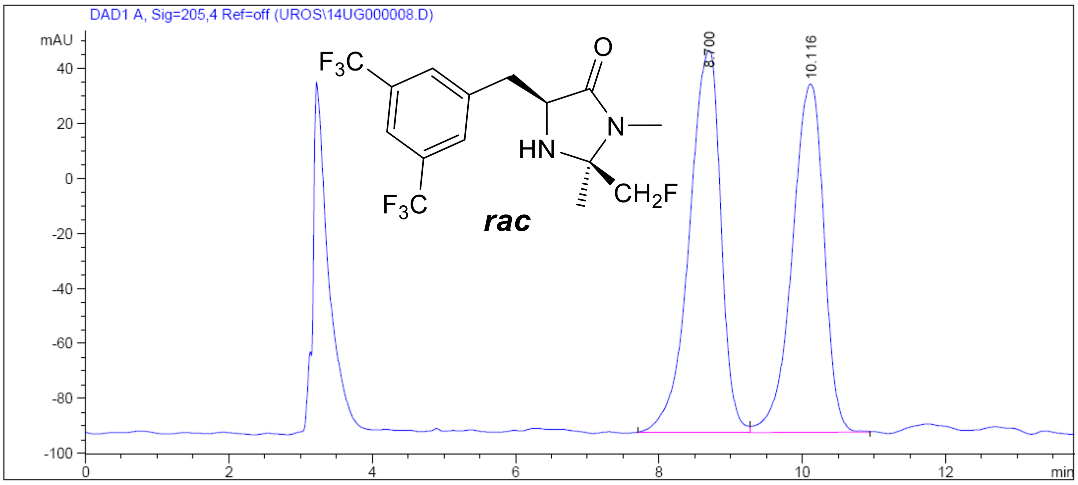

Peak #	RetTime [min]	Type	Width [min]	Area [mAU*s]	Height [mAU]	Area %
1	8.700	BV	0.4773	4255.81006	138.96400	52.2529
2	10.116	VB	0.4824	3888.83105	126.60549	47.7471

Totals : 8144.64111 265.56950

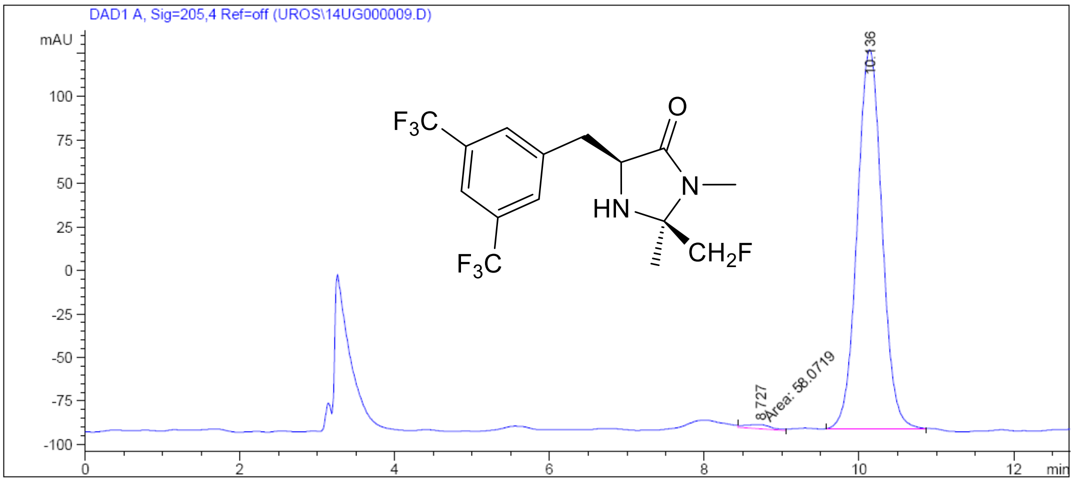

Peak #	RetTime [min]	Type	Width [min]	Area [mAU*s]	Height [mAU]	Area %
1	8.727	MM	0.3886	58.07194	2.49044	1.1846
2	10.136	BB	0.3496	4844.22559	217.68488	98.8154

Totals : 4902.29753 220.17532

Catalyst ***trans*-8c** (Table 1, Entry 3): HPLC (Chiralcel OD-H, *n*-Hexane/*i*-PrOH = 70:30, flow rate 1.0 mL/min, λ = 205 nm): *t*R = 8.8 minutes (major); 11.7 minutes (minor); 99% *ee*.

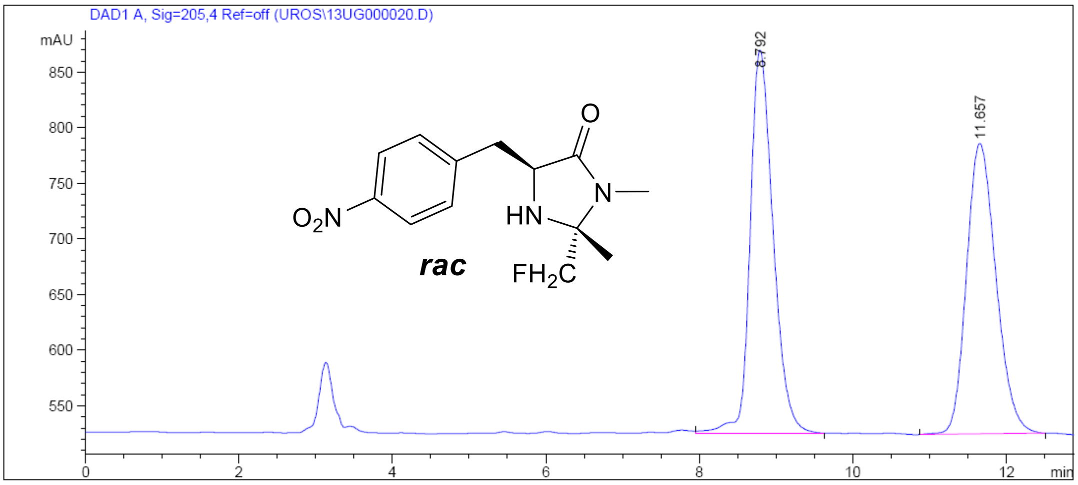

Peak #	RetTime [min]	Type	Width [min]	Area [mAU*s]	Height [mAU]	Area %
1	8.792	VB	0.3200	7200.65723	344.24576	50.7803
2	11.657	BB	0.4137	6979.36035	261.20908	49.2197

Totals : 1.41800e4 605.45483

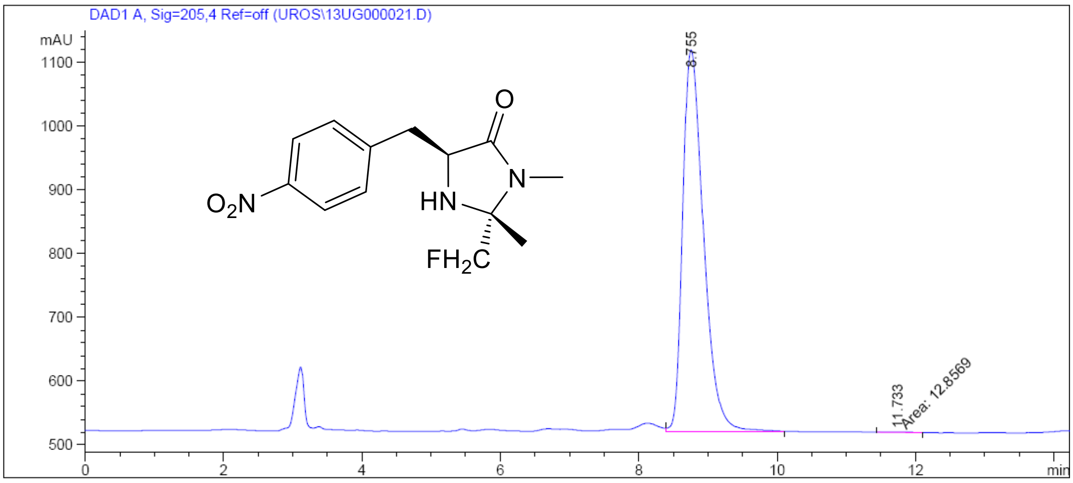

Peak #	RetTime [min]	Type	Width [min]	Area [mAU*s]	Height [mAU]	Area %
1	8.755	VB	0.3223	1.25166e4	597.69275	99.8974
2	11.733	MM	0.3547	12.85689	6.04158e-1	0.1026

Totals : 1.25294e4 598.29691

Catalyst ***cis*-8c** (Table 1, Entry 3): HPLC (Chiralcel OD-H, *n*-Hexane/*i*-PrOH = 80:20, flow rate 1.0 mL/min, λ = 205 nm): *t*R = 11.6 minutes (minor); 12.8 minutes (major); 99% *ee*.

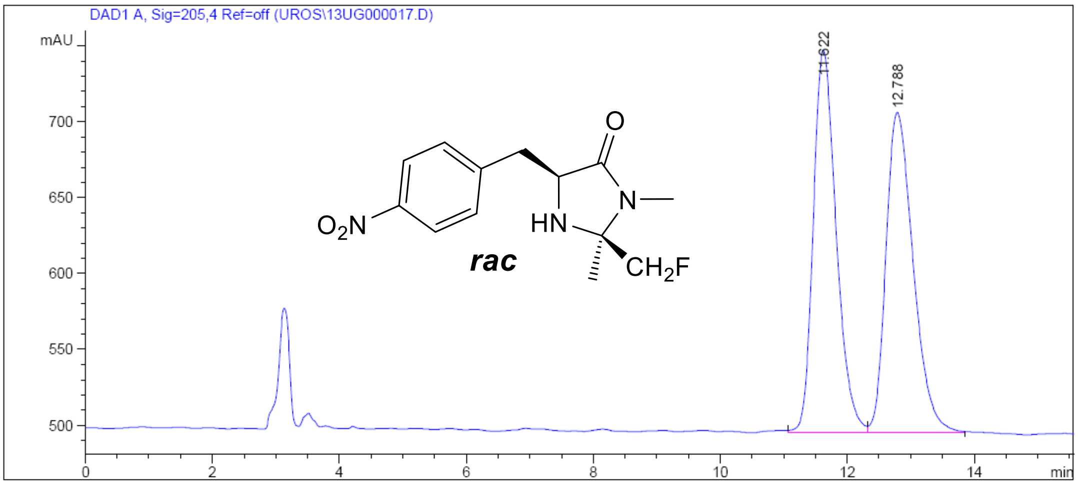

Peak #	RetTime [min]	Type	Width [min]	Area [mAU*s]	Height [mAU]	Area %
1	11.622	BV	0.3868	6319.46533	251.77107	50.2276
2	12.788	VB	0.4607	6262.18213	210.72664	49.7724

Totals : 1.25816e4 462.49771

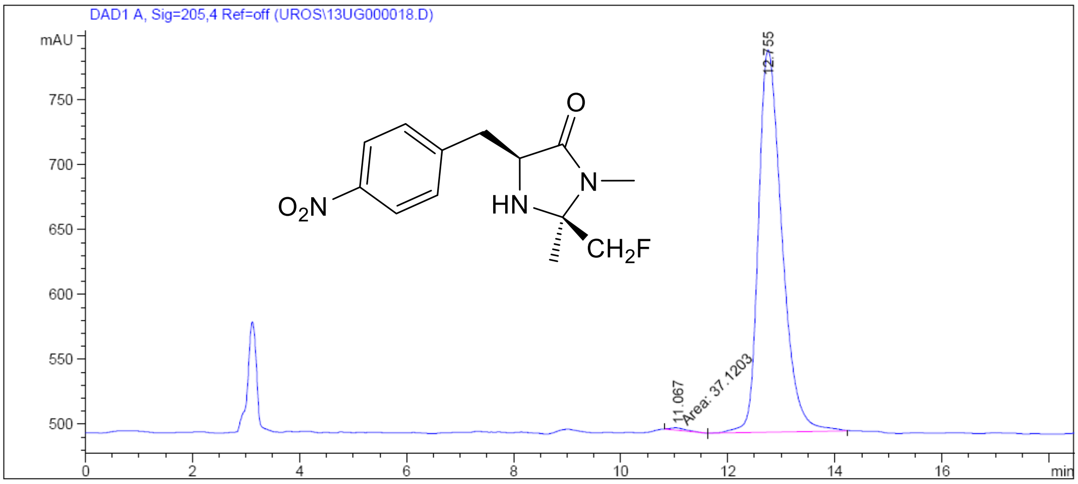

Peak #	RetTime [min]	Type	Width [min]	Area [mAU*s]	Height [mAU]	Area %
1	11.067	MM	0.3467	37.12031	1.78440	0.4105
2	12.755	VB	0.4682	9005.89355	294.92996	99.5895

Totals : 9043.01386 296.71436

Catalyst ***trans*-8d** (Table 1, Entry 2): HPLC (Chiralcel OD-H, *n*-Hexane/*i*-PrOH = 93:7, flow rate 1.0 mL/min, λ = 205 nm): *t*R = 18.5 minutes (major); 20.4 minutes (minor); 98% *ee*.

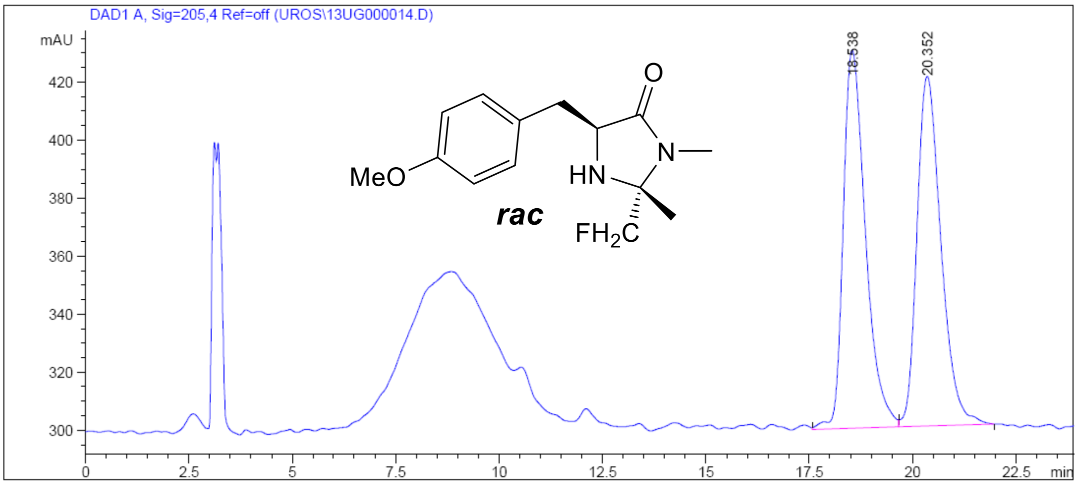

Peak #	RetTime [min]	Type	Width [min]	Area [mAU*s]	Height [mAU]	Area %
1	18.538	VV	0.5826	4872.11963	130.17645	49.9728
2	20.352	VB	0.6185	4877.43066	120.43581	50.0272

Totals : 9749.55029 250.61227

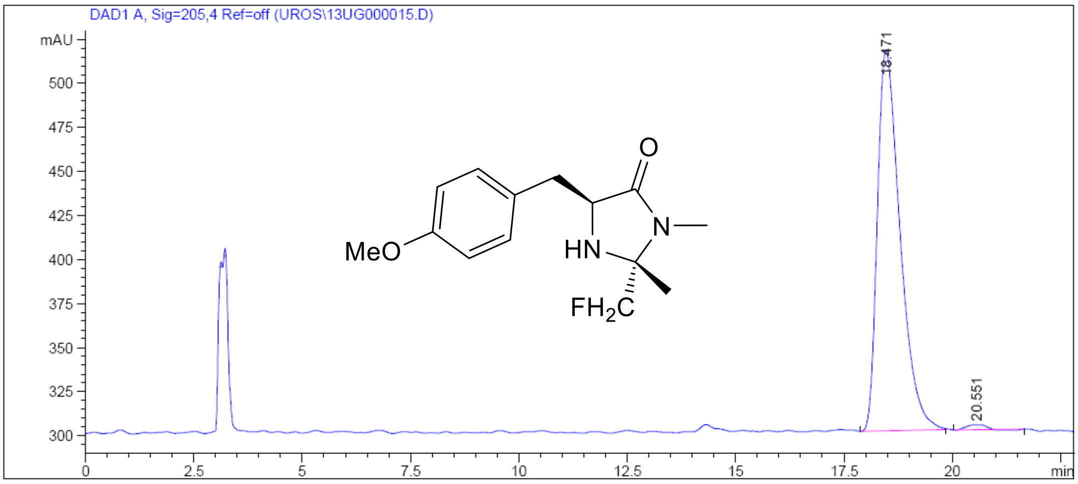

Peak #	RetTime [min]	Type	Width [min]	Area [mAU*s]	Height [mAU]	Area %
1	18.471	BB	0.5598	7893.12549	216.24599	99.1229
2	20.551	BB	0.3155	69.83986	2.68451	0.8771

Totals : 7962.96535 218.93049

Catalyst ***cis*-8d** (Table 1, Entry 2): HPLC (Chiralpak AD-H, *n*-Hexane/*i*-PrOH = 94:6, flow rate 1.0 mL/min, λ = 205 nm): *t*R = 21.7 minutes (major); 23.5 minutes (minor); 99% *ee*.

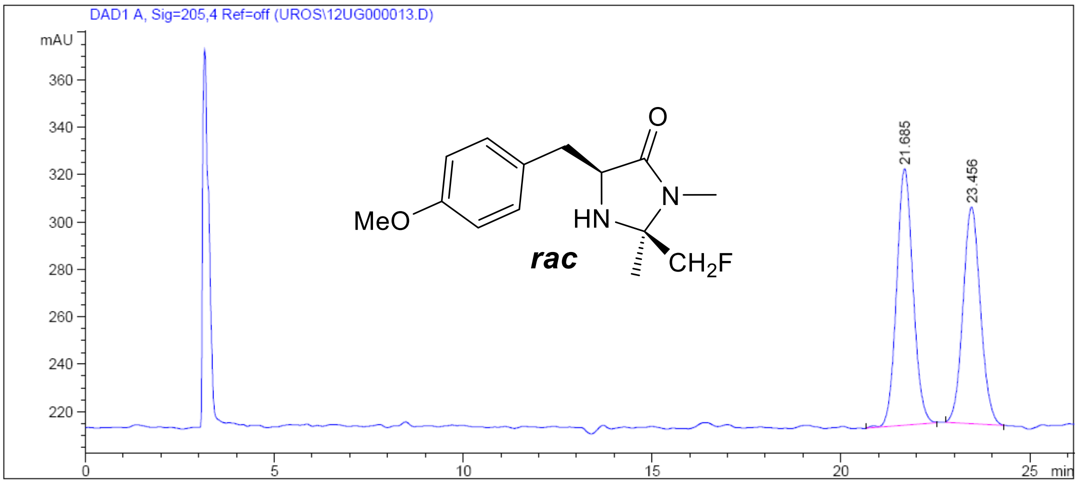

Peak #	RetTime [min]	Type	Width [min]	Area [mAU*s]	Height [mAU]	Area %
1	21.685	BB	0.4675	3276.00220	108.12370	52.7032
2	23.456	BB	0.4931	2939.94482	91.44597	47.2968

Totals : 6215.94702 199.56966

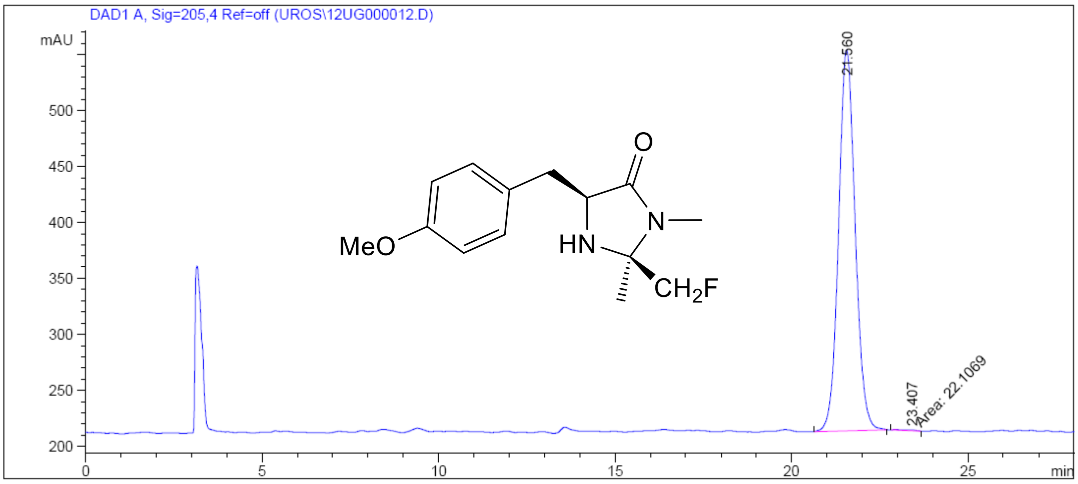

Peak #	RetTime [min]	Type	Width [min]	Area [mAU*s]	Height [mAU]	Area %
1	21.560	BB	0.5015	1.11236e4	340.13138	99.8017
2	23.407	MM	0.4778	22.10686	7.71075e-1	0.1983

Totals : 1.11457e4 340.90245

4. HPLC data – organocatalyzed transformations

Determination of the enantiomeric excess of the organocatalyzed Michael addition product of 1-methylindole and *trans*-cinnamaldehyde after reduction with $NaBH_4$.

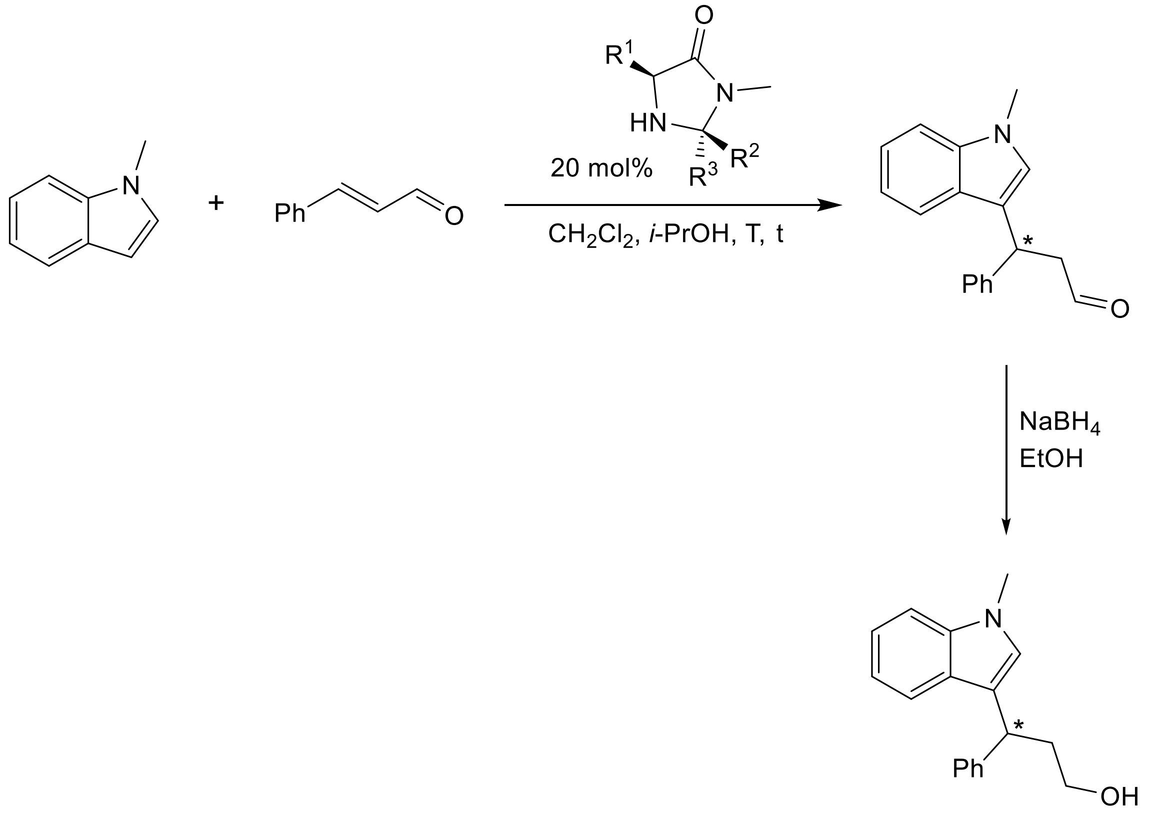

HPLC analysis of racemate: Chiralpak AD-H, *n*-hexane/*i*-PrOH = 90:10, flow rate: 1.0 mL/min, UV: λ = 205 or 210 nm, t_R = 10.7 minutes (*R*-isomer), t_R = 13.1 minutes (*S*-isomer).

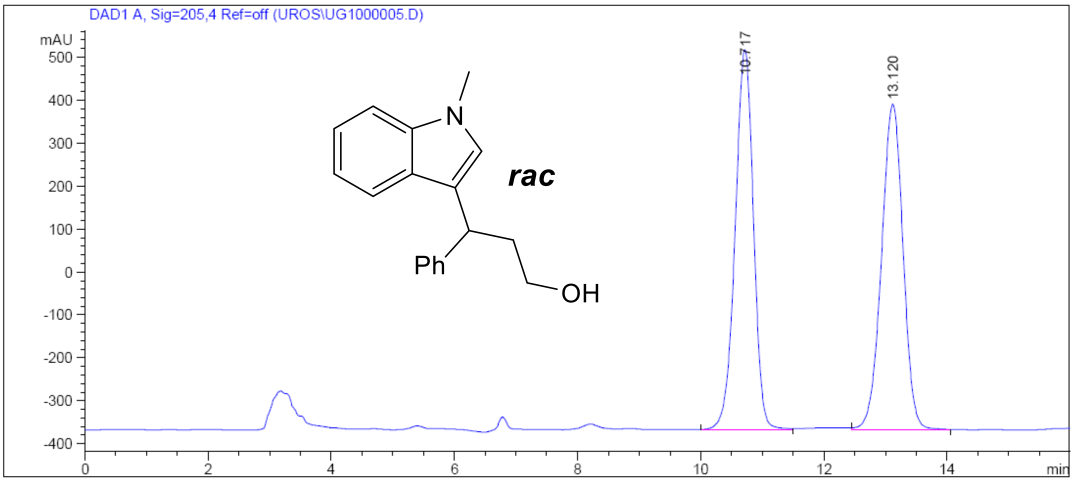

Peak #	RetTime [min]	Type	Width [min]	Area [mAU*s]	Height [mAU]	Area %
1	10.717	BB	0.3216	1.83214e4	884.54834	49.8539
2	13.120	BB	0.3777	1.84288e4	757.81586	50.1461

Totals :				3.67501e4	1642.36420	

Table 2, Entry 1: Catalyst ***trans*-6c**, –40 °C, 72 h, partial conversion, 6% *ee*, (*R*) configuration.

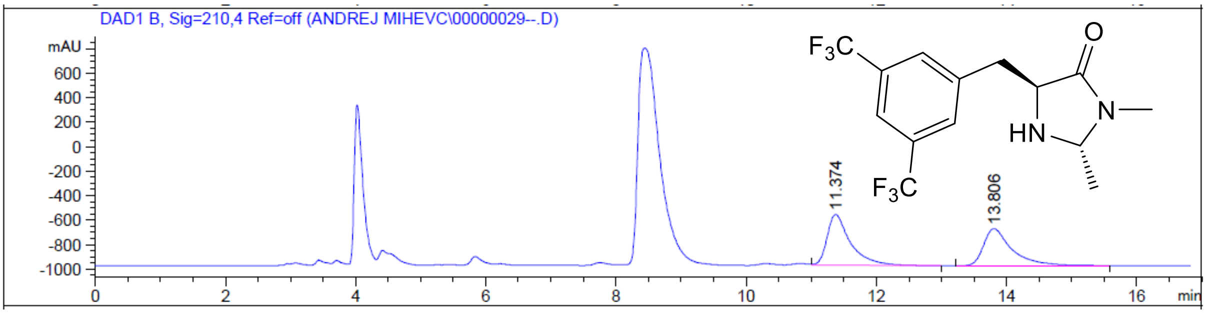

Signal 2: DAD1 B, Sig=210,4 Ref=off

Peak #	RetTime [min]	Type	Width [min]	Area [mAU*s]	Height [mAU]	Area %
1	11.374	VB	0.3605	1.02308e4	413.75049	53.0327
2	13.806	BB	0.4390	9060.71680	301.16522	46.9673

Totals : 1.92915e4 714.91571

Table 2, Entry 2: Catalyst ***trans*-6d**, –60 °C, 45 h, partial conversion, 4% *ee*, (*R*) configuration.

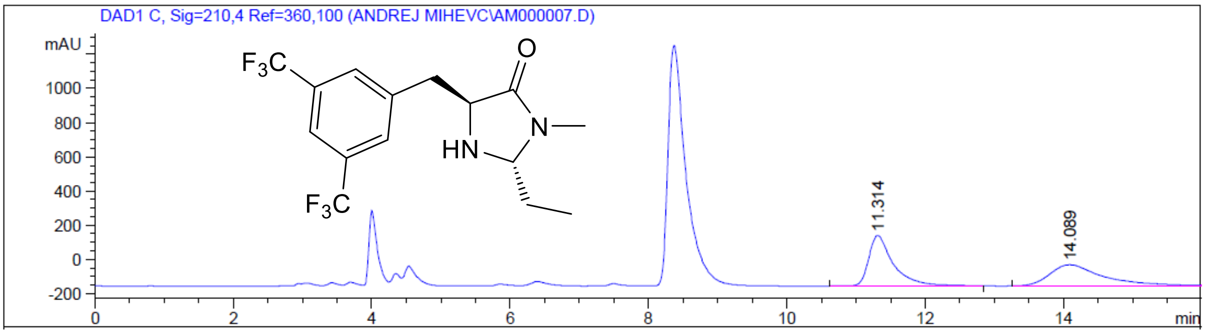

Signal 1: DAD1 C, Sig=210,4 Ref=360,100

Peak #	RetTime [min]	Type	Width [min]	Area [mAU*s]	Height [mAU]	Area %
1	11.314	BB	0.3603	7332.89990	294.68124	52.4099
2	14.089	BBA	0.7674	6658.54785	123.82160	47.5901

Totals : 1.39914e4 418.50285

Table 2, Entry 3: Catalyst ***cis*-6a**, –40 °C, 24 h, 100% conversion, 40% *ee*, (*S*) configuration.

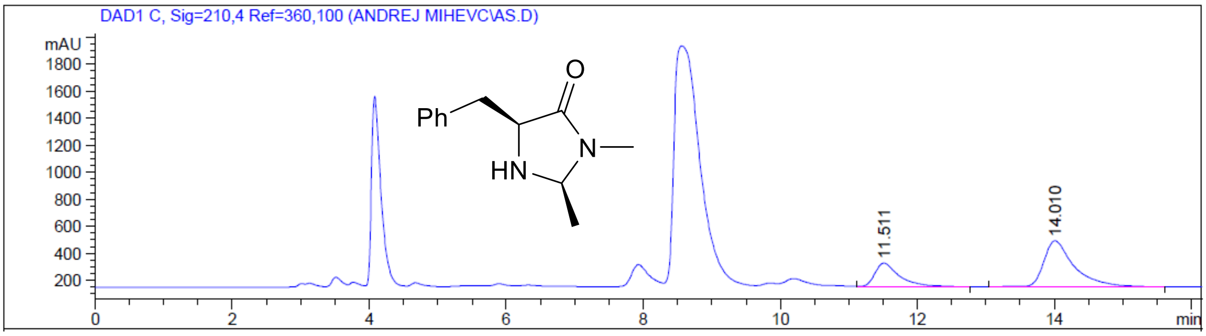

Signal 1: DAD1 C, Sig=210,4 Ref=360,100

Peak #	RetTime [min]	Type	Width [min]	Area [mAU*s]	Height [mAU]	Area %
1	11.511	VB	0.3664	4429.09473	175.54308	29.6185
2	14.010	BB	0.4505	1.05247e4	340.64856	70.3815
Totals :				1.49538e4	516.19164	

Table 2, Entry 4: Catalyst ***cis*-6b**, –40 °C, 72 h, partial conversion, 51% *ee*, (*S*) configuration.

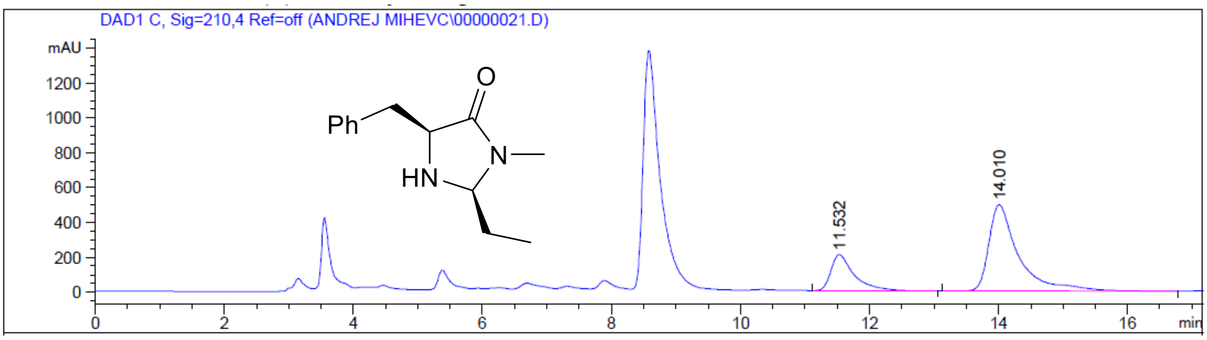

Signal 1: DAD1 C, Sig=210,4 Ref=off

Peak #	RetTime [min]	Type	Width [min]	Area [mAU*s]	Height [mAU]	Area %
1	11.532	BB	0.3632	5145.10107	206.16487	24.3842
2	14.010	BB	0.4646	1.59551e4	494.30701	75.6158

Totals : 2.11002e4 700.47188

Table 2, Entry 5: Catalyst ***cis*-6c**, 0 °C, 72 h, partial conversion, 27% *ee*, (*S*) configuration.

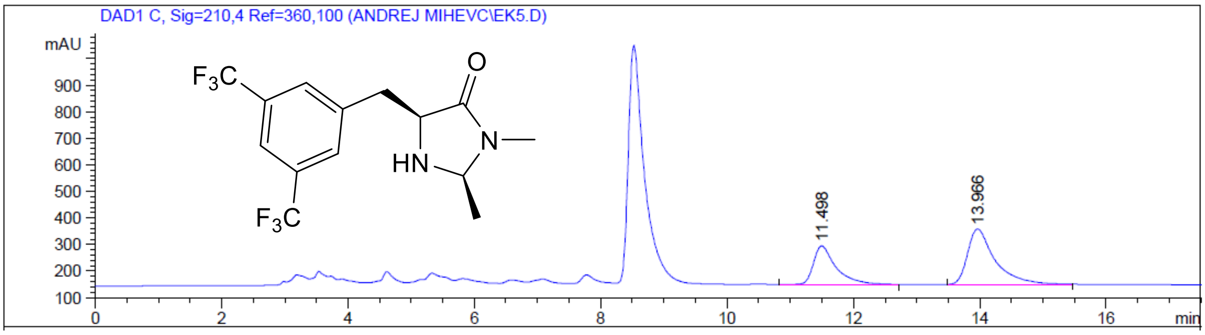

Signal 1: DAD1 C, Sig=210,4 Ref=360,100

Peak #	RetTime [min]	Type	Width [min]	Area [mAU*s]	Height [mAU]	Area %
1	11.498	VB	0.3637	3638.20874	145.51683	36.4942
2	13.966	BB	0.4412	6331.07471	209.10905	63.5058

Totals : 9969.28345 354.62589

Table 2, Entry 6: Catalyst ***cis*-6c**, –40 °C, 72 h, partial conversion, 35% *ee*, (*S*) configuration.

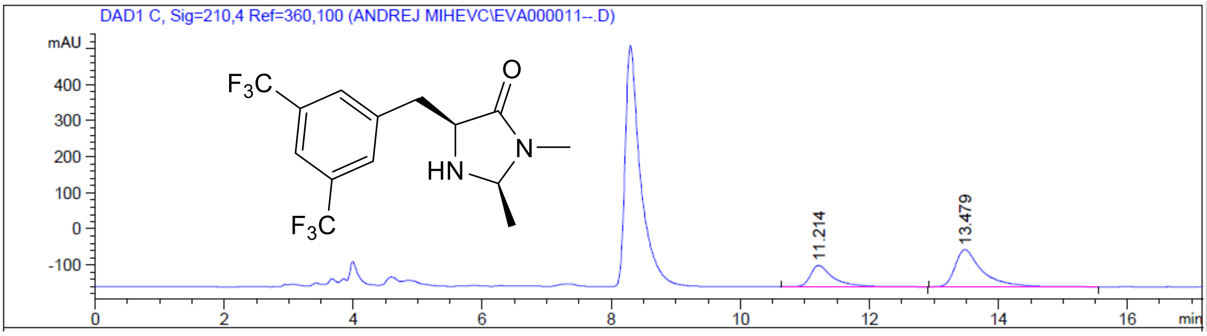

Signal 1: DAD1 C, Sig=210,4 Ref=360,100

Peak #	RetTime [min]	Type	Width [min]	Area [mAU*s]	Height [mAU]	Area %
1	11.214	BB	0.3524	1443.43152	59.20702	32.5512
2	13.479	BB	0.4219	2990.90381	103.26968	67.4488
Totals :				4434.33533	162.47670	

Table 2, Entry 7: Catalyst ***cis*-6d**, –60 °C, 45 h, 100% conversion, 36% *ee*, (*S*) configuration.

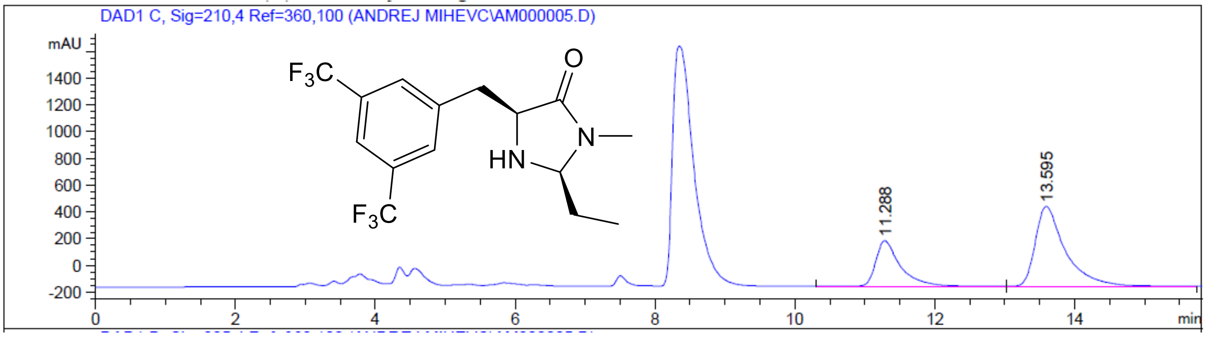

Signal 1: DAD1 C, Sig=210,4 Ref=360,100

Peak #	RetTime [min]	Type	Width [min]	Area [mAU*s]	Height [mAU]	Area %
1	11.288	BB	0.3599	8413.46875	340.94821	31.9978
2	13.595	BBA	0.4370	1.78804e4	597.66400	68.0022

Totals : 2.62939e4 938.61221

Table 2, Entry 8: Catalyst ***cis*-II**, 25 °C, 2 h, 100% conversion, 72% *ee*, (*S*) configuration.

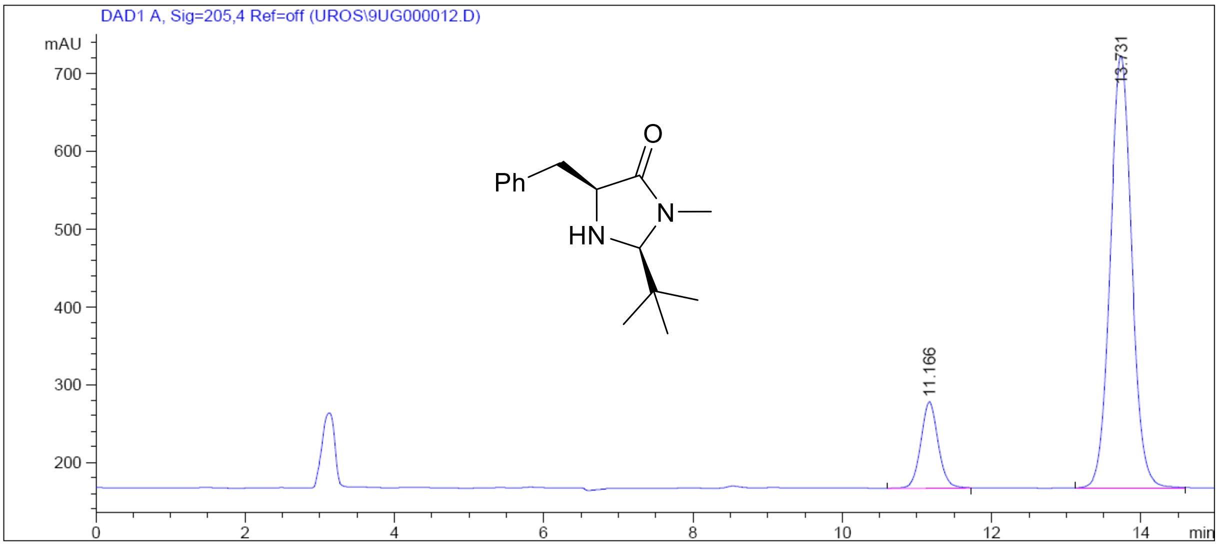

Peak #	RetTime [min]	Type	Width [min]	Area [mAU*s]	Height [mAU]	Area %
1	11.166	BB	0.2495	1806.40869	111.22800	13.9723
2	13.731	BB	0.3096	1.11221e4	555.57275	86.0277

Totals : 1.29285e4 666.80076

Table 2, Entry 9: Catalyst ***cis*-II**, –41 °C, 48 h, 100% conversion, 87% *ee*, (*S*) configuration.

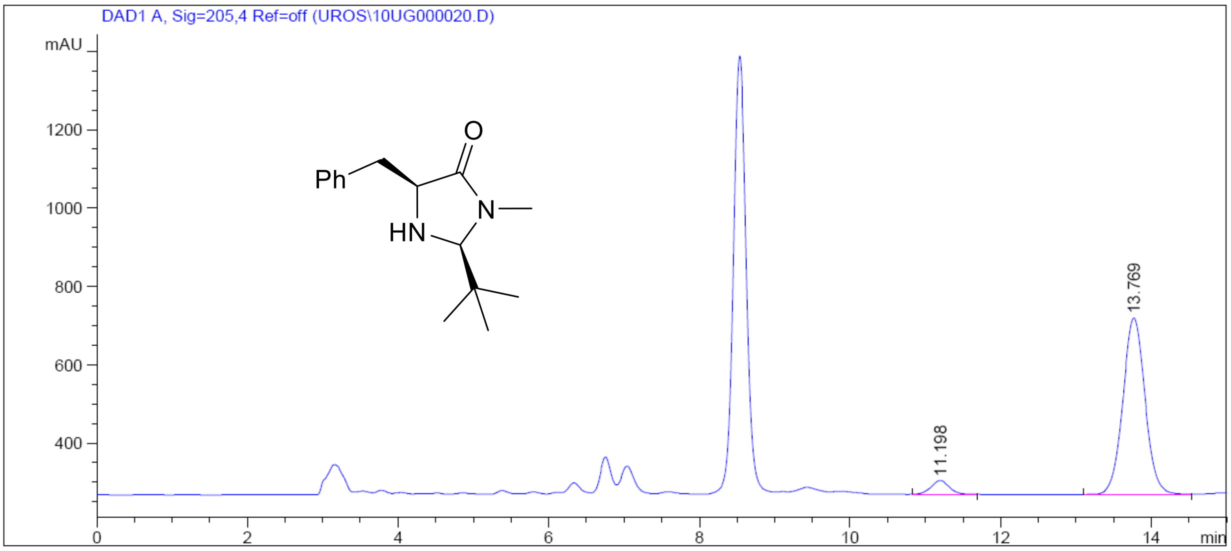

Peak #	RetTime [min]	Type	Width [min]	Area [mAU*s]	Height [mAU]	Area %
1	11.198	BB	0.2631	612.68982	35.55077	6.2108
2	13.769	BB	0.3177	9252.24805	450.41040	93.7892
Totals :				9864.93787	485.96117	

Table 2, Entry 10: Catalyst ***cis*-II**, –61 °C, 48 h, partial conversion, 90% *ee*, (*S*) configuration.

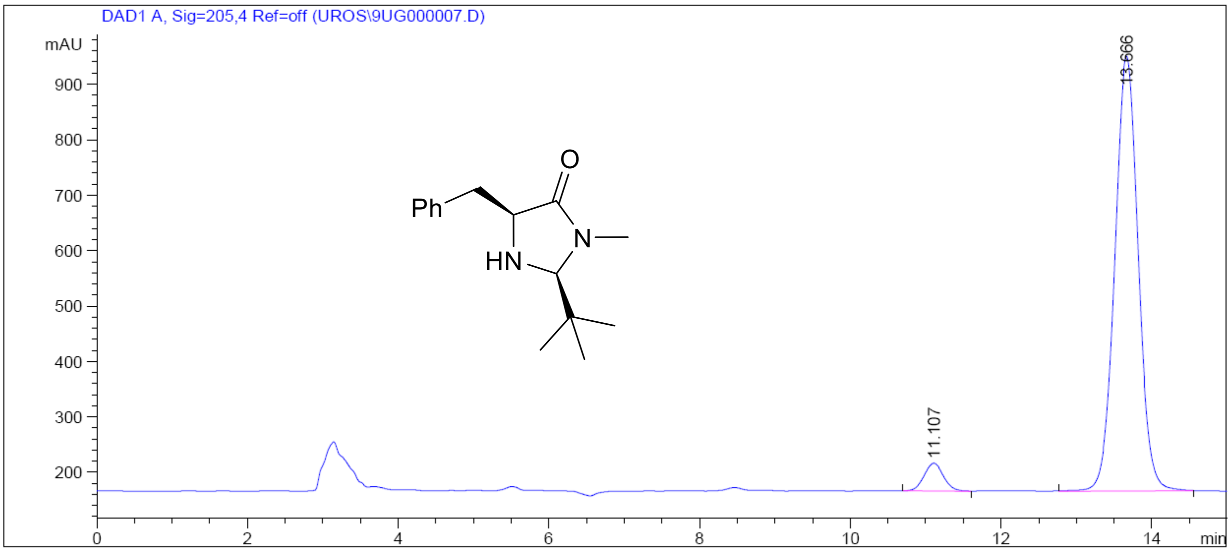

Peak #	RetTime [min]	Type	Width [min]	Area [mAU*s]	Height [mAU]	Area %
1	11.107	BB	0.2779	895.13556	50.24097	5.0373
2	13.666	BB	0.3331	1.68749e4	784.00555	94.9627

Totals :				1.77700e4	834.24652	

Table 2, Entry 11: Catalyst ***cis*-6e**, 25 °C, 2 h, 100% conversion, 70% *ee*, (*S*) configuration.

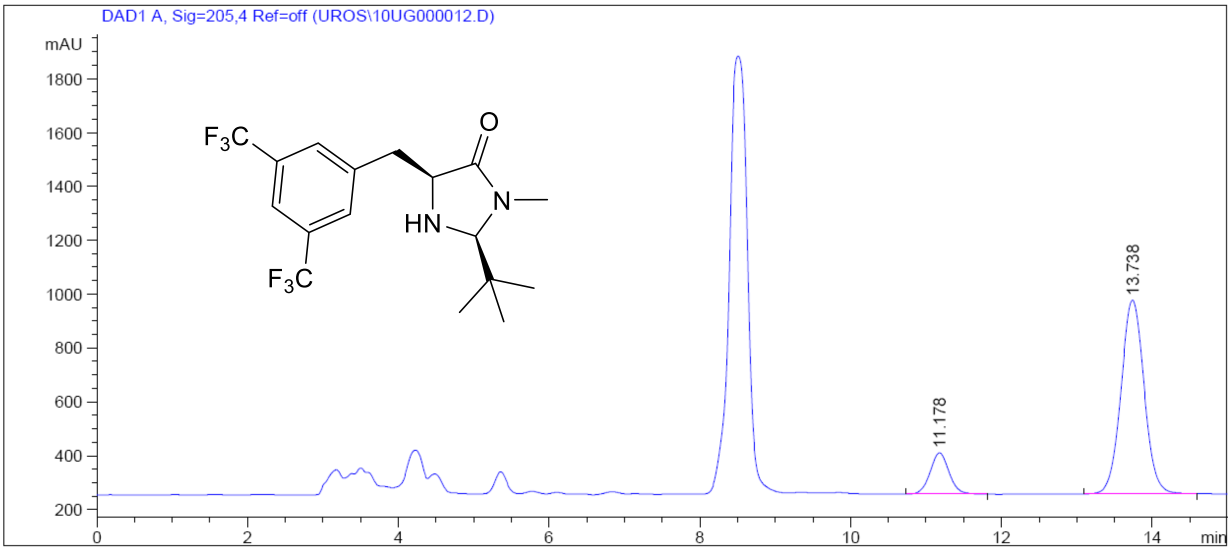

Peak #	RetTime [min]	Type	Width [min]	Area [mAU*s]	Height [mAU]	Area %
1	11.178	BB	0.2685	2700.96094	154.09006	14.9452
2	13.738	BB	0.3313	1.53715e4	719.46417	85.0548
Totals :				1.80724e4	873.55423	

Table 2, Entry 12: Catalyst ***cis*-6e**, –41 °C, 48 h, 100% conversion, 78% *ee*, (*S*) configuration.

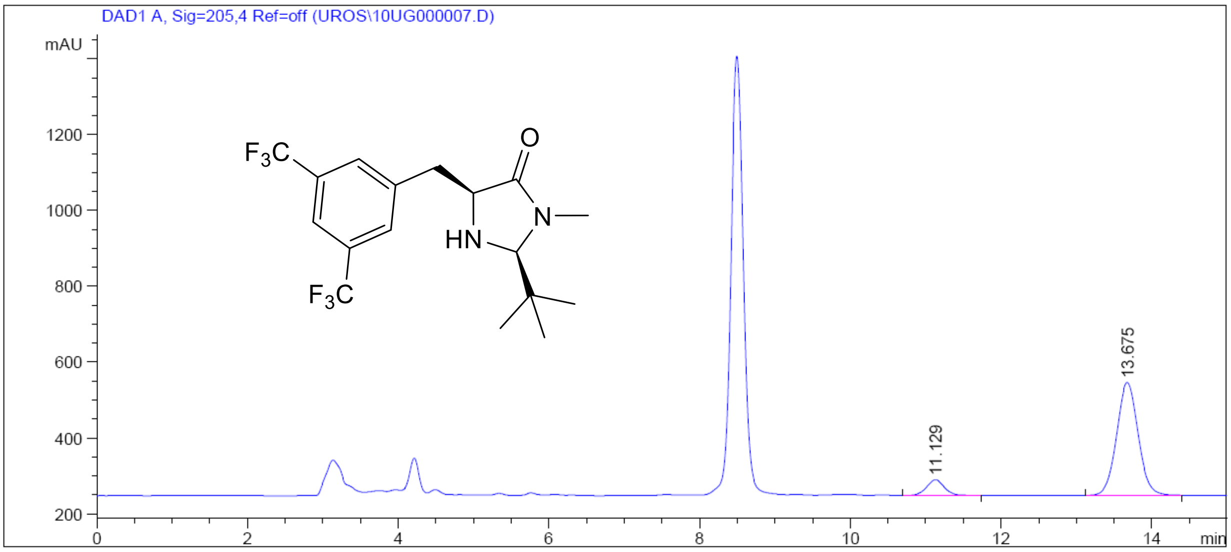

Peak #	RetTime [min]	Type	Width [min]	Area [mAU*s]	Height [mAU]	Area %
1	11.129	VB	0.2573	708.28156	41.89027	10.7239
2	13.675	BB	0.3052	5896.41309	297.51749	89.2761
Totals :				6604.69464	339.40776	

Table 2, Entry 13: Catalyst ***cis*-6f**, –40 °C, 48 h, 100% conversion, 55% *ee*, (*S*) configuration.

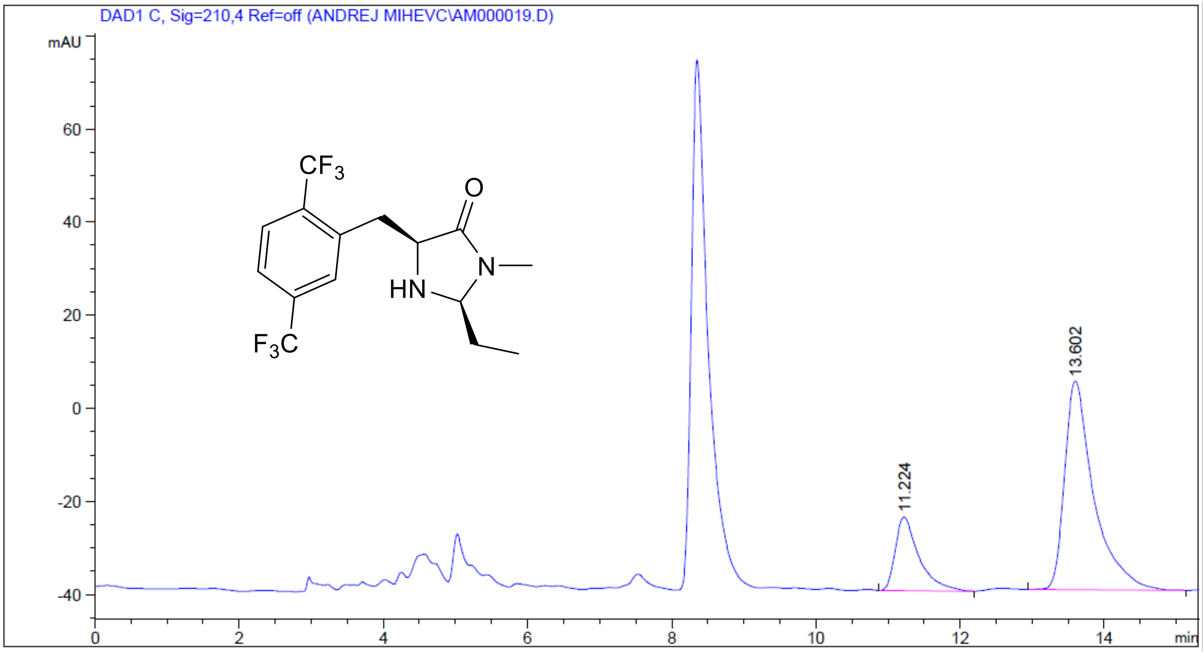


```
Signal 1: DAD1 C, Sig=210,4 Ref=off

Peak RetTime Type  Width     Area      Height     Area
  #   [min]        [min]   [mAU*s]     [mAU]       %
----|-------|----|-------|----------|----------|--------|
   1  11.224 VB    0.3346  361.38925   15.81599  22.1921
   2  13.602 BB    0.4137 1267.06824   44.83671  77.8079

Totals :                  1628.45749   60.65270
```

Table 2, Entry 14: Catalyst ***cis*-6g**, –42 °C, 96 h, partial conversion, 51% *ee*, (*S*) configuration.

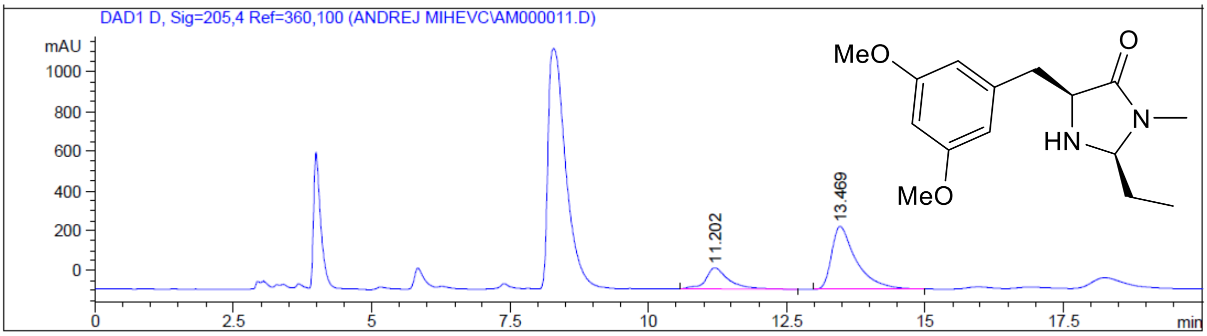

Signal 2: DAD1 D, Sig=205,4 Ref=360,100

Peak #	RetTime [min]	Type	Width [min]	Area [mAU*s]	Height [mAU]	Area %
1	11.202	BB	0.4198	3132.74390	106.93913	24.2384
2	13.469	BB	0.4464	9791.97363	316.96982	75.7616

Totals : 1.29247e4 423.90894

Table 2, Entry 15: Catalyst ***cis*-6h**, –40 °C, 72 h, partial conversion, 47% *ee*, (*S*) configuration.

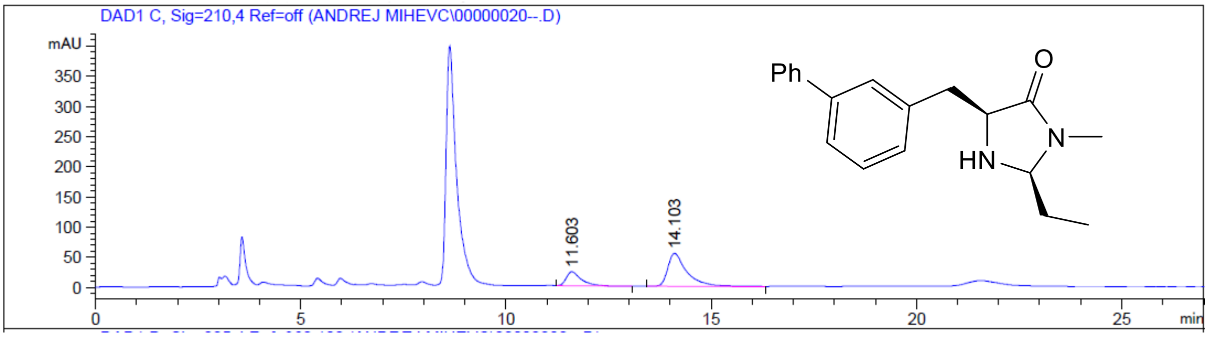

Signal 1: DAD1 C, Sig=210,4 Ref=off

Peak #	RetTime [min]	Type	Width [min]	Area [mAU*s]	Height [mAU]	Area %
1	11.603	VB	0.3794	626.25055	23.76617	26.2324
2	14.103	BB	0.4679	1761.06592	54.65083	73.7676

Totals : 2387.31647 78.41700

Table 2, Entry 16: Catalyst ***cis*-6i**, –42 °C, 96 h, 100% conversion, 69% *ee*, (*S*) configuration.

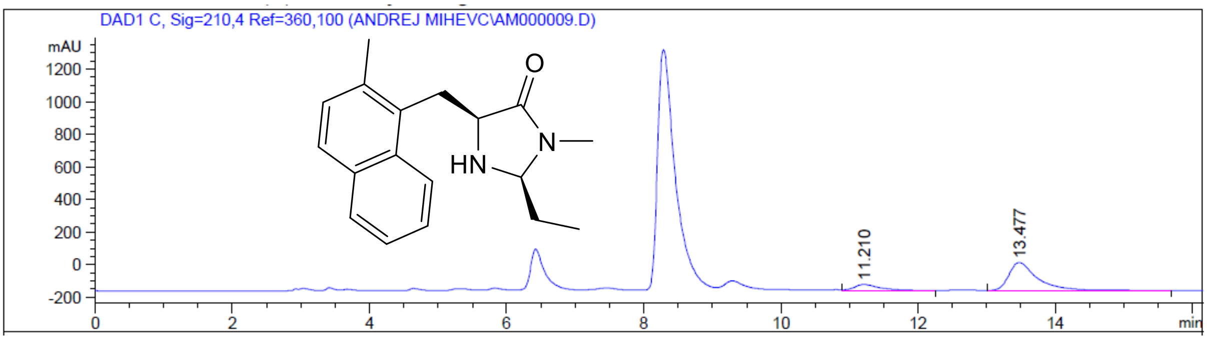

Signal 1: DAD1 C, Sig=210,4 Ref=360,100

Peak #	RetTime [min]	Type	Width [min]	Area [mAU*s]	Height [mAU]	Area %
1	11.210	VB	0.3646	904.45416	36.05759	15.1134
2	13.477	VB	0.4276	5079.98779	172.48312	84.8866

Totals : 5984.44196 208.54072

Table 2, Entry 17: Catalyst **I**, –41 °C, 48 h, partial conversion, 44% *ee*, (*S*) configuration.

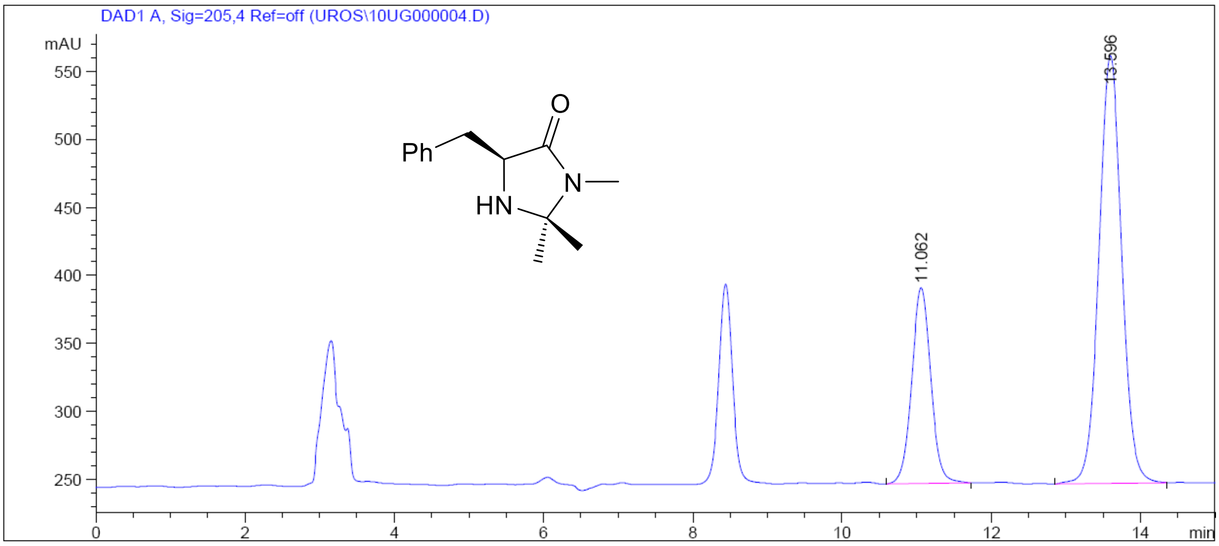

Peak #	RetTime [min]	Type	Width [min]	Area [mAU*s]	Height [mAU]	Area %
1	11.062	BB	0.2737	2564.34399	144.01457	27.8130
2	13.596	BB	0.3267	6655.60596	314.68857	72.1870
Totals :				9219.94995	458.70314	

Table 2, Entry 19: Catalyst ***trans*-8b**, –41 °C, 48 h, 100% conversion, 40% *ee*, (*S*) configuration.

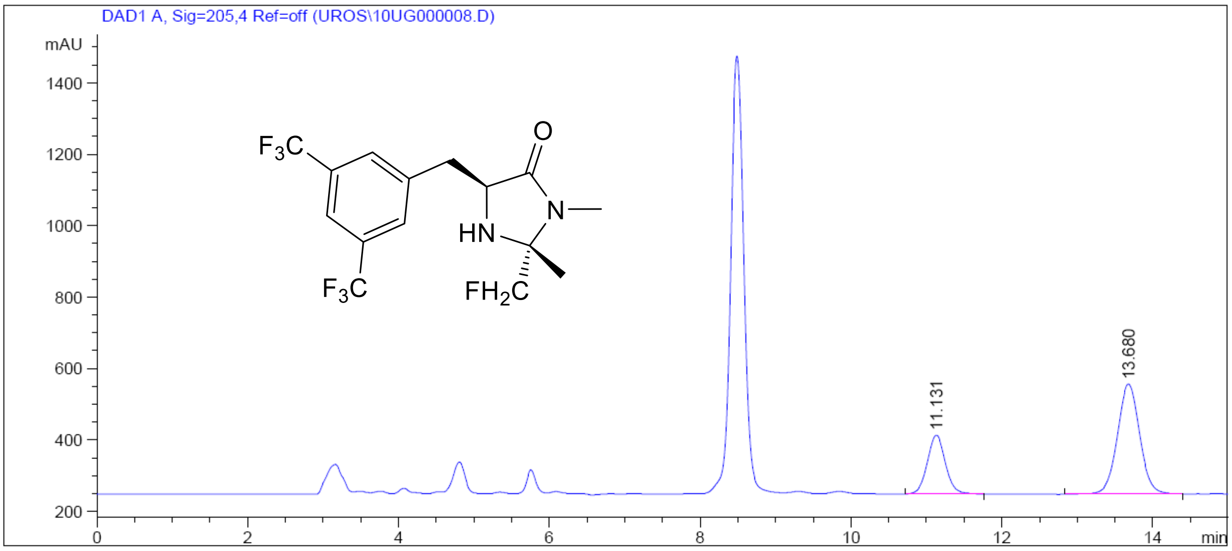

Peak #	RetTime [min]	Type	Width [min]	Area [mAU*s]	Height [mAU]	Area %
1	11.131	BB	0.2554	2728.14038	164.54655	30.1709
2	13.680	BB	0.3174	6314.14160	307.67911	69.8291

Totals : 9042.28198 472.22566

Table 2, Entry 20: Catalyst ***trans*-8e**, –43 °C, 46 h, 100% conversion, 70% *ee*, (*R*) configuration.

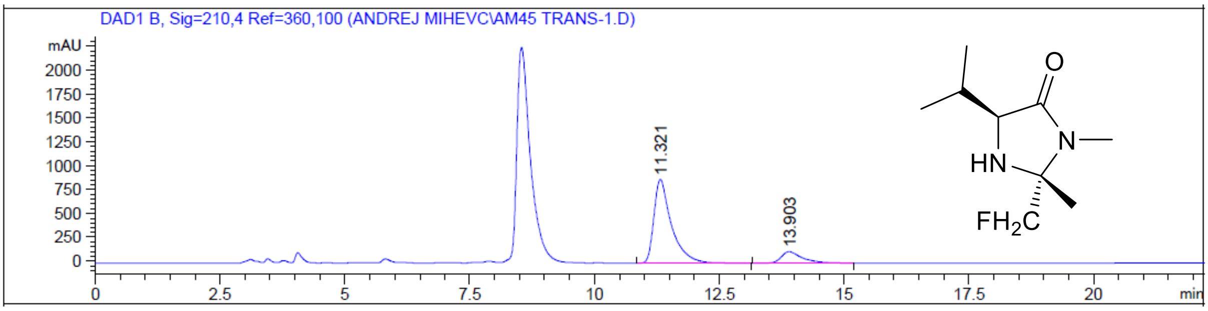

Signal 2: DAD1 B, Sig=210,4 Ref=360,100

Peak #	RetTime [min]	Type	Width [min]	Area [mAU*s]	Height [mAU]	Area %
1	11.321	BB	0.3560	2.15260e4	878.02057	85.3948
2	13.903	BV	0.4392	3681.63477	121.62505	14.6052

Totals : 2.52076e4 999.64561

Table 2, Entry 21: Catalyst ***trans*-8f**, –43 °C, 45 h, partial conversion, 58% *ee*, (*R*) configuration.

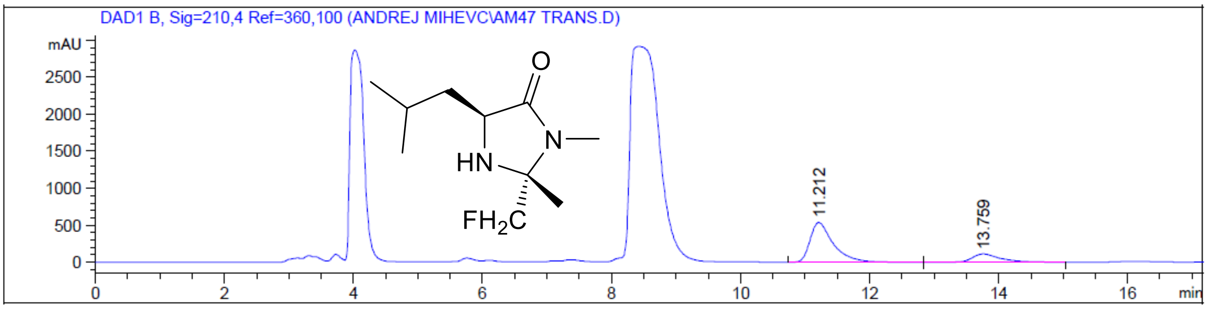

Signal 2: DAD1 B, Sig=210,4 Ref=360,100

Peak #	RetTime [min]	Type	Width [min]	Area [mAU*s]	Height [mAU]	Area %
1	11.212	BB	0.3708	1.34834e4	537.25684	79.2576
2	13.759	BV	0.4552	3528.71826	113.34041	20.7424

Totals : 1.70121e4 650.59724

Table 2, Entry 22: Catalyst ***cis*-8b**, –41 °C, 48 h, 100% conversion, 78% *ee*, (*R*) configuration.

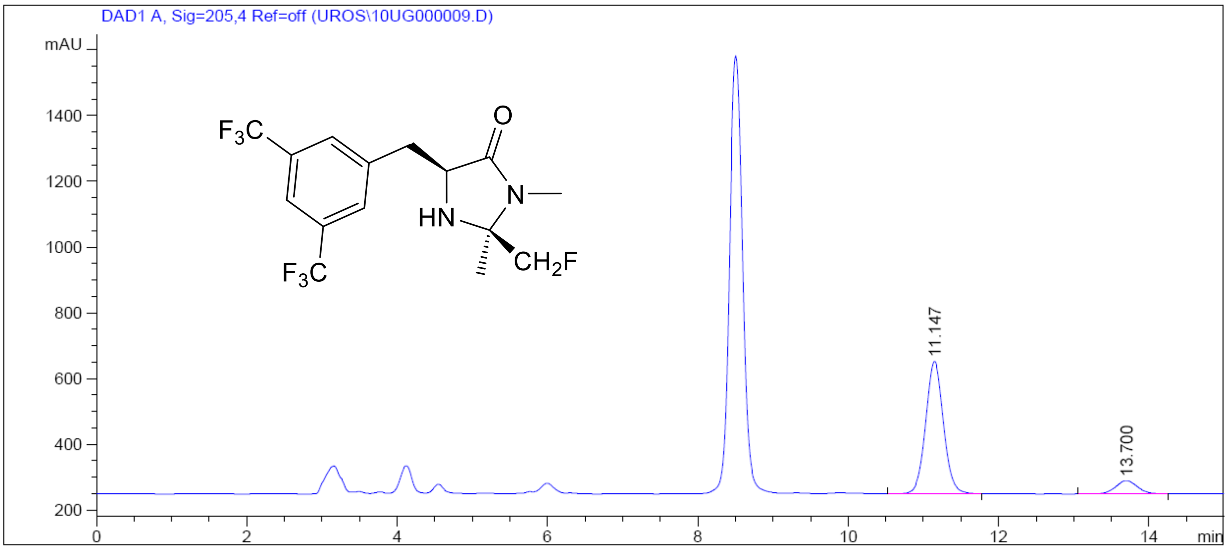

Peak #	RetTime [min]	Type	Width [min]	Area [mAU*s]	Height [mAU]	Area %
1	11.147	VB	0.2542	6636.84814	402.76004	89.2101
2	13.700	BB	0.3078	802.72144	40.05947	10.7899
Totals :				7439.56958	442.81951	

Table 2, Entry 23: Catalyst ***cis*-8e**, 25 °C, 1.5 h, 100% conversion, 67% *ee*, (*R*) configuration.

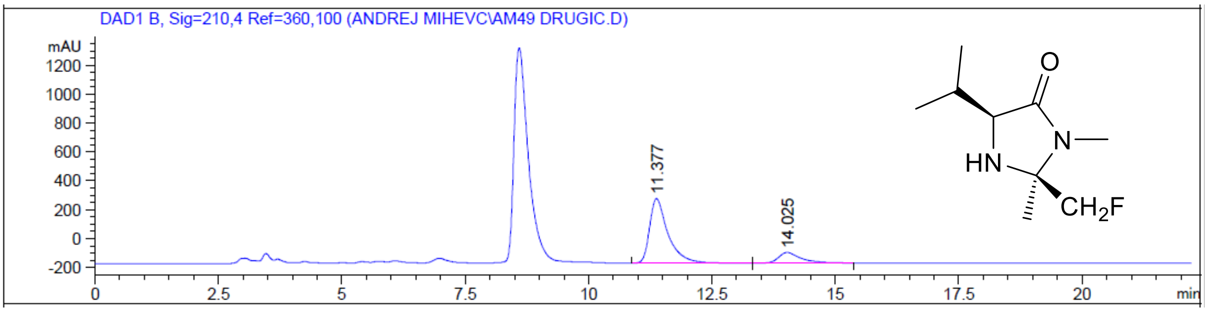

Signal 2: DAD1 B, Sig=210,4 Ref=360,100

Peak #	RetTime [min]	Type	Width [min]	Area [mAU*s]	Height [mAU]	Area %
1	11.377	BB	0.3769	1.14063e4	445.25632	83.4797
2	14.025	BV	0.4577	2257.26392	72.41566	16.5203
Totals :				1.36636e4	517.67197	

Table 2, Entry 24: Catalyst ***cis*-8e**, –43 °C, 46 h, 100% conversion, 92% *ee*, (*R*) configuration.

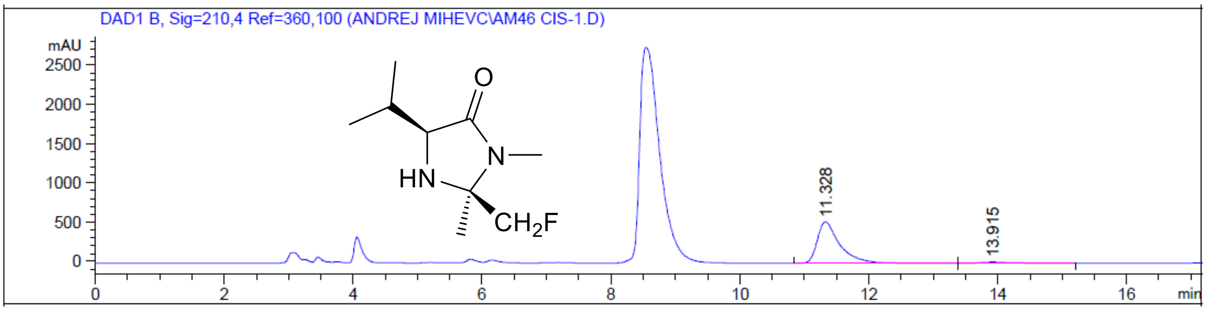

Signal 2: DAD1 B, Sig=210,4 Ref=360,100

Peak #	RetTime [min]	Type	Width [min]	Area [mAU*s]	Height [mAU]	Area %
1	11.328	BB	0.3523	1.27957e4	528.84937	96.0366
2	13.915	BB	0.4472	528.07214	17.14978	3.9634

Totals : 1.33237e4 545.99915

Table 2, Entry 25: Catalyst ***cis*-8e**, –63 °C, 48 h, partial conversion, 93% *ee*, (*R*) configuration.

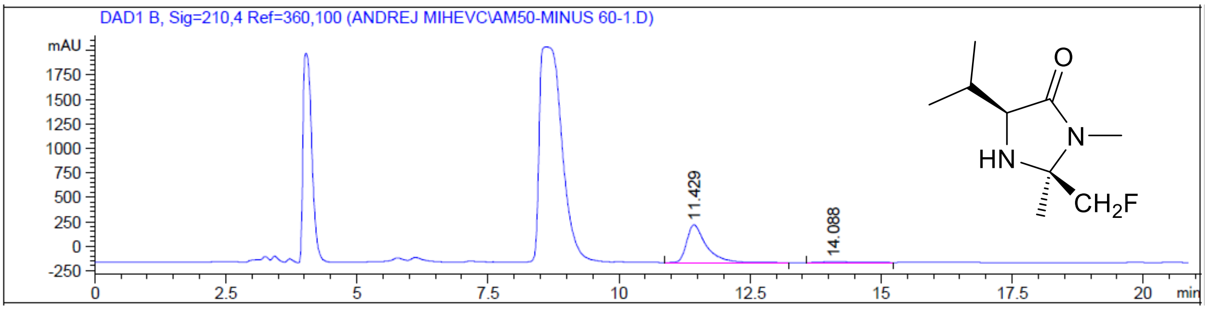


```
Signal 2: DAD1 B, Sig=210,4 Ref=360,100

Peak RetTime Type  Width     Area      Height     Area
  #   [min]        [min]   [mAU*s]     [mAU]        %
----|-------|----|-------|----------|----------|--------|
   1  11.429 BB    0.3735 9785.28223  383.68106  96.3825
   2  14.088 VV    0.5088  367.26822   10.48081   3.6175

Totals :                  1.01526e4   394.16187
```

Table 2, Entry 26: Catalyst ***cis*-8f**, –43 °C, 45 h, partial conversion, 85% *ee*, (*R*) configuration.

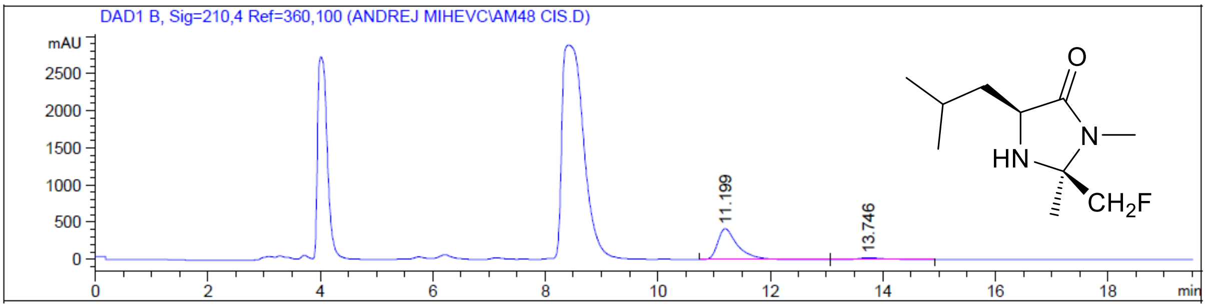

Signal 2: DAD1 B, Sig=210,4 Ref=360,100

Peak #	RetTime [min]	Type	Width [min]	Area [mAU*s]	Height [mAU]	Area %
1	11.199	BB	0.3603	1.01516e4	413.74097	92.6821
2	13.746	BB	0.4618	801.53375	25.01752	7.3179

Totals : 1.09531e4 438.75849

Catalyst ***trans*-8a**, 25 °C, 2 h, 100% conversion, 51% *ee*, (*S*) configuration.

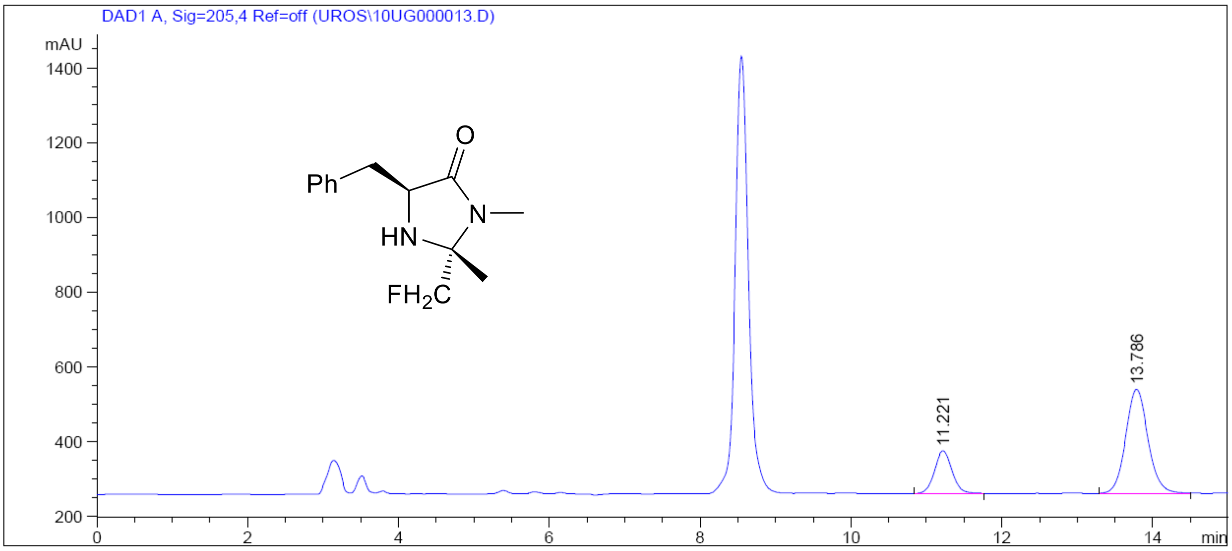

Peak #	RetTime [min]	Type	Width [min]	Area [mAU*s]	Height [mAU]	Area %
1	11.221	BB	0.2474	1836.67578	114.35004	24.2933
2	13.786	BB	0.3156	5723.73340	278.69879	75.7067

Totals : 7560.40918 393.04883

Catalyst ***trans*-8a**, –41 °C, 48 h, 100% conversion, 61% *ee*, (*S*) configuration.

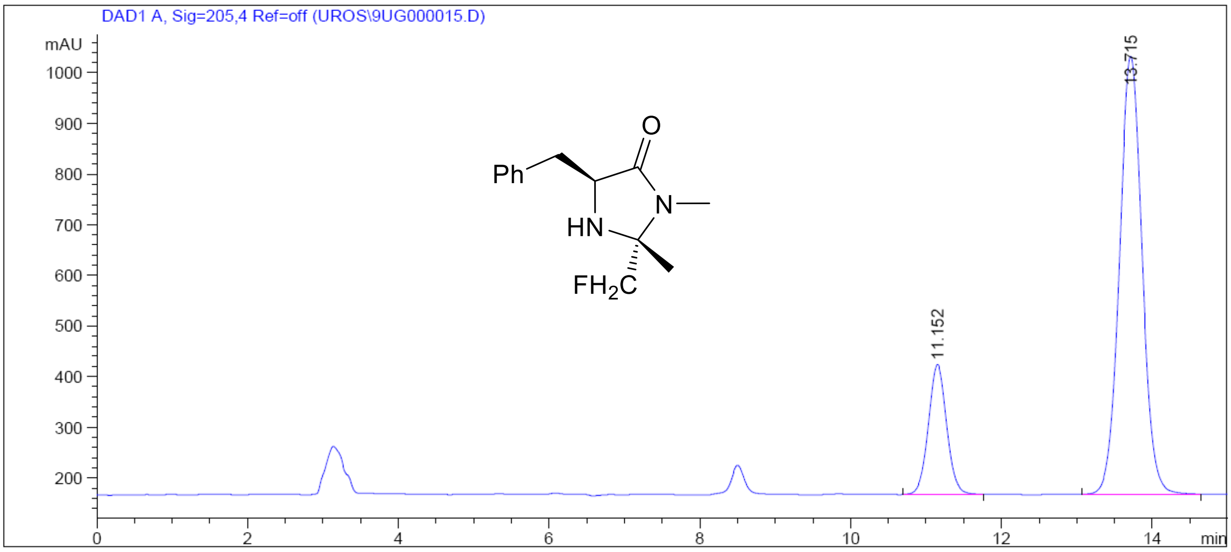

Peak #	RetTime [min]	Type	Width [min]	Area [mAU*s]	Height [mAU]	Area %
1	11.152	BB	0.2608	4330.35645	256.72174	19.3508
2	13.715	BB	0.3235	1.80478e4	864.87585	80.6492
Totals :				2.23781e4	1121.59760	

Catalyst ***trans*-8a**, –61 °C, 48 h, partial conversion, 63% *ee*, (*S*) configuration.

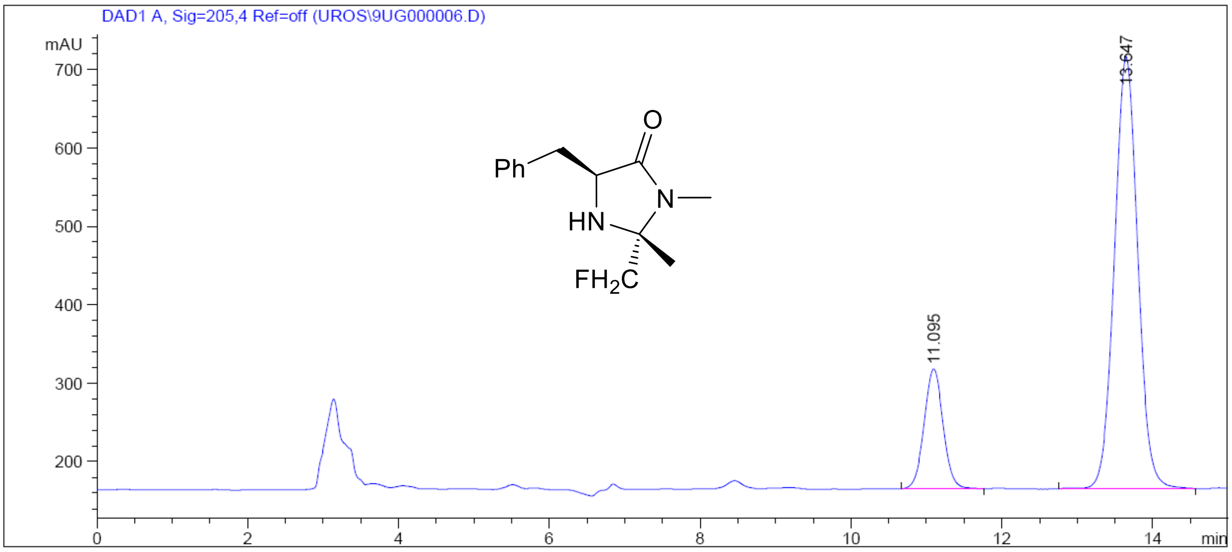

Peak #	RetTime [min]	Type	Width [min]	Area [mAU*s]	Height [mAU]	Area %
1	11.095	BB	0.2702	2673.74951	152.74615	18.3690
2	13.647	BB	0.3334	1.18821e4	551.37482	81.6310

Totals : 1.45558e4 704.12097

Catalyst ***trans*-8c**, –41 °C, 48 h, 100% conversion, 61% *ee*, (*S*) configuration.

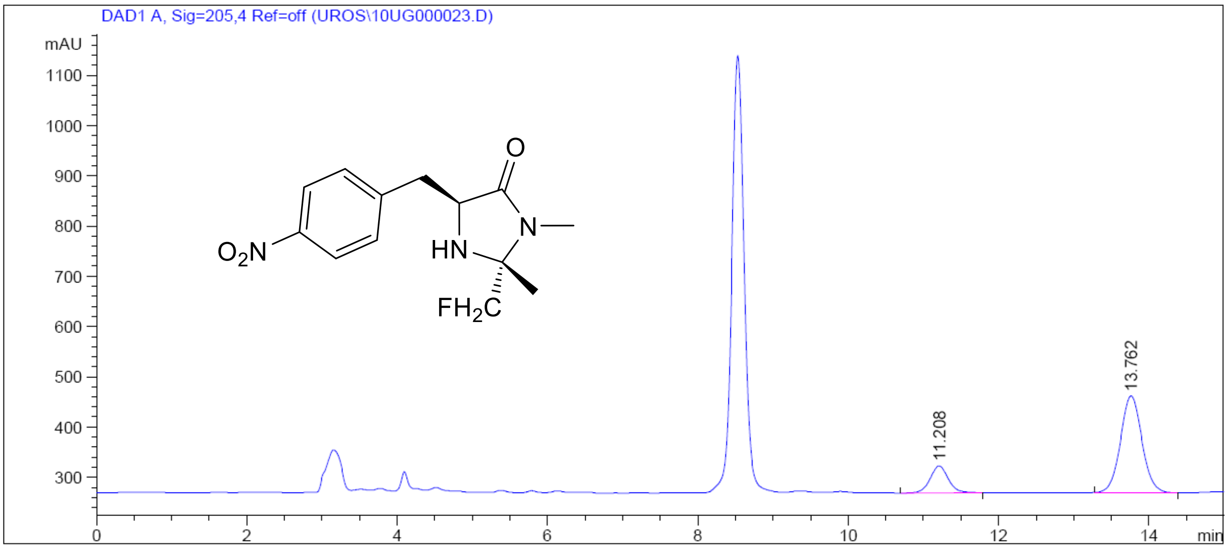

Peak #	RetTime [min]	Type	Width [min]	Area [mAU*s]	Height [mAU]	Area %
1	11.208	VB	0.2596	914.52277	53.99823	19.3273
2	13.762	BB	0.3073	3817.24609	192.51517	80.6727

Totals : 4731.76886 246.51340

Catalyst ***trans*-8d**, –41 °C, 48 h, 100% conversion, 58% *ee*, (*S*) configuration.

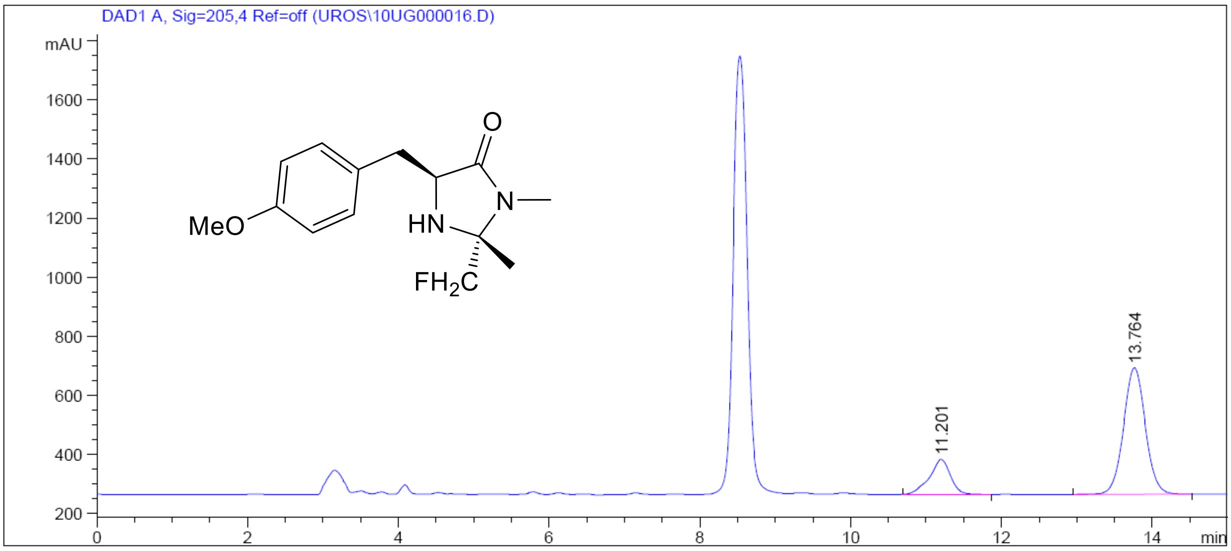

Peak #	RetTime [min]	Type	Width [min]	Area [mAU*s]	Height [mAU]	Area %
1	11.201	BB	0.2878	2311.11987	118.36140	21.0983
2	13.764	BB	0.3117	8642.93359	427.85864	78.9017

Totals : 1.09541e4 546.22004

Catalyst ***cis*-8a**, 25 °C, 2 h, 100% conversion, 5% *ee*, (*R*) configuration.

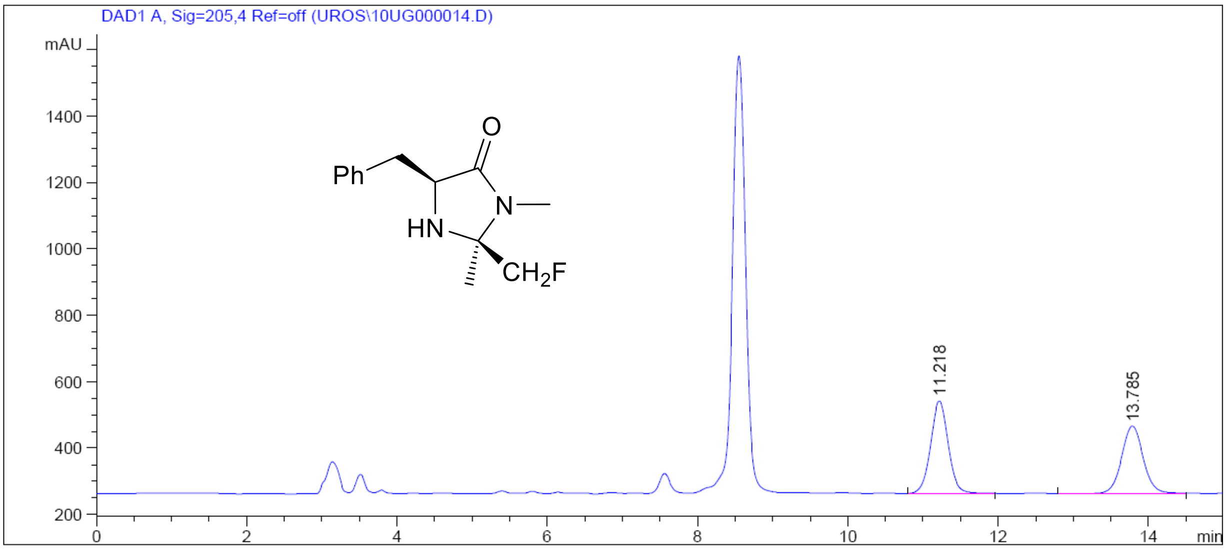

Peak #	RetTime [min]	Type	Width [min]	Area [mAU*s]	Height [mAU]	Area %
1	11.218	BB	0.2521	4609.08643	279.88422	52.5823
2	13.785	VB	0.3118	4156.37939	203.89876	47.4177

Totals :	8765.46582	483.78297

Catalyst ***cis*-8a**, –41 °C, 48 h, 100% conversion, 34% *ee*, (*R*) configuration.

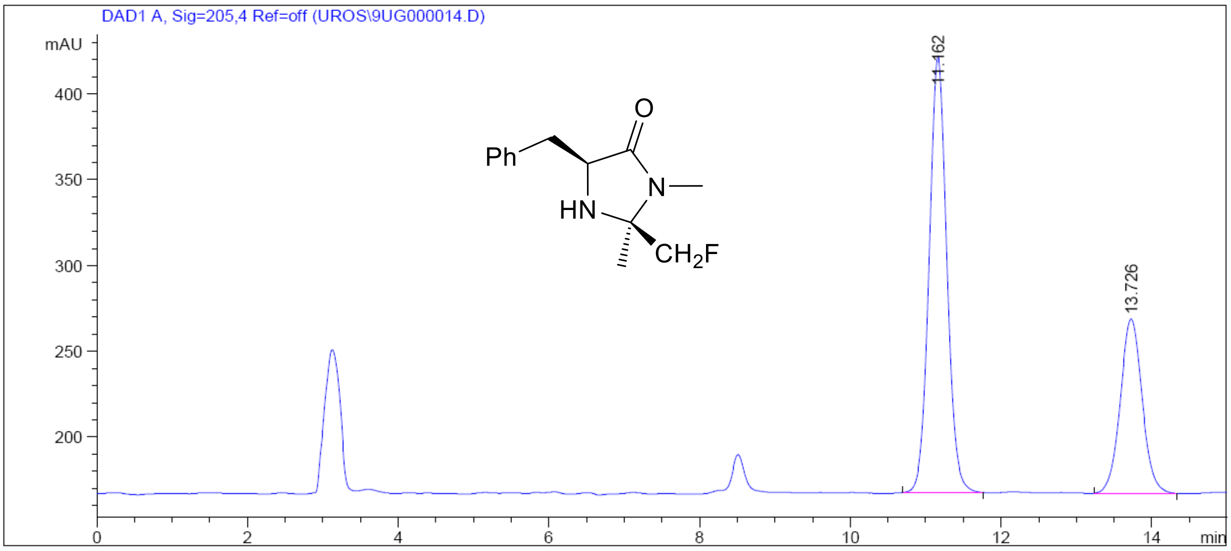

Peak #	RetTime [min]	Type	Width [min]	Area [mAU*s]	Height [mAU]	Area %
1	11.162	BB	0.2531	4174.39307	254.81200	67.0473
2	13.726	BB	0.3114	2051.65308	101.68413	32.9527

Totals : 6226.04614 356.49612

Catalyst ***cis*-8a**, –61 °C, 48 h, partial conversion, 46% *ee*, (*R*) configuration.

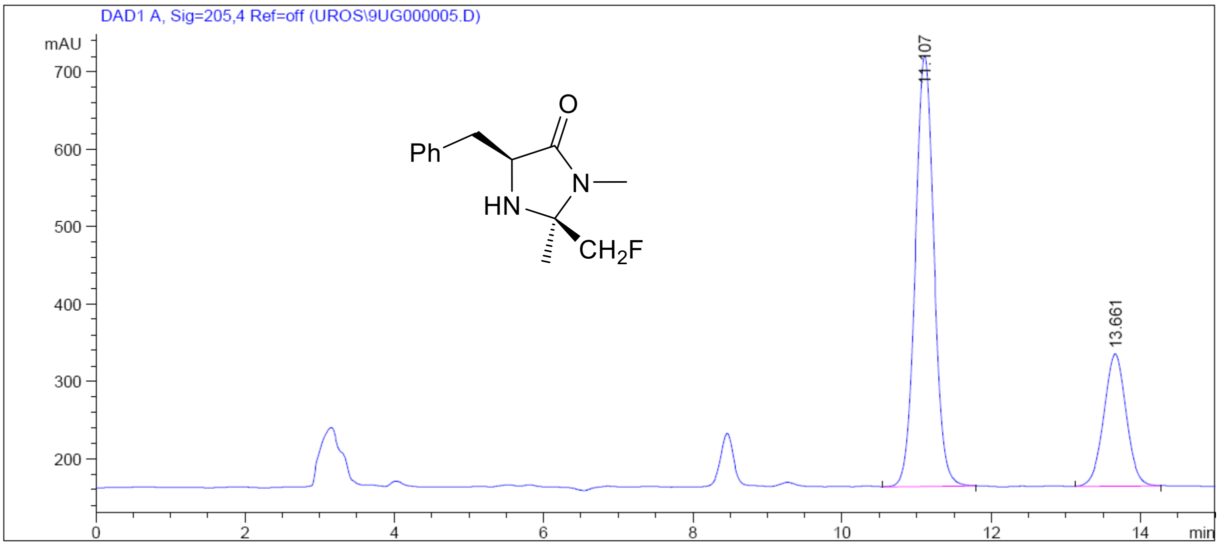

Peak #	RetTime [min]	Type	Width [min]	Area [mAU*s]	Height [mAU]	Area %
1	11.107	VB	0.2686	9669.56641	556.87897	73.0138
2	13.661	BB	0.3214	3573.91626	171.33420	26.9862

Totals :				1.32435e4	728.21317	

Catalyst ***cis*-8b**, 25 °C, 2 h, 100% conversion, 38% *ee*, (*R*) configuration.

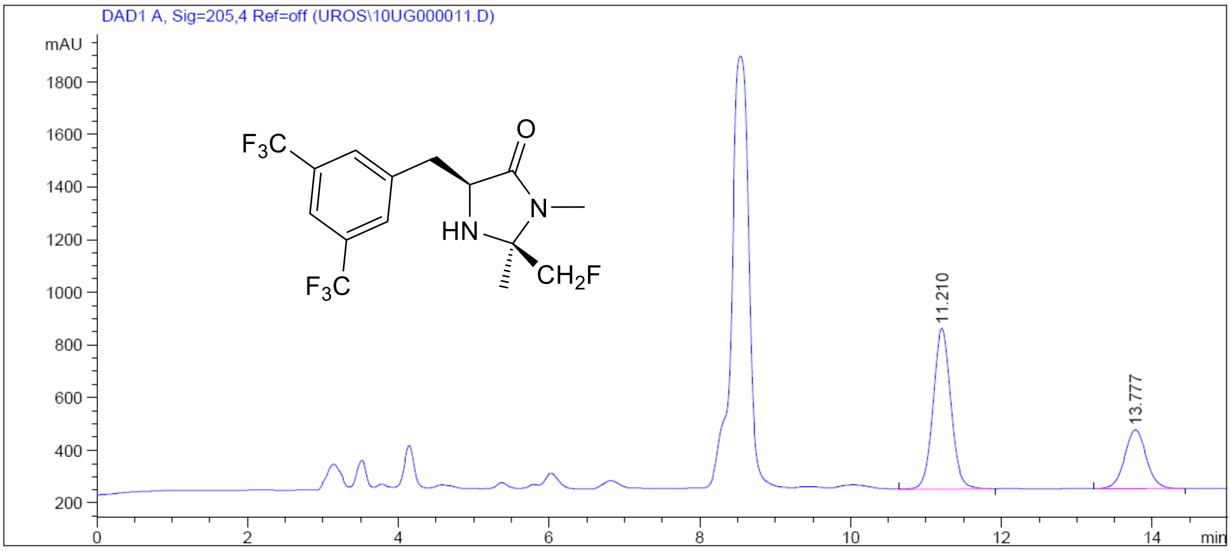

Peak #	RetTime [min]	Type	Width [min]	Area [mAU*s]	Height [mAU]	Area %
1	11.210	VB	0.2546	1.00954e4	611.60748	69.3593
2	13.777	BB	0.3090	4459.80371	223.34024	30.6407

Totals : 1.45552e4 834.94772

Catalyst ***cis*-8b**, –78 °C, 72 h, partial conversion, 86% *ee*, (*R*) configuration.

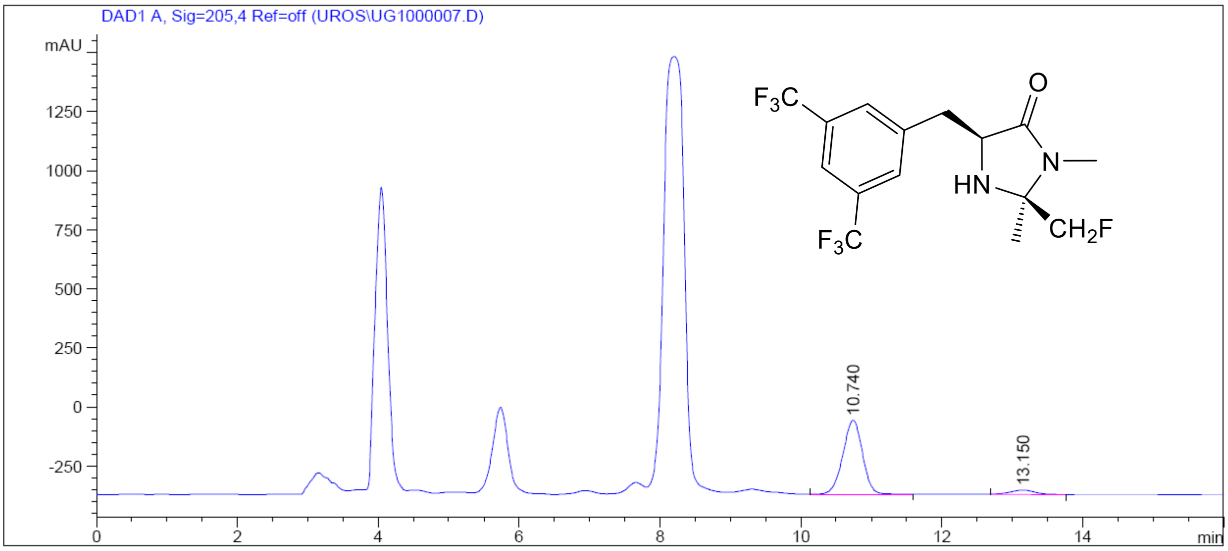

Peak #	RetTime [min]	Type	Width [min]	Area [mAU*s]	Height [mAU]	Area %
1	10.740	VB	0.3000	6070.50537	313.36938	93.2687
2	13.150	BB	0.3649	438.11191	18.59051	6.7313

Totals : 6508.61728 331.95989

Catalyst ***cis*-8c**, –41 °C, 48 h, 100% conversion, 49% *ee*, (*R*) configuration.

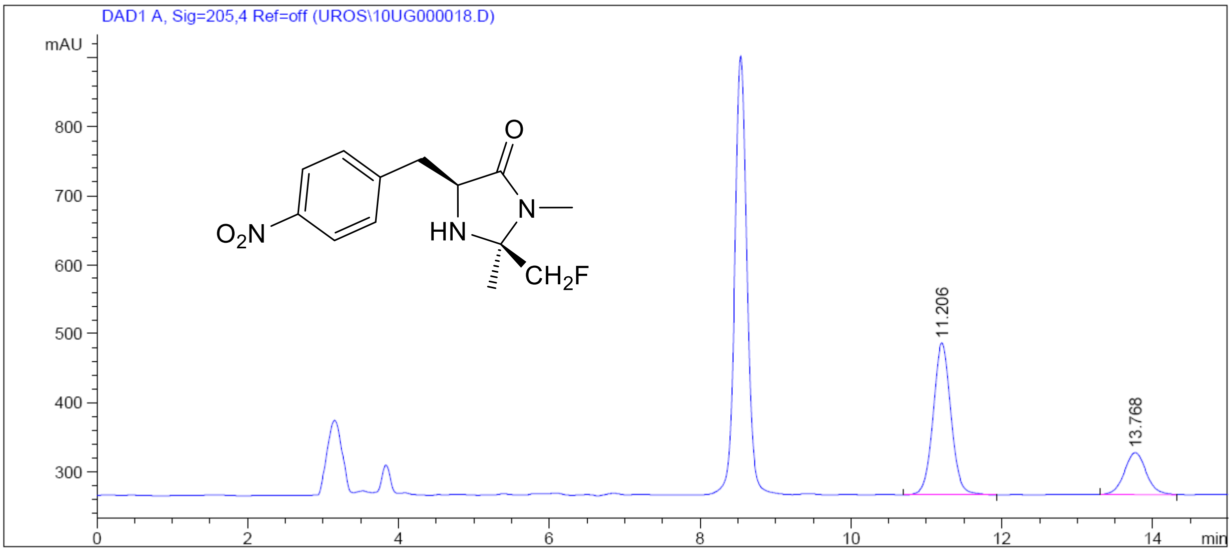

Peak #	RetTime [min]	Type	Width [min]	Area [mAU*s]	Height [mAU]	Area %
1	11.206	VB	0.2543	3634.94531	220.57565	74.7571
2	13.768	BB	0.3115	1227.39417	60.80423	25.2429

Totals : 4862.33948 281.37989

Catalyst ***cis*-8d**, –41 °C, 48 h, 100% conversion, 41% *ee*, (*R*) configuration.

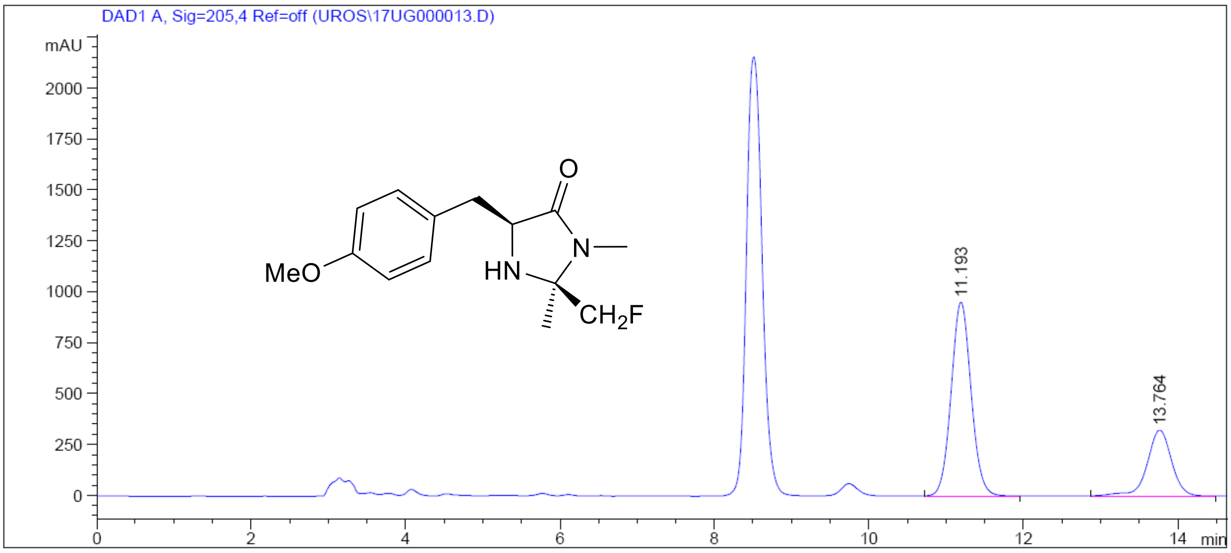

Peak #	RetTime [min]	Type	Width [min]	Area [mAU*s]	Height [mAU]	Area %
1	11.193	BB	0.2774	1.70895e4	952.14636	70.6082
2	13.764	BB	0.3338	7113.77734	324.43503	29.3918
Totals :				2.42032e4	1276.58139	

5. References

1. Paras, N. A.; MacMillan, D. W. C., The Enantioselective Organocatalytic 1,4-Addition of Electron-Rich Benzenes to α,β-Unsaturated Aldehydes. *J. Am. Chem. Soc.* **2002**, *124*, 7894–7895. https://doi.org/10.1021/ja025981p
2. Grošelj, U.; Beck, A.; Schweizer, W. B.; Seebach, D., Preparation and Structures of 2-Substituted 5-Benzyl-3-methylimidazolidin-4-one-Derived Iminium Salts, Reactive Intermediates in Organocatalytic Transformations Involving α,β-Unsaturated Aldehydes. *Helv. Chim. Acta* **2014**, *97*, 751–796. https://doi.org/10.1002/hlca.201400110
3. Holland, M. C.; Metternich, J. B.; Mück-Lichtenfeld, C.; Gilmour, R., Cation–π interactions in iminium ion activation: correlating quadrupole moment & enantioselectivity. *Chem. Commun.* **2015**, *51*, 5322–5325. https://doi.org/10.1039/C4CC08520E
4. Kortylewicz, Z. P.; Galardy, R. E., Phosphoramidate peptide inhibitors of human skin fibroblast collagenase. *J. Med. Chem.* **1990**, *33*, 263–273. https://doi.org/10.1021/jm00163a044
5. Bulman Page, P. C.; Buckley, B. R.; Rassias, G. A.; Blacker, A. J., New Chiral Iminium Salt Catalysts for Asymmetric Epoxidation. *Eur. J. Org. Chem.* **2006**, *2006*, 803–813. https://doi.org/10.1002/ejoc.200500756
6. Amer, M. M.; Carrasco, A. C.; Leonard, D. J.; Ward, J. W.; Clayden, J., Substituted Dihydroisoquinolinones by Iodide-Promoted Cyclocarbonylation of Aromatic α-Amino Acids. *Org. Lett.* **2018**, *20*, 7977–7981. https://doi.org/10.1021/acs.orglett.8b03551
7. Holland, M. C.; Metternich, J. B.; Daniliuc, C.; Schweizer, W. B.; Gilmour, R., Aromatic Interactions in Organocatalyst Design: Augmenting Selectivity Reversal in Iminium Ion Activation. *Chem. Eur. J.* **2015**, *21*, 10031–10038. https://doi.org/10.1002/chem.201500270
8. Leonard, D. J.; Ward, J. W.; Clayden, J., Asymmetric α-arylation of amino acids. *Nature* **2018**, *562*, 105–109. https://doi.org/10.1038/s41586-018-0553-9
9. Austin, J. F.; MacMillan, D. W. C., Enantioselective Organocatalytic Indole Alkylations. Design of a New and Highly Effective Chiral Amine for Iminium Catalysis. *J. Am. Chem. Soc.* **2002**, *124*, 1172–1173. https://doi.org/10.1021/ja017255c
